# Limited effects of population age on the genetic structure of spatially isolated forest herb populations in temperate Europe

**DOI:** 10.1002/ece3.10971

**Published:** 2024-02-26

**Authors:** Siyu Huang, Jannis Till Feigs, Stephanie I. J. Holzhauer, Katja Kramp, Jörg Brunet, Guillaume Decocq, Pieter De Frenne, Martin Diekmann, Jaan Liira, Fabien Spicher, Pieter Vangansbeke, Thomas Vanneste, Kris Verheyen, Tobias Naaf

**Affiliations:** ^1^ Leibniz Centre for Agricultural Landscape Research (ZALF) Müncheberg Germany; ^2^ Southern Swedish Forest Research Centre Swedish University of Agricultural Sciences Lomma Sweden; ^3^ Research Unit Ecology and Dynamics of Anthropized Systems University of Picardie Jules Verne Amiens Cedex France; ^4^ Forest & Nature Lab, Department of Environment Ghent University Gontrode Belgium; ^5^ Vegetation Ecology and Conservation Biology, Institute of Ecology, FB 2 University of Bremen Bremen Germany; ^6^ Institute of Ecology and Earth Science University of Tartu Tartu Estonia

**Keywords:** agricultural landscape, genetic connectivity, genetic differentiation, genetic diversity, habitat fragmentation, time lag

## Abstract

Due to multiple land‐cover changes, forest herb populations residing in forest patches embedded in agricultural landscapes display different ages and, thus, experience differences in genetic exchange, mutation accumulation and genetic drift. The extent of divergence in present‐day population genetic structure among these populations of different ages remains unclear, considering their diverse breeding systems and associated pollinators. Answering this question is essential to understand these species' persistence, maintenance of evolutionary potential and adaptability to changing environments. We applied a multi‐landscape setup to compare the genetic structure of forest herb populations across forest patches of different ages (18–338 years). We studied the impact on three common slow‐colonizer herb species with distinct breeding systems and associated pollinators: *Polygonatum multiflorum* (outcrossing, long‐distance pollinators), *Anemone nemorosa* (outcrossing, short‐distance pollinators) and *Oxalis acetosella* (mixed breeding). We aimed to assess if in general older populations displayed higher genetic diversity and lower differentiation than younger ones. We also anticipated that *P. multiflorum* would show the smallest while *O. acetosella* the largest difference, between old and young populations. We found that older populations had a higher observed heterozygosity (*H*_o_) but a similar level of allelic richness (*A*_r_) and expected heterozygosity (*H*_e_) as younger populations, except for *A. nemorosa*, which exhibited higher *A*_r_ and *H*_e_ in younger populations. As populations aged, their pairwise genetic differentiation measured by *D*_PS_ decreased independent of species identity while the other two genetic differentiation measures showed either comparable levels between old and young populations (*G"*_ST_) or inconsistency among three species (*cGD*). The age difference of the two populations did not explain their genetic differentiation. Synthesis: We found restricted evidence that forest herb populations with different ages differ in their genetic structure, indicating that populations of different ages can reach a similar genetic structure within decades and thus persist in the long term after habitat disturbance. Despite their distinct breeding systems and associated pollinators, the three studied species exhibited partly similar genetic patterns, suggesting that their common characteristics, such as being slow colonizers or their ability to propagate vegetatively, are important in determining their long‐term response to land‐cover change.

## Graphical abstract

This study applied a multi‐species multi‐landscape setup to compare the genetic diversity and differentiation among forest herb populations of different ages in agricultural landscapes. We found that the slow‐colonizer species populations of different ages can reach a similar genetic structure within decades and thus persist in the long term after habitat disturbance.

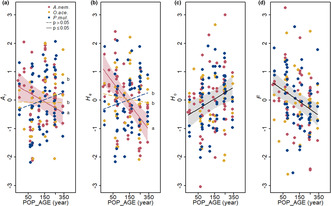

## 1 INTRODUCTION

Many ecosystems exhibit continuous changes in habitat structure. The spatial and temporal habitat heterogeneity, resulting from habitat destruction and creation, has significant demographic and genetic consequences for the organisms inhabiting them (Honnay et al., 2005; Young et al., 1996). Unlike species in habitats experiencing natural disturbance, species living in habitats disturbed by human activities may not be adequately adapted to these disturbances, thereby facing a greater challenge to their long‐term existence.

Temperate forest is the natural habitat for many herb species that once covered large parts of Central and Western Europe (Leuschner & Ellenberg, 2017). This forest‐dominated landscape went through multiple cycles of deforestation and reforestation periods, transforming into an intensively used agricultural landscape, consisting of a mosaic of patches mainly used for agricultural purposes, interspersed with patches of remnant (semi‐) natural habitats (Deckers et al., 2005; Hendrickx et al., 2007). Consequently, forest appears as patches with various ages (Hermy & Verheyen, 2007), and the forest herb populations dwelling in these patches also exhibit different ages.

Populations of different ages may exhibit differences in their genetic structure, that is, genetic diversity and genetic differentiation. Both of them are shaped by evolutionary forces and processes over time, that is, genetic drift, mutation and gene flow (Heywood, 1991; Slatkin, 1985; Waits & Storfer, 2016). The effects of these forces and processes accumulate over the time. Meanwhile, the magnitude of some of these forces can also be influenced by a changing surrounding landscape (Keyghobadi et al., 1999; Merriam et al., 1989). For instance, emergence and disappearance of barriers or corridors for seed or pollen dispersal over time can alter the level of gene flow (García‐Fernández et al., 2019; van Geert et al., 2010), of which the effects accumulate and are reflected in the present‐day population genetic structure (Plue et al., 2017; Reinula et al., 2021). This is especially true for long‐lived species with a long generation time (Aavik et al., 2019), for example, many forest herbs. Examining the genetic structure of populations with different ages is crucial to understand these species' ability to persist and adapt to changing environmental condition and maintain their evolutionary potential (Trapnell & Hamrick, 2023).

Theoretically, old populations in remnants of once large, contiguous forests experienced steady gene flow and/or benefited from a frequent introduction of new genotypes before fragmentation (Pagel et al., 2020). They also had more time for genetic mutations to accumulate (Willi et al., 2018). Moreover, certain reproduction traits that occur in many forest herbs, such as a long generation time and limited sexual reproduction (Whigham, 2004), may have mitigated the effect of genetic drift (Duminil et al., 2009), and thus preserved the genetic diversity after habitat fragmentation. As a result, these ancient populations often exhibit a relatively high genetic connectivity and genetic diversity even today (Landguth et al., 2010). Alternatively, they might have lost genetic diversity directly after habitat fragmentation due to a bottleneck effect caused by a sharp reduction of population size (Aguilar et al., 2008) and subsequent random drift and increased inbreeding (McCauley, 1991).

On the contrary, younger populations that colonized post‐agricultural forest patches had less time to accumulate genetic diversity and genetic exchange. They may show a lower genetic diversity due to the founder effect, which occurs if only a few individuals that do not represent the whole genetic variation of the parent populations and/or that are genetically very different from each other managed to colonize the new habitat (Mayr, 1942; Slatkin, 1977; Whitlock & McCauley, 1990). These small newly founded populations could experience further loss of genetic variation through random drift after the early founding stage, even if the founding individuals initially had a high genetic diversity (Jacquemyn et al., 2009; Nei et al., 1975). Furthermore, lack of specific adaptations that facilitate continuous long‐distance dispersal in species like forest herbs (Whigham, 2004) could limit subsequent migration, which otherwise might compensate the genetic diversity loss due to drift and improve genetic connectivity (Keller & Largiader, 2003; Mona et al., 2014). Nevertheless, given sufficient time and gene flow events, newly founded populations are expected to become less genetically differentiated and accumulate genetic diversity as they get older (Austerlitz & Garnier‐Géré, 2003; Lehmair et al., 2020; Rajora & Zinck, 2021). This process can be fast if the species is exposed to a high seed or pollen exchange (Helsen et al., 2013, 2019; Wang et al., 2011).

So far, few empirical case studies addressed the questions, whether and how these populations with different ages differ in their current genetic structure (Jacquemyn et al., 2004, 2006; Lehmair et al., 2020; Vellend, 2004). All of them focus on a single species in a single landscape. To generalize their findings is difficult for at least three reasons. First, the effect of age is often entangled with other factors such as population size, and thus inconsistent (Vandepitte et al., 2007; Vellend, 2004). It was found that small populations have a lower genetic diversity and higher genetic differentiation compared to large populations (Godt et al., 1996). This might have been mistaken as the effect of population age since young populations are usually also smaller in size (Jacquemyn & Brys, 2008; Reisch et al., 2007). Second, genetic differentiation between populations of different ages is often neglected, as genetic differentiation is usually quantified as a site‐specific measure, averaged across all population pairs (Jacquemyn et al., 2004). In such a measure, the temporal aspect, that is, the age difference of the two populations involved is lost. Last but not least, the outcome of a case study seems to depend on the colonization capacity and the mating strategies of the focal species (Brunet et al., 2012; Verheyen et al., 2003). For example, the genetic structure of a plant species with high extinction‐colonization dynamics and a high colonization capacity may not differ between older and younger populations (Honnay et al., 2009). Older populations of mainly selfing species were found to preserve large genetic variation among populations while those of outcrossing species rather within populations (Landergott et al., 2001). Within outcrossing plant species, different pollination mechanisms may also play an important role in determining the population genetic structure. Young populations of plant species mainly pollinated by far‐reaching vectors, for example, wind, can better overcome the founder effect (Hampe et al., 2013). Similarly, for many insect‐pollinated forest herb species in temperate Europe, their pollinators' foraging range and commuting behaviour should affect the distance and frequency of pollen transport among plant populations (Berge et al., 1998; Feigs et al., 2022; Naaf et al., 2021) and thus the strength of the age effect on the population genetic structure.

In order to disentangle potential interactions of these multiple factors, a population genetic study on multiple forest herb species with different ways of sexual reproduction is needed. In this study, we used six agricultural landscapes across temperate Europe as replicates to compare the effect of population age on the population genetic structure among three slow‐colonizing forest herb species, that is, *Anemone nemorosa* L., which is predominantly outcrossing and pollinated by pollinators with a small foraging rage; *Oxalis acetosella* L., which is self‐compatible and *Polygonatum multiflorum* (L.) ALL., which is obligatory outcrossing and associated with long‐distance foraging pollinators. We anticipated that population age would have varying degrees of impact due to differences in the species' breeding system.

We addressed the following hypotheses in general:

1. Younger populations exhibit a lower genetic diversity than older populations. This effect is beyond the indirect effect mediated by population size, but not independent of the degree of spatial isolation.
2. Younger populations are in general more genetically differentiated than older populations. Moreover, the degree of genetic differentiation rises as the relative age differences increase.
3. Older populations are less, whereas younger populations are more genetically differentiated to each other than that would be expected proportionally based on their geographical distance.
4. The difference of genetic diversity and differentiation between older and younger populations is most pronounced in *O. acetosella* and least pronounced in *P. multiflorum*.

## 2 MATERIALS AND METHODS

### 2.1 Study sites and study species

**FIGURE 1:**
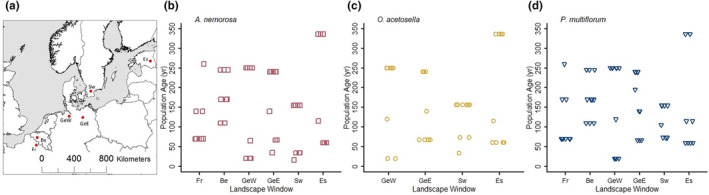
(a) Location of the six landscape windows in Europe. (b–d) Age distribution of all sampled populations of the three species across the six landscape windows (Fr: France; Be: Belgium; GeW: Western Germany; GeE: Eastern Germany; Sw: Sweden; Es: Estonia). Horizontal jitter function was applied in order to avoid overlapping.

We conducted our research in six landscape windows (5 × 5 km^2^) distributed across five countries in temperate Europe: France (Fr), Belgium (Be), Western Germany (GeW), Eastern Germany (GeE), Sweden (Sw) and Estonia (Es) (Figure 1a). Floristic inventories of all forest patches in these landscape windows have been done for earlier projects (Vanneste et al., 2019), which eased the process of population selection (see section 2.2).

**FIGURE 2:**
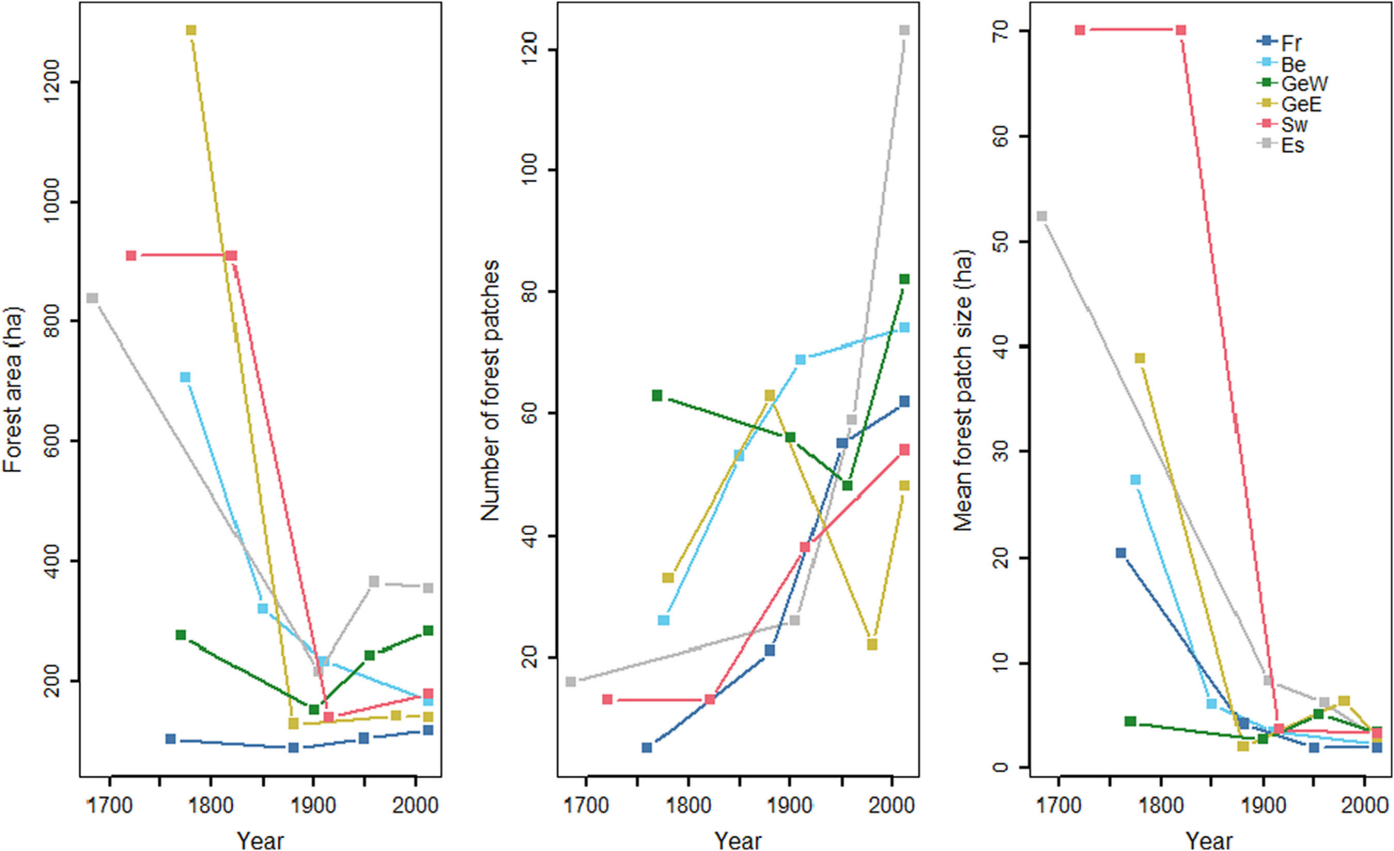
Forest cover changes from the 18th to the 21st century in the six landscape windows (Fr: France, Be: Belgium, GeW: Western Germany, GeE: Eastern Germany, Sw: Sweden, Es. Estonia), where our study took place. Data are based on historical maps (see Table S2).

All selected landscape windows represent common agricultural landscapes found in Europe and were, therefore, used as replicates (Table S1). These landscapes comprise large areas of cultivated fields, grasslands, interspersed with small settlement areas, scattered forest fragments and linear structures such as hedgerows, tree lines, water drainage ditches and roads. The current landscape is the result of several deforestation and afforestation events with a maximum degree of fragmentation at around 1900, at which time both the total forest area and mean forest patch size reached the lowest point (Figure 2). Consequently, the forest patches imbedded in these landscapes have emerged at different times, resulting in different ages (Figure 1b–d, Figure S4). The common history of landscape disturbing and the consequent forest age diversity in these landscape windows provide a suitable system for our study.

The three studied species (*A. nemorosa*, *O. acetosella*, *P. multiflorum*) are common perennial temperate forest herbs that share a similar life history of being slow‐colonizing forest specialists (Schmidt et al., 2014; Verheyen et al., 2003). They concurrently flower in spring (Klotz et al., 2002) and can propagate vegetatively besides regular seedling recruitment (Berg, 2002; Holderegger et al., 1998; Kosiński, 2012). However, they differ in their mating strategies (Table 1). *Anemone nemorosa* and *Polygonatum multiflorum* depend on pollinators for sexual reproduction (Kosiński, 2012; Müller et al., 2000). *Anemone nemorosa* is visited by different groups of insects (Erbar & Leins, 2013; Shirreffs, 1985), with solitary bees and hoverflies being the most important ones (Naaf et al., 2021). These insect groups typically have limited foraging distances and are unlikely to cross the agricultural matrix between forest patches frequently (Feigs et al., 2022). On the contrary, *P. multiflorum* is mainly pollinated by long‐tongued bumblebees (Feigs et al., 2022; Kosiński, 2012), which can cover up to 1000 m and traverse the agricultural matrix between forest patches regularly (Westphal et al., 2006). The third species, *Oxalis acetosella* L. is considered to produce most of its seeds from cleistogamous flowers (Berg & Redbo‐Torstensson, 2000; Packham, 1978). However, our previous research indicated that *O. acetosella* is mostly out‐crossing (Naaf et al., 2021) with potential flower visitors including flies, thrips, beetles, bees and bumblebees (Packham, 1978; Willemstein, 1987).

**TABLE 1:** Differences in life‐history traits for three forest herb species that might affect their response to habitat fragmentation.

	*A. nemorosa*	*O. acetosella*	*P. multiflorum*
Breeding system	Mostly outcrossing^7^	Mixed^2^	Outcrossing^7^
Associated pollinators	Bees, hoverflies and others^10,11,12^ (mostly short distance flyer)	Flies, beetles, thrips, bees^4,9^	Bumblebees^5,12^ (long distance flyer)
Ploidy	Tetraploid^1^	Diploid^8^	Diploid^7^
Age of first flowering	10 years^10^	≥1 year^3^	10 years^6^

*Note*: Superscripts indicate sources of information, which are provided in Supplementary Information S3.

Besides, the three species differ also in some other traits such as ploidy and age of first flowering, which might also affect their response to habitat fragmentation (Table 1).

### 2.2 Population attributes

Since it is seldom possible to determine the age of natural populations (Giles & Goudet, 1997), we used the habitat age to estimate the approximate age of the populations, as done previously by others (Holzhauer et al., 2009; Jacquemyn et al., 2004; Vandepitte et al., 2007). The habitat age may not precisely reflect population age, it is however an adequate proxy under the given conditions, that is, (1) the forest habitat is generally suitable for all three studied species, which occupy a rather broad niche in terms of soil conditions and are very common in temperate forests (Leuschner & Ellenberg, 2017); (2) any extinction followed by re‐colonization in old forest patches is very unlikely given the species' ability to persist even under unfavourable conditions, such as periods of dark shade or drought, for many decades via clonal growth (Eriksson, 1996); (3) any survival of individuals during periods of agricultural land use can be excluded given that the species do not build a persistent seed bank (Kleyer et al., 2008).

To identify the forest patch age, we used historical maps (Table S2). The oldest patches are remnants of forest that has been existing since at least the 18th century while the youngest patches emerged after the 1980s (Figure 1b–d). After excluding patches with no populations of these three species, we decided to sample about 10 populations of each species within each landscape window to cover a sufficient large age spectrum. We defined a population as a spatially distinct group of shoots >100 m apart from other shoots. Typically, these populations covered the whole forest patch. However, they were in some cases restricted to certain parts of a forest patch if habitat conditions were heterogeneous. We selected the populations within the landscape window according to the following criteria: (a) the populations had to represent the full range of available populations ages;(b) they should be maximally distributed across the entire landscape window; (c) the populations of all three species should preferably be in the same forest patches; and (d) more than one population within the same forest patch was only accepted if these populations were separated by ≥200 m and if no other populations were available in other forest patches. Since each forest patch may comprise several sub‐patches that emerged from different times, we chose the age of the oldest sub‐patch covered by the population area as a proxy of the population age, with the assumption that any younger part of the population was a later extension of the oldest part. In total, we included 60 *A. nemorosa* and 60 *P. multiflorum* populations. We could only include 37 populations of *O. acetosella*, since the selection criteria could not be fulfilled in the landscape windows Be and Fr (Supplementary Information S5).

Besides population age, we included two covariables in our analysis, that is, population size and spatial connectivity. These are important determinants of the genetic diversity within populations (Naaf et al., 2021), and may potentially interact with population age.

For each sampled population, we estimated census population size of *A. nemorosa* and *O. acetosella* by extrapolating flower density from a known area to the complete population area. The complete population area was either the corresponding forest patch area, or demarcated in the field by marking the outmost flowering shoots of a population with a GPS device. For calculating flower density, we measured the length of a 2‐m‐wide transect after counting 40 flowering shoots. The flower density of the population was then averaged across five randomly placed transects within the population. For *P. multiflorum*, we calculated the census population size by counting all flowering shoots in the population area since *P. multiflorum* individuals tend to grow in small patches rather than in a carpet‐like fashion across the population area.

The other covariable, spatial connectivity, measures the degree of spatial isolation of a certain population considering all populations within the 5 × 5 km^2^ landscape window. We calculated it with Hanski's (1994) incidence function model $C_{i}=\sum_{j\neq i}A_{j}^{b}\cdot e^{-{\mathrm{ad}}_{\mathrm{ij}}}$, where *C*_*i*_ is the spatial connectivity of population *i*, *A*_*j*_ is the size of population *j*, *d*_*ij*_ is the edge‐to‐edge distance between populations *i* and *j*, and *α* and *b* are calibration parameters. *α* scales the effect of distance to dispersal while *b* regulates the effect of population size on dispersal. We estimated 1/*α* for each species, using the mean nearest‐neighbour distance among all populations in this landscape window, averaged across six landscape windows (274 m, 316 m and 279 m for *A. nemorosa*, *O. acetosella* and *P. multiflorum* respectively). The parameter *b* was set to 0.5, as suggested by Moilanen and Nieminen (2002).

Similarly, we included geographical distance between populations as a covariable in determining the effect of population age on genetic differentiation, since geographical distance often influences genetic differentiation (Slatkin, 1985).

### 2.3 Sampling, DNA extraction and genotyping

Sampling was conducted in the spring of 2018 and 2019. In each population, we collected leaf material from 20 healthy flowering individuals, which were at least 10 meters away from each other to avoid sampling of clones. A total of 2885 leaf samples were included in this study. We extracted total genomic DNA from the leaf samples and genotyped them based on sets of microsatellite markers (Supplementary Information S5) that either had been developed for congeneric species (*A. nemorosa* and *P. multiflorum*) or were newly developed for *O. acetosella* by AllGenetics & Biology SL (Spain) on demand. The applied marker sets comprised six, nine and six markers with a total number of 102, 61 and 149 alleles for *A. nemorosa*, *O. acetosella* and *P. multiflorum* respectively. Samples for which genotyping failed at more than one locus were excluded. Thirty‐five percent of populations had fewer than 20 samples (Supplementary Information S5), either due to a small population size or genotyping failure. We repeated the genotyping procedure for 10% of the samples to estimate the multi‐locus genotyping error rate (3.7%, 2.7% and 4.0% for *A. nemorosa*, *O. acetosella* and *P. multiflorum* respectively). Finally, we excluded all repeated multi‐locus genotypes (MLG) in a population as assumed clones from our analysis. Repeated MLG were randomly distributed across all regions. Also, we excluded populations with less than four MLG as these did not allow a reliable estimation of genetic covariance among populations (see below). The complete allele tables are provided in Supplementary Information S5.

### 2.4 Population genetic measures

For all three species, we calculated four measures of genetic diversity within populations, that is, allelic richness (*A*_r_), observed heterozygosity (*H*_o_), unbiased expected heterozygosity (*H*_e_) and the inbreeding coefficient *F* = 1‐ *H*_o_ / *H*_e_. Since allelic richness is only comparable among similar sample sizes, we calculated rarefied allelic richness based on the mean sample size across six landscape windows, that is, 18, 16 and 16 samples for *A. nemorosa*, *O. acetosella* and *P. multiflorum* respectively. We used the mean instead of the minimum sample size as a trade‐off to avoid losing too much information, given the fact that some populations were very small (Supplementary Information S5). We sampled every findable genet so that these populations were 100% represented even with the small sample size, thus the allelic richness is not biased through extrapolation.

Furthermore, we used three measures to quantify pairwise genetic differentiation among populations, that is, *G"*_ST_, *D*_PS_ and *cGD*. *G"*_ST_ is based on heterozygosity, like traditional *F*_ST_ and *G*_ST_. It is recommended to be used with microsatellite markers and for small sample sizes (Meirmans & Hedrick, 2011). *D*_PS_ is based on the proportion of shared alleles (Bowcock et al., 1994) and thus features an intuitive interpretation. The third measure, *cGD* (conditional genetic distance) (Dyer et al., 2010), is based on the population graph approach (Dyer & Nason, 2004). This approach has been rarely used in landscape ecology so far, although it has been suggested to be complementary to traditional measures (Jones & Manseau, 2022). Traditional summary statistics such as *G"*_ST_ allow us to draw conclusions by inferring the magnitude of the genetic variance that exist among strata. However, the wide range of variance can arise from different and potentially mutually exclusive demographic histories (Dyer & Nason, 2004), which cannot be captured in this way. For example, they cannot distinguish between two populations that are similar to each other due to (a) constant gene flow between them or (b) both populations have constant gene flow with a third population. In contrast, the population graph approach defines genetic variation among strata without estimating the magnitude of the variance and retains only necessary information to describe the total observed variation (Dyer, 2015).

A population graph is a graph‐theoretic interpretation of the population genetic structure in a population network, where nodes represent populations connected by edges. The length of an edge represents the genetic covariance between the two populations. The idea of the population graph is to show an informative topology, which only contains the minimum number of edges to sufficiently describe the total genetic covariance structure. This is done by testing all the edges for their conditional independence. Edges that do not add unique information to describe the genetic covariance are “pruned” from the final population graph (Dyer & Nason, 2004). This process is essential since a saturated network with edges between all pairs of populations does not deliver information about how gene flow occurs through the network (Jones & Manseau, 2022). We estimated *cGD* as the length of the shortest path through the graph connecting a pair of populations which represents pairwise genetic differentiation between these two linked populations (Dyer et al., 2010). For this purpose, we created a population graph for each species at each landscape window (Figure S8). The functions used to produce a population graph provided by the packages ‘igraph’ (Csardi & Nepusz, 2006) and ‘popgraph’ (Dyer & Nason, 2004) require genetic data without missing values. In order to make *cGD* comparable to *G"*_ST_ and *D*_PS_, we adjusted these functions so that genetic data with missing values can be handled (Supplementary Information S11).

Additional to *cGD*, we calculated two further graph‐topological metrics to characterize the population genetic structure. These topological metrics can provide a biologically meaningful inference of a graphically depicted population assemblage that is similar to a meta‐population (Bode et al., 2008; Dallas et al., 2020), since certain topological metrics are correlated with population genetic parameters (Dyer, 2007). For nodes, we calculated normalized harmonic centrality (*NHc*) (Dekker, 2005; Marchiori & Latora, 2000), which could be interpreted as overall genetic connectivity of one population, when the internodal distance is measured by genetic distance (Murphy et al., 2016). In this sense, it is complementary to the pairwise genetic differentiation measures above, which indicate pairwise genetic connectivity. Harmonic centrality measures how close a node is to all the other nodes in a graph by summing up the inverse of the genetic distance of this node to all other nodes. Here, the genetic distances are standardized by their minimum value in each population graph and thus range in [1, +∞]. By normalizing harmonic centrality through its division by the number of populations in the graph, harmonic centrality can be compared among networks of different size. A high harmonic centrality means that a node is well connected, while a harmonic centrality approaching zero means that the node is highly isolated. A population with a high centrality has potentially a larger relative influence than those with a lower centrality (Murphy et al., 2016).

For edges, we calculated the difference between the proportional genetic distance ($P{\mathrm{cGD}}_{i}={\mathrm{cGD}}_{i}/\sum {\mathrm{cGD}}_{i}$) of a certain population pair in a population graph and the corresponding proportional geographical distance of this population pair (DIFF_GEN_GEO). A positive or negative DIFF_GEN_GEO value means that the genetic distance between two populations is larger or shorter than would be expected proportionally from their geographical distance (Dyer, 2015).

## 3 DATA ANALYSIS

### 3.1 Age effects on genetic diversity

To test our first hypothesis that population age affects population genetic diversity, we modelled genetic diversity as a function of population age using linear mixed models (LMMs). Landscape window and forest patch nested in each landscape window were used as random factors in a random‐intercept model. Besides the fixed effects population age (POP_AGE) and species (SPECIES), we included the two covariables, that is, population size (POP_SIZE) and spatial connectivity (SPA_CON), as predictors.

Due to the limited sample size, we first included only the interactions between SPECIES and the other three predictors in the global model: Y ~ (POP_SIZE + SPA_CON + POP_AGE) * SPECIES, in which Y represents different diversity measures. The purpose of this model was to test (1) whether POP_AGE has an effect on genetic diversity; (2) whether different species respond differently to POP_AGE or the covariables. We simplified the global models by fitting models with all subsets of predictors and selected the model with the lowest AIC_c_ as our final model. SPECIES was treated as a fixed term that could not be removed.

Then we used a second global model for each species separately to test whether the effect of population age (POP_AGE) on genetic diversity varies with the two covariables: Y ~ (POP_SIZE + SPA_CON) * POP_AGE. In this model, only landscape window was used as random effect.

Despite a certain level of collinearity between population size and population age as well as between spatial connectivity and population age (see Result section), we decided to keep them in the model since the generalized variation‐inflation factor (VIF) was low for all tested models (VIF < 4.0). Before modelling, quantitative variables were Box‐Cox transformed in order to enhance the symmetry of their distribution, and all variables were standardized to mean = 0 and SD = 1 in order to get standardized regression coefficients. Standardization of POP_SIZE and SPA_CON was conducted separately for each species to yield comparable effect sizes and to eliminate absolute differences in genetic diversity among the three species, while standardization of POP_AGE was conducted for all three species together since it was derived from the same set of maps for each species at each landscape window.

### 3.2 Age effect on genetic connectivity

To test our second hypothesis that population age affects pairwise genetic connectivity, we created two pairwise age‐related predictors: (1) AGE_BASE, which is the age of the younger population in a population pair. The rationale behind is that genetic exchange between two populations can only start after the younger population has established. (2) AGE_DIFF represents the age difference between the two populations in a population pair. When combined with AGE_BASE, AGE_DIFF provides a measure of the age of the older counterpart within the pair. The concept is rooted in the understanding that populations with different ages or demographic stages exhibit differences in flower or seed production (Ally et al., 2010; Pan & Price, 2002; Roach et al., 2009), and thus contribute differently in shaping genetic structure. Older populations tend to have a lower fertility and allocate fewer resources to sexual reproduction while prioritizing clonal growth (Piquot et al., 1998). Consequently, at a given AGE_BASE, a larger AGE_DIFF indicates a reduced effective gene flow through seeds or pollen, which may lead to greater pairwise genetic differentiation.

For the measure *cGD*, we included only those population pairs directly connected by an edge in the population graph into our analysis, since AGE_DIFF did not consider age information of in‐between populations in the graph.

Similar as above, we used a two‐step approach. First, we regressed genetic differentiation (*G"*_ST_, *D*_PS_ and *cGD*) against the two age‐related variables (AGE_DIFF and AGE_BASE) as well as geographic distance as a covariable (GEO_DIST) and their interaction with SPECIES by using maximum‐likelihood population effects (MLPE) models (Clarke et al., 2002): Y ~ (AGE_DIFF * AGE_BASE + GEO_DIST) * SPECIES. This model type takes the lack of independency among pairwise comparisons into account. We implemented MLPE models by defining a correlation structure within the lme function (R Package ‘nlme’), using the function corMLPE (Pope, 2022). Landscape window was used as a random intercept term. Models were simplified by fitting models with all subsets of predictors and selecting the model with the lowest AIC_c_ as our final model. SPECIES was treated as a fixed term that could not be removed.

Secondly, we applied another MLPE model for each species separately, to test whether any effects of AGE_BASE and AGE_DIFF on genetic differentiation would depend on GEO_DIST: Y ~ (AGE_BASE + AGE_DIFF) * GEO_DIST.

Similar as above, all quantitative variables were Box‐Cox transformed and then standardized to mean = 0 and SD = 1. Standardization of genetic differentiation measures was conducted for each species separately, while GEO_DIST and age‐related variables were standardized for all three species together.

### 3.3 Population graph analysis

We tested the second hypothesis also by regressing normalized harmonic centrality (*NHc*) against POP_AGE and SPECIES, using a LMM with landscape window as random factor. Then, we tested the third hypothesis, that is, how populations are connected to each other across a given geographical distance. Thus, we tested whether the age attributes of the population pair (AGE_DIFF and AGE_BASE) influenced the correspondence between genetic and geographical distance (DIFF_GEN_GEO) by applying a MLPE model: DIFF_GEN_GEO ~ AGE_DIFF * AGE_BASE * SPECIES, with landscape window as random factor. All continuous variables were Box‐Cox transformed and scaled to mean = 0 and SD = 1. We simplified the models by choosing the model with the lowest AIC_c_ after fitting models with all subsets of predictors. SPECIES was treated as a fixed term that could not be removed.

The entire data analysis was conducted using R version 4.1.3.

## 4 RESULTS

### 4.1 Population attributes and their collinearity

**FIGURE 3:**
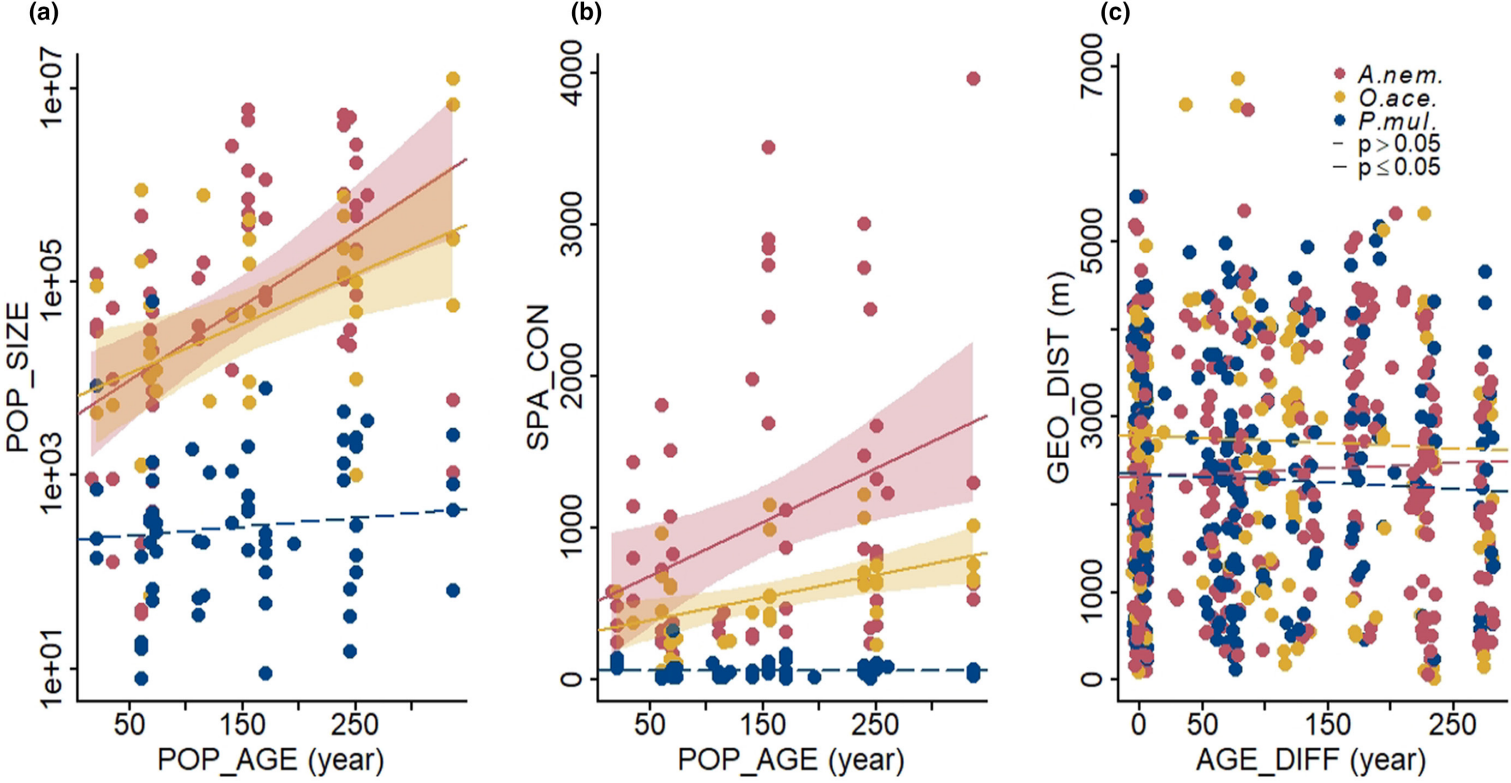
Differences among the three studied species (*A. nem*.: *Anemone nemorosa*; *O. ace*.: *Oxalis acetosella*; *P. mul*.; *Polygonatum multiflorum*) in (a) population size (POP_SIZE), (b) spatial connectivity (SPA_CON) in relation to population age (POP_AGE), and (c) pairwise geographical distance (GEO_DIST) in relation to population age difference (AGE_DIFF). Regression lines from simple linear regression models and 95% confidence bands (only of significant results) are shown.

For *A. nemorosa* and *O. acetosella*, population size and spatial connectivity increased with population age, whereas for *P. multiflorum*, population size and spatial connectivity were comparable across different age levels (Figure 3a + b). Population size and spatial connectivity were correlated in all three species (*A. nemorosa*: *r* = .76, *p* < .001; *O. acetosella*: *r* = .63, *p* < .001; *P. multiflorum*: *r* = .68, *p* < .001).

There were clear absolute differences in population attributes among the three species. *Polygonatum multiflorum* populations were smaller and had lower spatial connectivity values than populations of *A. nemorosa* and *O. acetosella* (Figure 3a + b), which reflects differences in species‐specific local shoot abundance among species. *Oxalis acetosella* populations were genetically less diverse than those of *A. nemorosa* and *P. multiflorum* (*A*_r_: *A. nem*.: 7.34 ± 1.34, *O. ace*.: 2.37 ± 0.36; *P. mul*.: 7.06 ± 1.72; Table S6), which is due to the difference in species‐specific microsatellite marker sets. Pairwise geographical distance did not covary with the age difference between populations in any of the three species, nor did it differ among species (Figure 2c).

### 4.2 Effects of population age on genetic diversity

**FIGURE 4:**
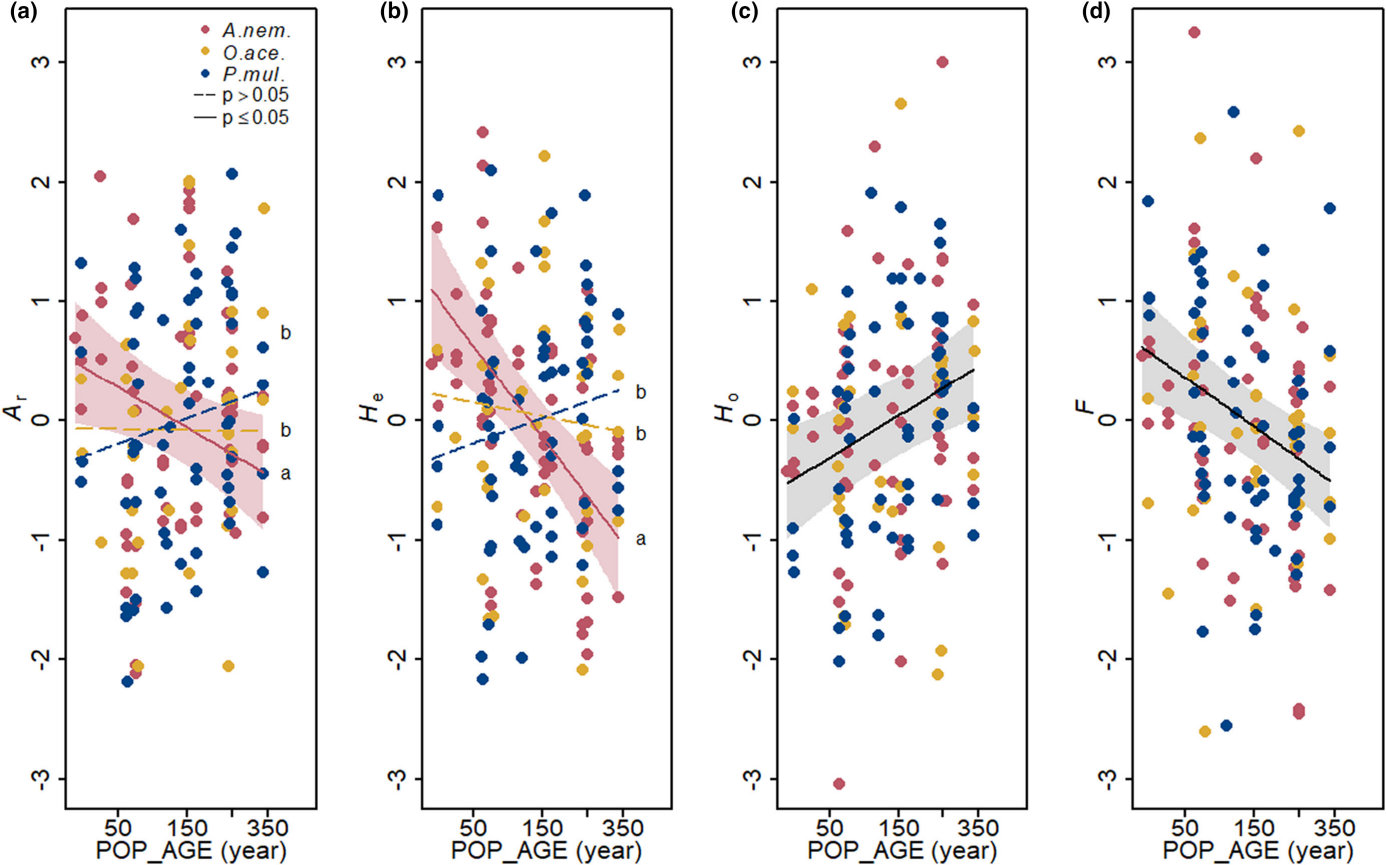
Partial effects of population age (POP_AGE) on measures of genetic diversity: (a) *A*_r_: allelic richness, (b) *H*_e_: expected heterozygosity, (c) *H*_o_: observed heterozygosity, (d) *F*: inbreeding coefficient as resulting from linear mixed models. All variables are scaled in standard deviation units. Regression lines and 95% confidence bands (only for significant results) in the respective colour of each species. Black lines represent effects that are independent of species identity.

When models were fitted for all three species combined, population age had a limited effect on genetic diversity regardless of species identity. Generally, observed heterozygosity (*H*_o_) increased while the inbreeding coefficient (*F*‐value) decreased with increasing population age (Table 2, Figure 4c + d). For *A. nemorosa*, both allelic richness (*A*_r_) and expected heterozygosity (*H*_e_) decreased with increasing population age (Table 2, Figure 4a + b).

**TABLE 2:** Genetic diversity (*A*_r_: allelic richness; *H*_e_: expected heterozygosity; *H*_o_: observed heterozygosity; *F*: inbreeding coefficient) as a function of population age (POP_AGE), population size (POP_SIZE), spatial connectivity (SPA_CON) and species identity according to the results of linear mixed modelling. Given are standardized regression coefficients.

	*A*_r_	*H*_e_	*H*_o_	*F*
POP_AGE				
*A. nem*.	−0.25*^a^	−0.57**^a^	0.26**	−0.31**
*O. ace*.	−0.01^n.s.ab^	−0.09^n.s.b^		
*P. mul*.	0.16^n.s.b^	0.16^n.s.b^		
POP_SIZE				
*A. nem*.				
*O. ace*.	0.26**			
*P. mul*.				
SPA_CON				
*A. nem*.	0.36**	0.24**	−0.33*^a^	0.16(*)
*O. ace*.			0.07^n.s.b^	
*P. mul*.			0.18^n.s.b^	
Marginal *R* ^*2*^	.31	.15	.10	.09
Conditional *R* ^*2*^	.40	.19	.23	.18

*Note*: Included are only variables left in the final model after model selection. Coefficients are stated in the middle across the three species if there was no significant interaction between species and the corresponding independent variable. Significance of regression coefficients is indicated by asterisks: ^n.s.^ *p* > .1; (*)*p* ≤ .1; *p ≤ .05; **p ≤ .01. Significant differences (*α* = .05) in slopes among species are indicated by lowercase letters.

**FIGURE 5:**
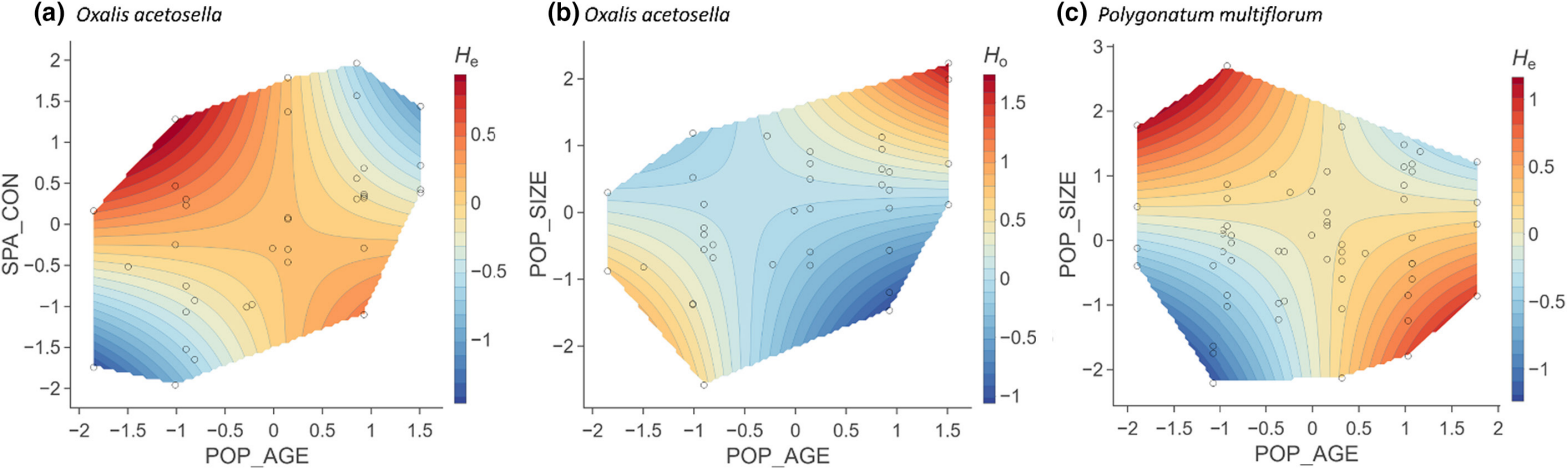
Visualization of statistically significant interactive partial effects between (a) spatial connectivity (SPA_CON) and population age (POP_AGE) on expected heterozygosity (*H*_e_) of *O. acetosella*; (b) population size (POP_SIZE) and population age (POP_AGE) on observed heterozygosity (*H*_o_) of *O. acetosella*; (c) population size (POP_SIZE) and population age (POP_AGE) on expected heterozygosity (*H*_e_) of *P. multiflorum*. All variables are scaled in standard deviation units.

When models were fitted separately for each species, population age interacted with population size or spatial connectivity in affecting two of the genetic diversity measures (*H*_e_ and *H*_o_) (Tables S7.1–S7.3). We detected a negative effect of population age on expected heterozygosity (*H*_e_) in spatially well‐connected *O. acetosella* and larger *P. multiflorum* populations (Figure 5a + c, Tables S7.2 and S7.3). Besides, population age had a positive effect on observed heterozygosity (*H*_o_) in larger *O. acetosella* populations (Figure 5b, Table S7.2).

### 4.3 Effects of population age on genetic differentiation

**FIGURE 6:**
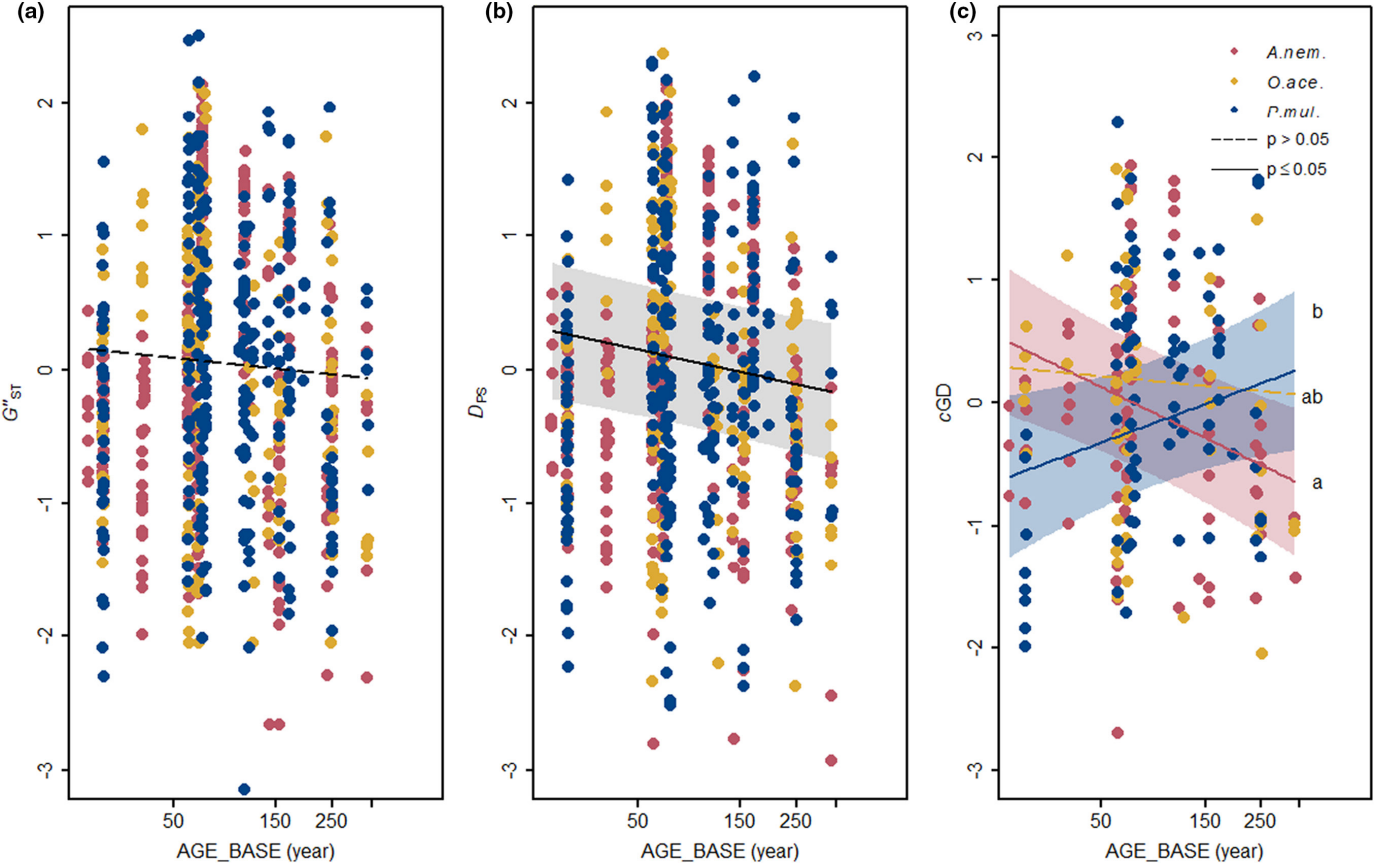
Pairwise genetic differentiation measured by (a) *G"*_ST_, (b) *D*_PS_ and (c) *cGD* in dependence of the age of the younger population (AGE_BASE) in the population pair. Shown is the partial effect of the model, in which the covariable geographical distance (GEO_DIST) and AGE_DIFF were held constant at their mean value. All variables are scaled in standard deviation units. Regression lines and 95% confidence bands (only for significant effects) are shown in the respective colour of each species. Black lines represent effects that are independent of species identity.

Pairs of older populations exhibited a lower genetic differentiation than pairs involving at least one younger population. This effect occurred in all three species when genetic differentiation was measured by *D*_PS_ (Figure 6b, Table 3), while it was not statistically significant or occurred only in *A. nemorosa* when genetic differentiation was measured by *G"*_ST_ or *cGD* respectively (Figure 6a + c, Table 3). In contrast, older *P. multiflorum* pairs exhibited a higher genetic differentiation than younger population pairs when measured by *cGD*. The age difference (AGE_DIFF) between the populations in a pair did not have a significant effect on genetic differentiation. Moreover, genetic differentiation generally increased with geographic distance between populations regardless of species identity (Table 3).

**TABLE 3:** Genetic differentiation (*G"*_ST_, *D*_PS_ and *cGD*) as a function of population age (AGE_BASE, AGE_DIFF), species identity and geographical distance (GEO_DIST).

	*G"*_ST_	*D*_PS_	*cGD*
AGE_BASE			
*A. nem*.			−0.28**^a^
*O. ace*.	−0.05^n.s.^	−0.11**	−0.05^n.s.ab^
*P. mul*.			0.21*^b^
GEO_DIST			
*A. nem*.	0.01^n.s.a^	0.09**	0.07*
*O. ace*.	0.03^n.s.ab^		
*P. mul*.	0.13**^b^		
Marginal *R* ^*2*^	.02	.02	.07
Conditional *R* ^*2*^	.17	.29	.35

*Note*: Given are standardized regression coefficients of variables that are included in the final model after model selection. Coefficients are stated in the middle across the three species if there was no significant interaction between species and the corresponding independent variable. Significance of regression coefficients is indicated by asterisks: ^n.s.^ *p* > .1; (*)*p* ≤ .1; *p ≤ .05; **p ≤ .01. Significant differences (*α* = .05) among species are indicated by lowercase letters.

**FIGURE 7:**
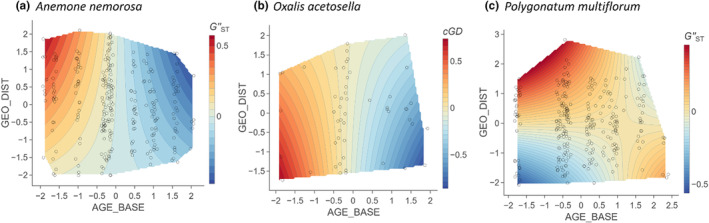
Visualization of statistically significant interactive partial effects between geographical distance (GEO_DIST) and the age of the younger population in a population pair (AGE_BASE) on pairwise genetic differentiation measures (*G"*_ST_, *cGD*) in (a) *Anemone nemorosa*, (b) *Oxalis acetosella*, (c) *Polygonatum multiflorum*.

In models fitted separately for each species, we detected interactions between the age of the population pair and geographical distance in all three species (Tables S9.1–S9.3). Both in *A. nemorosa* and *P. multiflorum*, the negative effect of the population pair age on genetic differentiation (*G"*_ST_) was stronger in populations further away from each other (Figure 7a + c), while in *O. acetosella*, it was stronger in populations closer to each other (*cGD*; Figure 7b).

Normalized harmonic centrality (*NHc*) was not higher in older than in younger populations, independent of species identity (*β* = 0.09 ± 0.08, *p* = .219, Figure S10a).

### 4.4 Effects of age on the correspondence between genetic and geographical distance

Among all existing edges, the correspondence between genetic distance and geographical distance (*DIFF_GEN_GEO*) did not vary with AGE_DIFF, nor with AGE_BASE. (AGE_DIFF: *β* = −0.01 ± 0.07, *p* = .958; AGE_BASE: *β* = −0.07 ± 0.08, *p* = .299, Figure S10b+c). This was true independent of species identity.

## 5 DISCUSSION

Our study used a multi‐species, multi‐landscape approach to show that population age influences the genetic diversity and genetic differentiation of spatially isolated forest herb populations. The observable effects of population age were generally less pronounced than expected, with a part of the results aligning with our initial hypotheses. Some patterns occurred independent of species identity and in multiple alternative population genetic measures, while others occurred only in certain species and/or with certain measures.

### 5.1 Restricted evidence for a positive effect of age on genetic diversity

We found some evidence supporting our hypothesis that younger populations are genetically less diverse than older populations, although this was limited to certain genetic diversity measures for all three species (*H*_o_ and *F*, Table 2, Figure 4c + d) and not independent of other covariables, that is, spatially more isolated populations in *O. acetosella* (Figure 5a) and small populations in *P. multiflorum* (Figure 5c). This result indicates that (a) remnant populations from old forest patches may preserve a high level of heterozygosity until today (Otálora et al., 2011) and/or that (b) younger populations are still under founder effect (Helsen et al., 2019).

Nevertheless, our first hypothesis was not supported by the result on genetic diversity measured by *A*_r_ and *H*_e_ (Figure 4a + b), where the patterns were partly opposite to the expectation and/or inconsistent among three species. This will be further discussed in section 5.3.

### 5.2 Population pair age and geographical distance rather than relative age difference explain pairwise genetic differentiation

Our second hypothesis that younger populations exhibit a higher genetic differentiation compared to older populations was partly supported by the result on *D*_PS_. It was, however, contradicted by the other two genetic differentiation measures which showed inconsistent patterns. Since the three measures have a different reaction time and sensitivity to sampling effort, we should interpret the results with caution.

The allele frequency‐based measure *D*_PS_ (as well as *cGD*) responds more rapidly (Dyer et al., 2010; Murphy et al., 2010), and thus may reflect a more recent landscape configuration. In comparison, the *F*_ST_‐related measure *G"*_ST_ is more efficient in quantifying historical gene flow (Balkenhol et al., 2009). In respect of the long generation time of the three species, the time range included in this study (max. 338 years) may still be too short to display the difference, which might explain the weak signal of *G"*_ST_.

The third measure *cGD* showed contrasting responses among the three species (Figure 6c). Only the response of *A. nemorosa* supported our hypothesis. Since *cGD* excludes unnecessary genetic covariance (Dyer, 2015), we may consider that this pattern shows a more realistic picture of gene flow. Nevertheless, we should still keep in mind that *cGD* is sensitive to incomplete sampling of populations because, instead of relying on pairwise relationships, *cGD* is estimated based on the totality of the data (population network) (Beerli, 2004; Koen et al., 2013). The more populations sampled, the higher is the accuracy in assessing gene flow. In our study, the percentage of sampled populations (*A. nem*.: 44%; *O. ace*.: 36%, *P. mul*.: 42%) might be responsible for partly unclear and inconsistent effects of AGE_BASE on *cGD*. This same argument may also explain, why we found no evidence for a higher centrality of older compared to younger populations. Nevertheless, we still think using a network‐based approach is justified, because it is even more difficult to assess the effect of unsampled populations on pairwise metrics like *G"*_ST_ (Koen et al., 2013).

Different from the expectation, AGE_DIFF had no effect on pairwise genetic differentiation (*G"*_ST_, *D*_PS_, *cGD*), nor had the correspondence between genetic and geographic distance (DIFF_GEN_GEO) (Figure S10b+c). This suggests that old and young populations contribute comparably to gene flow. One possible explanation is the strong dispersal limitation of all three species (Schmidt et al., 2014), so that the difference in seed and/or pollen production potential between populations with a different age may not play a significant role in shaping genetic differentiation. As a support, we also found a strong effect of geographic distance on genetic differentiation (Table 3), indicating that gene flow is primarily determined by distance between populations rather than their age.

### 5.3 Common and species‐specific characteristics explain unexpected and contradictory patterns

While we hypothesized the responses to population age to differ among species (H4), we expected mostly differences in effect size rather than effect direction. However, some of the species‐specific patterns observed do not conform to the general trends expected (H1–H3). These unexpected patterns might be explained by species‐specific traits.

Contrary to our hypothesis, we observed a lower allelic richness (*A*_r_) and expected heterozygosity (*H*_e_) in older than in younger populations. This effect was generally found in *A. nemorosa* (Figure 4a + b), and partly in relatively well‐connected *O. acetosella* (Figure 5a) and large *P. multiflorum* populations (Figure 5c). Clonal growth, as one of the common forest herb characteristics, may help to explain these unexpected patterns. Older populations, inhabiting more stable and more competitive environments such as ancient forests (Hermy et al., 1999; Salibury, 1942; Sarukhan & Gadgil, 1974), may display a higher prevalence of vegetative propagation compared to sexual reproduction. This is because the balance between vegetative and sexual reproduction is influenced by abiotic factors including moisture, light and temperature, and biotic factors like competition (Solbrig, 1980). Consequently, in older populations, particular dominant genotypes may outcompete other less dominant genotypes through higher vegetative reproduction (Stehlik & Holderegger, 2000). This strong clonal growth could result in biparental inbreeding or even self‐fertilization, further contributing to a lower genetic diversity in offspring generations (Lloyd & Barrett, 1996).

Furthermore, a long generation time, as exhibited by *A. nemorosa* and *P. multiflorum* (Kosiński, 2008; Shirreffs, 1985), may provide another explanation. Genetic diversity takes generations to reach an equilibrium (Caplins et al., 2014; Epps et al., 2005). Thus, the current genetic diversity may still reflect the historical landscape (Aavik et al., 2019). A reduced spatial connectivity in the past, more precisely the time of minimum forest cover and patch size around 1900 (Figure 2), might explain the unexpected patterns in which older *P. multiflorum* populations have lower expected heterozygosity (Figure 5c).

For *O. acetosella*, the unexpected low *A*_r_ and *H*_e_ in well‐connected old populations (Figure 5a) could be further attributed to its nature of having both chasmogamous and cleistogamous flowers. The relative dominance of chasmogamous and cleistogamous flowers varies with population age, with young ramets producing more chasmogamous flowers (Berg & Redbo‐Torstensson, 1998; Koontz et al., 2017). Also, a high resource availability in postagricultural forests (Brunet et al., 2012) may trigger the production of more chasmogamous flowers, given that they are less cost efficient (Diaz & Macnair, 1998). Consequently, well‐connected young populations of *O. acetosella* with more chasmogamous flowers may experience frequent cross‐population pollination, which increases population genetic diversity. In contrast, older populations with fewer chasmogamous flowers may experience reduced cross‐population pollination.

The unexpected positive effect of age on *cGD* in *P. multiflorum* (Figure 6c), could be explained by its associated pollinators. Different from *A. nemorosa*, which is associated with short‐distance, forest‐specialized pollinators (Feigs et al., 2022), *P. multiflorum* is pollinated by bumblebees (Kosiński, 2012) that are able to fly over a heterogeneous landscape (Persson & Smith, 2011). During the forest fragmentation period, large forest habitats of *P. multiflorum* were turned into a large number of small patches (Figure 2), intersected with many other land‐use types, including bumblebee friendly land‐use types, such as grassland and hedgerows (Byrne & delBarco‐Trillo, 2019; Naaf et al., 2022). This type of heterogeneous habitat may benefit bumblebee species not specific to forest by providing nesting and foraging sites (Gómez‐Martínez et al., 2020), which may in turn facilitate a higher genetic connectivity of younger *P. multiflorum* populations than of older ones.

### 5.4 The importance of the surrounding environment out of the habitat

A large unexplained variation in the global models (Tables 2 and 3) indicates that factors other than geographical distance and the considered population attributes, that is, population size, connectivity and population age, may further explain the population genetic structure. Gene flow among plant populations is largely influenced by the environment outside of their actual habitats, since the surrounding environment contributes to the habitat of pollinators or seed vectors (Breitbach et al., 2012; Jauker et al., 2009). Thus, we might find more species‐specific patterns if we take the landscape structure into account (Naaf et al., 2022). This was, however, beyond the scope of this study.

## 6 CONCLUSION

Our study provides several important insights. Firstly, we found restricted signals of population age in the population genetic structure of all three studied species. This means that gene flow among spatially isolated forest herb populations is taking place and may even out any differences in genetic diversity and differentiation between populations of different age within the course of several decades. It indicates that certain forest herb species, despite being specialists of the forest, still exhibit resilience in terms of long‐term persistence and adaptation within landscapes disturbed by human activities. Secondly, next to species' difference in their breeding systems and associated pollinators, it is important to consider their common characteristic, such as being slow colonizers, as well as further species‐specific traits, in order to comprehend the unexpected patterns in genetic diversity and differentiation. This is crucial for determining the extent to which the capacity for long‐term persistence can be generalized. Last but not least, by considering pairwise rather than only population‐focused analyses and deriving population genetic measures from population graphs, we gained the knowledge that, in addition to geographical distance, the age of population pairs rather than their relative age influences genetic structure. The discrepancies among the different genetic differentiation measures emphasized the importance of not relying on a single measure but of combining the advantages of multiple measures to achieve a more comprehensive understanding.

## AUTHOR CONTRIBUTIONS

**Siyu Huang:** Conceptualization (supporting); formal analysis (lead); investigation (lead); methodology (equal); visualization (lead); writing – original draft (lead); writing – review and editing (lead). **Jannis Till Feigs:** Conceptualization (supporting); formal analysis (supporting); investigation (supporting); methodology (supporting); writing – original draft (supporting); writing – review and editing (supporting). **Stephanie I. J. Holzhauer:** Conceptualization (supporting); formal analysis (supporting); funding acquisition (supporting); investigation (supporting); methodology (supporting); writing – original draft (supporting); writing – review and editing (supporting). **Katja Kramp:** Conceptualization (supporting); formal analysis (supporting); funding acquisition (supporting); investigation (supporting); methodology (supporting); writing – original draft (supporting); writing – review and editing (supporting). **Jörg Brunet:** Investigation (supporting); writing – review and editing (supporting). **Guillaume Decocq:** Investigation (supporting); writing – review and editing (supporting). **Pieter De Frenne:** Investigation (supporting); writing – review and editing (supporting). **Martin Diekmann:** Investigation (supporting); writing – review and editing (supporting). **Jaan Liira:** Investigation (supporting); writing – review and editing (supporting). **Fabien Spicher:** Investigation (supporting); writing – review and editing (supporting). **Pieter Vangansbeke:** Investigation (supporting); writing – review and editing (supporting). **Thomas Vanneste:** Investigation (supporting); writing – review and editing (supporting). **Kris Verheyen:** Investigation (supporting); writing – review and editing (supporting). **Tobias Naaf:** Conceptualization (lead); formal analysis (supporting); funding acquisition (lead); investigation (supporting); methodology (equal); project administration (lead); supervision (lead); writing – original draft (supporting); writing – review and editing (supporting).

## FUNDING INFORMATION

This research was founded by the German Research Foundation (Research Grants NA 1067/2‐1, HO 4742/2‐1, and KR 5060/1‐1). This includes the research work of JTF, KK, SH, SIJH and TN. PDF and PV were supported by the European Research Council (ERC Starting Grant FORMICA No. 757833, 2018). JL was supported by the Estonian Research Competency Council Grant PRG1223 and the European Regional Development Fund (the Centre of Excellence, EcolChange). TV was funded by the Special Research Fund (BOF) from Ghent University (Grant Number 01N02817). This work was supported by the FWO Scientific research network FLEUR (http://www.fleur.ugent.be). This research was also partly funded by the German Federal Ministry of Food and Agriculture (BMEL) and the Ministry for Science, Research and Culture of the State of Brandenburg (MWFK).

## CONFLICT OF INTEREST STATEMENT

The authors declare that the research was conducted in the absence of any commercial or financial relationships that could be construed as a potential conflict of interest.

## Supporting information


Appendix S1.

## Data Availability

The microsatellite allele tables for all species and populations as well as population locations and R‐code for analysis are available as Supporting Information.
