# Supplementary material for: Limited effects of population age on the genetic structure of spatially isolated forest herb populations in temperate Europe
Source: Ecol Evol. 2024 Feb 26;14(2):e10971. doi: 10.1002/ece3.10971 (PMC10897356; doi:10.1002/ece3.10971)
Supplement: Supplementary file 1 — Appendix S1. [file ECE3-14-e10971-s001.zip › 01_Data_Preparation.nb.html]

01\_Data\_Preparation


Code 

- Show All Code
- Hide All Code
- Download Rmd

# 01\_Data\_Preparation


```
library(poppr)
library(pegas)
library(polysat)
library(RUtils)
source("P:/PB2-Projects/Landgen/05_Data analysis/Functions for population genetics.r")

##Anemone nemorosa
Ane<-read.csv2("Ane_AlleleTable_Est_correct.csv",sep=",")
rownames(Ane)<-Ane[,1]
Ane<-Ane[,-1]
## prepare genetic data                      
ane.l<-alleles2loci(Ane,ploidy=4,population=1)
colnames(ane.l) <- sub("_1", "", colnames(ane.l)) 
ane.gi<-loci2genind(ane.l,na.alleles="-999",ploidy=4)## there is one indiv in Be without sucessful genotyping, is excluded in this step
ane.gic<-ane.gi
#levels(ane.gic@pop)
#summary(ane.gi@pop)
## prepera strata
strats <- base::strsplit(as.character(ane.gic@pop), "_")
ane.gic@strata <- data.frame(Population = ane.gic@pop,
                            Species = sapply(strats, function(x) x[[1]]),
                            Region = sapply(strats, function(x) x[[2]]),
                            ForestPatch = sapply(strats, function(x) x[[3]]))
## there are in total 6 loci, we exclude individuals with more than missing value in 1 locus (17%)                            
info_table(missingno(ane.gic, "geno", cutoff=0.17),plot=T) 
## remove individuals with more than 17% missing value
ane.narm<-missingno(ane.gic, "geno", cutoff=0.17)
## summary table
sumtab.ane<-poppr(ane.narm, quiet=T)
sumtab.ane[,1:3]
# remove clones
anecc_all <- clonecorrect(ane.narm, strata=~Population)
save(file="anecc_all.RData", list="anecc_all")
```


```
## Polygonatum multiflorum
Pol<-read.csv2("Pol_AlleleTable.csv",sep=",")
rownames(Pol)<-Pol[,1]
Pol<-Pol[,-1]
pol.l<-alleles2loci(Pol,population=1)
colnames(pol.l) <- sub("_1", "", colnames(pol.l)) 
pol.gi<-loci2genind(pol.l,na.alleles="-999")
summary(pol.gi@pop)
pol.gic<-popsub(pol.gi,exclude=c("Pol_Est_02","Pol_Est_61"))## these two populations are excluded due to low individual number:1 and 3 individuals
genotype_curve(pol.gic)
strats <- base::strsplit(as.character(pol.gic@pop), "_")
pol.gic@strata <- data.frame(Population = pol.gic@pop,
                               Species = sapply(strats, function(x) x[[1]]),
                               Region = sapply(strats, function(x) x[[2]]),
                               ForestPatch = sapply(strats, function(x) x[[3]]))
## same as in Anemone nemorosa, we exclude individuals with missing value in more than 1 locus
info_table(missingno(pol.gic, "geno", cutoff=0.17), plot=TRUE) 
pol.narm<-missingno(pol.gic, "geno", cutoff=0.17)
##remove clones
polcc_all<-clonecorrect(pol.narm,strata=~Population)
summary(polcc_all@pop)
polcc_all<-popsub(polcc_all,exclude=c("Pol_Est_01","Pol_FrN_09"))## populations with less than 4 MLG is removed
poppr(polcc_all)
save(file="polcc_all.RData", list="polcc_all")
```


```
##Oxalis acetosella
Oxa<-read.csv2("Oxa_AlleleTable_FrNmoved.csv",sep=",")
rownames(Oxa)<-Oxa[,1]
Oxa<-Oxa[,-1]
oxa.l<-alleles2loci(Oxa,population=1)
colnames(oxa.l) <- sub("_1", "", colnames(oxa.l)) 
oxa.gic<-oxa.gi<-loci2genind(oxa.l,na.alleles="-999")
strats <- base::strsplit(as.character(oxa.gic@pop), "_")
oxa.gic@strata <- data.frame(Population = oxa.gic@pop,
                            Species = sapply(strats, function(x) x[[1]]),
                            Region = sapply(strats, function(x) x[[2]]),
                            ForestPatch = sapply(strats, function(x) x[[3]]))


info_table(missingno(oxa.gic, "geno", cutoff=0.1),plot=T) 
oxa.narm<-missingno(oxa.gic, "geno", cutoff=0.1)
oxacc_all<-clonecorrect(oxa.narm, strata=~Population)
summary(oxacc_all@pop)
oxacc_all<-popsub(oxacc_all,exclude=c("Oxa_GeE_08","Oxa_Est_03","Oxa_Est_41","Oxa_GeW_27","Oxa_SwS_E2"))## populations with less than 4 MLG is removed
poppr(oxacc_all)## there are repeated MLG in different Populations. these MLGs are kept
save(file="oxacc_all.RData", list="oxacc_all")
```


LS0tDQp0aXRsZTogIjAxX0RhdGFfUHJlcGFyYXRpb24iDQpvdXRwdXQ6IGh0bWxfbm90ZWJvb2sNCi0tLQ0KDQoNCmBgYHtyfQ0KbGlicmFyeShwb3BwcikNCmxpYnJhcnkocGVnYXMpDQpsaWJyYXJ5KHBvbHlzYXQpDQpsaWJyYXJ5KFJVdGlscykNCnNvdXJjZSgiUDovUEIyLVByb2plY3RzL0xhbmRnZW4vMDVfRGF0YSBhbmFseXNpcy9GdW5jdGlvbnMgZm9yIHBvcHVsYXRpb24gZ2VuZXRpY3MuciIpDQoNCiMjQW5lbW9uZSBuZW1vcm9zYQ0KQW5lPC1yZWFkLmNzdjIoIkFuZV9BbGxlbGVUYWJsZV9Fc3RfY29ycmVjdC5jc3YiLHNlcD0iLCIpDQpyb3duYW1lcyhBbmUpPC1BbmVbLDFdDQpBbmU8LUFuZVssLTFdDQojIyBwcmVwYXJlIGdlbmV0aWMgZGF0YSAgICAgICAgICAgICAgICAgICAgICANCmFuZS5sPC1hbGxlbGVzMmxvY2koQW5lLHBsb2lkeT00LHBvcHVsYXRpb249MSkNCmNvbG5hbWVzKGFuZS5sKSA8LSBzdWIoIl8xIiwgIiIsIGNvbG5hbWVzKGFuZS5sKSkgDQphbmUuZ2k8LWxvY2kyZ2VuaW5kKGFuZS5sLG5hLmFsbGVsZXM9Ii05OTkiLHBsb2lkeT00KSMjIHRoZXJlIGlzIG9uZSBpbmRpdiBpbiBCZSB3aXRob3V0IHN1Y2Vzc2Z1bCBnZW5vdHlwaW5nLCBpcyBleGNsdWRlZCBpbiB0aGlzIHN0ZXANCmFuZS5naWM8LWFuZS5naQ0KI2xldmVscyhhbmUuZ2ljQHBvcCkNCiNzdW1tYXJ5KGFuZS5naUBwb3ApDQojIyBwcmVwZXJhIHN0cmF0YQ0Kc3RyYXRzIDwtIGJhc2U6OnN0cnNwbGl0KGFzLmNoYXJhY3RlcihhbmUuZ2ljQHBvcCksICJfIikNCmFuZS5naWNAc3RyYXRhIDwtIGRhdGEuZnJhbWUoUG9wdWxhdGlvbiA9IGFuZS5naWNAcG9wLA0KICAgICAgICAgICAgICAgICAgICAgICAgICAgIFNwZWNpZXMgPSBzYXBwbHkoc3RyYXRzLCBmdW5jdGlvbih4KSB4W1sxXV0pLA0KICAgICAgICAgICAgICAgICAgICAgICAgICAgIFJlZ2lvbiA9IHNhcHBseShzdHJhdHMsIGZ1bmN0aW9uKHgpIHhbWzJdXSksDQogICAgICAgICAgICAgICAgICAgICAgICAgICAgRm9yZXN0UGF0Y2ggPSBzYXBwbHkoc3RyYXRzLCBmdW5jdGlvbih4KSB4W1szXV0pKQ0KIyMgdGhlcmUgYXJlIGluIHRvdGFsIDYgbG9jaSwgd2UgZXhjbHVkZSBpbmRpdmlkdWFscyB3aXRoIG1vcmUgdGhhbiBtaXNzaW5nIHZhbHVlIGluIDEgbG9jdXMgKDE3JSkgICAgICAgICAgICAgICAgICAgICAgICAgICAgDQppbmZvX3RhYmxlKG1pc3NpbmdubyhhbmUuZ2ljLCAiZ2VubyIsIGN1dG9mZj0wLjE3KSxwbG90PVQpIA0KIyMgcmVtb3ZlIGluZGl2aWR1YWxzIHdpdGggbW9yZSB0aGFuIDE3JSBtaXNzaW5nIHZhbHVlDQphbmUubmFybTwtbWlzc2luZ25vKGFuZS5naWMsICJnZW5vIiwgY3V0b2ZmPTAuMTcpDQojIyBzdW1tYXJ5IHRhYmxlDQpzdW10YWIuYW5lPC1wb3BwcihhbmUubmFybSwgcXVpZXQ9VCkNCnN1bXRhYi5hbmVbLDE6M10NCiMgcmVtb3ZlIGNsb25lcw0KYW5lY2NfYWxsIDwtIGNsb25lY29ycmVjdChhbmUubmFybSwgc3RyYXRhPX5Qb3B1bGF0aW9uKQ0Kc2F2ZShmaWxlPSJhbmVjY19hbGwuUkRhdGEiLCBsaXN0PSJhbmVjY19hbGwiKQ0KYGBgDQpgYGB7cn0NCiMjIFBvbHlnb25hdHVtIG11bHRpZmxvcnVtDQpQb2w8LXJlYWQuY3N2MigiUG9sX0FsbGVsZVRhYmxlLmNzdiIsc2VwPSIsIikNCnJvd25hbWVzKFBvbCk8LVBvbFssMV0NClBvbDwtUG9sWywtMV0NCnBvbC5sPC1hbGxlbGVzMmxvY2koUG9sLHBvcHVsYXRpb249MSkNCmNvbG5hbWVzKHBvbC5sKSA8LSBzdWIoIl8xIiwgIiIsIGNvbG5hbWVzKHBvbC5sKSkgDQpwb2wuZ2k8LWxvY2kyZ2VuaW5kKHBvbC5sLG5hLmFsbGVsZXM9Ii05OTkiKQ0Kc3VtbWFyeShwb2wuZ2lAcG9wKQ0KcG9sLmdpYzwtcG9wc3ViKHBvbC5naSxleGNsdWRlPWMoIlBvbF9Fc3RfMDIiLCJQb2xfRXN0XzYxIikpIyMgdGhlc2UgdHdvIHBvcHVsYXRpb25zIGFyZSBleGNsdWRlZCBkdWUgdG8gbG93IGluZGl2aWR1YWwgbnVtYmVyOjEgYW5kIDMgaW5kaXZpZHVhbHMNCmdlbm90eXBlX2N1cnZlKHBvbC5naWMpDQpzdHJhdHMgPC0gYmFzZTo6c3Ryc3BsaXQoYXMuY2hhcmFjdGVyKHBvbC5naWNAcG9wKSwgIl8iKQ0KcG9sLmdpY0BzdHJhdGEgPC0gZGF0YS5mcmFtZShQb3B1bGF0aW9uID0gcG9sLmdpY0Bwb3AsDQogICAgICAgICAgICAgICAgICAgICAgICAgICAgICAgU3BlY2llcyA9IHNhcHBseShzdHJhdHMsIGZ1bmN0aW9uKHgpIHhbWzFdXSksDQogICAgICAgICAgICAgICAgICAgICAgICAgICAgICAgUmVnaW9uID0gc2FwcGx5KHN0cmF0cywgZnVuY3Rpb24oeCkgeFtbMl1dKSwNCiAgICAgICAgICAgICAgICAgICAgICAgICAgICAgICBGb3Jlc3RQYXRjaCA9IHNhcHBseShzdHJhdHMsIGZ1bmN0aW9uKHgpIHhbWzNdXSkpDQojIyBzYW1lIGFzIGluIEFuZW1vbmUgbmVtb3Jvc2EsIHdlIGV4Y2x1ZGUgaW5kaXZpZHVhbHMgd2l0aCBtaXNzaW5nIHZhbHVlIGluIG1vcmUgdGhhbiAxIGxvY3VzDQppbmZvX3RhYmxlKG1pc3Npbmdubyhwb2wuZ2ljLCAiZ2VubyIsIGN1dG9mZj0wLjE3KSwgcGxvdD1UUlVFKSANCnBvbC5uYXJtPC1taXNzaW5nbm8ocG9sLmdpYywgImdlbm8iLCBjdXRvZmY9MC4xNykNCiMjcmVtb3ZlIGNsb25lcw0KcG9sY2NfYWxsPC1jbG9uZWNvcnJlY3QocG9sLm5hcm0sc3RyYXRhPX5Qb3B1bGF0aW9uKQ0Kc3VtbWFyeShwb2xjY19hbGxAcG9wKQ0KcG9sY2NfYWxsPC1wb3BzdWIocG9sY2NfYWxsLGV4Y2x1ZGU9YygiUG9sX0VzdF8wMSIsIlBvbF9Gck5fMDkiKSkjIyBwb3B1bGF0aW9ucyB3aXRoIGxlc3MgdGhhbiA0IE1MRyBpcyByZW1vdmVkDQpwb3Bwcihwb2xjY19hbGwpDQpzYXZlKGZpbGU9InBvbGNjX2FsbC5SRGF0YSIsIGxpc3Q9InBvbGNjX2FsbCIpDQoNCmBgYA0KYGBge3J9DQojI094YWxpcyBhY2V0b3NlbGxhDQpPeGE8LXJlYWQuY3N2MigiT3hhX0FsbGVsZVRhYmxlX0ZyTm1vdmVkLmNzdiIsc2VwPSIsIikNCnJvd25hbWVzKE94YSk8LU94YVssMV0NCk94YTwtT3hhWywtMV0NCm94YS5sPC1hbGxlbGVzMmxvY2koT3hhLHBvcHVsYXRpb249MSkNCmNvbG5hbWVzKG94YS5sKSA8LSBzdWIoIl8xIiwgIiIsIGNvbG5hbWVzKG94YS5sKSkgDQpveGEuZ2ljPC1veGEuZ2k8LWxvY2kyZ2VuaW5kKG94YS5sLG5hLmFsbGVsZXM9Ii05OTkiKQ0Kc3RyYXRzIDwtIGJhc2U6OnN0cnNwbGl0KGFzLmNoYXJhY3RlcihveGEuZ2ljQHBvcCksICJfIikNCm94YS5naWNAc3RyYXRhIDwtIGRhdGEuZnJhbWUoUG9wdWxhdGlvbiA9IG94YS5naWNAcG9wLA0KICAgICAgICAgICAgICAgICAgICAgICAgICAgIFNwZWNpZXMgPSBzYXBwbHkoc3RyYXRzLCBmdW5jdGlvbih4KSB4W1sxXV0pLA0KICAgICAgICAgICAgICAgICAgICAgICAgICAgIFJlZ2lvbiA9IHNhcHBseShzdHJhdHMsIGZ1bmN0aW9uKHgpIHhbWzJdXSksDQogICAgICAgICAgICAgICAgICAgICAgICAgICAgRm9yZXN0UGF0Y2ggPSBzYXBwbHkoc3RyYXRzLCBmdW5jdGlvbih4KSB4W1szXV0pKQ0KDQoNCmluZm9fdGFibGUobWlzc2luZ25vKG94YS5naWMsICJnZW5vIiwgY3V0b2ZmPTAuMSkscGxvdD1UKSANCm94YS5uYXJtPC1taXNzaW5nbm8ob3hhLmdpYywgImdlbm8iLCBjdXRvZmY9MC4xKQ0Kb3hhY2NfYWxsPC1jbG9uZWNvcnJlY3Qob3hhLm5hcm0sIHN0cmF0YT1+UG9wdWxhdGlvbikNCnN1bW1hcnkob3hhY2NfYWxsQHBvcCkNCm94YWNjX2FsbDwtcG9wc3ViKG94YWNjX2FsbCxleGNsdWRlPWMoIk94YV9HZUVfMDgiLCJPeGFfRXN0XzAzIiwiT3hhX0VzdF80MSIsIk94YV9HZVdfMjciLCJPeGFfU3dTX0UyIikpIyMgcG9wdWxhdGlvbnMgd2l0aCBsZXNzIHRoYW4gNCBNTEcgaXMgcmVtb3ZlZA0KcG9wcHIob3hhY2NfYWxsKSMjIHRoZXJlIGFyZSByZXBlYXRlZCBNTEcgaW4gZGlmZmVyZW50IFBvcHVsYXRpb25zLiB0aGVzZSBNTEdzIGFyZSBrZXB0DQpzYXZlKGZpbGU9Im94YWNjX2FsbC5SRGF0YSIsIGxpc3Q9Im94YWNjX2FsbCIpDQoNCmBgYA0KDQoNCg0KDQo=
