# Supplementary material for: Limited effects of population age on the genetic structure of spatially isolated forest herb populations in temperate Europe
Source: Ecol Evol. 2024 Feb 26;14(2):e10971. doi: 10.1002/ece3.10971 (PMC10897356; doi:10.1002/ece3.10971)
Supplement: Supplementary file 1 — Appendix S1. [file ECE3-14-e10971-s001.zip › 02_Genetic_Diversity.nb.html]

02\_Genetic Diversity


Code 

- Show All Code
- Hide All Code
- Download Rmd

# 02\_Genetic Diversity


```
## Allelic richness with rarefaction
Ar.pmul<-Ar(pmul.lc,ipop=1,subsample=16,assignNA=F,nsub=1000)$mNa## there are in total 967 MLG in 60 populations, averagely 16 MLG/Population
```


```
Error in Ar(pmul.lc, ipop = 1, subsample = 16, assignNA = F, nsub = 1000) : 
  could not find function "Ar"
```


```
## Anemone nemorosa
anem.lc<-genind2loci(anecc_all)
anecc_all_genambig<-as.genambig(anecc_all)
save(file="anecc_all_genambig.RData",list="anecc_all_genambig")
Ploidies(anecc_all_genambig)<-4

#allelic frequency with selfing rate. It takes a very long time
af.anem_DeSilva_all<-deSilvaFreq(anecc_all_genambig,
                       self=0.06, #the rate of selfing (between 0 and 1)
                       initNull=0.01)
#load("af.anem_DeSilva_all.RData")
##Allelic richness
anem.lc <- genind2loci(anecc_all)
#The function Ar requires a loci object, however, in loci objects, the number of alleles must 
# be the same in each sample (therefore open places are filled with "0"). But we can manually
# delete all "0" and give the "pruned" data manually the class attribute "loci".
ane.prun <- apply(anem.lc,2,function(x) {
                            sapply(strsplit(x, "/", fixed=TRUE), function(y) {
                              paste(y[y!="0"], collapse="/")
                            })
})
ane.prun <- as.data.frame(ane.prun)
                          
class(ane.prun) <- c("loci", "data.frame")
##Allelic richness
Ar.anem <- Ar(ane.prun, ipop=1, subsample=18,assignNA=F, nsub=1000)$mNa ## there are in total 1088 MLG in 60 Populations
## Ho
Ho.anem<- Hg(anecc_all_genambig, method="DeSilva", self=0.06, AllFreq = af.anem_DeSilva_all)

## He
He.anem <- He.poly(anecc_all_genambig, Ho=Ho.anem,AllFreq=af.anem_DeSilva_all, unbiased=T)
## Fis
gprobs.anem <- genoprob(anecc_all_genambig, freq=af.anem_DeSilva_all, self=0.06, all=TRUE)##could take a while
Fis.raw.ane<- Fis.test.poly(anecc_all_genambig, He.anem, Ho.anem, gprobs=gprobs.anem, perm=999, alternative="two-sided") #takes a long time
Fis.anem <- Fis.raw.ane$observed
Fis.pval.anem <- Fis.raw.ane$pval
```


```
```r
## Oxalis acetosella
oace.lc<- genind2loci(oxacc_all)
## Allelic richness
Ar.oxa <- Ar(oace.lc, ipop=1, subsample=16, assignNA = F,nsub=1000)$mNa## 597 individuals with 576 MLG in 37 populations 
## He
He.oxa<- He(oace.lc, iloc=2:ncol(oace.lc), ipop=1, unbiased=TRUE) #He according to GeneAlEx (--> equal to uHe in GeneAlEx (Command \Frequencies...\)
## Ho
Ho.oxa <- Ho(oace.lc, iloc=2:ncol(oace.lc), ipop=1) #--> equal to Ho in GeneAlEx
## Fis
Fis.raw.oxa <- Fis.test(oace.lc, method=\unbiased\, alternative=\two-sided\, perm=999, iloc=2:ncol(oace.lc), ipop=1)
Fis.oace <- Fis.raw.oxa$observed
Fis.pval.oace <- Fis.raw.oxa$pval
```

```
<!-- rnb-source-end -->

<!-- rnb-chunk-end -->


<!-- rnb-chunk-begin -->


<!-- rnb-source-begin eyJkYXRhIjoiYGBgclxuYGBgclxuR2VuRGl2X2FsbC5wbXVsIDwtIGRhdEdlbkRpdi5wbXVsIDwtIGRhdGEuZnJhbWUoTj1zdW1tYXJ5KHBvbGNjX2FsbEBwb3ApLCBBcj1Bci5wbXVsLFxuICAgICAgICAgICAgICAgICAgICAgICAgICAgICAgICAgICAgICAgICAgICBIZT1hcHBseShIZS5wbXVsLDEsZnVuY3Rpb24oeCkgbWVhbih4LG5hLnJtID1UKSksIFxuICAgICAgICAgICAgICAgICAgICAgICAgICAgICAgICAgICAgICAgICAgICBIbz1hcHBseShIby5wbXVsLDEsZnVuY3Rpb24oeCkgbWVhbih4LG5hLnJtPVQpKSwgXG4gICAgICAgICAgICAgICAgICAgICAgICAgICAgICAgICAgICAgICAgICAgIEZpcz1GaXMucG11bCwgRmlzLnB2YWw9RmlzLnB2YWwucG11bClcblxuR2VuRGl2X2FsbC5hbmVtIDwtIGRhdEdlbkRpdi5hbmVtIDwtIGRhdGEuZnJhbWUoTj1zdW1tYXJ5KGFuZWNjX2FsbEBwb3ApLCBBcj1Bci5hbmVtLFxuICAgICAgICAgICAgICAgICAgICAgICAgICAgICAgICAgICAgSGU9YXBwbHkoSGUuYW5lbSwxLGZ1bmN0aW9uKHgpIG1lYW4oeCwgbmEucm0gPSBUKSksIFxuICAgICAgICAgICAgICAgICAgICAgICAgICAgICAgICAgICAgSG89YXBwbHkoSG8uYW5lbSwxLGZ1bmN0aW9uKHgpIG1lYW4oeCwgbmEucm09VCkpLCBGaXM9RmlzLmFuZW0sIEZpcy5wdmFsPUZpcy5wdmFsLmFuZW0pXG5cbkdlbkRpdl9hbGwub2FjZTwtIGRhdEdlbkRpdi5vYWNlIDwtIGRhdGEuZnJhbWUoTj1zdW1tYXJ5KG94YWNjX2FsbEBwb3ApLCBBcj1Bci5veGEsXG4gICAgICAgICAgICAgICAgICAgICAgICAgICAgICAgICAgICBIZT1hcHBseShIZS5veGEsMSxtZWFuKSwgXG4gICAgICAgICAgICAgICAgICAgICAgICAgICAgICAgICAgICBIbz1hcHBseShIby5veGEsMSxtZWFuKSwgRmlzPUZpcy5vYWNlLCBGaXMucHZhbD1GaXMucHZhbC5vYWNlKVxuXG5cbnNhdmUoZmlsZT1cXEdlbkRpdl9hbGwucG11bC5SRGF0YVxcLGxpc3Q9XFxHZW5EaXZfYWxsLnBtdWxcXClcbnNhdmUoZmlsZT1cXEdlbkRpdl9hbGwuYW5lbS5SRGF0YVxcLGxpc3Q9XFxHZW5EaXZfYWxsLmFuZW1cXClcbnNhdmUoZmlsZT1cXEdlbkRpdl9hbGwub2FjZS5SRGF0YVxcLGxpc3Q9XFxHZW5EaXZfYWxsLm9hY2VcXClcbmBgYFxuYGBgIn0= -->

```r
```r
GenDiv_all.pmul <- datGenDiv.pmul <- data.frame(N=summary(polcc_all@pop), Ar=Ar.pmul,
                                            He=apply(He.pmul,1,function(x) mean(x,na.rm =T)), 
                                            Ho=apply(Ho.pmul,1,function(x) mean(x,na.rm=T)), 
                                            Fis=Fis.pmul, Fis.pval=Fis.pval.pmul)

GenDiv_all.anem <- datGenDiv.anem <- data.frame(N=summary(anecc_all@pop), Ar=Ar.anem,
                                    He=apply(He.anem,1,function(x) mean(x, na.rm = T)), 
                                    Ho=apply(Ho.anem,1,function(x) mean(x, na.rm=T)), Fis=Fis.anem, Fis.pval=Fis.pval.anem)

GenDiv_all.oace<- datGenDiv.oace <- data.frame(N=summary(oxacc_all@pop), Ar=Ar.oxa,
                                    He=apply(He.oxa,1,mean), 
                                    Ho=apply(Ho.oxa,1,mean), Fis=Fis.oace, Fis.pval=Fis.pval.oace)


save(file=\GenDiv_all.pmul.RData\,list=\GenDiv_all.pmul\)
save(file=\GenDiv_all.anem.RData\,list=\GenDiv_all.anem\)
save(file=\GenDiv_all.oace.RData\,list=\GenDiv_all.oace\)
```

```


LS0tDQp0aXRsZTogIjAyX0dlbmV0aWMgRGl2ZXJzaXR5Ig0Kb3V0cHV0OiBodG1sX25vdGVib29rDQotLS0NCg0KDQpgYGB7cn0NCmxpYnJhcnkocG9wcHIpDQpsaWJyYXJ5KHBlZ2FzKQ0KbGlicmFyeShwb2x5c2F0KQ0KbGlicmFyeShST0RCQykNCmxpYnJhcnkodmVnYW4pDQpsaWJyYXJ5KFJVdGlscykNCmxpYnJhcnkocmVzaGFwZTIpDQpsaWJyYXJ5KGdncGxvdDIpDQpsaWJyYXJ5KGRwbHlyKQ0Kc291cmNlKCJMOi8wNV9EYXRhIGFuYWx5c2lzL0Z1bmN0aW9ucyBmb3IgcG9wdWxhdGlvbiBnZW5ldGljcy5yIikNCmxpYnJhcnkoZGF0YS50YWJsZSkNCg0KbG9hZCgicG9sY2NfYWxsLlJEYXRhIikNCmxvYWQoIm94YWNjX2FsbC5SRGF0YSIpDQpsb2FkKCJhbmVjY19hbGwuUkRhdGEiKQ0KDQojI1BvbHlnb25hdHVtIG11bHRpZmxvcnVtDQpwbXVsLmxjPC0gZ2VuaW5kMmxvY2kocG9sY2NfYWxsKQ0KIyMgQWxsZWxpYyByaWNobmVzcyB3aXRoIHJhcmVmYWN0aW9uDQpBci5wbXVsPC1BcihwbXVsLmxjLGlwb3A9MSxzdWJzYW1wbGU9MTYsYXNzaWduTkE9Rixuc3ViPTEwMDApJG1OYSMjIHRoZXJlIGFyZSBpbiB0b3RhbCA5NjcgTUxHIGluIDYwIHBvcHVsYXRpb25zLCBhdmVyYWdlbHkgMTYgTUxHL1BvcHVsYXRpb24NCiMjIEhlDQpIZS5wbXVsPC0gSGUocG11bC5sYywgaWxvYz0yOm5jb2wocG11bC5sYyksIGlwb3A9MSwgdW5iaWFzZWQ9VFJVRSkgDQojIyBIbw0KSG8ucG11bCA8LSBIbyhwbXVsLmxjLCBpbG9jPTI6bmNvbChwbXVsLmxjKSwgaXBvcD0xKQ0KIyMgRmlzDQpGaXMucmF3LnBvbCA8LSBGaXMudGVzdChwbXVsLmxjLCBtZXRob2Q9InVuYmlhc2VkIiwgYWx0ZXJuYXRpdmU9InR3by1zaWRlZCIsIHBlcm09OTk5LCBpbG9jPTI6bmNvbChwbXVsLmxjKSwgaXBvcD0xKSMjIHRha2VzIHNvbWUgdGltZQ0KRmlzLnBtdWwgPC0gRmlzLnJhdy5wb2wkb2JzZXJ2ZWQNCkZpcy5wdmFsLnBtdWwgPC0gRmlzLnJhdy5wb2wkcHZhbA0KYGBgDQoNCg0KYGBge3J9DQojIyBBbmVtb25lIG5lbW9yb3NhDQphbmVtLmxjPC1nZW5pbmQybG9jaShhbmVjY19hbGwpDQphbmVjY19hbGxfZ2VuYW1iaWc8LWFzLmdlbmFtYmlnKGFuZWNjX2FsbCkNCnNhdmUoZmlsZT0iYW5lY2NfYWxsX2dlbmFtYmlnLlJEYXRhIixsaXN0PSJhbmVjY19hbGxfZ2VuYW1iaWciKQ0KUGxvaWRpZXMoYW5lY2NfYWxsX2dlbmFtYmlnKTwtNA0KDQojYWxsZWxpYyBmcmVxdWVuY3kgd2l0aCBzZWxmaW5nIHJhdGUuIEl0IHRha2VzIGEgdmVyeSBsb25nIHRpbWUNCmFmLmFuZW1fRGVTaWx2YV9hbGw8LWRlU2lsdmFGcmVxKGFuZWNjX2FsbF9nZW5hbWJpZywNCiAgICAgICAgICAgICAgICAgICAgICAgc2VsZj0wLjA2LCAjdGhlIHJhdGUgb2Ygc2VsZmluZyAoYmV0d2VlbiAwIGFuZCAxKQ0KICAgICAgICAgICAgICAgICAgICAgICBpbml0TnVsbD0wLjAxKQ0KI2xvYWQoImFmLmFuZW1fRGVTaWx2YV9hbGwuUkRhdGEiKQ0KIyNBbGxlbGljIHJpY2huZXNzDQphbmVtLmxjIDwtIGdlbmluZDJsb2NpKGFuZWNjX2FsbCkNCiNUaGUgZnVuY3Rpb24gQXIgcmVxdWlyZXMgYSBsb2NpIG9iamVjdCwgaG93ZXZlciwgaW4gbG9jaSBvYmplY3RzLCB0aGUgbnVtYmVyIG9mIGFsbGVsZXMgbXVzdCANCiMgYmUgdGhlIHNhbWUgaW4gZWFjaCBzYW1wbGUgKHRoZXJlZm9yZSBvcGVuIHBsYWNlcyBhcmUgZmlsbGVkIHdpdGggIjAiKS4gQnV0IHdlIGNhbiBtYW51YWxseQ0KIyBkZWxldGUgYWxsICIwIiBhbmQgZ2l2ZSB0aGUgInBydW5lZCIgZGF0YSBtYW51YWxseSB0aGUgY2xhc3MgYXR0cmlidXRlICJsb2NpIi4NCmFuZS5wcnVuIDwtIGFwcGx5KGFuZW0ubGMsMixmdW5jdGlvbih4KSB7DQogICAgICAgICAgICAgICAgICAgICAgICAgICAgc2FwcGx5KHN0cnNwbGl0KHgsICIvIiwgZml4ZWQ9VFJVRSksIGZ1bmN0aW9uKHkpIHsNCiAgICAgICAgICAgICAgICAgICAgICAgICAgICAgIHBhc3RlKHlbeSE9IjAiXSwgY29sbGFwc2U9Ii8iKQ0KICAgICAgICAgICAgICAgICAgICAgICAgICAgIH0pDQp9KQ0KYW5lLnBydW4gPC0gYXMuZGF0YS5mcmFtZShhbmUucHJ1bikNCiAgICAgICAgICAgICAgICAgICAgICAgICAgDQpjbGFzcyhhbmUucHJ1bikgPC0gYygibG9jaSIsICJkYXRhLmZyYW1lIikNCiMjQWxsZWxpYyByaWNobmVzcw0KQXIuYW5lbSA8LSBBcihhbmUucHJ1biwgaXBvcD0xLCBzdWJzYW1wbGU9MTgsYXNzaWduTkE9RiwgbnN1Yj0xMDAwKSRtTmEgIyMgdGhlcmUgYXJlIGluIHRvdGFsIDEwODggTUxHIGluIDYwIFBvcHVsYXRpb25zDQojIyBIbw0KSG8uYW5lbTwtIEhnKGFuZWNjX2FsbF9nZW5hbWJpZywgbWV0aG9kPSJEZVNpbHZhIiwgc2VsZj0wLjA2LCBBbGxGcmVxID0gYWYuYW5lbV9EZVNpbHZhX2FsbCkNCg0KIyMgSGUNCkhlLmFuZW0gPC0gSGUucG9seShhbmVjY19hbGxfZ2VuYW1iaWcsIEhvPUhvLmFuZW0sQWxsRnJlcT1hZi5hbmVtX0RlU2lsdmFfYWxsLCB1bmJpYXNlZD1UKQ0KIyMgRmlzDQpncHJvYnMuYW5lbSA8LSBnZW5vcHJvYihhbmVjY19hbGxfZ2VuYW1iaWcsIGZyZXE9YWYuYW5lbV9EZVNpbHZhX2FsbCwgc2VsZj0wLjA2LCBhbGw9VFJVRSkjI2NvdWxkIHRha2UgYSB3aGlsZQ0KRmlzLnJhdy5hbmU8LSBGaXMudGVzdC5wb2x5KGFuZWNjX2FsbF9nZW5hbWJpZywgSGUuYW5lbSwgSG8uYW5lbSwgZ3Byb2JzPWdwcm9icy5hbmVtLCBwZXJtPTk5OSwgYWx0ZXJuYXRpdmU9InR3by1zaWRlZCIpICN0YWtlcyBhIGxvbmcgdGltZQ0KRmlzLmFuZW0gPC0gRmlzLnJhdy5hbmUkb2JzZXJ2ZWQNCkZpcy5wdmFsLmFuZW0gPC0gRmlzLnJhdy5hbmUkcHZhbA0KDQpgYGANCmBgYHtyfQ0KIyMgT3hhbGlzIGFjZXRvc2VsbGENCm9hY2UubGM8LSBnZW5pbmQybG9jaShveGFjY19hbGwpDQojIyBBbGxlbGljIHJpY2huZXNzDQpBci5veGEgPC0gQXIob2FjZS5sYywgaXBvcD0xLCBzdWJzYW1wbGU9MTYsIGFzc2lnbk5BID0gRixuc3ViPTEwMDApJG1OYSMjIDU5NyBpbmRpdmlkdWFscyB3aXRoIDU3NiBNTEcgaW4gMzcgcG9wdWxhdGlvbnMgDQojIyBIZQ0KSGUub3hhPC0gSGUob2FjZS5sYywgaWxvYz0yOm5jb2wob2FjZS5sYyksIGlwb3A9MSwgdW5iaWFzZWQ9VFJVRSkgI0hlIGFjY29yZGluZyB0byBHZW5lQWxFeCAoLS0+IGVxdWFsIHRvIHVIZSBpbiBHZW5lQWxFeCAoQ29tbWFuZCAiRnJlcXVlbmNpZXMuLi4iKQ0KIyMgSG8NCkhvLm94YSA8LSBIbyhvYWNlLmxjLCBpbG9jPTI6bmNvbChvYWNlLmxjKSwgaXBvcD0xKSAjLS0+IGVxdWFsIHRvIEhvIGluIEdlbmVBbEV4DQojIyBGaXMNCkZpcy5yYXcub3hhIDwtIEZpcy50ZXN0KG9hY2UubGMsIG1ldGhvZD0idW5iaWFzZWQiLCBhbHRlcm5hdGl2ZT0idHdvLXNpZGVkIiwgcGVybT05OTksIGlsb2M9MjpuY29sKG9hY2UubGMpLCBpcG9wPTEpDQpGaXMub2FjZSA8LSBGaXMucmF3Lm94YSRvYnNlcnZlZA0KRmlzLnB2YWwub2FjZSA8LSBGaXMucmF3Lm94YSRwdmFsDQpgYGANCmBgYHtyfQ0KR2VuRGl2X2FsbC5wbXVsIDwtIGRhdEdlbkRpdi5wbXVsIDwtIGRhdGEuZnJhbWUoTj1zdW1tYXJ5KHBvbGNjX2FsbEBwb3ApLCBBcj1Bci5wbXVsLA0KICAgICAgICAgICAgICAgICAgICAgICAgICAgICAgICAgICAgICAgICAgICBIZT1hcHBseShIZS5wbXVsLDEsZnVuY3Rpb24oeCkgbWVhbih4LG5hLnJtID1UKSksIA0KICAgICAgICAgICAgICAgICAgICAgICAgICAgICAgICAgICAgICAgICAgICBIbz1hcHBseShIby5wbXVsLDEsZnVuY3Rpb24oeCkgbWVhbih4LG5hLnJtPVQpKSwgDQogICAgICAgICAgICAgICAgICAgICAgICAgICAgICAgICAgICAgICAgICAgIEZpcz1GaXMucG11bCwgRmlzLnB2YWw9RmlzLnB2YWwucG11bCkNCg0KR2VuRGl2X2FsbC5hbmVtIDwtIGRhdEdlbkRpdi5hbmVtIDwtIGRhdGEuZnJhbWUoTj1zdW1tYXJ5KGFuZWNjX2FsbEBwb3ApLCBBcj1Bci5hbmVtLA0KICAgICAgICAgICAgICAgICAgICAgICAgICAgICAgICAgICAgSGU9YXBwbHkoSGUuYW5lbSwxLGZ1bmN0aW9uKHgpIG1lYW4oeCwgbmEucm0gPSBUKSksIA0KICAgICAgICAgICAgICAgICAgICAgICAgICAgICAgICAgICAgSG89YXBwbHkoSG8uYW5lbSwxLGZ1bmN0aW9uKHgpIG1lYW4oeCwgbmEucm09VCkpLCBGaXM9RmlzLmFuZW0sIEZpcy5wdmFsPUZpcy5wdmFsLmFuZW0pDQoNCkdlbkRpdl9hbGwub2FjZTwtIGRhdEdlbkRpdi5vYWNlIDwtIGRhdGEuZnJhbWUoTj1zdW1tYXJ5KG94YWNjX2FsbEBwb3ApLCBBcj1Bci5veGEsDQogICAgICAgICAgICAgICAgICAgICAgICAgICAgICAgICAgICBIZT1hcHBseShIZS5veGEsMSxtZWFuKSwgDQogICAgICAgICAgICAgICAgICAgICAgICAgICAgICAgICAgICBIbz1hcHBseShIby5veGEsMSxtZWFuKSwgRmlzPUZpcy5vYWNlLCBGaXMucHZhbD1GaXMucHZhbC5vYWNlKQ0KDQoNCnNhdmUoZmlsZT0iR2VuRGl2X2FsbC5wbXVsLlJEYXRhIixsaXN0PSJHZW5EaXZfYWxsLnBtdWwiKQ0Kc2F2ZShmaWxlPSJHZW5EaXZfYWxsLmFuZW0uUkRhdGEiLGxpc3Q9IkdlbkRpdl9hbGwuYW5lbSIpDQpzYXZlKGZpbGU9IkdlbkRpdl9hbGwub2FjZS5SRGF0YSIsbGlzdD0iR2VuRGl2X2FsbC5vYWNlIikNCmBgYA0KDQoNCg==
