# Supplementary material for: Limited effects of population age on the genetic structure of spatially isolated forest herb populations in temperate Europe
Source: Ecol Evol. 2024 Feb 26;14(2):e10971. doi: 10.1002/ece3.10971 (PMC10897356; doi:10.1002/ece3.10971)
Supplement: Supplementary file 1 — Appendix S1. [file ECE3-14-e10971-s001.zip › 03_Genetic_Differentiation.nb.html]

03\_Genetic\_Differentiation


Code 

- Show All Code
- Hide All Code
- Download Rmd

# 03\_Genetic\_Differentiation


```
library(poppr)
library(pegas)
library(polysat)
library(RUtils)
library(dplyr)
library(stringr)
library(otuSummary)
source("P:/PB2-Projects/Landgen/05_Data analysis/Functions for population genetics.r")
load("polcc_all.RData") 
load("oxacc_all.RData")
load("anecc_all.RData")
load("anecc_all_genambig.RData") 
load("af.anem_DeSilva_all.Rdata")
```


Pairwise G statistics among populations within regions (G”st)


```
pmul.lc <- genind2loci(polcc_all)
region.pmul <- sapply(strsplit(as.character(pmul.lc[[1]]),"_"), function(x) x[2])


oace.lc<-genind2loci(oxacc_all)
region.oace <- sapply(strsplit(as.character(oace.lc[[1]]),"_"), function(x) x[2])


region.anem <- str_sort(sapply(strsplit(PopNames(anecc_all_genambig),"_"), function(x) x[2]))## for the population name of allele table of anemone was not ordered alphabetically. here use this function to make the region of anem fit to the population name of af.anem_DeSilva_all

## Polygonatum multiflorum
Gpair.pmul <- PairDiff(pmul.lc, strat=region.pmul, across.loci = TRUE)
GstL.pmul<-lapply(Gpair.pmul,function(x) matrixConvert(as.matrix(x$GstNeiHed),colname=c("IN_POP","NEAR_POP","Dist")))
GstDF.pmul <- GstL.pmul[[1]]
for(r in 2:length(GstL.pmul)) GstDF.pmul <- rbind(GstDF.pmul, GstL.pmul[[r]]) 
colnames(GstDF.pmul)<-c("IN_POP","NEAR_POP","Gst")
```


```
## Oxalis acetosella
Gpair.oace<-PairDiff(oace.lc,strat=region.oace,across.loci=T)
GstL.oace<-lapply(Gpair.oace,function(x) matrixConvert(as.matrix(x$GstNeiHed)))
GstDF.oace <- GstL.oace[[1]]
for(r in 2:length(GstL.oace)) GstDF.oace <- rbind(GstDF.oace, GstL.oace[[r]]) 
colnames(GstDF.oace)<-c("IN_POP","NEAR_POP","Gst")
```


```
## Anemone nemorosa
Gpair.anem <- PairDiff.poly(af.anem_DeSilva_all, stats=c("GstNeiHed"), strat=region.anem, across.loci=TRUE)
GstL.anem<-lapply(Gpair.anem,function(x) matrixConvert(as.matrix(x$GstNeiHed)))
GstDF.anem <- GstL.anem[[1]]
for(r in 2:length(GstL.anem)) GstDF.anem <- rbind(GstDF.anem, GstL.anem[[r]]) 
colnames(GstDF.anem)<-c("IN_POP","NEAR_POP","Gst")
```


Proportion of shared allele (Dps)


```
library(PopGenReport)
## Polygonatum multiflorum
reg.pmul <- sort(unique(polcc_all@strata$Region)) 
Dps.pmul <- list()
for(r in reg.pmul) {
  Dps.pmul[[r]] <- 1 - pairwise.propShared(stratsub(polcc_all, level="Region", stratum=r))
}
DpsL.pmul<-lapply(Dps.pmul, function(x) matrixConvert(as.matrix(x)))
DpsDF.pmul <- DpsL.pmul[[1]]
for(r in 2:length(DpsL.pmul)) DpsDF.pmul<-rbind(DpsDF.pmul, DpsL.pmul[[r]]) 
colnames(DpsDF.pmul)<-c("IN_POP","NEAR_POP","Dps")
```


```
## Oxalis acetosella
reg.oace <- sort(unique(oxacc_all@strata$Region)) 
Dps.oace <- list()
for(r in reg.oace) {
  Dps.oace[[r]] <- 1 - pairwise.propShared(stratsub(oxacc_all, level="Region", stratum=r))
}

DpsL.oace<-lapply(Dps.oace, function(x) matrixConvert(as.matrix(x)))
DpsDF.oace <- DpsL.oace[[1]]
for(r in 2:length(DpsL.oace)) DpsDF.oace<-rbind(DpsDF.oace, DpsL.oace[[r]]) 

colnames(DpsDF.oace)<-c("IN_POP","NEAR_POP","Dps")
```


```
## Anemone nemorosa
Dps.anem <- list()
for (r in unique(region.anem)) {
  Dps.anem[[r]] <- Dps(af.anem_DeSilva_all[region.anem==r,-1])
}
DpsL.anem<-lapply(Dps.anem,function(x) matrixConvert(as.matrix(x)))
DpsDF.anem <- DpsL.anem[[1]]
for(r in 2:length(DpsL.anem)) DpsDF.anem<-rbind(DpsDF.anem, DpsL.anem[[r]]) 
colnames(DpsDF.anem)<-c("IN_POP","NEAR_POP","Dps")
```


```
## Data Storage
PairDiff_all.pmul<-data.frame(IN_POP=GstDF.pmul$IN_POP,NEAR_POP=GstDF.pmul$NEAR_POP,
                          Gst=GstDF.pmul$Gst,Dps=DpsDF.pmul$Dps)


PairDiff_all.oace<-data.frame(IN_POP=GstDF.oace$IN_POP,NEAR_POP=GstDF.oace$NEAR_POP,
                          Gst=GstDF.oace$Gst,Dps=DpsDF.oace$Dps)


PairDiff_all.anem<-data.frame(IN_POP=GstDF.anem$IN_POP,NEAR_POP=GstDF.anem$NEAR_POP,
                          Gst=GstDF.anem$Gst,Dps=DpsDF.anem$Dps)


save(file="PairDiff_all_pmul.RData",list="PairDiff_all.pmul")
save(file="PairDiff_all_oace.RData",list="PairDiff_all.oace")
save(file="PairDiff_all_anem.RData",list="PairDiff_all.anem")
```


LS0tDQp0aXRsZTogIjAzX0dlbmV0aWNfRGlmZmVyZW50aWF0aW9uIg0Kb3V0cHV0OiBodG1sX25vdGVib29rDQotLS0NCg0KDQpgYGB7cn0NCmxpYnJhcnkocG9wcHIpDQpsaWJyYXJ5KHBlZ2FzKQ0KbGlicmFyeShwb2x5c2F0KQ0KbGlicmFyeShSVXRpbHMpDQpsaWJyYXJ5KGRwbHlyKQ0KbGlicmFyeShzdHJpbmdyKQ0KbGlicmFyeShvdHVTdW1tYXJ5KQ0Kc291cmNlKCJQOi9QQjItUHJvamVjdHMvTGFuZGdlbi8wNV9EYXRhIGFuYWx5c2lzL0Z1bmN0aW9ucyBmb3IgcG9wdWxhdGlvbiBnZW5ldGljcy5yIikNCmxvYWQoInBvbGNjX2FsbC5SRGF0YSIpIA0KbG9hZCgib3hhY2NfYWxsLlJEYXRhIikNCmxvYWQoImFuZWNjX2FsbC5SRGF0YSIpDQpsb2FkKCJhbmVjY19hbGxfZ2VuYW1iaWcuUkRhdGEiKSANCmxvYWQoImFmLmFuZW1fRGVTaWx2YV9hbGwuUmRhdGEiKSANCmBgYA0KUGFpcndpc2UgRyBzdGF0aXN0aWNzIGFtb25nIHBvcHVsYXRpb25zIHdpdGhpbiByZWdpb25zIChHInN0KQ0KYGBge3J9DQpwbXVsLmxjIDwtIGdlbmluZDJsb2NpKHBvbGNjX2FsbCkNCnJlZ2lvbi5wbXVsIDwtIHNhcHBseShzdHJzcGxpdChhcy5jaGFyYWN0ZXIocG11bC5sY1tbMV1dKSwiXyIpLCBmdW5jdGlvbih4KSB4WzJdKQ0KDQoNCm9hY2UubGM8LWdlbmluZDJsb2NpKG94YWNjX2FsbCkNCnJlZ2lvbi5vYWNlIDwtIHNhcHBseShzdHJzcGxpdChhcy5jaGFyYWN0ZXIob2FjZS5sY1tbMV1dKSwiXyIpLCBmdW5jdGlvbih4KSB4WzJdKQ0KDQoNCnJlZ2lvbi5hbmVtIDwtIHN0cl9zb3J0KHNhcHBseShzdHJzcGxpdChQb3BOYW1lcyhhbmVjY19hbGxfZ2VuYW1iaWcpLCJfIiksIGZ1bmN0aW9uKHgpIHhbMl0pKSMjIGZvciB0aGUgcG9wdWxhdGlvbiBuYW1lIG9mIGFsbGVsZSB0YWJsZSBvZiBhbmVtb25lIHdhcyBub3Qgb3JkZXJlZCBhbHBoYWJldGljYWxseS4gaGVyZSB1c2UgdGhpcyBmdW5jdGlvbiB0byBtYWtlIHRoZSByZWdpb24gb2YgYW5lbSBmaXQgdG8gdGhlIHBvcHVsYXRpb24gbmFtZSBvZiBhZi5hbmVtX0RlU2lsdmFfYWxsDQoNCiMjIFBvbHlnb25hdHVtIG11bHRpZmxvcnVtDQpHcGFpci5wbXVsIDwtIFBhaXJEaWZmKHBtdWwubGMsIHN0cmF0PXJlZ2lvbi5wbXVsLCBhY3Jvc3MubG9jaSA9IFRSVUUpDQpHc3RMLnBtdWw8LWxhcHBseShHcGFpci5wbXVsLGZ1bmN0aW9uKHgpIG1hdHJpeENvbnZlcnQoYXMubWF0cml4KHgkR3N0TmVpSGVkKSxjb2xuYW1lPWMoIklOX1BPUCIsIk5FQVJfUE9QIiwiRGlzdCIpKSkNCkdzdERGLnBtdWwgPC0gR3N0TC5wbXVsW1sxXV0NCmZvcihyIGluIDI6bGVuZ3RoKEdzdEwucG11bCkpIEdzdERGLnBtdWwgPC0gcmJpbmQoR3N0REYucG11bCwgR3N0TC5wbXVsW1tyXV0pIA0KY29sbmFtZXMoR3N0REYucG11bCk8LWMoIklOX1BPUCIsIk5FQVJfUE9QIiwiR3N0IikNCmBgYA0KYGBge3J9DQojIyBPeGFsaXMgYWNldG9zZWxsYQ0KR3BhaXIub2FjZTwtUGFpckRpZmYob2FjZS5sYyxzdHJhdD1yZWdpb24ub2FjZSxhY3Jvc3MubG9jaT1UKQ0KR3N0TC5vYWNlPC1sYXBwbHkoR3BhaXIub2FjZSxmdW5jdGlvbih4KSBtYXRyaXhDb252ZXJ0KGFzLm1hdHJpeCh4JEdzdE5laUhlZCkpKQ0KR3N0REYub2FjZSA8LSBHc3RMLm9hY2VbWzFdXQ0KZm9yKHIgaW4gMjpsZW5ndGgoR3N0TC5vYWNlKSkgR3N0REYub2FjZSA8LSByYmluZChHc3RERi5vYWNlLCBHc3RMLm9hY2VbW3JdXSkgDQpjb2xuYW1lcyhHc3RERi5vYWNlKTwtYygiSU5fUE9QIiwiTkVBUl9QT1AiLCJHc3QiKQ0KDQpgYGANCmBgYHtyfQ0KIyMgQW5lbW9uZSBuZW1vcm9zYQ0KR3BhaXIuYW5lbSA8LSBQYWlyRGlmZi5wb2x5KGFmLmFuZW1fRGVTaWx2YV9hbGwsIHN0YXRzPWMoIkdzdE5laUhlZCIpLCBzdHJhdD1yZWdpb24uYW5lbSwgYWNyb3NzLmxvY2k9VFJVRSkNCkdzdEwuYW5lbTwtbGFwcGx5KEdwYWlyLmFuZW0sZnVuY3Rpb24oeCkgbWF0cml4Q29udmVydChhcy5tYXRyaXgoeCRHc3ROZWlIZWQpKSkNCkdzdERGLmFuZW0gPC0gR3N0TC5hbmVtW1sxXV0NCmZvcihyIGluIDI6bGVuZ3RoKEdzdEwuYW5lbSkpIEdzdERGLmFuZW0gPC0gcmJpbmQoR3N0REYuYW5lbSwgR3N0TC5hbmVtW1tyXV0pIA0KY29sbmFtZXMoR3N0REYuYW5lbSk8LWMoIklOX1BPUCIsIk5FQVJfUE9QIiwiR3N0IikNCg0KYGBgDQpQcm9wb3J0aW9uIG9mIHNoYXJlZCBhbGxlbGUgKERwcykNCmBgYHtyfQ0KbGlicmFyeShQb3BHZW5SZXBvcnQpDQojIyBQb2x5Z29uYXR1bSBtdWx0aWZsb3J1bQ0KcmVnLnBtdWwgPC0gc29ydCh1bmlxdWUocG9sY2NfYWxsQHN0cmF0YSRSZWdpb24pKSANCkRwcy5wbXVsIDwtIGxpc3QoKQ0KZm9yKHIgaW4gcmVnLnBtdWwpIHsNCiAgRHBzLnBtdWxbW3JdXSA8LSAxIC0gcGFpcndpc2UucHJvcFNoYXJlZChzdHJhdHN1Yihwb2xjY19hbGwsIGxldmVsPSJSZWdpb24iLCBzdHJhdHVtPXIpKQ0KfQ0KRHBzTC5wbXVsPC1sYXBwbHkoRHBzLnBtdWwsIGZ1bmN0aW9uKHgpIG1hdHJpeENvbnZlcnQoYXMubWF0cml4KHgpKSkNCkRwc0RGLnBtdWwgPC0gRHBzTC5wbXVsW1sxXV0NCmZvcihyIGluIDI6bGVuZ3RoKERwc0wucG11bCkpIERwc0RGLnBtdWw8LXJiaW5kKERwc0RGLnBtdWwsIERwc0wucG11bFtbcl1dKSANCmNvbG5hbWVzKERwc0RGLnBtdWwpPC1jKCJJTl9QT1AiLCJORUFSX1BPUCIsIkRwcyIpDQoNCmBgYA0KYGBge3J9DQojIyBPeGFsaXMgYWNldG9zZWxsYQ0KcmVnLm9hY2UgPC0gc29ydCh1bmlxdWUob3hhY2NfYWxsQHN0cmF0YSRSZWdpb24pKSANCkRwcy5vYWNlIDwtIGxpc3QoKQ0KZm9yKHIgaW4gcmVnLm9hY2UpIHsNCiAgRHBzLm9hY2VbW3JdXSA8LSAxIC0gcGFpcndpc2UucHJvcFNoYXJlZChzdHJhdHN1YihveGFjY19hbGwsIGxldmVsPSJSZWdpb24iLCBzdHJhdHVtPXIpKQ0KfQ0KDQpEcHNMLm9hY2U8LWxhcHBseShEcHMub2FjZSwgZnVuY3Rpb24oeCkgbWF0cml4Q29udmVydChhcy5tYXRyaXgoeCkpKQ0KRHBzREYub2FjZSA8LSBEcHNMLm9hY2VbWzFdXQ0KZm9yKHIgaW4gMjpsZW5ndGgoRHBzTC5vYWNlKSkgRHBzREYub2FjZTwtcmJpbmQoRHBzREYub2FjZSwgRHBzTC5vYWNlW1tyXV0pIA0KDQpjb2xuYW1lcyhEcHNERi5vYWNlKTwtYygiSU5fUE9QIiwiTkVBUl9QT1AiLCJEcHMiKQ0KYGBgDQoNCmBgYHtyfQ0KIyMgQW5lbW9uZSBuZW1vcm9zYQ0KRHBzLmFuZW0gPC0gbGlzdCgpDQpmb3IgKHIgaW4gdW5pcXVlKHJlZ2lvbi5hbmVtKSkgew0KICBEcHMuYW5lbVtbcl1dIDwtIERwcyhhZi5hbmVtX0RlU2lsdmFfYWxsW3JlZ2lvbi5hbmVtPT1yLC0xXSkNCn0NCkRwc0wuYW5lbTwtbGFwcGx5KERwcy5hbmVtLGZ1bmN0aW9uKHgpIG1hdHJpeENvbnZlcnQoYXMubWF0cml4KHgpKSkNCkRwc0RGLmFuZW0gPC0gRHBzTC5hbmVtW1sxXV0NCmZvcihyIGluIDI6bGVuZ3RoKERwc0wuYW5lbSkpIERwc0RGLmFuZW08LXJiaW5kKERwc0RGLmFuZW0sIERwc0wuYW5lbVtbcl1dKSANCmNvbG5hbWVzKERwc0RGLmFuZW0pPC1jKCJJTl9QT1AiLCJORUFSX1BPUCIsIkRwcyIpDQpgYGANCmBgYHtyfQ0KIyMgRGF0YSBTdG9yYWdlDQpQYWlyRGlmZl9hbGwucG11bDwtZGF0YS5mcmFtZShJTl9QT1A9R3N0REYucG11bCRJTl9QT1AsTkVBUl9QT1A9R3N0REYucG11bCRORUFSX1BPUCwNCiAgICAgICAgICAgICAgICAgICAgICAgICAgR3N0PUdzdERGLnBtdWwkR3N0LERwcz1EcHNERi5wbXVsJERwcykNCg0KDQpQYWlyRGlmZl9hbGwub2FjZTwtZGF0YS5mcmFtZShJTl9QT1A9R3N0REYub2FjZSRJTl9QT1AsTkVBUl9QT1A9R3N0REYub2FjZSRORUFSX1BPUCwNCiAgICAgICAgICAgICAgICAgICAgICAgICAgR3N0PUdzdERGLm9hY2UkR3N0LERwcz1EcHNERi5vYWNlJERwcykNCg0KDQpQYWlyRGlmZl9hbGwuYW5lbTwtZGF0YS5mcmFtZShJTl9QT1A9R3N0REYuYW5lbSRJTl9QT1AsTkVBUl9QT1A9R3N0REYuYW5lbSRORUFSX1BPUCwNCiAgICAgICAgICAgICAgICAgICAgICAgICAgR3N0PUdzdERGLmFuZW0kR3N0LERwcz1EcHNERi5hbmVtJERwcykNCg0KDQpzYXZlKGZpbGU9IlBhaXJEaWZmX2FsbF9wbXVsLlJEYXRhIixsaXN0PSJQYWlyRGlmZl9hbGwucG11bCIpDQpzYXZlKGZpbGU9IlBhaXJEaWZmX2FsbF9vYWNlLlJEYXRhIixsaXN0PSJQYWlyRGlmZl9hbGwub2FjZSIpDQpzYXZlKGZpbGU9IlBhaXJEaWZmX2FsbF9hbmVtLlJEYXRhIixsaXN0PSJQYWlyRGlmZl9hbGwuYW5lbSIpDQpgYGANCg0K
