# Supplementary material for: Limited effects of population age on the genetic structure of spatially isolated forest herb populations in temperate Europe
Source: Ecol Evol. 2024 Feb 26;14(2):e10971. doi: 10.1002/ece3.10971 (PMC10897356; doi:10.1002/ece3.10971)
Supplement: Supplementary file 1 — Appendix S1. [file ECE3-14-e10971-s001.zip › 04_Population_Graph.nb.html]

04\_Population\_Graph


Code 

- Show All Code
- Hide All Code
- Download Rmd

# 04\_Population\_Graph

in this part, i calculated conditional genetic distance (cGD) [Dyer
2017]. Based on that, i calculated population graph based
characteristics both in node level: centrality measure of each node; and
also Diff\_Gen\_GEO: the differentiation between geographic distance and
genetic distance


```
library(adegenet)
library(gstudio)
library(popgraph)
library(igraph)
library(poppr)
library(polysat)
library(RUtils)
library(reshape2)
library(ggplot2)
library(dplyr)
library(stringr)


source("L:/05_Data analysis/boxcox.r")
source("L:/05_Data analysis/CU_WP1/functions/edge_contortion_corected.r")
source("L:/05_Data analysis/CU_WP1/functions/harmonic_centrality_CU.r")
source("L:/05_Data analysis/CU_WP1/functions/list2dist_corrected.r")
source("P:/PB2-Projects/Landgen/05_Data analysis/Functions for population genetics.r")
source("L:/05_Data analysis/CU_WP1/functions/popgraph_CU.r")
```


Conditional Genetic Distance.


```
## in order to make three species comparable, we also calculated the genetic distance for Polygonatum and Oxalis using meandistance matrix (calculated euclidean distance). For this function, a ganambig object is needed, thus both diploid species were treated same as Anemone, transfer into a genambig object. 
## Polygonatum multiflorum
load("polcc_all.RData")
Be.pol<-stratsub(polcc_all,"Region","Be")
Est.pol<-stratsub(polcc_all,"Region","Est")
FrN.pol<-stratsub(polcc_all,"Region","FrN")
GeE.pol <- stratsub(polcc_all, "Region", "GeE")
GeW.pol<-stratsub (polcc_all, "Region","GeW")
SwS.pol<-stratsub (polcc_all,"Region","SwS")
Be.pol_gm<-as.genambig(Be.pol)
Est.pol_gm<-as.genambig(Est.pol)
FrN.pol_gm<-as.genambig(FrN.pol)
GeE.pol_gm<-as.genambig(GeE.pol)
GeW.pol_gm<-as.genambig(GeW.pol)
SwS.pol_gm<-as.genambig(SwS.pol)
Ploidies(Be.pol_gm)<-2
Ploidies(Est.pol_gm)<-2
Ploidies(FrN.pol_gm)<-2
Ploidies(GeE.pol_gm)<-2
Ploidies(GeW.pol_gm)<-2
Ploidies(SwS.pol_gm)<-2

groups_Be.pol<-Be.pol@pop
groups_Est.pol<-Est.pol@pop
groups_FrN.pol<-FrN.pol@pop
groups_GeE.pol<-GeE.pol@pop
groups_GeW.pol<-GeW.pol@pop
groups_SwS.pol<-SwS.pol@pop

##Be##
dm_Be.pol<-meandistance.matrix(Be.pol_gm,loci=Loci(Be.pol_gm),distmetric = Euc.dist)
gBe_pol<-popgraph_CU(dm_Be.pol,groups=groups_Be.pol)
##Est##
dm_Est.pol<-meandistance.matrix(Est.pol_gm,loci=Loci(Est.pol_gm),distmetric = Euc.dist)
gEst_pol<-popgraph_CU(dm_Est.pol,groups=groups_Est.pol)
##FrN##
dm_FrN.pol<-meandistance.matrix(FrN.pol_gm,loci=Loci(FrN.pol_gm),distmetric = Euc.dist)
gFrN_pol<-popgraph_CU(dm_FrN.pol,groups=groups_FrN.pol)
###GeE##
dm_GeE.pol<-meandistance.matrix(GeE.pol_gm,loci=Loci(GeE.pol_gm),distmetric = Euc.dist)
gGeE_pol<-popgraph_CU(dm_GeE.pol,groups=groups_GeE.pol)
###GeW###
dm_GeW.pol<-meandistance.matrix(GeW.pol_gm,loci=Loci(GeW.pol_gm),distmetric = Euc.dist)
gGeW_pol<-popgraph_CU(dm_GeW.pol,groups=groups_GeW.pol)
####SwS##
dm_SwS.pol<-meandistance.matrix(SwS.pol_gm,loci=Loci(SwS.pol_gm),distmetric = Euc.dist)
gSwS_pol<-popgraph_CU(dm_SwS.pol,groups=groups_SwS.pol)
```


```
```r
a<-as.data.frame(harmonic_centrality_CU(gBe_ane,normalized=T))
colnames(a)<-\closeness\
b<-as.data.frame(harmonic_centrality_CU(gEst_ane,normalized=T))
colnames(b)<-\closeness\
c<-as.data.frame(harmonic_centrality_CU(gFrN_ane,normalized = T))
colnames(c)<-\closeness\
d<-as.data.frame(harmonic_centrality_CU(gGeE_ane,normalized = T))
colnames(d)<-\closeness\
e<-as.data.frame(harmonic_centrality_CU(gGeW_ane,normalized = T))
colnames(e)<-\closeness\
f<-as.data.frame(harmonic_centrality_CU(gSwS_ane,normalized = T))
colnames(f)<-\closeness\
ane_closeness<-rbind(a,b,c,d,e,f)


a<-as.data.frame(harmonic_centrality_CU(gEst_oxa,normalized = T))
colnames(a)<-\closeness\
b<-as.data.frame(harmonic_centrality_CU(gGeE_oxa,normalized=T))
colnames(b)<-\closeness\
c<-as.data.frame(harmonic_centrality_CU(gGeW_oxa,normalized=T))
colnames(c)<-\closeness\
d<-as.data.frame(harmonic_centrality_CU(gSwS_oxa,normalized=T))
colnames(d)<-\closeness\
oxa_closeness<-rbind(a,b,c,d)

a<-as.data.frame(harmonic_centrality_CU(gBe_pol,normalized=T))
colnames(a)<-\closeness\
b<-as.data.frame(harmonic_centrality_CU(gEst_pol,normalized=T)) 
colnames(b)<-\closeness\
c<-as.data.frame(harmonic_centrality_CU(gFrN_pol,normalized = T))
colnames(c)<-\closeness\
d<-as.data.frame(harmonic_centrality_CU(gGeE_pol,normalized=T))
colnames(d)<-\closeness\
e<-as.data.frame(harmonic_centrality_CU(gGeW_pol,normalized=T))
colnames(e)<-\closeness\
f<-as.data.frame(harmonic_centrality_CU(gSwS_pol,normalized = T))
colnames(f)<-\closeness\
pol_closeness<-rbind(a,b,c,d,e,f)

closeness<-rbind(ane_closeness,oxa_closeness,pol_closeness)
closeness$population<-rownames(closeness)
Node<-merge(closeness, PopAge,by=\population\)
Node<-Node%>%
  rowwise()%>%
  mutate(Age_abs=2020-Age)
save(file=\Node.RData\,list=\Node\)
```

```
<!-- rnb-source-end -->

<!-- rnb-chunk-end -->


<!-- rnb-text-begin -->


<!-- rnb-text-end -->


<!-- rnb-chunk-begin -->


<!-- rnb-source-begin eyJkYXRhIjoiYGBgclxuIyNBbmVtb25lIG5lbW9yb3NhXG5sb2FkKFwiYW5lY2NfYWxsLlJEYXRhXCIpXG5cbkJlLmFuZTwtc3RyYXRzdWIoYW5lY2NfYWxsLFwiUmVnaW9uXCIsXCJCZVwiKVxuRXN0LmFuZTwtc3RyYXRzdWIoYW5lY2NfYWxsLFwiUmVnaW9uXCIsXCJFc3RcIilcbkZyTi5hbmU8LXN0cmF0c3ViKGFuZWNjX2FsbCxcIlJlZ2lvblwiLFwiRnJOXCIpXG5HZUUuYW5lIDwtIHN0cmF0c3ViKGFuZWNjX2FsbCwgXCJSZWdpb25cIiwgXCJHZUVcIilcbkdlVy5hbmU8LXN0cmF0c3ViIChhbmVjY19hbGwsIFwiUmVnaW9uXCIsXCJHZVdcIilcblN3Uy5hbmU8LXN0cmF0c3ViIChhbmVjY19hbGwsXCJSZWdpb25cIixcIlN3U1wiKVxuXG5CZS5hbmVfZ208LWFzLmdlbmFtYmlnKEJlLmFuZSlcbkVzdC5hbmVfZ208LWFzLmdlbmFtYmlnKEVzdC5hbmUpXG5Gck4uYW5lX2dtPC1hcy5nZW5hbWJpZyhGck4uYW5lKVxuR2VFLmFuZV9nbTwtYXMuZ2VuYW1iaWcoR2VFLmFuZSlcbkdlVy5hbmVfZ208LWFzLmdlbmFtYmlnKEdlVy5hbmUpXG5Td1MuYW5lX2dtPC1hcy5nZW5hbWJpZyhTd1MuYW5lKVxuXG5QbG9pZGllcyhCZS5hbmVfZ20pPC00XG5QbG9pZGllcyhFc3QuYW5lX2dtKTwtNFxuUGxvaWRpZXMoRnJOLmFuZV9nbSk8LTRcblBsb2lkaWVzKEdlRS5hbmVfZ20pPC00XG5QbG9pZGllcyhHZVcuYW5lX2dtKTwtNFxuUGxvaWRpZXMoU3dTLmFuZV9nbSk8LTRcblxuZ3JvdXBzX0JlLmFuZTwtQmUuYW5lQHBvcFxuZ3JvdXBzX0VzdC5hbmU8LUVzdC5hbmVAcG9wXG5ncm91cHNfRnJOLmFuZTwtRnJOLmFuZUBwb3Bcbmdyb3Vwc19HZUUuYW5lPC1HZUUuYW5lQHBvcFxuZ3JvdXBzX0dlVy5hbmU8LUdlVy5hbmVAcG9wXG5ncm91cHNfU3dTLmFuZTwtU3dTLmFuZUBwb3BcbiMjIGhlcmUgYXJlIGFscmVhZHkgcHJlY2FsY3VsYXRlZCBhbGxlbGUgZnJlcXVlbnkgYWJqZWN0IGZvciBlYWNoIGxhbmRzY2FwZSB3aW5kb3cuXG5sb2FkKFwiYWYuYW5lbV9EZVNpbHZhX0JlX2FsbC5SRGF0YVwiKVxubG9hZChcImFmLmFuZW1fRGVTaWx2YV9Fc3RfYWxsLlJEYXRhXCIpXG5sb2FkKFwiYWYuYW5lbV9EZVNpbHZhX0ZyTl9hbGwuUkRhdGFcIilcbmxvYWQoXCJhZi5hbmVtX0RlU2lsdmFfR2VFX2FsbC5SRGF0YVwiKVxubG9hZChcImFmLmFuZW1fRGVTaWx2YV9HZVdfYWxsLlJEYXRhXCIpXG5sb2FkKFwiYWYuYW5lbV9EZVNpbHZhX1N3U19hbGwuUkRhdGFcIilcblxuIyNCZSMjXG5kbV9CZS5hbmU8LW1lYW5kaXN0YW5jZS5tYXRyaXgyKEJlLmFuZV9nbSxsb2NpPUxvY2koQmUuYW5lX2dtKSxmcmVxPWFmLmFuZW1fRGVTaWx2YV9CZV9hbGwsZGlzdG1ldHJpYyA9IEV1Yy5kaXN0KVxuZ0JlX2FuZTwtcG9wZ3JhcGhfQ1UoZG1fQmUuYW5lLGdyb3Vwcz1ncm91cHNfQmUuYW5lKVxuIyNFc3QjI1xuZG1fRXN0LmFuZTwtbWVhbmRpc3RhbmNlLm1hdHJpeDIoRXN0LmFuZV9nbSxsb2NpPUxvY2koRXN0LmFuZV9nbSksZnJlcT1hZi5hbmVtX0RlU2lsdmFfRXN0X2FsbCxkaXN0bWV0cmljID0gRXVjLmRpc3QpXG5nRXN0X2FuZTwtcG9wZ3JhcGhfQ1UoZG1fRXN0LmFuZSxncm91cHM9Z3JvdXBzX0VzdC5hbmUpXG4jI0ZyTiMjXG5kbV9Gck4uYW5lPC1tZWFuZGlzdGFuY2UubWF0cml4MihGck4uYW5lX2dtLGxvY2k9TG9jaShGck4uYW5lX2dtKSxmcmVxPWFmLmFuZW1fRGVTaWx2YV9Gck5fYWxsLGRpc3RtZXRyaWMgPSBFdWMuZGlzdClcbmdGck5fYW5lPC1wb3BncmFwaF9DVShkbV9Gck4uYW5lLGdyb3Vwcz1ncm91cHNfRnJOLmFuZSlcbiMjI0dlRSMjXG5kbV9HZUUuYW5lPC1tZWFuZGlzdGFuY2UubWF0cml4MihHZUUuYW5lX2dtLGxvY2k9TG9jaShHZUUuYW5lX2dtKSxmcmVxPWFmLmFuZW1fRGVTaWx2YV9HZUVfYWxsLGRpc3RtZXRyaWMgPSBFdWMuZGlzdClcbmdHZUVfYW5lPC1wb3BncmFwaF9DVShkbV9HZUUuYW5lLGdyb3Vwcz1ncm91cHNfR2VFLmFuZSlcbiMjI0dlVyMjI1xuZG1fR2VXLmFuZTwtbWVhbmRpc3RhbmNlLm1hdHJpeDIoR2VXLmFuZV9nbSxsb2NpPUxvY2koR2VXLmFuZV9nbSksZnJlcT1hZi5hbmVtX0RlU2lsdmFfR2VXX2FsbCxkaXN0bWV0cmljID0gRXVjLmRpc3QpXG5nR2VXX2FuZTwtcG9wZ3JhcGhfQ1UoZG1fR2VXLmFuZSxncm91cHM9Z3JvdXBzX0dlVy5hbmUpXG4jIyMjU3dTIyNcbmRtX1N3Uy5hbmU8LW1lYW5kaXN0YW5jZS5tYXRyaXgyKFN3Uy5hbmVfZ20sbG9jaT1Mb2NpKFN3Uy5hbmVfZ20pLGZyZXE9YWYuYW5lbV9EZVNpbHZhX1N3U19hbGwsZGlzdG1ldHJpYyA9IEV1Yy5kaXN0KVxuZ1N3U19hbmU8LXBvcGdyYXBoX0NVKGRtX1N3Uy5hbmUsZ3JvdXBzPWdyb3Vwc19Td1MuYW5lKVxuXG5cbkJlX2FuZTwtY2JpbmQoYXMuZGF0YS5mcmFtZShnZXQuZWRnZWxpc3QoZ0JlX2FuZSkpLGFzLmRhdGEuZnJhbWUoZ2V0LmVkZ2UuYXR0cmlidXRlKGdCZV9hbmUpKSlcbkVzdF9hbmU8LWNiaW5kKGFzLmRhdGEuZnJhbWUoZ2V0LmVkZ2VsaXN0KGdFc3RfYW5lKSksYXMuZGF0YS5mcmFtZShnZXQuZWRnZS5hdHRyaWJ1dGUoZ0VzdF9hbmUpKSlcbkZyTl9hbmU8LWNiaW5kKGFzLmRhdGEuZnJhbWUoZ2V0LmVkZ2VsaXN0KGdGck5fYW5lKSksYXMuZGF0YS5mcmFtZShnZXQuZWRnZS5hdHRyaWJ1dGUoZ0ZyTl9hbmUpKSlcbkdlRV9hbmU8LWNiaW5kKGFzLmRhdGEuZnJhbWUoZ2V0LmVkZ2VsaXN0KGdHZUVfYW5lKSksYXMuZGF0YS5mcmFtZShnZXQuZWRnZS5hdHRyaWJ1dGUoZ0dlRV9hbmUpKSlcbkdlV19hbmU8LWNiaW5kKGFzLmRhdGEuZnJhbWUoZ2V0LmVkZ2VsaXN0KGdHZVdfYW5lKSksYXMuZGF0YS5mcmFtZShnZXQuZWRnZS5hdHRyaWJ1dGUoZ0dlV19hbmUpKSlcblN3U19hbmU8LWNiaW5kKGFzLmRhdGEuZnJhbWUoZ2V0LmVkZ2VsaXN0KGdTd1NfYW5lKSksYXMuZGF0YS5mcmFtZShnZXQuZWRnZS5hdHRyaWJ1dGUoZ1N3U19hbmUpKSlcblxuRXN0X294YTwtY2JpbmQoYXMuZGF0YS5mcmFtZShnZXQuZWRnZWxpc3QoZ0VzdF9veGEpKSxhcy5kYXRhLmZyYW1lKGdldC5lZGdlLmF0dHJpYnV0ZShnRXN0X294YSkpKVxuR2VFX294YTwtY2JpbmQoYXMuZGF0YS5mcmFtZShnZXQuZWRnZWxpc3QoZ0dlRV9veGEpKSxhcy5kYXRhLmZyYW1lKGdldC5lZGdlLmF0dHJpYnV0ZShnR2VFX294YSkpKVxuR2VXX294YTwtY2JpbmQoYXMuZGF0YS5mcmFtZShnZXQuZWRnZWxpc3QoZ0dlV19veGEpKSxhcy5kYXRhLmZyYW1lKGdldC5lZGdlLmF0dHJpYnV0ZShnR2VXX294YSkpKVxuU3dTX294YTwtY2JpbmQoYXMuZGF0YS5mcmFtZShnZXQuZWRnZWxpc3QoZ1N3U19veGEpKSxhcy5kYXRhLmZyYW1lKGdldC5lZGdlLmF0dHJpYnV0ZShnU3dTX294YSkpKVxuXG5CZV9wb2w8LWNiaW5kKGFzLmRhdGEuZnJhbWUoZ2V0LmVkZ2VsaXN0KGdCZV9wb2wpKSxhcy5kYXRhLmZyYW1lKGdldC5lZGdlLmF0dHJpYnV0ZShnQmVfcG9sKSkpXG5Fc3RfcG9sPC1jYmluZChhcy5kYXRhLmZyYW1lKGdldC5lZGdlbGlzdChnRXN0X3BvbCkpLGFzLmRhdGEuZnJhbWUoZ2V0LmVkZ2UuYXR0cmlidXRlKGdFc3RfcG9sKSkpXG5Gck5fcG9sPC1jYmluZChhcy5kYXRhLmZyYW1lKGdldC5lZGdlbGlzdChnRnJOX3BvbCkpLGFzLmRhdGEuZnJhbWUoZ2V0LmVkZ2UuYXR0cmlidXRlKGdGck5fcG9sKSkpXG5HZUVfcG9sPC1jYmluZChhcy5kYXRhLmZyYW1lKGdldC5lZGdlbGlzdChnR2VFX3BvbCkpLGFzLmRhdGEuZnJhbWUoZ2V0LmVkZ2UuYXR0cmlidXRlKGdHZUVfcG9sKSkpXG5HZVdfcG9sPC1jYmluZChhcy5kYXRhLmZyYW1lKGdldC5lZGdlbGlzdChnR2VXX3BvbCkpLGFzLmRhdGEuZnJhbWUoZ2V0LmVkZ2UuYXR0cmlidXRlKGdHZVdfcG9sKSkpXG5Td1NfcG9sPC1jYmluZChhcy5kYXRhLmZyYW1lKGdldC5lZGdlbGlzdChnU3dTX3BvbCkpLGFzLmRhdGEuZnJhbWUoZ2V0LmVkZ2UuYXR0cmlidXRlKGdTd1NfcG9sKSkpXG5cbkNEPC1yYmluZChCZV9hbmUsRXN0X2FuZSxGck5fYW5lLEdlRV9hbmUsR2VXX2FuZSxTd1NfYW5lLEVzdF9veGEsR2VFX294YSxHZVdfb3hhLFN3U19veGEsQmVfcG9sLEVzdF9wb2wsRnJOX3BvbCxHZUVfcG9sLEdlV19wb2wsU3dTX3BvbClcbmNvbG5hbWVzKENEKTwtYyhcIklOX1BPUFwiLFwiTkVBUl9QT1BcIixcIkNvbi5EaXNcIilcbkNEJFNwZWNpZXM8LWFzLmZhY3Rvcihhcy5jaGFyYWN0ZXIobGFwcGx5KHN0cnNwbGl0KENEJElOX1BPUCxcIl9cIiksZnVuY3Rpb24oeCkgeFsxXSkpKVxuQ0QkTkVBUl9QT1BfSUQ8LWFzLmNoYXJhY3RlcihsYXBwbHkoc3Ryc3BsaXQoQ0QkTkVBUl9QT1AsXCJfXCIpLGZ1bmN0aW9uKHgpIHhbM10pKVxuQ0QkSU5fUE9QX0lEPC1hcy5jaGFyYWN0ZXIobGFwcGx5KHN0cnNwbGl0KENEJElOX1BPUCxcIl9cIiksZnVuY3Rpb24oeCkgeFszXSkpXG5DRDwtQ0QlPiVcbm11dGF0ZShEaXN0SUQ9cGFzdGUoYXMuY2hhcmFjdGVyKElOX1BPUCksTkVBUl9QT1BfSUQsc2VwPVwiX1wiKSlcbnNhdmUoZmlsZT1cIkNELlJEYXRhXCIsbGlzdD1cIkNEXCIpXG5gYGAifQ== -->

```r
##Anemone nemorosa
load("anecc_all.RData")

Be.ane<-stratsub(anecc_all,"Region","Be")
Est.ane<-stratsub(anecc_all,"Region","Est")
FrN.ane<-stratsub(anecc_all,"Region","FrN")
GeE.ane <- stratsub(anecc_all, "Region", "GeE")
GeW.ane<-stratsub (anecc_all, "Region","GeW")
SwS.ane<-stratsub (anecc_all,"Region","SwS")

Be.ane_gm<-as.genambig(Be.ane)
Est.ane_gm<-as.genambig(Est.ane)
FrN.ane_gm<-as.genambig(FrN.ane)
GeE.ane_gm<-as.genambig(GeE.ane)
GeW.ane_gm<-as.genambig(GeW.ane)
SwS.ane_gm<-as.genambig(SwS.ane)

Ploidies(Be.ane_gm)<-4
Ploidies(Est.ane_gm)<-4
Ploidies(FrN.ane_gm)<-4
Ploidies(GeE.ane_gm)<-4
Ploidies(GeW.ane_gm)<-4
Ploidies(SwS.ane_gm)<-4

groups_Be.ane<-Be.ane@pop
groups_Est.ane<-Est.ane@pop
groups_FrN.ane<-FrN.ane@pop
groups_GeE.ane<-GeE.ane@pop
groups_GeW.ane<-GeW.ane@pop
groups_SwS.ane<-SwS.ane@pop
## here are already precalculated allele frequeny abject for each landscape window.
load("af.anem_DeSilva_Be_all.RData")
load("af.anem_DeSilva_Est_all.RData")
load("af.anem_DeSilva_FrN_all.RData")
load("af.anem_DeSilva_GeE_all.RData")
load("af.anem_DeSilva_GeW_all.RData")
load("af.anem_DeSilva_SwS_all.RData")

##Be##
dm_Be.ane<-meandistance.matrix2(Be.ane_gm,loci=Loci(Be.ane_gm),freq=af.anem_DeSilva_Be_all,distmetric = Euc.dist)
gBe_ane<-popgraph_CU(dm_Be.ane,groups=groups_Be.ane)
##Est##
dm_Est.ane<-meandistance.matrix2(Est.ane_gm,loci=Loci(Est.ane_gm),freq=af.anem_DeSilva_Est_all,distmetric = Euc.dist)
gEst_ane<-popgraph_CU(dm_Est.ane,groups=groups_Est.ane)
##FrN##
dm_FrN.ane<-meandistance.matrix2(FrN.ane_gm,loci=Loci(FrN.ane_gm),freq=af.anem_DeSilva_FrN_all,distmetric = Euc.dist)
gFrN_ane<-popgraph_CU(dm_FrN.ane,groups=groups_FrN.ane)
###GeE##
dm_GeE.ane<-meandistance.matrix2(GeE.ane_gm,loci=Loci(GeE.ane_gm),freq=af.anem_DeSilva_GeE_all,distmetric = Euc.dist)
gGeE_ane<-popgraph_CU(dm_GeE.ane,groups=groups_GeE.ane)
###GeW###
dm_GeW.ane<-meandistance.matrix2(GeW.ane_gm,loci=Loci(GeW.ane_gm),freq=af.anem_DeSilva_GeW_all,distmetric = Euc.dist)
gGeW_ane<-popgraph_CU(dm_GeW.ane,groups=groups_GeW.ane)
####SwS##
dm_SwS.ane<-meandistance.matrix2(SwS.ane_gm,loci=Loci(SwS.ane_gm),freq=af.anem_DeSilva_SwS_all,distmetric = Euc.dist)
gSwS_ane<-popgraph_CU(dm_SwS.ane,groups=groups_SwS.ane)


Be_ane<-cbind(as.data.frame(get.edgelist(gBe_ane)),as.data.frame(get.edge.attribute(gBe_ane)))
Est_ane<-cbind(as.data.frame(get.edgelist(gEst_ane)),as.data.frame(get.edge.attribute(gEst_ane)))
FrN_ane<-cbind(as.data.frame(get.edgelist(gFrN_ane)),as.data.frame(get.edge.attribute(gFrN_ane)))
GeE_ane<-cbind(as.data.frame(get.edgelist(gGeE_ane)),as.data.frame(get.edge.attribute(gGeE_ane)))
GeW_ane<-cbind(as.data.frame(get.edgelist(gGeW_ane)),as.data.frame(get.edge.attribute(gGeW_ane)))
SwS_ane<-cbind(as.data.frame(get.edgelist(gSwS_ane)),as.data.frame(get.edge.attribute(gSwS_ane)))

Est_oxa<-cbind(as.data.frame(get.edgelist(gEst_oxa)),as.data.frame(get.edge.attribute(gEst_oxa)))
GeE_oxa<-cbind(as.data.frame(get.edgelist(gGeE_oxa)),as.data.frame(get.edge.attribute(gGeE_oxa)))
GeW_oxa<-cbind(as.data.frame(get.edgelist(gGeW_oxa)),as.data.frame(get.edge.attribute(gGeW_oxa)))
SwS_oxa<-cbind(as.data.frame(get.edgelist(gSwS_oxa)),as.data.frame(get.edge.attribute(gSwS_oxa)))

Be_pol<-cbind(as.data.frame(get.edgelist(gBe_pol)),as.data.frame(get.edge.attribute(gBe_pol)))
Est_pol<-cbind(as.data.frame(get.edgelist(gEst_pol)),as.data.frame(get.edge.attribute(gEst_pol)))
FrN_pol<-cbind(as.data.frame(get.edgelist(gFrN_pol)),as.data.frame(get.edge.attribute(gFrN_pol)))
GeE_pol<-cbind(as.data.frame(get.edgelist(gGeE_pol)),as.data.frame(get.edge.attribute(gGeE_pol)))
GeW_pol<-cbind(as.data.frame(get.edgelist(gGeW_pol)),as.data.frame(get.edge.attribute(gGeW_pol)))
SwS_pol<-cbind(as.data.frame(get.edgelist(gSwS_pol)),as.data.frame(get.edge.attribute(gSwS_pol)))

CD<-rbind(Be_ane,Est_ane,FrN_ane,GeE_ane,GeW_ane,SwS_ane,Est_oxa,GeE_oxa,GeW_oxa,SwS_oxa,Be_pol,Est_pol,FrN_pol,GeE_pol,GeW_pol,SwS_pol)
colnames(CD)<-c("IN_POP","NEAR_POP","Con.Dis")
CD$Species<-as.factor(as.character(lapply(strsplit(CD$IN_POP,"_"),function(x) x[1])))
CD$NEAR_POP_ID<-as.character(lapply(strsplit(CD$NEAR_POP,"_"),function(x) x[3]))
CD$IN_POP_ID<-as.character(lapply(strsplit(CD$IN_POP,"_"),function(x) x[3]))
CD<-CD%>%
mutate(DistID=paste(as.character(IN_POP),NEAR_POP_ID,sep="_"))
save(file="CD.RData",list="CD")
```


DIFF\_GEN\_GEO


```
## create distance matrix based on geographical distance and compare whether conditional genetic distance is proportionally larger or smaller than expected by geographical distance 
library(dendextend)
library(spaa)
library(openxlsx)
geodist<-read.xlsx("Distances_among_surveyed_populations.xlsx",1,rowNames = T)
geodist$X<-paste(geodist$Species,"_",geodist$Region,"_",geodist$IN_POP,"_",geodist$NEAR_POP,sep="")

## before conducting this part, please first go to 05_Preparation_For_Linear_Model.
geodist_all<-merge(geodist,PairDiff_all,by.x="X",by.y="Link")
geodistane<-subset(geodist_all,geodist_all$Species=="Ane")
geodistoxa<-subset(geodist_all,geodist_all$Species=="Oxa")
geodistpol<-subset(geodist_all,geodist_all$Species=="Pol")
## Anemone nemorosa
distane_Be<-subset(geodistane,geodistane$Region=="Be")
distane_Be<-as.matrix(list2dist_CU(distane_Be[,c(7,8,6)]))

distane_Est<-subset(geodistane,geodistane$Region=="Est")
distane_Est<-as.matrix(list2dist_CU(distane_Est[,c(7,8,6)]))

distane_FrN<-subset(geodistane,geodistane$Region=="FrN")
distane_FrN<-as.matrix(list2dist_CU(distane_FrN[,c(7,8,6)]))

distane_GeE<-subset(geodistane,geodistane$Region=="GeE")
distane_GeE<-as.matrix(list2dist_CU(distane_GeE[,c(7,8,6)]))

distane_GeW<-subset(geodistane,geodistane$Region=="GeW")
distane_GeW<-as.matrix(list2dist_CU(distane_GeW[,c(7,8,6)]))

distane_SwS<-subset(geodistane,geodistane$Region=="SwS")
distane_SwS<-as.matrix(list2dist_CU(distane_SwS[,c(7,8,6)]))

gBe_ane <- edge_contortion_CU(gBe_ane, P=distane_Be)
name<-as_ids(E(gBe_ane))
edge_type<-E(gBe_ane)$stretch
Diff_G_P<-E(gBe_ane)$contortion
gBe_ane_edge<-as.data.frame(cbind(name,edge_type,Diff_G_P))

gEst_ane <- edge_contortion_CU(gEst_ane, P=distane_Est)
name<-as_ids(E(gEst_ane))
edge_type<-E(gEst_ane)$stretch
Diff_G_P<-E(gEst_ane)$contortion
gEst_ane_edge<-as.data.frame(cbind(name,edge_type,Diff_G_P))

gFrN_ane <- edge_contortion_CU(gFrN_ane, P=distane_FrN)
name<-as_ids(E(gFrN_ane))
edge_type<-E(gFrN_ane)$stretch
Diff_G_P<-E(gFrN_ane)$contortion
gFrN_ane_edge<-as.data.frame(cbind(name,edge_type,Diff_G_P))

gGeE_ane <- edge_contortion_CU(gGeE_ane, P=distane_GeE)
name<-as_ids(E(gGeE_ane))
edge_type<-E(gGeE_ane)$stretch
Diff_G_P<-E(gGeE_ane)$contortion
gGeE_ane_edge<-as.data.frame(cbind(name,edge_type,Diff_G_P))

gGeW_ane <- edge_contortion_CU(gGeW_ane, P=distane_GeW)
name<-as_ids(E(gGeW_ane))
edge_type<-E(gGeW_ane)$stretch
Diff_G_P<-E(gGeW_ane)$contortion
gGeW_ane_edge<-as.data.frame(cbind(name,edge_type,Diff_G_P))

gSwS_ane <- edge_contortion_CU(gSwS_ane, P=distane_SwS)
name<-as_ids(E(gSwS_ane))
edge_type<-E(gSwS_ane)$stretch
Diff_G_P<-E(gSwS_ane)$contortion
gSwS_ane_edge<-as.data.frame(cbind(name,edge_type,Diff_G_P))

ane_edge<-rbind(gBe_ane_edge,gEst_ane_edge,gFrN_ane_edge,gGeE_ane_edge,gGeW_ane_edge,gSwS_ane_edge)

##Oxalis acetosella
distoxa_Est<-subset(geodistoxa,geodistoxa$Region=="Est")
distoxa_Est<-as.matrix(list2dist_CU(distoxa_Est[,c(7,8,6)]))

distoxa_GeE<-subset(geodistoxa,geodistoxa$Region=="GeE")
distoxa_GeE<-as.matrix(list2dist_CU(distoxa_GeE[,c(7,8,6)]))

distoxa_GeW<-subset(geodistoxa,geodistoxa$Region=="GeW")
distoxa_GeW<-as.matrix(list2dist_CU(distoxa_GeW[,c(7,8,6)]))

distoxa_SwS<-subset(geodistoxa,geodistoxa$Region=="SwS")
distoxa_SwS<-as.matrix(list2dist_CU(distoxa_SwS[,c(7,8,6)]))

gEst_oxa <- edge_contortion_CU(gEst_oxa, P=distoxa_Est)
name<-as_ids(E(gEst_oxa))
edge_type<-E(gEst_oxa)$stretch
Diff_G_P<-E(gEst_oxa)$contortion
gEst_oxa_edge<-as.data.frame(cbind(name,edge_type,Diff_G_P))

gGeE_oxa <- edge_contortion_CU(gGeE_oxa, P=distoxa_GeE)
name<-as_ids(E(gGeE_oxa))
edge_type<-E(gGeE_oxa)$stretch
Diff_G_P<-E(gGeE_oxa)$contortion
gGeE_oxa_edge<-as.data.frame(cbind(name,edge_type,Diff_G_P))

gGeW_oxa <- edge_contortion_CU(gGeW_oxa, P=distoxa_GeW)
name<-as_ids(E(gGeW_oxa))
edge_type<-E(gGeW_oxa)$stretch
Diff_G_P<-E(gGeW_oxa)$contortion
gGeW_oxa_edge<-as.data.frame(cbind(name,edge_type,Diff_G_P))

gSwS_oxa <- edge_contortion_CU(gSwS_oxa, P=distoxa_SwS)
name<-as_ids(E(gSwS_oxa))
edge_type<-E(gSwS_oxa)$stretch
Diff_G_P<-E(gSwS_oxa)$contortion
gSwS_oxa_edge<-as.data.frame(cbind(name,edge_type,Diff_G_P))

oxa_edge<-rbind(gEst_oxa_edge,gGeE_oxa_edge,gGeW_oxa_edge,gSwS_oxa_edge)

## Polygonatum multiflorum
distpol_Be<-subset(geodistpol,geodistpol$Region=="Be")
distpol_Be<-as.matrix(list2dist_CU(distpol_Be[,c(7,8,6)]))

distpol_Est<-subset(geodistpol,geodistpol$Region=="Est")
distpol_Est<-as.matrix(list2dist_CU(distpol_Est[,c(7,8,6)]))

distpol_FrN<-subset(geodistpol,geodistpol$Region=="FrN")
distpol_FrN<-as.matrix(list2dist_CU(distpol_FrN[,c(7,8,6)]))

distpol_GeE<-subset(geodistpol,geodistpol$Region=="GeE")
distpol_GeE<-as.matrix(list2dist_CU(distpol_GeE[,c(7,8,6)]))

distpol_GeW<-subset(geodistpol,geodistpol$Region=="GeW")
distpol_GeW<-as.matrix(list2dist_CU(distpol_GeW[,c(7,8,6)]))

distpol_SwS<-subset(geodistpol,geodistpol$Region=="SwS")
distpol_SwS<-as.matrix(list2dist_CU(distpol_SwS[,c(7,8,6)]))

gBe_pol <- edge_contortion_CU(gBe_pol, P=distpol_Be)
name<-as_ids(E(gBe_pol))
edge_type<-E(gBe_pol)$stretch
Diff_G_P<-E(gBe_pol)$contortion
gBe_pol_edge<-as.data.frame(cbind(name,edge_type,Diff_G_P))

gEst_pol <- edge_contortion_CU(gEst_pol, P=distpol_Est)
name<-as_ids(E(gEst_pol))
edge_type<-E(gEst_pol)$stretch
Diff_G_P<-E(gEst_pol)$contortion
gEst_pol_edge<-as.data.frame(cbind(name,edge_type,Diff_G_P))

gFrN_pol <- edge_contortion_CU(gFrN_pol, P=distpol_FrN)
name<-as_ids(E(gFrN_pol))
edge_type<-E(gFrN_pol)$stretch
Diff_G_P<-E(gFrN_pol)$contortion
gFrN_pol_edge<-as.data.frame(cbind(name,edge_type,Diff_G_P))

gGeE_pol <- edge_contortion_CU(gGeE_pol, P=distpol_GeE)
name<-as_ids(E(gGeE_pol))
edge_type<-E(gGeE_pol)$stretch
Diff_G_P<-E(gGeE_pol)$contortion
gGeE_pol_edge<-as.data.frame(cbind(name,edge_type,Diff_G_P))

gGeW_pol <- edge_contortion_CU(gGeW_pol, P=distpol_GeW)
name<-as_ids(E(gGeW_pol))
edge_type<-E(gGeW_pol)$stretch
Diff_G_P<-E(gGeW_pol)$contortion
gGeW_pol_edge<-as.data.frame(cbind(name,edge_type,Diff_G_P))

gSwS_pol <- edge_contortion_CU(gSwS_pol, P=distpol_SwS)
name<-as_ids(E(gSwS_pol))
edge_type<-E(gSwS_pol)$stretch
Diff_G_P<-E(gSwS_pol)$contortion
gSwS_pol_edge<-as.data.frame(cbind(name,edge_type,Diff_G_P))

pol_edge<-rbind(gBe_pol_edge,gEst_pol_edge,gFrN_pol_edge,gGeE_pol_edge,gGeW_pol_edge,gSwS_pol_edge)

Link<-rbind(ane_edge,oxa_edge,pol_edge)

Link<-Link %>%
  separate(name, into=c("node_1","node_2"),sep="\\|")%>%
  mutate(species=substr(node_1,1,3)) %>%
  mutate(LW=sapply(strsplit(node_1,"_"),"[",2))

Link<-merge(Link,popagediffDF,by.x=c("node_1","node_2"),by.y=c("IN_POP","NEAR_POP"))
save(file="Link.RData",list="Link")
```


Centrality measure


```
a<-as.data.frame(harmonic_centrality_CU(gBe_ane,normalized=T))
colnames(a)<-"closeness"
b<-as.data.frame(harmonic_centrality_CU(gEst_ane,normalized=T))
colnames(b)<-"closeness"
c<-as.data.frame(harmonic_centrality_CU(gFrN_ane,normalized = T))
colnames(c)<-"closeness"
d<-as.data.frame(harmonic_centrality_CU(gGeE_ane,normalized = T))
colnames(d)<-"closeness"
e<-as.data.frame(harmonic_centrality_CU(gGeW_ane,normalized = T))
colnames(e)<-"closeness"
f<-as.data.frame(harmonic_centrality_CU(gSwS_ane,normalized = T))
colnames(f)<-"closeness"
ane_closeness<-rbind(a,b,c,d,e,f)


a<-as.data.frame(harmonic_centrality_CU(gEst_oxa,normalized = T))
colnames(a)<-"closeness"
b<-as.data.frame(harmonic_centrality_CU(gGeE_oxa,normalized=T))
colnames(b)<-"closeness"
c<-as.data.frame(harmonic_centrality_CU(gGeW_oxa,normalized=T))
colnames(c)<-"closeness"
d<-as.data.frame(harmonic_centrality_CU(gSwS_oxa,normalized=T))
colnames(d)<-"closeness"
oxa_closeness<-rbind(a,b,c,d)

a<-as.data.frame(harmonic_centrality_CU(gBe_pol,normalized=T))
colnames(a)<-"closeness"
b<-as.data.frame(harmonic_centrality_CU(gEst_pol,normalized=T)) 
colnames(b)<-"closeness"
c<-as.data.frame(harmonic_centrality_CU(gFrN_pol,normalized = T))
colnames(c)<-"closeness"
d<-as.data.frame(harmonic_centrality_CU(gGeE_pol,normalized=T))
colnames(d)<-"closeness"
e<-as.data.frame(harmonic_centrality_CU(gGeW_pol,normalized=T))
colnames(e)<-"closeness"
f<-as.data.frame(harmonic_centrality_CU(gSwS_pol,normalized = T))
colnames(f)<-"closeness"
pol_closeness<-rbind(a,b,c,d,e,f)

closeness<-rbind(ane_closeness,oxa_closeness,pol_closeness)
closeness$population<-rownames(closeness)
Node<-merge(closeness, PopAge,by="population")
Node<-Node%>%
  rowwise()%>%
  mutate(Age_abs=2020-Age)
save(file="Node.RData",list="Node")
```


LS0tDQp0aXRsZTogIjA0X1BvcHVsYXRpb25fR3JhcGgiDQpvdXRwdXQ6IGh0bWxfbm90ZWJvb2sNCi0tLQ0KaW4gdGhpcyBwYXJ0LCBpIGNhbGN1bGF0ZWQgY29uZGl0aW9uYWwgZ2VuZXRpYyBkaXN0YW5jZSAoY0dEKSBbRHllciAyMDE3XS4gQmFzZWQgb24gdGhhdCwgaSBjYWxjdWxhdGVkIHBvcHVsYXRpb24gZ3JhcGggYmFzZWQgY2hhcmFjdGVyaXN0aWNzIGJvdGggaW4gbm9kZSBsZXZlbDogY2VudHJhbGl0eSBtZWFzdXJlIG9mIGVhY2ggbm9kZTsgYW5kIGFsc28gRGlmZl9HZW5fR0VPOiB0aGUgZGlmZmVyZW50aWF0aW9uIGJldHdlZW4gZ2VvZ3JhcGhpYyBkaXN0YW5jZSBhbmQgZ2VuZXRpYyBkaXN0YW5jZQ0KDQpgYGB7cn0NCmxpYnJhcnkoYWRlZ2VuZXQpDQpsaWJyYXJ5KGdzdHVkaW8pDQpsaWJyYXJ5KHBvcGdyYXBoKQ0KbGlicmFyeShpZ3JhcGgpDQpsaWJyYXJ5KHBvcHByKQ0KbGlicmFyeShwb2x5c2F0KQ0KbGlicmFyeShSVXRpbHMpDQpsaWJyYXJ5KHJlc2hhcGUyKQ0KbGlicmFyeShnZ3Bsb3QyKQ0KbGlicmFyeShkcGx5cikNCmxpYnJhcnkoc3RyaW5ncikNCg0KDQpzb3VyY2UoIkw6LzA1X0RhdGEgYW5hbHlzaXMvYm94Y294LnIiKQ0Kc291cmNlKCJMOi8wNV9EYXRhIGFuYWx5c2lzL0NVX1dQMS9mdW5jdGlvbnMvZWRnZV9jb250b3J0aW9uX2NvcmVjdGVkLnIiKQ0Kc291cmNlKCJMOi8wNV9EYXRhIGFuYWx5c2lzL0NVX1dQMS9mdW5jdGlvbnMvaGFybW9uaWNfY2VudHJhbGl0eV9DVS5yIikNCnNvdXJjZSgiTDovMDVfRGF0YSBhbmFseXNpcy9DVV9XUDEvZnVuY3Rpb25zL2xpc3QyZGlzdF9jb3JyZWN0ZWQuciIpDQpzb3VyY2UoIlA6L1BCMi1Qcm9qZWN0cy9MYW5kZ2VuLzA1X0RhdGEgYW5hbHlzaXMvRnVuY3Rpb25zIGZvciBwb3B1bGF0aW9uIGdlbmV0aWNzLnIiKQ0Kc291cmNlKCJMOi8wNV9EYXRhIGFuYWx5c2lzL0NVX1dQMS9mdW5jdGlvbnMvcG9wZ3JhcGhfQ1UuciIpDQpgYGANCkNvbmRpdGlvbmFsIEdlbmV0aWMgRGlzdGFuY2UuIA0KYGBge3J9DQojIyBpbiBvcmRlciB0byBtYWtlIHRocmVlIHNwZWNpZXMgY29tcGFyYWJsZSwgd2UgYWxzbyBjYWxjdWxhdGVkIHRoZSBnZW5ldGljIGRpc3RhbmNlIGZvciBQb2x5Z29uYXR1bSBhbmQgT3hhbGlzIHVzaW5nIG1lYW5kaXN0YW5jZSBtYXRyaXggKGNhbGN1bGF0ZWQgZXVjbGlkZWFuIGRpc3RhbmNlKS4gRm9yIHRoaXMgZnVuY3Rpb24sIGEgZ2FuYW1iaWcgb2JqZWN0IGlzIG5lZWRlZCwgdGh1cyBib3RoIGRpcGxvaWQgc3BlY2llcyB3ZXJlIHRyZWF0ZWQgc2FtZSBhcyBBbmVtb25lLCB0cmFuc2ZlciBpbnRvIGEgZ2VuYW1iaWcgb2JqZWN0LiANCiMjIFBvbHlnb25hdHVtIG11bHRpZmxvcnVtDQpsb2FkKCJwb2xjY19hbGwuUkRhdGEiKQ0KQmUucG9sPC1zdHJhdHN1Yihwb2xjY19hbGwsIlJlZ2lvbiIsIkJlIikNCkVzdC5wb2w8LXN0cmF0c3ViKHBvbGNjX2FsbCwiUmVnaW9uIiwiRXN0IikNCkZyTi5wb2w8LXN0cmF0c3ViKHBvbGNjX2FsbCwiUmVnaW9uIiwiRnJOIikNCkdlRS5wb2wgPC0gc3RyYXRzdWIocG9sY2NfYWxsLCAiUmVnaW9uIiwgIkdlRSIpDQpHZVcucG9sPC1zdHJhdHN1YiAocG9sY2NfYWxsLCAiUmVnaW9uIiwiR2VXIikNClN3Uy5wb2w8LXN0cmF0c3ViIChwb2xjY19hbGwsIlJlZ2lvbiIsIlN3UyIpDQpCZS5wb2xfZ208LWFzLmdlbmFtYmlnKEJlLnBvbCkNCkVzdC5wb2xfZ208LWFzLmdlbmFtYmlnKEVzdC5wb2wpDQpGck4ucG9sX2dtPC1hcy5nZW5hbWJpZyhGck4ucG9sKQ0KR2VFLnBvbF9nbTwtYXMuZ2VuYW1iaWcoR2VFLnBvbCkNCkdlVy5wb2xfZ208LWFzLmdlbmFtYmlnKEdlVy5wb2wpDQpTd1MucG9sX2dtPC1hcy5nZW5hbWJpZyhTd1MucG9sKQ0KUGxvaWRpZXMoQmUucG9sX2dtKTwtMg0KUGxvaWRpZXMoRXN0LnBvbF9nbSk8LTINClBsb2lkaWVzKEZyTi5wb2xfZ20pPC0yDQpQbG9pZGllcyhHZUUucG9sX2dtKTwtMg0KUGxvaWRpZXMoR2VXLnBvbF9nbSk8LTINClBsb2lkaWVzKFN3Uy5wb2xfZ20pPC0yDQoNCmdyb3Vwc19CZS5wb2w8LUJlLnBvbEBwb3ANCmdyb3Vwc19Fc3QucG9sPC1Fc3QucG9sQHBvcA0KZ3JvdXBzX0ZyTi5wb2w8LUZyTi5wb2xAcG9wDQpncm91cHNfR2VFLnBvbDwtR2VFLnBvbEBwb3ANCmdyb3Vwc19HZVcucG9sPC1HZVcucG9sQHBvcA0KZ3JvdXBzX1N3Uy5wb2w8LVN3Uy5wb2xAcG9wDQoNCiMjQmUjIw0KZG1fQmUucG9sPC1tZWFuZGlzdGFuY2UubWF0cml4KEJlLnBvbF9nbSxsb2NpPUxvY2koQmUucG9sX2dtKSxkaXN0bWV0cmljID0gRXVjLmRpc3QpDQpnQmVfcG9sPC1wb3BncmFwaF9DVShkbV9CZS5wb2wsZ3JvdXBzPWdyb3Vwc19CZS5wb2wpDQojI0VzdCMjDQpkbV9Fc3QucG9sPC1tZWFuZGlzdGFuY2UubWF0cml4KEVzdC5wb2xfZ20sbG9jaT1Mb2NpKEVzdC5wb2xfZ20pLGRpc3RtZXRyaWMgPSBFdWMuZGlzdCkNCmdFc3RfcG9sPC1wb3BncmFwaF9DVShkbV9Fc3QucG9sLGdyb3Vwcz1ncm91cHNfRXN0LnBvbCkNCiMjRnJOIyMNCmRtX0ZyTi5wb2w8LW1lYW5kaXN0YW5jZS5tYXRyaXgoRnJOLnBvbF9nbSxsb2NpPUxvY2koRnJOLnBvbF9nbSksZGlzdG1ldHJpYyA9IEV1Yy5kaXN0KQ0KZ0ZyTl9wb2w8LXBvcGdyYXBoX0NVKGRtX0ZyTi5wb2wsZ3JvdXBzPWdyb3Vwc19Gck4ucG9sKQ0KIyMjR2VFIyMNCmRtX0dlRS5wb2w8LW1lYW5kaXN0YW5jZS5tYXRyaXgoR2VFLnBvbF9nbSxsb2NpPUxvY2koR2VFLnBvbF9nbSksZGlzdG1ldHJpYyA9IEV1Yy5kaXN0KQ0KZ0dlRV9wb2w8LXBvcGdyYXBoX0NVKGRtX0dlRS5wb2wsZ3JvdXBzPWdyb3Vwc19HZUUucG9sKQ0KIyMjR2VXIyMjDQpkbV9HZVcucG9sPC1tZWFuZGlzdGFuY2UubWF0cml4KEdlVy5wb2xfZ20sbG9jaT1Mb2NpKEdlVy5wb2xfZ20pLGRpc3RtZXRyaWMgPSBFdWMuZGlzdCkNCmdHZVdfcG9sPC1wb3BncmFwaF9DVShkbV9HZVcucG9sLGdyb3Vwcz1ncm91cHNfR2VXLnBvbCkNCiMjIyNTd1MjIw0KZG1fU3dTLnBvbDwtbWVhbmRpc3RhbmNlLm1hdHJpeChTd1MucG9sX2dtLGxvY2k9TG9jaShTd1MucG9sX2dtKSxkaXN0bWV0cmljID0gRXVjLmRpc3QpDQpnU3dTX3BvbDwtcG9wZ3JhcGhfQ1UoZG1fU3dTLnBvbCxncm91cHM9Z3JvdXBzX1N3Uy5wb2wpDQpgYGANCmBgYHtyfQ0KIyNPeGFsaXMgYWNldG9zZWxsYQ0KbG9hZCgib3hhY2NfYWxsLlJEYXRhIikNCkVzdC5veGE8LXN0cmF0c3ViKG94YWNjX2FsbCwiUmVnaW9uIiwiRXN0IikNCkdlRS5veGEgPC1zdHJhdHN1YihveGFjY19hbGwsICJSZWdpb24iLCAiR2VFIikNCkdlVy5veGE8LXN0cmF0c3ViIChveGFjY19hbGwsICJSZWdpb24iLCJHZVciKQ0KU3dTLm94YTwtc3RyYXRzdWIgKG94YWNjX2FsbCwiUmVnaW9uIiwiU3dTIikNCkVzdC5veGFfZ208LWFzLmdlbmFtYmlnKEVzdC5veGEpDQpHZUUub3hhX2dtPC1hcy5nZW5hbWJpZyhHZUUub3hhKQ0KR2VXLm94YV9nbTwtYXMuZ2VuYW1iaWcoR2VXLm94YSkNClN3Uy5veGFfZ208LWFzLmdlbmFtYmlnKFN3Uy5veGEpDQoNClBsb2lkaWVzKEVzdC5veGFfZ20pPC0yDQpQbG9pZGllcyhHZUUub3hhX2dtKTwtMg0KUGxvaWRpZXMoR2VXLm94YV9nbSk8LTINClBsb2lkaWVzKFN3Uy5veGFfZ20pPC0yDQoNCmdyb3Vwc19Fc3Qub3hhPC1Fc3Qub3hhQHBvcA0KZ3JvdXBzX0dlRS5veGE8LUdlRS5veGFAcG9wDQpncm91cHNfR2VXLm94YTwtR2VXLm94YUBwb3ANCmdyb3Vwc19Td1Mub3hhPC1Td1Mub3hhQHBvcA0KDQojI0VzdCMjDQpkbV9Fc3Qub3hhPC1tZWFuZGlzdGFuY2UubWF0cml4KEVzdC5veGFfZ20sbG9jaT1Mb2NpKEVzdC5veGFfZ20pLGRpc3RtZXRyaWMgPSBFdWMuZGlzdCkNCmdFc3Rfb3hhPC1wb3BncmFwaF9DVShkbV9Fc3Qub3hhLGdyb3Vwcz1ncm91cHNfRXN0Lm94YSkNCiMjI0dlRSMjDQpkbV9HZUUub3hhPC1tZWFuZGlzdGFuY2UubWF0cml4KEdlRS5veGFfZ20sbG9jaT1Mb2NpKEdlRS5veGFfZ20pLGRpc3RtZXRyaWMgPSBFdWMuZGlzdCkNCmdHZUVfb3hhPC1wb3BncmFwaF9DVShkbV9HZUUub3hhLGdyb3Vwcz1ncm91cHNfR2VFLm94YSkNCiMjI0dlVyMjIw0KZG1fR2VXLm94YTwtbWVhbmRpc3RhbmNlLm1hdHJpeChHZVcub3hhX2dtLGxvY2k9TG9jaShHZVcub3hhX2dtKSxkaXN0bWV0cmljID0gRXVjLmRpc3QpDQpnR2VXX294YTwtcG9wZ3JhcGhfQ1UoZG1fR2VXLm94YSxncm91cHM9Z3JvdXBzX0dlVy5veGEpDQojIyMjU3dTIyMNCmRtX1N3Uy5veGE8LW1lYW5kaXN0YW5jZS5tYXRyaXgoU3dTLm94YV9nbSxsb2NpPUxvY2koU3dTLm94YV9nbSksZGlzdG1ldHJpYyA9IEV1Yy5kaXN0KQ0KZ1N3U19veGE8LXBvcGdyYXBoX0NVKGRtX1N3Uy5veGEsZ3JvdXBzPWdyb3Vwc19Td1Mub3hhKQ0KDQpgYGANCg0KYGBge3J9DQojI0FuZW1vbmUgbmVtb3Jvc2ENCmxvYWQoImFuZWNjX2FsbC5SRGF0YSIpDQoNCkJlLmFuZTwtc3RyYXRzdWIoYW5lY2NfYWxsLCJSZWdpb24iLCJCZSIpDQpFc3QuYW5lPC1zdHJhdHN1YihhbmVjY19hbGwsIlJlZ2lvbiIsIkVzdCIpDQpGck4uYW5lPC1zdHJhdHN1YihhbmVjY19hbGwsIlJlZ2lvbiIsIkZyTiIpDQpHZUUuYW5lIDwtIHN0cmF0c3ViKGFuZWNjX2FsbCwgIlJlZ2lvbiIsICJHZUUiKQ0KR2VXLmFuZTwtc3RyYXRzdWIgKGFuZWNjX2FsbCwgIlJlZ2lvbiIsIkdlVyIpDQpTd1MuYW5lPC1zdHJhdHN1YiAoYW5lY2NfYWxsLCJSZWdpb24iLCJTd1MiKQ0KDQpCZS5hbmVfZ208LWFzLmdlbmFtYmlnKEJlLmFuZSkNCkVzdC5hbmVfZ208LWFzLmdlbmFtYmlnKEVzdC5hbmUpDQpGck4uYW5lX2dtPC1hcy5nZW5hbWJpZyhGck4uYW5lKQ0KR2VFLmFuZV9nbTwtYXMuZ2VuYW1iaWcoR2VFLmFuZSkNCkdlVy5hbmVfZ208LWFzLmdlbmFtYmlnKEdlVy5hbmUpDQpTd1MuYW5lX2dtPC1hcy5nZW5hbWJpZyhTd1MuYW5lKQ0KDQpQbG9pZGllcyhCZS5hbmVfZ20pPC00DQpQbG9pZGllcyhFc3QuYW5lX2dtKTwtNA0KUGxvaWRpZXMoRnJOLmFuZV9nbSk8LTQNClBsb2lkaWVzKEdlRS5hbmVfZ20pPC00DQpQbG9pZGllcyhHZVcuYW5lX2dtKTwtNA0KUGxvaWRpZXMoU3dTLmFuZV9nbSk8LTQNCg0KZ3JvdXBzX0JlLmFuZTwtQmUuYW5lQHBvcA0KZ3JvdXBzX0VzdC5hbmU8LUVzdC5hbmVAcG9wDQpncm91cHNfRnJOLmFuZTwtRnJOLmFuZUBwb3ANCmdyb3Vwc19HZUUuYW5lPC1HZUUuYW5lQHBvcA0KZ3JvdXBzX0dlVy5hbmU8LUdlVy5hbmVAcG9wDQpncm91cHNfU3dTLmFuZTwtU3dTLmFuZUBwb3ANCiMjIGhlcmUgYXJlIGFscmVhZHkgcHJlY2FsY3VsYXRlZCBhbGxlbGUgZnJlcXVlbnkgYWJqZWN0IGZvciBlYWNoIGxhbmRzY2FwZSB3aW5kb3cuDQpsb2FkKCJhZi5hbmVtX0RlU2lsdmFfQmVfYWxsLlJEYXRhIikNCmxvYWQoImFmLmFuZW1fRGVTaWx2YV9Fc3RfYWxsLlJEYXRhIikNCmxvYWQoImFmLmFuZW1fRGVTaWx2YV9Gck5fYWxsLlJEYXRhIikNCmxvYWQoImFmLmFuZW1fRGVTaWx2YV9HZUVfYWxsLlJEYXRhIikNCmxvYWQoImFmLmFuZW1fRGVTaWx2YV9HZVdfYWxsLlJEYXRhIikNCmxvYWQoImFmLmFuZW1fRGVTaWx2YV9Td1NfYWxsLlJEYXRhIikNCg0KIyNCZSMjDQpkbV9CZS5hbmU8LW1lYW5kaXN0YW5jZS5tYXRyaXgyKEJlLmFuZV9nbSxsb2NpPUxvY2koQmUuYW5lX2dtKSxmcmVxPWFmLmFuZW1fRGVTaWx2YV9CZV9hbGwsZGlzdG1ldHJpYyA9IEV1Yy5kaXN0KQ0KZ0JlX2FuZTwtcG9wZ3JhcGhfQ1UoZG1fQmUuYW5lLGdyb3Vwcz1ncm91cHNfQmUuYW5lKQ0KIyNFc3QjIw0KZG1fRXN0LmFuZTwtbWVhbmRpc3RhbmNlLm1hdHJpeDIoRXN0LmFuZV9nbSxsb2NpPUxvY2koRXN0LmFuZV9nbSksZnJlcT1hZi5hbmVtX0RlU2lsdmFfRXN0X2FsbCxkaXN0bWV0cmljID0gRXVjLmRpc3QpDQpnRXN0X2FuZTwtcG9wZ3JhcGhfQ1UoZG1fRXN0LmFuZSxncm91cHM9Z3JvdXBzX0VzdC5hbmUpDQojI0ZyTiMjDQpkbV9Gck4uYW5lPC1tZWFuZGlzdGFuY2UubWF0cml4MihGck4uYW5lX2dtLGxvY2k9TG9jaShGck4uYW5lX2dtKSxmcmVxPWFmLmFuZW1fRGVTaWx2YV9Gck5fYWxsLGRpc3RtZXRyaWMgPSBFdWMuZGlzdCkNCmdGck5fYW5lPC1wb3BncmFwaF9DVShkbV9Gck4uYW5lLGdyb3Vwcz1ncm91cHNfRnJOLmFuZSkNCiMjI0dlRSMjDQpkbV9HZUUuYW5lPC1tZWFuZGlzdGFuY2UubWF0cml4MihHZUUuYW5lX2dtLGxvY2k9TG9jaShHZUUuYW5lX2dtKSxmcmVxPWFmLmFuZW1fRGVTaWx2YV9HZUVfYWxsLGRpc3RtZXRyaWMgPSBFdWMuZGlzdCkNCmdHZUVfYW5lPC1wb3BncmFwaF9DVShkbV9HZUUuYW5lLGdyb3Vwcz1ncm91cHNfR2VFLmFuZSkNCiMjI0dlVyMjIw0KZG1fR2VXLmFuZTwtbWVhbmRpc3RhbmNlLm1hdHJpeDIoR2VXLmFuZV9nbSxsb2NpPUxvY2koR2VXLmFuZV9nbSksZnJlcT1hZi5hbmVtX0RlU2lsdmFfR2VXX2FsbCxkaXN0bWV0cmljID0gRXVjLmRpc3QpDQpnR2VXX2FuZTwtcG9wZ3JhcGhfQ1UoZG1fR2VXLmFuZSxncm91cHM9Z3JvdXBzX0dlVy5hbmUpDQojIyMjU3dTIyMNCmRtX1N3Uy5hbmU8LW1lYW5kaXN0YW5jZS5tYXRyaXgyKFN3Uy5hbmVfZ20sbG9jaT1Mb2NpKFN3Uy5hbmVfZ20pLGZyZXE9YWYuYW5lbV9EZVNpbHZhX1N3U19hbGwsZGlzdG1ldHJpYyA9IEV1Yy5kaXN0KQ0KZ1N3U19hbmU8LXBvcGdyYXBoX0NVKGRtX1N3Uy5hbmUsZ3JvdXBzPWdyb3Vwc19Td1MuYW5lKQ0KDQoNCkJlX2FuZTwtY2JpbmQoYXMuZGF0YS5mcmFtZShnZXQuZWRnZWxpc3QoZ0JlX2FuZSkpLGFzLmRhdGEuZnJhbWUoZ2V0LmVkZ2UuYXR0cmlidXRlKGdCZV9hbmUpKSkNCkVzdF9hbmU8LWNiaW5kKGFzLmRhdGEuZnJhbWUoZ2V0LmVkZ2VsaXN0KGdFc3RfYW5lKSksYXMuZGF0YS5mcmFtZShnZXQuZWRnZS5hdHRyaWJ1dGUoZ0VzdF9hbmUpKSkNCkZyTl9hbmU8LWNiaW5kKGFzLmRhdGEuZnJhbWUoZ2V0LmVkZ2VsaXN0KGdGck5fYW5lKSksYXMuZGF0YS5mcmFtZShnZXQuZWRnZS5hdHRyaWJ1dGUoZ0ZyTl9hbmUpKSkNCkdlRV9hbmU8LWNiaW5kKGFzLmRhdGEuZnJhbWUoZ2V0LmVkZ2VsaXN0KGdHZUVfYW5lKSksYXMuZGF0YS5mcmFtZShnZXQuZWRnZS5hdHRyaWJ1dGUoZ0dlRV9hbmUpKSkNCkdlV19hbmU8LWNiaW5kKGFzLmRhdGEuZnJhbWUoZ2V0LmVkZ2VsaXN0KGdHZVdfYW5lKSksYXMuZGF0YS5mcmFtZShnZXQuZWRnZS5hdHRyaWJ1dGUoZ0dlV19hbmUpKSkNClN3U19hbmU8LWNiaW5kKGFzLmRhdGEuZnJhbWUoZ2V0LmVkZ2VsaXN0KGdTd1NfYW5lKSksYXMuZGF0YS5mcmFtZShnZXQuZWRnZS5hdHRyaWJ1dGUoZ1N3U19hbmUpKSkNCg0KRXN0X294YTwtY2JpbmQoYXMuZGF0YS5mcmFtZShnZXQuZWRnZWxpc3QoZ0VzdF9veGEpKSxhcy5kYXRhLmZyYW1lKGdldC5lZGdlLmF0dHJpYnV0ZShnRXN0X294YSkpKQ0KR2VFX294YTwtY2JpbmQoYXMuZGF0YS5mcmFtZShnZXQuZWRnZWxpc3QoZ0dlRV9veGEpKSxhcy5kYXRhLmZyYW1lKGdldC5lZGdlLmF0dHJpYnV0ZShnR2VFX294YSkpKQ0KR2VXX294YTwtY2JpbmQoYXMuZGF0YS5mcmFtZShnZXQuZWRnZWxpc3QoZ0dlV19veGEpKSxhcy5kYXRhLmZyYW1lKGdldC5lZGdlLmF0dHJpYnV0ZShnR2VXX294YSkpKQ0KU3dTX294YTwtY2JpbmQoYXMuZGF0YS5mcmFtZShnZXQuZWRnZWxpc3QoZ1N3U19veGEpKSxhcy5kYXRhLmZyYW1lKGdldC5lZGdlLmF0dHJpYnV0ZShnU3dTX294YSkpKQ0KDQpCZV9wb2w8LWNiaW5kKGFzLmRhdGEuZnJhbWUoZ2V0LmVkZ2VsaXN0KGdCZV9wb2wpKSxhcy5kYXRhLmZyYW1lKGdldC5lZGdlLmF0dHJpYnV0ZShnQmVfcG9sKSkpDQpFc3RfcG9sPC1jYmluZChhcy5kYXRhLmZyYW1lKGdldC5lZGdlbGlzdChnRXN0X3BvbCkpLGFzLmRhdGEuZnJhbWUoZ2V0LmVkZ2UuYXR0cmlidXRlKGdFc3RfcG9sKSkpDQpGck5fcG9sPC1jYmluZChhcy5kYXRhLmZyYW1lKGdldC5lZGdlbGlzdChnRnJOX3BvbCkpLGFzLmRhdGEuZnJhbWUoZ2V0LmVkZ2UuYXR0cmlidXRlKGdGck5fcG9sKSkpDQpHZUVfcG9sPC1jYmluZChhcy5kYXRhLmZyYW1lKGdldC5lZGdlbGlzdChnR2VFX3BvbCkpLGFzLmRhdGEuZnJhbWUoZ2V0LmVkZ2UuYXR0cmlidXRlKGdHZUVfcG9sKSkpDQpHZVdfcG9sPC1jYmluZChhcy5kYXRhLmZyYW1lKGdldC5lZGdlbGlzdChnR2VXX3BvbCkpLGFzLmRhdGEuZnJhbWUoZ2V0LmVkZ2UuYXR0cmlidXRlKGdHZVdfcG9sKSkpDQpTd1NfcG9sPC1jYmluZChhcy5kYXRhLmZyYW1lKGdldC5lZGdlbGlzdChnU3dTX3BvbCkpLGFzLmRhdGEuZnJhbWUoZ2V0LmVkZ2UuYXR0cmlidXRlKGdTd1NfcG9sKSkpDQoNCkNEPC1yYmluZChCZV9hbmUsRXN0X2FuZSxGck5fYW5lLEdlRV9hbmUsR2VXX2FuZSxTd1NfYW5lLEVzdF9veGEsR2VFX294YSxHZVdfb3hhLFN3U19veGEsQmVfcG9sLEVzdF9wb2wsRnJOX3BvbCxHZUVfcG9sLEdlV19wb2wsU3dTX3BvbCkNCmNvbG5hbWVzKENEKTwtYygiSU5fUE9QIiwiTkVBUl9QT1AiLCJDb24uRGlzIikNCkNEJFNwZWNpZXM8LWFzLmZhY3Rvcihhcy5jaGFyYWN0ZXIobGFwcGx5KHN0cnNwbGl0KENEJElOX1BPUCwiXyIpLGZ1bmN0aW9uKHgpIHhbMV0pKSkNCkNEJE5FQVJfUE9QX0lEPC1hcy5jaGFyYWN0ZXIobGFwcGx5KHN0cnNwbGl0KENEJE5FQVJfUE9QLCJfIiksZnVuY3Rpb24oeCkgeFszXSkpDQpDRCRJTl9QT1BfSUQ8LWFzLmNoYXJhY3RlcihsYXBwbHkoc3Ryc3BsaXQoQ0QkSU5fUE9QLCJfIiksZnVuY3Rpb24oeCkgeFszXSkpDQpDRDwtQ0QlPiUNCm11dGF0ZShEaXN0SUQ9cGFzdGUoYXMuY2hhcmFjdGVyKElOX1BPUCksTkVBUl9QT1BfSUQsc2VwPSJfIikpDQpzYXZlKGZpbGU9IkNELlJEYXRhIixsaXN0PSJDRCIpDQpgYGANCkRJRkZfR0VOX0dFTw0KYGBge3J9DQojIyBjcmVhdGUgZGlzdGFuY2UgbWF0cml4IGJhc2VkIG9uIGdlb2dyYXBoaWNhbCBkaXN0YW5jZSBhbmQgY29tcGFyZSB3aGV0aGVyIGNvbmRpdGlvbmFsIGdlbmV0aWMgZGlzdGFuY2UgaXMgcHJvcG9ydGlvbmFsbHkgbGFyZ2VyIG9yIHNtYWxsZXIgdGhhbiBleHBlY3RlZCBieSBnZW9ncmFwaGljYWwgZGlzdGFuY2UgDQpsaWJyYXJ5KGRlbmRleHRlbmQpDQpsaWJyYXJ5KHNwYWEpDQpsaWJyYXJ5KG9wZW54bHN4KQ0KZ2VvZGlzdDwtcmVhZC54bHN4KCJEaXN0YW5jZXNfYW1vbmdfc3VydmV5ZWRfcG9wdWxhdGlvbnMueGxzeCIsMSxyb3dOYW1lcyA9IFQpDQpnZW9kaXN0JFg8LXBhc3RlKGdlb2Rpc3QkU3BlY2llcywiXyIsZ2VvZGlzdCRSZWdpb24sIl8iLGdlb2Rpc3QkSU5fUE9QLCJfIixnZW9kaXN0JE5FQVJfUE9QLHNlcD0iIikNCg0KIyMgYmVmb3JlIGNvbmR1Y3RpbmcgdGhpcyBwYXJ0LCBwbGVhc2UgZmlyc3QgZ28gdG8gMDVfUHJlcGFyYXRpb25fRm9yX0xpbmVhcl9Nb2RlbC4NCmdlb2Rpc3RfYWxsPC1tZXJnZShnZW9kaXN0LFBhaXJEaWZmX2FsbCxieS54PSJYIixieS55PSJMaW5rIikNCmdlb2Rpc3RhbmU8LXN1YnNldChnZW9kaXN0X2FsbCxnZW9kaXN0X2FsbCRTcGVjaWVzPT0iQW5lIikNCmdlb2Rpc3RveGE8LXN1YnNldChnZW9kaXN0X2FsbCxnZW9kaXN0X2FsbCRTcGVjaWVzPT0iT3hhIikNCmdlb2Rpc3Rwb2w8LXN1YnNldChnZW9kaXN0X2FsbCxnZW9kaXN0X2FsbCRTcGVjaWVzPT0iUG9sIikNCiMjIEFuZW1vbmUgbmVtb3Jvc2ENCmRpc3RhbmVfQmU8LXN1YnNldChnZW9kaXN0YW5lLGdlb2Rpc3RhbmUkUmVnaW9uPT0iQmUiKQ0KZGlzdGFuZV9CZTwtYXMubWF0cml4KGxpc3QyZGlzdF9DVShkaXN0YW5lX0JlWyxjKDcsOCw2KV0pKQ0KDQpkaXN0YW5lX0VzdDwtc3Vic2V0KGdlb2Rpc3RhbmUsZ2VvZGlzdGFuZSRSZWdpb249PSJFc3QiKQ0KZGlzdGFuZV9Fc3Q8LWFzLm1hdHJpeChsaXN0MmRpc3RfQ1UoZGlzdGFuZV9Fc3RbLGMoNyw4LDYpXSkpDQoNCmRpc3RhbmVfRnJOPC1zdWJzZXQoZ2VvZGlzdGFuZSxnZW9kaXN0YW5lJFJlZ2lvbj09IkZyTiIpDQpkaXN0YW5lX0ZyTjwtYXMubWF0cml4KGxpc3QyZGlzdF9DVShkaXN0YW5lX0ZyTlssYyg3LDgsNildKSkNCg0KZGlzdGFuZV9HZUU8LXN1YnNldChnZW9kaXN0YW5lLGdlb2Rpc3RhbmUkUmVnaW9uPT0iR2VFIikNCmRpc3RhbmVfR2VFPC1hcy5tYXRyaXgobGlzdDJkaXN0X0NVKGRpc3RhbmVfR2VFWyxjKDcsOCw2KV0pKQ0KDQpkaXN0YW5lX0dlVzwtc3Vic2V0KGdlb2Rpc3RhbmUsZ2VvZGlzdGFuZSRSZWdpb249PSJHZVciKQ0KZGlzdGFuZV9HZVc8LWFzLm1hdHJpeChsaXN0MmRpc3RfQ1UoZGlzdGFuZV9HZVdbLGMoNyw4LDYpXSkpDQoNCmRpc3RhbmVfU3dTPC1zdWJzZXQoZ2VvZGlzdGFuZSxnZW9kaXN0YW5lJFJlZ2lvbj09IlN3UyIpDQpkaXN0YW5lX1N3UzwtYXMubWF0cml4KGxpc3QyZGlzdF9DVShkaXN0YW5lX1N3U1ssYyg3LDgsNildKSkNCg0KZ0JlX2FuZSA8LSBlZGdlX2NvbnRvcnRpb25fQ1UoZ0JlX2FuZSwgUD1kaXN0YW5lX0JlKQ0KbmFtZTwtYXNfaWRzKEUoZ0JlX2FuZSkpDQplZGdlX3R5cGU8LUUoZ0JlX2FuZSkkc3RyZXRjaA0KRGlmZl9HX1A8LUUoZ0JlX2FuZSkkY29udG9ydGlvbg0KZ0JlX2FuZV9lZGdlPC1hcy5kYXRhLmZyYW1lKGNiaW5kKG5hbWUsZWRnZV90eXBlLERpZmZfR19QKSkNCg0KZ0VzdF9hbmUgPC0gZWRnZV9jb250b3J0aW9uX0NVKGdFc3RfYW5lLCBQPWRpc3RhbmVfRXN0KQ0KbmFtZTwtYXNfaWRzKEUoZ0VzdF9hbmUpKQ0KZWRnZV90eXBlPC1FKGdFc3RfYW5lKSRzdHJldGNoDQpEaWZmX0dfUDwtRShnRXN0X2FuZSkkY29udG9ydGlvbg0KZ0VzdF9hbmVfZWRnZTwtYXMuZGF0YS5mcmFtZShjYmluZChuYW1lLGVkZ2VfdHlwZSxEaWZmX0dfUCkpDQoNCmdGck5fYW5lIDwtIGVkZ2VfY29udG9ydGlvbl9DVShnRnJOX2FuZSwgUD1kaXN0YW5lX0ZyTikNCm5hbWU8LWFzX2lkcyhFKGdGck5fYW5lKSkNCmVkZ2VfdHlwZTwtRShnRnJOX2FuZSkkc3RyZXRjaA0KRGlmZl9HX1A8LUUoZ0ZyTl9hbmUpJGNvbnRvcnRpb24NCmdGck5fYW5lX2VkZ2U8LWFzLmRhdGEuZnJhbWUoY2JpbmQobmFtZSxlZGdlX3R5cGUsRGlmZl9HX1ApKQ0KDQpnR2VFX2FuZSA8LSBlZGdlX2NvbnRvcnRpb25fQ1UoZ0dlRV9hbmUsIFA9ZGlzdGFuZV9HZUUpDQpuYW1lPC1hc19pZHMoRShnR2VFX2FuZSkpDQplZGdlX3R5cGU8LUUoZ0dlRV9hbmUpJHN0cmV0Y2gNCkRpZmZfR19QPC1FKGdHZUVfYW5lKSRjb250b3J0aW9uDQpnR2VFX2FuZV9lZGdlPC1hcy5kYXRhLmZyYW1lKGNiaW5kKG5hbWUsZWRnZV90eXBlLERpZmZfR19QKSkNCg0KZ0dlV19hbmUgPC0gZWRnZV9jb250b3J0aW9uX0NVKGdHZVdfYW5lLCBQPWRpc3RhbmVfR2VXKQ0KbmFtZTwtYXNfaWRzKEUoZ0dlV19hbmUpKQ0KZWRnZV90eXBlPC1FKGdHZVdfYW5lKSRzdHJldGNoDQpEaWZmX0dfUDwtRShnR2VXX2FuZSkkY29udG9ydGlvbg0KZ0dlV19hbmVfZWRnZTwtYXMuZGF0YS5mcmFtZShjYmluZChuYW1lLGVkZ2VfdHlwZSxEaWZmX0dfUCkpDQoNCmdTd1NfYW5lIDwtIGVkZ2VfY29udG9ydGlvbl9DVShnU3dTX2FuZSwgUD1kaXN0YW5lX1N3UykNCm5hbWU8LWFzX2lkcyhFKGdTd1NfYW5lKSkNCmVkZ2VfdHlwZTwtRShnU3dTX2FuZSkkc3RyZXRjaA0KRGlmZl9HX1A8LUUoZ1N3U19hbmUpJGNvbnRvcnRpb24NCmdTd1NfYW5lX2VkZ2U8LWFzLmRhdGEuZnJhbWUoY2JpbmQobmFtZSxlZGdlX3R5cGUsRGlmZl9HX1ApKQ0KDQphbmVfZWRnZTwtcmJpbmQoZ0JlX2FuZV9lZGdlLGdFc3RfYW5lX2VkZ2UsZ0ZyTl9hbmVfZWRnZSxnR2VFX2FuZV9lZGdlLGdHZVdfYW5lX2VkZ2UsZ1N3U19hbmVfZWRnZSkNCg0KIyNPeGFsaXMgYWNldG9zZWxsYQ0KZGlzdG94YV9Fc3Q8LXN1YnNldChnZW9kaXN0b3hhLGdlb2Rpc3RveGEkUmVnaW9uPT0iRXN0IikNCmRpc3RveGFfRXN0PC1hcy5tYXRyaXgobGlzdDJkaXN0X0NVKGRpc3RveGFfRXN0WyxjKDcsOCw2KV0pKQ0KDQpkaXN0b3hhX0dlRTwtc3Vic2V0KGdlb2Rpc3RveGEsZ2VvZGlzdG94YSRSZWdpb249PSJHZUUiKQ0KZGlzdG94YV9HZUU8LWFzLm1hdHJpeChsaXN0MmRpc3RfQ1UoZGlzdG94YV9HZUVbLGMoNyw4LDYpXSkpDQoNCmRpc3RveGFfR2VXPC1zdWJzZXQoZ2VvZGlzdG94YSxnZW9kaXN0b3hhJFJlZ2lvbj09IkdlVyIpDQpkaXN0b3hhX0dlVzwtYXMubWF0cml4KGxpc3QyZGlzdF9DVShkaXN0b3hhX0dlV1ssYyg3LDgsNildKSkNCg0KZGlzdG94YV9Td1M8LXN1YnNldChnZW9kaXN0b3hhLGdlb2Rpc3RveGEkUmVnaW9uPT0iU3dTIikNCmRpc3RveGFfU3dTPC1hcy5tYXRyaXgobGlzdDJkaXN0X0NVKGRpc3RveGFfU3dTWyxjKDcsOCw2KV0pKQ0KDQpnRXN0X294YSA8LSBlZGdlX2NvbnRvcnRpb25fQ1UoZ0VzdF9veGEsIFA9ZGlzdG94YV9Fc3QpDQpuYW1lPC1hc19pZHMoRShnRXN0X294YSkpDQplZGdlX3R5cGU8LUUoZ0VzdF9veGEpJHN0cmV0Y2gNCkRpZmZfR19QPC1FKGdFc3Rfb3hhKSRjb250b3J0aW9uDQpnRXN0X294YV9lZGdlPC1hcy5kYXRhLmZyYW1lKGNiaW5kKG5hbWUsZWRnZV90eXBlLERpZmZfR19QKSkNCg0KZ0dlRV9veGEgPC0gZWRnZV9jb250b3J0aW9uX0NVKGdHZUVfb3hhLCBQPWRpc3RveGFfR2VFKQ0KbmFtZTwtYXNfaWRzKEUoZ0dlRV9veGEpKQ0KZWRnZV90eXBlPC1FKGdHZUVfb3hhKSRzdHJldGNoDQpEaWZmX0dfUDwtRShnR2VFX294YSkkY29udG9ydGlvbg0KZ0dlRV9veGFfZWRnZTwtYXMuZGF0YS5mcmFtZShjYmluZChuYW1lLGVkZ2VfdHlwZSxEaWZmX0dfUCkpDQoNCmdHZVdfb3hhIDwtIGVkZ2VfY29udG9ydGlvbl9DVShnR2VXX294YSwgUD1kaXN0b3hhX0dlVykNCm5hbWU8LWFzX2lkcyhFKGdHZVdfb3hhKSkNCmVkZ2VfdHlwZTwtRShnR2VXX294YSkkc3RyZXRjaA0KRGlmZl9HX1A8LUUoZ0dlV19veGEpJGNvbnRvcnRpb24NCmdHZVdfb3hhX2VkZ2U8LWFzLmRhdGEuZnJhbWUoY2JpbmQobmFtZSxlZGdlX3R5cGUsRGlmZl9HX1ApKQ0KDQpnU3dTX294YSA8LSBlZGdlX2NvbnRvcnRpb25fQ1UoZ1N3U19veGEsIFA9ZGlzdG94YV9Td1MpDQpuYW1lPC1hc19pZHMoRShnU3dTX294YSkpDQplZGdlX3R5cGU8LUUoZ1N3U19veGEpJHN0cmV0Y2gNCkRpZmZfR19QPC1FKGdTd1Nfb3hhKSRjb250b3J0aW9uDQpnU3dTX294YV9lZGdlPC1hcy5kYXRhLmZyYW1lKGNiaW5kKG5hbWUsZWRnZV90eXBlLERpZmZfR19QKSkNCg0Kb3hhX2VkZ2U8LXJiaW5kKGdFc3Rfb3hhX2VkZ2UsZ0dlRV9veGFfZWRnZSxnR2VXX294YV9lZGdlLGdTd1Nfb3hhX2VkZ2UpDQoNCiMjIFBvbHlnb25hdHVtIG11bHRpZmxvcnVtDQpkaXN0cG9sX0JlPC1zdWJzZXQoZ2VvZGlzdHBvbCxnZW9kaXN0cG9sJFJlZ2lvbj09IkJlIikNCmRpc3Rwb2xfQmU8LWFzLm1hdHJpeChsaXN0MmRpc3RfQ1UoZGlzdHBvbF9CZVssYyg3LDgsNildKSkNCg0KZGlzdHBvbF9Fc3Q8LXN1YnNldChnZW9kaXN0cG9sLGdlb2Rpc3Rwb2wkUmVnaW9uPT0iRXN0IikNCmRpc3Rwb2xfRXN0PC1hcy5tYXRyaXgobGlzdDJkaXN0X0NVKGRpc3Rwb2xfRXN0WyxjKDcsOCw2KV0pKQ0KDQpkaXN0cG9sX0ZyTjwtc3Vic2V0KGdlb2Rpc3Rwb2wsZ2VvZGlzdHBvbCRSZWdpb249PSJGck4iKQ0KZGlzdHBvbF9Gck48LWFzLm1hdHJpeChsaXN0MmRpc3RfQ1UoZGlzdHBvbF9Gck5bLGMoNyw4LDYpXSkpDQoNCmRpc3Rwb2xfR2VFPC1zdWJzZXQoZ2VvZGlzdHBvbCxnZW9kaXN0cG9sJFJlZ2lvbj09IkdlRSIpDQpkaXN0cG9sX0dlRTwtYXMubWF0cml4KGxpc3QyZGlzdF9DVShkaXN0cG9sX0dlRVssYyg3LDgsNildKSkNCg0KZGlzdHBvbF9HZVc8LXN1YnNldChnZW9kaXN0cG9sLGdlb2Rpc3Rwb2wkUmVnaW9uPT0iR2VXIikNCmRpc3Rwb2xfR2VXPC1hcy5tYXRyaXgobGlzdDJkaXN0X0NVKGRpc3Rwb2xfR2VXWyxjKDcsOCw2KV0pKQ0KDQpkaXN0cG9sX1N3Uzwtc3Vic2V0KGdlb2Rpc3Rwb2wsZ2VvZGlzdHBvbCRSZWdpb249PSJTd1MiKQ0KZGlzdHBvbF9Td1M8LWFzLm1hdHJpeChsaXN0MmRpc3RfQ1UoZGlzdHBvbF9Td1NbLGMoNyw4LDYpXSkpDQoNCmdCZV9wb2wgPC0gZWRnZV9jb250b3J0aW9uX0NVKGdCZV9wb2wsIFA9ZGlzdHBvbF9CZSkNCm5hbWU8LWFzX2lkcyhFKGdCZV9wb2wpKQ0KZWRnZV90eXBlPC1FKGdCZV9wb2wpJHN0cmV0Y2gNCkRpZmZfR19QPC1FKGdCZV9wb2wpJGNvbnRvcnRpb24NCmdCZV9wb2xfZWRnZTwtYXMuZGF0YS5mcmFtZShjYmluZChuYW1lLGVkZ2VfdHlwZSxEaWZmX0dfUCkpDQoNCmdFc3RfcG9sIDwtIGVkZ2VfY29udG9ydGlvbl9DVShnRXN0X3BvbCwgUD1kaXN0cG9sX0VzdCkNCm5hbWU8LWFzX2lkcyhFKGdFc3RfcG9sKSkNCmVkZ2VfdHlwZTwtRShnRXN0X3BvbCkkc3RyZXRjaA0KRGlmZl9HX1A8LUUoZ0VzdF9wb2wpJGNvbnRvcnRpb24NCmdFc3RfcG9sX2VkZ2U8LWFzLmRhdGEuZnJhbWUoY2JpbmQobmFtZSxlZGdlX3R5cGUsRGlmZl9HX1ApKQ0KDQpnRnJOX3BvbCA8LSBlZGdlX2NvbnRvcnRpb25fQ1UoZ0ZyTl9wb2wsIFA9ZGlzdHBvbF9Gck4pDQpuYW1lPC1hc19pZHMoRShnRnJOX3BvbCkpDQplZGdlX3R5cGU8LUUoZ0ZyTl9wb2wpJHN0cmV0Y2gNCkRpZmZfR19QPC1FKGdGck5fcG9sKSRjb250b3J0aW9uDQpnRnJOX3BvbF9lZGdlPC1hcy5kYXRhLmZyYW1lKGNiaW5kKG5hbWUsZWRnZV90eXBlLERpZmZfR19QKSkNCg0KZ0dlRV9wb2wgPC0gZWRnZV9jb250b3J0aW9uX0NVKGdHZUVfcG9sLCBQPWRpc3Rwb2xfR2VFKQ0KbmFtZTwtYXNfaWRzKEUoZ0dlRV9wb2wpKQ0KZWRnZV90eXBlPC1FKGdHZUVfcG9sKSRzdHJldGNoDQpEaWZmX0dfUDwtRShnR2VFX3BvbCkkY29udG9ydGlvbg0KZ0dlRV9wb2xfZWRnZTwtYXMuZGF0YS5mcmFtZShjYmluZChuYW1lLGVkZ2VfdHlwZSxEaWZmX0dfUCkpDQoNCmdHZVdfcG9sIDwtIGVkZ2VfY29udG9ydGlvbl9DVShnR2VXX3BvbCwgUD1kaXN0cG9sX0dlVykNCm5hbWU8LWFzX2lkcyhFKGdHZVdfcG9sKSkNCmVkZ2VfdHlwZTwtRShnR2VXX3BvbCkkc3RyZXRjaA0KRGlmZl9HX1A8LUUoZ0dlV19wb2wpJGNvbnRvcnRpb24NCmdHZVdfcG9sX2VkZ2U8LWFzLmRhdGEuZnJhbWUoY2JpbmQobmFtZSxlZGdlX3R5cGUsRGlmZl9HX1ApKQ0KDQpnU3dTX3BvbCA8LSBlZGdlX2NvbnRvcnRpb25fQ1UoZ1N3U19wb2wsIFA9ZGlzdHBvbF9Td1MpDQpuYW1lPC1hc19pZHMoRShnU3dTX3BvbCkpDQplZGdlX3R5cGU8LUUoZ1N3U19wb2wpJHN0cmV0Y2gNCkRpZmZfR19QPC1FKGdTd1NfcG9sKSRjb250b3J0aW9uDQpnU3dTX3BvbF9lZGdlPC1hcy5kYXRhLmZyYW1lKGNiaW5kKG5hbWUsZWRnZV90eXBlLERpZmZfR19QKSkNCg0KcG9sX2VkZ2U8LXJiaW5kKGdCZV9wb2xfZWRnZSxnRXN0X3BvbF9lZGdlLGdGck5fcG9sX2VkZ2UsZ0dlRV9wb2xfZWRnZSxnR2VXX3BvbF9lZGdlLGdTd1NfcG9sX2VkZ2UpDQoNCkxpbms8LXJiaW5kKGFuZV9lZGdlLG94YV9lZGdlLHBvbF9lZGdlKQ0KDQpMaW5rPC1MaW5rICU+JQ0KICBzZXBhcmF0ZShuYW1lLCBpbnRvPWMoIm5vZGVfMSIsIm5vZGVfMiIpLHNlcD0iXFx8IiklPiUNCiAgbXV0YXRlKHNwZWNpZXM9c3Vic3RyKG5vZGVfMSwxLDMpKSAlPiUNCiAgbXV0YXRlKExXPXNhcHBseShzdHJzcGxpdChub2RlXzEsIl8iKSwiWyIsMikpDQoNCkxpbms8LW1lcmdlKExpbmsscG9wYWdlZGlmZkRGLGJ5Lng9Yygibm9kZV8xIiwibm9kZV8yIiksYnkueT1jKCJJTl9QT1AiLCJORUFSX1BPUCIpKQ0Kc2F2ZShmaWxlPSJMaW5rLlJEYXRhIixsaXN0PSJMaW5rIikNCmBgYA0KDQpDZW50cmFsaXR5IG1lYXN1cmUNCmBgYHtyfQ0KYTwtYXMuZGF0YS5mcmFtZShoYXJtb25pY19jZW50cmFsaXR5X0NVKGdCZV9hbmUsbm9ybWFsaXplZD1UKSkNCmNvbG5hbWVzKGEpPC0iY2xvc2VuZXNzIg0KYjwtYXMuZGF0YS5mcmFtZShoYXJtb25pY19jZW50cmFsaXR5X0NVKGdFc3RfYW5lLG5vcm1hbGl6ZWQ9VCkpDQpjb2xuYW1lcyhiKTwtImNsb3NlbmVzcyINCmM8LWFzLmRhdGEuZnJhbWUoaGFybW9uaWNfY2VudHJhbGl0eV9DVShnRnJOX2FuZSxub3JtYWxpemVkID0gVCkpDQpjb2xuYW1lcyhjKTwtImNsb3NlbmVzcyINCmQ8LWFzLmRhdGEuZnJhbWUoaGFybW9uaWNfY2VudHJhbGl0eV9DVShnR2VFX2FuZSxub3JtYWxpemVkID0gVCkpDQpjb2xuYW1lcyhkKTwtImNsb3NlbmVzcyINCmU8LWFzLmRhdGEuZnJhbWUoaGFybW9uaWNfY2VudHJhbGl0eV9DVShnR2VXX2FuZSxub3JtYWxpemVkID0gVCkpDQpjb2xuYW1lcyhlKTwtImNsb3NlbmVzcyINCmY8LWFzLmRhdGEuZnJhbWUoaGFybW9uaWNfY2VudHJhbGl0eV9DVShnU3dTX2FuZSxub3JtYWxpemVkID0gVCkpDQpjb2xuYW1lcyhmKTwtImNsb3NlbmVzcyINCmFuZV9jbG9zZW5lc3M8LXJiaW5kKGEsYixjLGQsZSxmKQ0KDQoNCmE8LWFzLmRhdGEuZnJhbWUoaGFybW9uaWNfY2VudHJhbGl0eV9DVShnRXN0X294YSxub3JtYWxpemVkID0gVCkpDQpjb2xuYW1lcyhhKTwtImNsb3NlbmVzcyINCmI8LWFzLmRhdGEuZnJhbWUoaGFybW9uaWNfY2VudHJhbGl0eV9DVShnR2VFX294YSxub3JtYWxpemVkPVQpKQ0KY29sbmFtZXMoYik8LSJjbG9zZW5lc3MiDQpjPC1hcy5kYXRhLmZyYW1lKGhhcm1vbmljX2NlbnRyYWxpdHlfQ1UoZ0dlV19veGEsbm9ybWFsaXplZD1UKSkNCmNvbG5hbWVzKGMpPC0iY2xvc2VuZXNzIg0KZDwtYXMuZGF0YS5mcmFtZShoYXJtb25pY19jZW50cmFsaXR5X0NVKGdTd1Nfb3hhLG5vcm1hbGl6ZWQ9VCkpDQpjb2xuYW1lcyhkKTwtImNsb3NlbmVzcyINCm94YV9jbG9zZW5lc3M8LXJiaW5kKGEsYixjLGQpDQoNCmE8LWFzLmRhdGEuZnJhbWUoaGFybW9uaWNfY2VudHJhbGl0eV9DVShnQmVfcG9sLG5vcm1hbGl6ZWQ9VCkpDQpjb2xuYW1lcyhhKTwtImNsb3NlbmVzcyINCmI8LWFzLmRhdGEuZnJhbWUoaGFybW9uaWNfY2VudHJhbGl0eV9DVShnRXN0X3BvbCxub3JtYWxpemVkPVQpKSANCmNvbG5hbWVzKGIpPC0iY2xvc2VuZXNzIg0KYzwtYXMuZGF0YS5mcmFtZShoYXJtb25pY19jZW50cmFsaXR5X0NVKGdGck5fcG9sLG5vcm1hbGl6ZWQgPSBUKSkNCmNvbG5hbWVzKGMpPC0iY2xvc2VuZXNzIg0KZDwtYXMuZGF0YS5mcmFtZShoYXJtb25pY19jZW50cmFsaXR5X0NVKGdHZUVfcG9sLG5vcm1hbGl6ZWQ9VCkpDQpjb2xuYW1lcyhkKTwtImNsb3NlbmVzcyINCmU8LWFzLmRhdGEuZnJhbWUoaGFybW9uaWNfY2VudHJhbGl0eV9DVShnR2VXX3BvbCxub3JtYWxpemVkPVQpKQ0KY29sbmFtZXMoZSk8LSJjbG9zZW5lc3MiDQpmPC1hcy5kYXRhLmZyYW1lKGhhcm1vbmljX2NlbnRyYWxpdHlfQ1UoZ1N3U19wb2wsbm9ybWFsaXplZCA9IFQpKQ0KY29sbmFtZXMoZik8LSJjbG9zZW5lc3MiDQpwb2xfY2xvc2VuZXNzPC1yYmluZChhLGIsYyxkLGUsZikNCg0KY2xvc2VuZXNzPC1yYmluZChhbmVfY2xvc2VuZXNzLG94YV9jbG9zZW5lc3MscG9sX2Nsb3NlbmVzcykNCmNsb3NlbmVzcyRwb3B1bGF0aW9uPC1yb3duYW1lcyhjbG9zZW5lc3MpDQpOb2RlPC1tZXJnZShjbG9zZW5lc3MsIFBvcEFnZSxieT0icG9wdWxhdGlvbiIpDQpOb2RlPC1Ob2RlJT4lDQogIHJvd3dpc2UoKSU+JQ0KICBtdXRhdGUoQWdlX2Ficz0yMDIwLUFnZSkNCnNhdmUoZmlsZT0iTm9kZS5SRGF0YSIsbGlzdD0iTm9kZSIpDQpgYGANCg0KDQo=
