# Supplementary material for: Limited effects of population age on the genetic structure of spatially isolated forest herb populations in temperate Europe
Source: Ecol Evol. 2024 Feb 26;14(2):e10971. doi: 10.1002/ece3.10971 (PMC10897356; doi:10.1002/ece3.10971)
Supplement: Supplementary file 1 — Appendix S1. [file ECE3-14-e10971-s001.zip › 05_Preparation_For_Linear_Model.nb.html]

05\_Preparation\_For\_Linear\_Model


Code 

- Show All Code
- Hide All Code
- Download Rmd

# 05\_Preparation\_For\_Linear\_Model

Genetic Diversity


```
library(openxlsx)
library(dplyr)
library(psych)
library(car)
library(MASS)
library(tidyverse)
library(otuSummary)
source("L:/05_Data analysis/boxcox.r")

load("GenDiv_all.anem.RData")
load("GenDiv_all.oace.RData")
load("GenDiv_all.pmul.RData")

PopAge<-read.xlsx("PopAge.xlsx")  
rownames(PopAge)<-PopAge$population

df<-rbind(GenDiv_all.anem,GenDiv_all.oace,GenDiv_all.pmul)
df$Pop<-rownames(df)
blacklist<-setdiff(rownames(PopAge),rownames(df)) ## exclude populations which are excluded in the first step (due to low number of individuals)

df$LW<-as.character(lapply(strsplit(df$Pop,"_"),function(x) x[2]))
df$Species<-as.character(lapply(strsplit(df$Pop,"_"),function(x) x[1]))
df_age<-merge(PopAge,df,by.x="population",by.y="Pop")
df_age<-df_age[,c(1:11)]

load("attr.RData")## load the data of the arrtibutes of populations, including population size, species connectivity etc.
attr<-attr %>% 
  mutate(Population = str_replace(Population, "04.1", "4a"))%>%
  mutate(Population=str_replace(Population,"04.2","4b"))%>%
  mutate(Population=str_replace(Population,"04.3","4c"))%>%
  mutate(Population=str_replace(Population,"08.1","8a"))%>%
  mutate(Population=str_replace(Population,"08.2","8b"))%>%
  mutate(Population=str_replace(Population,"54_05","54e"))
attr$ID<-paste(lapply(strsplit(attr$Species.x,"_"), function(x) x[1]),"_",attr$Region,"_",attr$Population,sep="")
attr$ID[!attr$ID%in%df_age$population]

attr_pol<-subset(attr,attr$Species.x=="Pol_mul")
rownames(attr_pol)<-attr_pol$ID
attr_rest<-subset(attr,attr$Species.x!="Pol_mul")
attr<-rbind(attr_pol,attr_rest)
attr<- attr[match(df_age$population, rownames(attr)),]
GenDiv<- cbind(df_age, attr)
```


boxcox transformation and scaling


```
subane<-subset(GenDiv,GenDiv$Species.x=="Ane")
subane$PopSize_transformed<-boxcox(subane$PopSize,method="pc",lam=seq(-2,2,0.05),plot=T)
subane$IFM_transformed<-boxcox(subane$IFM,method="pc",lam=seq(-2,2,0.05),plot=T)
subane$PopSize_transformed<-as.vector(scale(subane$PopSize_transformed))
subane$IFM_transformed<-as.vector(scale(subane$IFM_transformed))
##response variables scale
subane$Ar_t<-as.vector(scale(boxcox(subane$Ar,method="pc",lam=seq(-3,3,0.05),plot=T)))
subane$Ho_t<-as.vector(scale(boxcox(subane$Ho,method="pc",lam=seq(-2,2,0.05),plot=T)))
subane$He_t<-as.vector(scale(boxcox(subane$He,method="pc",lam=seq(-20,20,0.05),plot=T))) 
subane$Fis_shifted<-subane$Fis-min(subane$Fis)## there are negative value in F value, shift it to positive range so that boxcox transformation works
subane$Fis_t<-as.vector(scale(boxcox(subane$Fis_shifted,method="pc",lam=seq(-5,5,0.05),plot=T)))

suboxa<-subset(GenDiv,GenDiv$Species.x=="Oxa")
suboxa$PopSize_transformed<-c(boxcox(suboxa$PopSize,method="pc",lam=seq(-2,2,0.05),plot=T))
suboxa$IFM_transformed<-boxcox(suboxa$IFM,method="pc",lam=seq(-2,2,0.05),plot=T)
suboxa$PopSize_transformed<-as.vector(scale(suboxa$PopSize_transformed))
suboxa$IFM_transformed<-as.vector(scale(suboxa$IFM_transformed))
suboxa$Ar_t<-as.vector(scale(boxcox(suboxa$Ar,method="pc",lam=seq(-2,2,0.05),plot=T)))
suboxa$Ho_t<-as.vector(scale(boxcox(suboxa$Ho,method="pc",lam=seq(-2,2,0.05),plot=T))) 
suboxa$He_t<-as.vector(scale(boxcox(suboxa$He,method="pc",lam=seq(-5,5,0.05),plot=T)))
suboxa$Fis_shifted<-suboxa$Fis-min(suboxa$Fis)## there are negative value in F value, shift it to positive range so that boxcox transformation works
suboxa$Fis_t<-as.vector(scale(boxcox(suboxa$Fis_shifted,method="pc",lam=seq(-2,2,0.05),plot=T)))

subpol<-subset(GenDiv,GenDiv$Species.x=="Pol")
subpol$PopSize_transformed<-boxcox(subpol$PopSize,method="pc",lam=seq(-2,2,0.05))
subpol$IFM_transformed<-boxcox(subpol$IFM,method="pc",lam=seq(-2,2,0.05))
subpol$PopSize_transformed<-as.vector(scale(subpol$PopSize_transformed))
subpol$IFM_transformed<-as.vector(scale(subpol$IFM_transformed))
subpol$Ar_t<-as.vector(scale(boxcox(subpol$Ar,method="pc",lam=seq(-3,3,0.05))))
subpol$Ho_t<-as.vector(scale(boxcox(subpol$Ho,method="pc",lam=seq(-5,5,0.05))))
subpol$He_t<-as.vector(scale(boxcox(subpol$He,method="pc",lam=seq(-10,10,0.05)))) 
subpol$Fis_shifted<-subpol$Fis-min(subpol$Fis)## there are negative value in F value, shift it to positive range so that boxcox transformation works
subpol$Fis_t<-as.vector(scale(boxcox(subpol$Fis_shifted,method="pc",lam=seq(-2,2,0.05))))
```


```
GenDiv_all<-as.data.frame(rbind(subane,suboxa,subpol))
GenDiv_all<-GenDiv_all%>%
  rowwise()%>%
  mutate(Age_abs=2020-Age)## the absolute age
hist(GenDiv_all$Age_abs)
tx<-boxcox(GenDiv_all$Age_abs,method="pc")
GenDiv_all_age_lambda<-attr(tx,"lambda")#0.45
GenDiv_all_age_added<-attr(tx,"added")#0
GenDiv_all$Age_t<-as.vector(scale(tx))
mean<-mean(tx)
sd<-sd(tx)
((50^GenDiv_all_age_lambda-1)/GenDiv_all_age_lambda-mean)/sd##-1.16596
((150^GenDiv_all_age_lambda-1)/GenDiv_all_age_lambda-mean)/sd##0.159139
(((250+GenDiv_all_age_added)^GenDiv_all_age_lambda-1)/GenDiv_all_age_lambda-mean)/sd##1.037124
(((350+GenDiv_all_age_added)^GenDiv_all_age_lambda-1)/GenDiv_all_age_lambda-mean)/sd##1.736027

save(file="GenDiv_all.RData",list="GenDiv_all")
```


Genetic Differentiation (Gst, Dps)


```
```r
PopAge<-subset(PopAge,!PopAge$population%in%blacklist)
## create a age difference matrix
list<-split(PopAge,list(PopAge$Species,PopAge$LW))
popagediffLT<-list()

for (i in 1:length(list)) {
  df<-list[[i]]
  age<-as.data.frame(df$Age)
  rownames(age)<-df$population
  popagediffLT[[i]]<-matrixConvert(dist(age))
}

popagediffDF<-NULL
for (i in 1:length(popagediffLT)){
  if (nrow(popagediffLT[[i]])!=0) popagediffDF<-rbind(popagediffDF,popagediffLT[[i]])
}
popagediffDF$sp1<-as.character(popagediffDF$sp1)
popagediffDF$sp2<-as.character(popagediffDF$sp2)


popagediffDF_a<-merge(popagediffDF,PopAge,by.x=\sp1\,by.y=\population\)
colnames(popagediffDF_a)[colnames(popagediffDF_a) == \Age\]<-c(\Age_sp1\)
popagediffDF_b<-merge(popagediffDF_a,PopAge,by.x=\sp2\,by.y=\population\)
colnames(popagediffDF_b)[colnames(popagediffDF_b) == \Age\]<-c(\Age_sp2\)
popagediffDF_b<-popagediffDF_b%>%
  rowwise()%>%
  mutate(Age_younger=max(Age_sp1,Age_sp2))
popagediffDF<-popagediffDF_b[,c(1:5,12)]
colnames(popagediffDF)<-c(\NEAR_POP\,\IN_POP\,\Age_Diff\,\Species\,\LW\,\Age_younger\)
```

```
<!-- rnb-source-end -->

<!-- rnb-chunk-end -->


<!-- rnb-chunk-begin -->


<!-- rnb-source-begin eyJkYXRhIjoiYGBgclxubG9hZChcIlBhaXJEaWZmX2FsbF9hbmVtLlJEYXRhXCIpXG5sb2FkKFwiUGFpckRpZmZfYWxsX29hY2UuUkRhdGFcIilcbmxvYWQoXCJQYWlyRGlmZl9hbGxfcG11bC5SRGF0YVwiKVxuXG5QYWlyRGlmZl9hbGwuYW5lbSRJTl9QT1A8LWFzLmNoYXJhY3RlcihQYWlyRGlmZl9hbGwuYW5lbSRJTl9QT1ApXG5QYWlyRGlmZl9hbGwuYW5lbSRORUFSX1BPUDwtYXMuY2hhcmFjdGVyKFBhaXJEaWZmX2FsbC5hbmVtJE5FQVJfUE9QKVxuUGFpckRpZmZfYWxsLm9hY2UkSU5fUE9QPC1hcy5jaGFyYWN0ZXIoUGFpckRpZmZfYWxsLm9hY2UkSU5fUE9QKVxuUGFpckRpZmZfYWxsLm9hY2UkTkVBUl9QT1A8LWFzLmNoYXJhY3RlcihQYWlyRGlmZl9hbGwub2FjZSRORUFSX1BPUClcblBhaXJEaWZmX2FsbC5wbXVsJElOX1BPUDwtYXMuY2hhcmFjdGVyKFBhaXJEaWZmX2FsbC5wbXVsJElOX1BPUClcblBhaXJEaWZmX2FsbC5wbXVsJE5FQVJfUE9QPC1hcy5jaGFyYWN0ZXIoUGFpckRpZmZfYWxsLnBtdWwkTkVBUl9QT1ApXG5cblBhaXJEaWZmX2FsbDwtcmJpbmQoUGFpckRpZmZfYWxsLmFuZW0sUGFpckRpZmZfYWxsLm9hY2UsUGFpckRpZmZfYWxsLnBtdWwpXG50ZW1wPC1wbWF4KFBhaXJEaWZmX2FsbCRJTl9QT1AsUGFpckRpZmZfYWxsJE5FQVJfUE9QKVxuUGFpckRpZmZfYWxsJElOX1BPUDwtcG1pbihQYWlyRGlmZl9hbGwkSU5fUE9QLFBhaXJEaWZmX2FsbCRORUFSX1BPUClcblBhaXJEaWZmX2FsbCRORUFSX1BPUDwtdGVtcFxuXG5cblBhaXJEaWZmX2FsbCRMaW5rPC1wYXN0ZShQYWlyRGlmZl9hbGwkSU5fUE9QLFwiX1wiLGFzLmNoYXJhY3RlcihsYXBwbHkoc3Ryc3BsaXQoUGFpckRpZmZfYWxsJE5FQVJfUE9QLFwiX1wiKSxmdW5jdGlvbih4KSB4WzNdKSksc2VwPVwiXCIpXG5cbnRlbXA8LXBtYXgocG9wYWdlZGlmZkRGJElOX1BPUCxwb3BhZ2VkaWZmREYkTkVBUl9QT1ApXG5wb3BhZ2VkaWZmREYkSU5fUE9QPC1wbWluKHBvcGFnZWRpZmZERiRJTl9QT1AscG9wYWdlZGlmZkRGJE5FQVJfUE9QKVxucG9wYWdlZGlmZkRGJE5FQVJfUE9QPC10ZW1wXG5cblBhaXJEaWZmPC1tZXJnZShQYWlyRGlmZl9hbGwscG9wYWdlZGlmZkRGLGJ5PWMoXCJJTl9QT1BcIixcIk5FQVJfUE9QXCIpKVxuXG5nZW9kaXN0PC1yZWFkLnhsc3goXCJEaXN0YW5jZXNfYW1vbmdfc3VydmV5ZWRfcG9wdWxhdGlvbnMueGxzeFwiLDEscm93TmFtZXMgPSBUKVxuZ2VvZGlzdCRYPC1wYXN0ZShnZW9kaXN0JFNwZWNpZXMsXCJfXCIsZ2VvZGlzdCRSZWdpb24sXCJfXCIsZ2VvZGlzdCRJTl9QT1AsXCJfXCIsZ2VvZGlzdCRORUFSX1BPUCxzZXA9XCJcIilcbmRpc3RfR3N0X0RwczwtbWVyZ2UoZ2VvZGlzdCxQYWlyRGlmZixieS54PVwiWFwiLGJ5Lnk9XCJMaW5rXCIpXG5cbmBgYCJ9 -->

```r
load("PairDiff_all_anem.RData")
load("PairDiff_all_oace.RData")
load("PairDiff_all_pmul.RData")

PairDiff_all.anem$IN_POP<-as.character(PairDiff_all.anem$IN_POP)
PairDiff_all.anem$NEAR_POP<-as.character(PairDiff_all.anem$NEAR_POP)
PairDiff_all.oace$IN_POP<-as.character(PairDiff_all.oace$IN_POP)
PairDiff_all.oace$NEAR_POP<-as.character(PairDiff_all.oace$NEAR_POP)
PairDiff_all.pmul$IN_POP<-as.character(PairDiff_all.pmul$IN_POP)
PairDiff_all.pmul$NEAR_POP<-as.character(PairDiff_all.pmul$NEAR_POP)

PairDiff_all<-rbind(PairDiff_all.anem,PairDiff_all.oace,PairDiff_all.pmul)
temp<-pmax(PairDiff_all$IN_POP,PairDiff_all$NEAR_POP)
PairDiff_all$IN_POP<-pmin(PairDiff_all$IN_POP,PairDiff_all$NEAR_POP)
PairDiff_all$NEAR_POP<-temp


PairDiff_all$Link<-paste(PairDiff_all$IN_POP,"_",as.character(lapply(strsplit(PairDiff_all$NEAR_POP,"_"),function(x) x[3])),sep="")

temp<-pmax(popagediffDF$IN_POP,popagediffDF$NEAR_POP)
popagediffDF$IN_POP<-pmin(popagediffDF$IN_POP,popagediffDF$NEAR_POP)
popagediffDF$NEAR_POP<-temp

PairDiff<-merge(PairDiff_all,popagediffDF,by=c("IN_POP","NEAR_POP"))

geodist<-read.xlsx("Distances_among_surveyed_populations.xlsx",1,rowNames = T)
geodist$X<-paste(geodist$Species,"_",geodist$Region,"_",geodist$IN_POP,"_",geodist$NEAR_POP,sep="")
dist_Gst_Dps<-merge(geodist,PairDiff,by.x="X",by.y="Link")
```


explanatory and response variable boxcox transformation and
scaling


```
distane<-subset(dist_Gst_Dps,dist_Gst_Dps$Species.x=="Ane")
distane$Gst_shift<-distane$Gst
distane$Gst_shift[distane$Gst_shift < 0] <- 0 ## replace negative value with 0
distane$Gst_transformed<-boxcox(distane$Gst_shift,method="pc",lam=seq(-2,2,0.05))
distane$Dps_transformed<-boxcox(distane$Dps,method="pc",lam=seq(-2,2,0.05))


distoxa<-subset(dist_Gst_Dps,dist_Gst_Dps$Species.x=="Oxa")
distoxa$Gst_shift<-distoxa$Gst
distoxa$Gst_shift[distoxa$Gst_shift < 0] <- 0
distoxa$Gst_transformed<-boxcox(distoxa$Gst_shift,method="pc",lam=seq(-2,2,0.05))
distoxa$Dps_transformed<-boxcox(distoxa$Dps,method="pc",lam=seq(-2,2,0.05))

distpol<-subset(dist_Gst_Dps,dist_Gst_Dps$Species.x=="Pol")
distpol$Gst_shift<-distpol$Gst
distpol$Gst_shift[distpol$Gst_shift < 0] <- 0
distpol$Gst_transformed<-boxcox(distpol$Gst_shift,method="pc",lam=seq(-2,2,0.05))
distpol$Dps_transformed<-boxcox(distpol$Dps,method="pc",lam=seq(-2,2,0.05))

distpol$Gst_t<-scale(distpol$Gst_transformed)
distpol$Dps_t<-scale(distpol$Dps_transformed)

distoxa$Gst_t<-scale(distoxa$Gst_transformed)
distoxa$Dps_t<-scale(distoxa$Dps_transformed)

distane$Gst_t<-scale(distane$Gst_transformed)
distane$Dps_t<-scale(distane$Dps_transformed)

dist<-rbind(distane,distoxa,distpol)

dist$Distance_t<-scale(boxcox(dist$Distance,method="pc"))
dist$Age_Diff_t<-scale(boxcox(dist$Age_Diff) )
dist<-dist%>%
  rowwise()%>%
  mutate(Age_abs_y=2020-Age_younger)
tx<-boxcox(dist$Age_abs_y)
dist$Age_younger_t<-scale(tx)

mean<-mean(tx)
sd<-sd(tx)

dist_ageabs_lambda<-attr(tx,"lambda")#0.2
dist_ageabs_added<-attr(tx,"added")#0

(((50+dist_ageabs_added)^dist_ageabs_lambda-1)/dist_ageabs_lambda-mean)/sd##-0.6929692
(((150+dist_ageabs_added)^dist_ageabs_lambda-1)/dist_ageabs_lambda-mean)/sd##0.7916849
(((250+dist_ageabs_added)^dist_ageabs_lambda-1)/dist_ageabs_lambda-mean)/sd##1.601277
(((350+dist_ageabs_added)^dist_ageabs_lambda-1)/dist_ageabs_lambda-mean)/sd##2.181551

save(file="dist.RData",list="dist")
```


Genetic Differentiation (cGD)


```
load("CD.RData")
Con.dist<-merge(geodist,CD,by.x="X",by.y="DistID")
#switch the position of in_pop and near_pop if necessary to match the order of population pair id 

Con.dist<-merge(Con.dist,popagediffDF,by.x=c("IN_POP.y","NEAR_POP.y"),by.y=c("IN_POP","NEAR_POP"))
```


explanatory and response variable boxcox transformation and
scaling


```
Con.dist.Ane<-subset(Con.dist,Con.dist$Species.x=="Ane")
Con.dist.Ane$Con.D_t<-scale(boxcox(Con.dist.Ane$Con.Dis,method="pc"))

Con.dist.Oxa<-subset(Con.dist,Con.dist$Species.x=="Oxa")
Con.dist.Oxa$Con.D_t<-scale(boxcox(Con.dist.Oxa$Con.Dis,method="pc"))

Con.dist.Pol<-subset(Con.dist,Con.dist$Species.x=="Pol")
Con.dist.Pol$Con.D_t<-scale(boxcox(Con.dist.Pol$Con.Dis,method="pc",plot=T))

Con.dist<-rbind(Con.dist.Ane,Con.dist.Oxa,Con.dist.Pol)

Con.dist$geodist_t<-scale(boxcox(Con.dist$Distance))
Con.dist$Age_Diff_t<-scale(boxcox(Con.dist$Age_Diff))
Con.dist<-Con.dist%>%
  rowwise()%>%
  mutate(Age_abs_y=2020-Age_younger)
tx<-boxcox(Con.dist$Age_abs_y)
Con.dist$Age_younger_t<-scale(tx)
mean<-mean(tx)
sd<-sd(tx)
Condist_age_lambda<-attr(tx,"lambda")##0.15
Condist_age_added<-attr(tx,"added")##0
(((16+Condist_age_added)^Condist_age_lambda-1)/Condist_age_lambda-mean)/sd##-2.037879
(((50+Condist_age_added)^Condist_age_lambda-1)/Condist_age_lambda-mean)/sd##-0.7075473
(((150+Condist_age_added)^Condist_age_lambda-1)/Condist_age_lambda-mean)/sd##0.8094188
(((250+Condist_age_added)^Condist_age_lambda-1)/Condist_age_lambda-mean)/sd##1.604555
(((350+Condist_age_added)^Condist_age_lambda-1)/Condist_age_lambda-mean)/sd##2.162584


Con.Dis<-Con.dist[,c(1:5,8:9,13,16:21)]
save(file="Con_Dis.RData",list="Con.Dis")
```


LS0tDQp0aXRsZTogIjA1X1ByZXBhcmF0aW9uX0Zvcl9MaW5lYXJfTW9kZWwiDQpvdXRwdXQ6IGh0bWxfbm90ZWJvb2sNCi0tLQ0KDQpHZW5ldGljIERpdmVyc2l0eQ0KYGBge3J9DQpsaWJyYXJ5KG9wZW54bHN4KQ0KbGlicmFyeShkcGx5cikNCmxpYnJhcnkocHN5Y2gpDQpsaWJyYXJ5KGNhcikNCmxpYnJhcnkoTUFTUykNCmxpYnJhcnkodGlkeXZlcnNlKQ0KbGlicmFyeShvdHVTdW1tYXJ5KQ0Kc291cmNlKCJMOi8wNV9EYXRhIGFuYWx5c2lzL2JveGNveC5yIikNCg0KbG9hZCgiR2VuRGl2X2FsbC5hbmVtLlJEYXRhIikNCmxvYWQoIkdlbkRpdl9hbGwub2FjZS5SRGF0YSIpDQpsb2FkKCJHZW5EaXZfYWxsLnBtdWwuUkRhdGEiKQ0KDQpQb3BBZ2U8LXJlYWQueGxzeCgiUG9wQWdlLnhsc3giKSAgDQpyb3duYW1lcyhQb3BBZ2UpPC1Qb3BBZ2UkcG9wdWxhdGlvbg0KDQpkZjwtcmJpbmQoR2VuRGl2X2FsbC5hbmVtLEdlbkRpdl9hbGwub2FjZSxHZW5EaXZfYWxsLnBtdWwpDQpkZiRQb3A8LXJvd25hbWVzKGRmKQ0KYmxhY2tsaXN0PC1zZXRkaWZmKHJvd25hbWVzKFBvcEFnZSkscm93bmFtZXMoZGYpKSAjIyBleGNsdWRlIHBvcHVsYXRpb25zIHdoaWNoIGFyZSBleGNsdWRlZCBpbiB0aGUgZmlyc3Qgc3RlcCAoZHVlIHRvIGxvdyBudW1iZXIgb2YgaW5kaXZpZHVhbHMpDQoNCmRmJExXPC1hcy5jaGFyYWN0ZXIobGFwcGx5KHN0cnNwbGl0KGRmJFBvcCwiXyIpLGZ1bmN0aW9uKHgpIHhbMl0pKQ0KZGYkU3BlY2llczwtYXMuY2hhcmFjdGVyKGxhcHBseShzdHJzcGxpdChkZiRQb3AsIl8iKSxmdW5jdGlvbih4KSB4WzFdKSkNCmRmX2FnZTwtbWVyZ2UoUG9wQWdlLGRmLGJ5Lng9InBvcHVsYXRpb24iLGJ5Lnk9IlBvcCIpDQpkZl9hZ2U8LWRmX2FnZVssYygxOjExKV0NCg0KbG9hZCgiYXR0ci5SRGF0YSIpIyMgbG9hZCB0aGUgZGF0YSBvZiB0aGUgYXJydGlidXRlcyBvZiBwb3B1bGF0aW9ucywgaW5jbHVkaW5nIHBvcHVsYXRpb24gc2l6ZSwgc3BlY2llcyBjb25uZWN0aXZpdHkgZXRjLg0KYXR0cjwtYXR0ciAlPiUgDQogIG11dGF0ZShQb3B1bGF0aW9uID0gc3RyX3JlcGxhY2UoUG9wdWxhdGlvbiwgIjA0LjEiLCAiNGEiKSklPiUNCiAgbXV0YXRlKFBvcHVsYXRpb249c3RyX3JlcGxhY2UoUG9wdWxhdGlvbiwiMDQuMiIsIjRiIikpJT4lDQogIG11dGF0ZShQb3B1bGF0aW9uPXN0cl9yZXBsYWNlKFBvcHVsYXRpb24sIjA0LjMiLCI0YyIpKSU+JQ0KICBtdXRhdGUoUG9wdWxhdGlvbj1zdHJfcmVwbGFjZShQb3B1bGF0aW9uLCIwOC4xIiwiOGEiKSklPiUNCiAgbXV0YXRlKFBvcHVsYXRpb249c3RyX3JlcGxhY2UoUG9wdWxhdGlvbiwiMDguMiIsIjhiIikpJT4lDQogIG11dGF0ZShQb3B1bGF0aW9uPXN0cl9yZXBsYWNlKFBvcHVsYXRpb24sIjU0XzA1IiwiNTRlIikpDQphdHRyJElEPC1wYXN0ZShsYXBwbHkoc3Ryc3BsaXQoYXR0ciRTcGVjaWVzLngsIl8iKSwgZnVuY3Rpb24oeCkgeFsxXSksIl8iLGF0dHIkUmVnaW9uLCJfIixhdHRyJFBvcHVsYXRpb24sc2VwPSIiKQ0KYXR0ciRJRFshYXR0ciRJRCVpbiVkZl9hZ2UkcG9wdWxhdGlvbl0NCg0KYXR0cl9wb2w8LXN1YnNldChhdHRyLGF0dHIkU3BlY2llcy54PT0iUG9sX211bCIpDQpyb3duYW1lcyhhdHRyX3BvbCk8LWF0dHJfcG9sJElEDQphdHRyX3Jlc3Q8LXN1YnNldChhdHRyLGF0dHIkU3BlY2llcy54IT0iUG9sX211bCIpDQphdHRyPC1yYmluZChhdHRyX3BvbCxhdHRyX3Jlc3QpDQphdHRyPC0gYXR0clttYXRjaChkZl9hZ2UkcG9wdWxhdGlvbiwgcm93bmFtZXMoYXR0cikpLF0NCkdlbkRpdjwtIGNiaW5kKGRmX2FnZSwgYXR0cikNCg0KYGBgDQpib3hjb3ggdHJhbnNmb3JtYXRpb24gYW5kIHNjYWxpbmcNCmBgYHtyfQ0Kc3ViYW5lPC1zdWJzZXQoR2VuRGl2LEdlbkRpdiRTcGVjaWVzLng9PSJBbmUiKQ0Kc3ViYW5lJFBvcFNpemVfdHJhbnNmb3JtZWQ8LWJveGNveChzdWJhbmUkUG9wU2l6ZSxtZXRob2Q9InBjIixsYW09c2VxKC0yLDIsMC4wNSkscGxvdD1UKQ0Kc3ViYW5lJElGTV90cmFuc2Zvcm1lZDwtYm94Y294KHN1YmFuZSRJRk0sbWV0aG9kPSJwYyIsbGFtPXNlcSgtMiwyLDAuMDUpLHBsb3Q9VCkNCnN1YmFuZSRQb3BTaXplX3RyYW5zZm9ybWVkPC1hcy52ZWN0b3Ioc2NhbGUoc3ViYW5lJFBvcFNpemVfdHJhbnNmb3JtZWQpKQ0Kc3ViYW5lJElGTV90cmFuc2Zvcm1lZDwtYXMudmVjdG9yKHNjYWxlKHN1YmFuZSRJRk1fdHJhbnNmb3JtZWQpKQ0KIyNyZXNwb25zZSB2YXJpYWJsZXMgc2NhbGUNCnN1YmFuZSRBcl90PC1hcy52ZWN0b3Ioc2NhbGUoYm94Y294KHN1YmFuZSRBcixtZXRob2Q9InBjIixsYW09c2VxKC0zLDMsMC4wNSkscGxvdD1UKSkpDQpzdWJhbmUkSG9fdDwtYXMudmVjdG9yKHNjYWxlKGJveGNveChzdWJhbmUkSG8sbWV0aG9kPSJwYyIsbGFtPXNlcSgtMiwyLDAuMDUpLHBsb3Q9VCkpKQ0Kc3ViYW5lJEhlX3Q8LWFzLnZlY3RvcihzY2FsZShib3hjb3goc3ViYW5lJEhlLG1ldGhvZD0icGMiLGxhbT1zZXEoLTIwLDIwLDAuMDUpLHBsb3Q9VCkpKSANCnN1YmFuZSRGaXNfc2hpZnRlZDwtc3ViYW5lJEZpcy1taW4oc3ViYW5lJEZpcykjIyB0aGVyZSBhcmUgbmVnYXRpdmUgdmFsdWUgaW4gRiB2YWx1ZSwgc2hpZnQgaXQgdG8gcG9zaXRpdmUgcmFuZ2Ugc28gdGhhdCBib3hjb3ggdHJhbnNmb3JtYXRpb24gd29ya3MNCnN1YmFuZSRGaXNfdDwtYXMudmVjdG9yKHNjYWxlKGJveGNveChzdWJhbmUkRmlzX3NoaWZ0ZWQsbWV0aG9kPSJwYyIsbGFtPXNlcSgtNSw1LDAuMDUpLHBsb3Q9VCkpKQ0KDQpzdWJveGE8LXN1YnNldChHZW5EaXYsR2VuRGl2JFNwZWNpZXMueD09Ik94YSIpDQpzdWJveGEkUG9wU2l6ZV90cmFuc2Zvcm1lZDwtYyhib3hjb3goc3Vib3hhJFBvcFNpemUsbWV0aG9kPSJwYyIsbGFtPXNlcSgtMiwyLDAuMDUpLHBsb3Q9VCkpDQpzdWJveGEkSUZNX3RyYW5zZm9ybWVkPC1ib3hjb3goc3Vib3hhJElGTSxtZXRob2Q9InBjIixsYW09c2VxKC0yLDIsMC4wNSkscGxvdD1UKQ0Kc3Vib3hhJFBvcFNpemVfdHJhbnNmb3JtZWQ8LWFzLnZlY3RvcihzY2FsZShzdWJveGEkUG9wU2l6ZV90cmFuc2Zvcm1lZCkpDQpzdWJveGEkSUZNX3RyYW5zZm9ybWVkPC1hcy52ZWN0b3Ioc2NhbGUoc3Vib3hhJElGTV90cmFuc2Zvcm1lZCkpDQpzdWJveGEkQXJfdDwtYXMudmVjdG9yKHNjYWxlKGJveGNveChzdWJveGEkQXIsbWV0aG9kPSJwYyIsbGFtPXNlcSgtMiwyLDAuMDUpLHBsb3Q9VCkpKQ0Kc3Vib3hhJEhvX3Q8LWFzLnZlY3RvcihzY2FsZShib3hjb3goc3Vib3hhJEhvLG1ldGhvZD0icGMiLGxhbT1zZXEoLTIsMiwwLjA1KSxwbG90PVQpKSkgDQpzdWJveGEkSGVfdDwtYXMudmVjdG9yKHNjYWxlKGJveGNveChzdWJveGEkSGUsbWV0aG9kPSJwYyIsbGFtPXNlcSgtNSw1LDAuMDUpLHBsb3Q9VCkpKQ0Kc3Vib3hhJEZpc19zaGlmdGVkPC1zdWJveGEkRmlzLW1pbihzdWJveGEkRmlzKSMjIHRoZXJlIGFyZSBuZWdhdGl2ZSB2YWx1ZSBpbiBGIHZhbHVlLCBzaGlmdCBpdCB0byBwb3NpdGl2ZSByYW5nZSBzbyB0aGF0IGJveGNveCB0cmFuc2Zvcm1hdGlvbiB3b3Jrcw0Kc3Vib3hhJEZpc190PC1hcy52ZWN0b3Ioc2NhbGUoYm94Y294KHN1Ym94YSRGaXNfc2hpZnRlZCxtZXRob2Q9InBjIixsYW09c2VxKC0yLDIsMC4wNSkscGxvdD1UKSkpDQoNCnN1YnBvbDwtc3Vic2V0KEdlbkRpdixHZW5EaXYkU3BlY2llcy54PT0iUG9sIikNCnN1YnBvbCRQb3BTaXplX3RyYW5zZm9ybWVkPC1ib3hjb3goc3VicG9sJFBvcFNpemUsbWV0aG9kPSJwYyIsbGFtPXNlcSgtMiwyLDAuMDUpKQ0Kc3VicG9sJElGTV90cmFuc2Zvcm1lZDwtYm94Y294KHN1YnBvbCRJRk0sbWV0aG9kPSJwYyIsbGFtPXNlcSgtMiwyLDAuMDUpKQ0Kc3VicG9sJFBvcFNpemVfdHJhbnNmb3JtZWQ8LWFzLnZlY3RvcihzY2FsZShzdWJwb2wkUG9wU2l6ZV90cmFuc2Zvcm1lZCkpDQpzdWJwb2wkSUZNX3RyYW5zZm9ybWVkPC1hcy52ZWN0b3Ioc2NhbGUoc3VicG9sJElGTV90cmFuc2Zvcm1lZCkpDQpzdWJwb2wkQXJfdDwtYXMudmVjdG9yKHNjYWxlKGJveGNveChzdWJwb2wkQXIsbWV0aG9kPSJwYyIsbGFtPXNlcSgtMywzLDAuMDUpKSkpDQpzdWJwb2wkSG9fdDwtYXMudmVjdG9yKHNjYWxlKGJveGNveChzdWJwb2wkSG8sbWV0aG9kPSJwYyIsbGFtPXNlcSgtNSw1LDAuMDUpKSkpDQpzdWJwb2wkSGVfdDwtYXMudmVjdG9yKHNjYWxlKGJveGNveChzdWJwb2wkSGUsbWV0aG9kPSJwYyIsbGFtPXNlcSgtMTAsMTAsMC4wNSkpKSkgDQpzdWJwb2wkRmlzX3NoaWZ0ZWQ8LXN1YnBvbCRGaXMtbWluKHN1YnBvbCRGaXMpIyMgdGhlcmUgYXJlIG5lZ2F0aXZlIHZhbHVlIGluIEYgdmFsdWUsIHNoaWZ0IGl0IHRvIHBvc2l0aXZlIHJhbmdlIHNvIHRoYXQgYm94Y294IHRyYW5zZm9ybWF0aW9uIHdvcmtzDQpzdWJwb2wkRmlzX3Q8LWFzLnZlY3RvcihzY2FsZShib3hjb3goc3VicG9sJEZpc19zaGlmdGVkLG1ldGhvZD0icGMiLGxhbT1zZXEoLTIsMiwwLjA1KSkpKQ0KDQpgYGANCmBgYHtyfQ0KR2VuRGl2X2FsbDwtYXMuZGF0YS5mcmFtZShyYmluZChzdWJhbmUsc3Vib3hhLHN1YnBvbCkpDQpHZW5EaXZfYWxsPC1HZW5EaXZfYWxsJT4lDQogIHJvd3dpc2UoKSU+JQ0KICBtdXRhdGUoQWdlX2Ficz0yMDIwLUFnZSkjIyB0aGUgYWJzb2x1dGUgYWdlDQpoaXN0KEdlbkRpdl9hbGwkQWdlX2FicykNCnR4PC1ib3hjb3goR2VuRGl2X2FsbCRBZ2VfYWJzLG1ldGhvZD0icGMiKQ0KR2VuRGl2X2FsbF9hZ2VfbGFtYmRhPC1hdHRyKHR4LCJsYW1iZGEiKSMwLjQ1DQpHZW5EaXZfYWxsX2FnZV9hZGRlZDwtYXR0cih0eCwiYWRkZWQiKSMwDQpHZW5EaXZfYWxsJEFnZV90PC1hcy52ZWN0b3Ioc2NhbGUodHgpKQ0KbWVhbjwtbWVhbih0eCkNCnNkPC1zZCh0eCkNCigoNTBeR2VuRGl2X2FsbF9hZ2VfbGFtYmRhLTEpL0dlbkRpdl9hbGxfYWdlX2xhbWJkYS1tZWFuKS9zZCMjLTEuMTY1OTYNCigoMTUwXkdlbkRpdl9hbGxfYWdlX2xhbWJkYS0xKS9HZW5EaXZfYWxsX2FnZV9sYW1iZGEtbWVhbikvc2QjIzAuMTU5MTM5DQooKCgyNTArR2VuRGl2X2FsbF9hZ2VfYWRkZWQpXkdlbkRpdl9hbGxfYWdlX2xhbWJkYS0xKS9HZW5EaXZfYWxsX2FnZV9sYW1iZGEtbWVhbikvc2QjIzEuMDM3MTI0DQooKCgzNTArR2VuRGl2X2FsbF9hZ2VfYWRkZWQpXkdlbkRpdl9hbGxfYWdlX2xhbWJkYS0xKS9HZW5EaXZfYWxsX2FnZV9sYW1iZGEtbWVhbikvc2QjIzEuNzM2MDI3DQoNCnNhdmUoZmlsZT0iR2VuRGl2X2FsbC5SRGF0YSIsbGlzdD0iR2VuRGl2X2FsbCIpDQpgYGANCg0KR2VuZXRpYyBEaWZmZXJlbnRpYXRpb24gKEdzdCwgRHBzKQ0KYGBge3J9DQpQb3BBZ2U8LXN1YnNldChQb3BBZ2UsIVBvcEFnZSRwb3B1bGF0aW9uJWluJWJsYWNrbGlzdCkNCiMjIGNyZWF0ZSBhIGFnZSBkaWZmZXJlbmNlIG1hdHJpeA0KbGlzdDwtc3BsaXQoUG9wQWdlLGxpc3QoUG9wQWdlJFNwZWNpZXMsUG9wQWdlJExXKSkNCnBvcGFnZWRpZmZMVDwtbGlzdCgpDQoNCmZvciAoaSBpbiAxOmxlbmd0aChsaXN0KSkgew0KICBkZjwtbGlzdFtbaV1dDQogIGFnZTwtYXMuZGF0YS5mcmFtZShkZiRBZ2UpDQogIHJvd25hbWVzKGFnZSk8LWRmJHBvcHVsYXRpb24NCiAgcG9wYWdlZGlmZkxUW1tpXV08LW1hdHJpeENvbnZlcnQoZGlzdChhZ2UpKQ0KfQ0KDQpwb3BhZ2VkaWZmREY8LU5VTEwNCmZvciAoaSBpbiAxOmxlbmd0aChwb3BhZ2VkaWZmTFQpKXsNCiAgaWYgKG5yb3cocG9wYWdlZGlmZkxUW1tpXV0pIT0wKSBwb3BhZ2VkaWZmREY8LXJiaW5kKHBvcGFnZWRpZmZERixwb3BhZ2VkaWZmTFRbW2ldXSkNCn0NCnBvcGFnZWRpZmZERiRzcDE8LWFzLmNoYXJhY3Rlcihwb3BhZ2VkaWZmREYkc3AxKQ0KcG9wYWdlZGlmZkRGJHNwMjwtYXMuY2hhcmFjdGVyKHBvcGFnZWRpZmZERiRzcDIpDQoNCg0KcG9wYWdlZGlmZkRGX2E8LW1lcmdlKHBvcGFnZWRpZmZERixQb3BBZ2UsYnkueD0ic3AxIixieS55PSJwb3B1bGF0aW9uIikNCmNvbG5hbWVzKHBvcGFnZWRpZmZERl9hKVtjb2xuYW1lcyhwb3BhZ2VkaWZmREZfYSkgPT0gIkFnZSJdPC1jKCJBZ2Vfc3AxIikNCnBvcGFnZWRpZmZERl9iPC1tZXJnZShwb3BhZ2VkaWZmREZfYSxQb3BBZ2UsYnkueD0ic3AyIixieS55PSJwb3B1bGF0aW9uIikNCmNvbG5hbWVzKHBvcGFnZWRpZmZERl9iKVtjb2xuYW1lcyhwb3BhZ2VkaWZmREZfYikgPT0gIkFnZSJdPC1jKCJBZ2Vfc3AyIikNCnBvcGFnZWRpZmZERl9iPC1wb3BhZ2VkaWZmREZfYiU+JQ0KICByb3d3aXNlKCklPiUNCiAgbXV0YXRlKEFnZV95b3VuZ2VyPW1heChBZ2Vfc3AxLEFnZV9zcDIpKQ0KcG9wYWdlZGlmZkRGPC1wb3BhZ2VkaWZmREZfYlssYygxOjUsMTIpXQ0KY29sbmFtZXMocG9wYWdlZGlmZkRGKTwtYygiTkVBUl9QT1AiLCJJTl9QT1AiLCJBZ2VfRGlmZiIsIlNwZWNpZXMiLCJMVyIsIkFnZV95b3VuZ2VyIikNCmBgYA0KYGBge3J9DQpsb2FkKCJQYWlyRGlmZl9hbGxfYW5lbS5SRGF0YSIpDQpsb2FkKCJQYWlyRGlmZl9hbGxfb2FjZS5SRGF0YSIpDQpsb2FkKCJQYWlyRGlmZl9hbGxfcG11bC5SRGF0YSIpDQoNClBhaXJEaWZmX2FsbC5hbmVtJElOX1BPUDwtYXMuY2hhcmFjdGVyKFBhaXJEaWZmX2FsbC5hbmVtJElOX1BPUCkNClBhaXJEaWZmX2FsbC5hbmVtJE5FQVJfUE9QPC1hcy5jaGFyYWN0ZXIoUGFpckRpZmZfYWxsLmFuZW0kTkVBUl9QT1ApDQpQYWlyRGlmZl9hbGwub2FjZSRJTl9QT1A8LWFzLmNoYXJhY3RlcihQYWlyRGlmZl9hbGwub2FjZSRJTl9QT1ApDQpQYWlyRGlmZl9hbGwub2FjZSRORUFSX1BPUDwtYXMuY2hhcmFjdGVyKFBhaXJEaWZmX2FsbC5vYWNlJE5FQVJfUE9QKQ0KUGFpckRpZmZfYWxsLnBtdWwkSU5fUE9QPC1hcy5jaGFyYWN0ZXIoUGFpckRpZmZfYWxsLnBtdWwkSU5fUE9QKQ0KUGFpckRpZmZfYWxsLnBtdWwkTkVBUl9QT1A8LWFzLmNoYXJhY3RlcihQYWlyRGlmZl9hbGwucG11bCRORUFSX1BPUCkNCg0KUGFpckRpZmZfYWxsPC1yYmluZChQYWlyRGlmZl9hbGwuYW5lbSxQYWlyRGlmZl9hbGwub2FjZSxQYWlyRGlmZl9hbGwucG11bCkNCnRlbXA8LXBtYXgoUGFpckRpZmZfYWxsJElOX1BPUCxQYWlyRGlmZl9hbGwkTkVBUl9QT1ApDQpQYWlyRGlmZl9hbGwkSU5fUE9QPC1wbWluKFBhaXJEaWZmX2FsbCRJTl9QT1AsUGFpckRpZmZfYWxsJE5FQVJfUE9QKQ0KUGFpckRpZmZfYWxsJE5FQVJfUE9QPC10ZW1wDQoNCg0KUGFpckRpZmZfYWxsJExpbms8LXBhc3RlKFBhaXJEaWZmX2FsbCRJTl9QT1AsIl8iLGFzLmNoYXJhY3RlcihsYXBwbHkoc3Ryc3BsaXQoUGFpckRpZmZfYWxsJE5FQVJfUE9QLCJfIiksZnVuY3Rpb24oeCkgeFszXSkpLHNlcD0iIikNCg0KdGVtcDwtcG1heChwb3BhZ2VkaWZmREYkSU5fUE9QLHBvcGFnZWRpZmZERiRORUFSX1BPUCkNCnBvcGFnZWRpZmZERiRJTl9QT1A8LXBtaW4ocG9wYWdlZGlmZkRGJElOX1BPUCxwb3BhZ2VkaWZmREYkTkVBUl9QT1ApDQpwb3BhZ2VkaWZmREYkTkVBUl9QT1A8LXRlbXANCg0KUGFpckRpZmY8LW1lcmdlKFBhaXJEaWZmX2FsbCxwb3BhZ2VkaWZmREYsYnk9YygiSU5fUE9QIiwiTkVBUl9QT1AiKSkNCg0KZ2VvZGlzdDwtcmVhZC54bHN4KCJEaXN0YW5jZXNfYW1vbmdfc3VydmV5ZWRfcG9wdWxhdGlvbnMueGxzeCIsMSxyb3dOYW1lcyA9IFQpDQpnZW9kaXN0JFg8LXBhc3RlKGdlb2Rpc3QkU3BlY2llcywiXyIsZ2VvZGlzdCRSZWdpb24sIl8iLGdlb2Rpc3QkSU5fUE9QLCJfIixnZW9kaXN0JE5FQVJfUE9QLHNlcD0iIikNCmRpc3RfR3N0X0RwczwtbWVyZ2UoZ2VvZGlzdCxQYWlyRGlmZixieS54PSJYIixieS55PSJMaW5rIikNCg0KYGBgDQpleHBsYW5hdG9yeSBhbmQgcmVzcG9uc2UgdmFyaWFibGUgYm94Y294IHRyYW5zZm9ybWF0aW9uIGFuZCBzY2FsaW5nDQpgYGB7cn0NCmRpc3RhbmU8LXN1YnNldChkaXN0X0dzdF9EcHMsZGlzdF9Hc3RfRHBzJFNwZWNpZXMueD09IkFuZSIpDQpkaXN0YW5lJEdzdF9zaGlmdDwtZGlzdGFuZSRHc3QNCmRpc3RhbmUkR3N0X3NoaWZ0W2Rpc3RhbmUkR3N0X3NoaWZ0IDwgMF0gPC0gMCAjIyByZXBsYWNlIG5lZ2F0aXZlIHZhbHVlIHdpdGggMA0KZGlzdGFuZSRHc3RfdHJhbnNmb3JtZWQ8LWJveGNveChkaXN0YW5lJEdzdF9zaGlmdCxtZXRob2Q9InBjIixsYW09c2VxKC0yLDIsMC4wNSkpDQpkaXN0YW5lJERwc190cmFuc2Zvcm1lZDwtYm94Y294KGRpc3RhbmUkRHBzLG1ldGhvZD0icGMiLGxhbT1zZXEoLTIsMiwwLjA1KSkNCg0KDQpkaXN0b3hhPC1zdWJzZXQoZGlzdF9Hc3RfRHBzLGRpc3RfR3N0X0RwcyRTcGVjaWVzLng9PSJPeGEiKQ0KZGlzdG94YSRHc3Rfc2hpZnQ8LWRpc3RveGEkR3N0DQpkaXN0b3hhJEdzdF9zaGlmdFtkaXN0b3hhJEdzdF9zaGlmdCA8IDBdIDwtIDANCmRpc3RveGEkR3N0X3RyYW5zZm9ybWVkPC1ib3hjb3goZGlzdG94YSRHc3Rfc2hpZnQsbWV0aG9kPSJwYyIsbGFtPXNlcSgtMiwyLDAuMDUpKQ0KZGlzdG94YSREcHNfdHJhbnNmb3JtZWQ8LWJveGNveChkaXN0b3hhJERwcyxtZXRob2Q9InBjIixsYW09c2VxKC0yLDIsMC4wNSkpDQoNCmRpc3Rwb2w8LXN1YnNldChkaXN0X0dzdF9EcHMsZGlzdF9Hc3RfRHBzJFNwZWNpZXMueD09IlBvbCIpDQpkaXN0cG9sJEdzdF9zaGlmdDwtZGlzdHBvbCRHc3QNCmRpc3Rwb2wkR3N0X3NoaWZ0W2Rpc3Rwb2wkR3N0X3NoaWZ0IDwgMF0gPC0gMA0KZGlzdHBvbCRHc3RfdHJhbnNmb3JtZWQ8LWJveGNveChkaXN0cG9sJEdzdF9zaGlmdCxtZXRob2Q9InBjIixsYW09c2VxKC0yLDIsMC4wNSkpDQpkaXN0cG9sJERwc190cmFuc2Zvcm1lZDwtYm94Y294KGRpc3Rwb2wkRHBzLG1ldGhvZD0icGMiLGxhbT1zZXEoLTIsMiwwLjA1KSkNCg0KZGlzdHBvbCRHc3RfdDwtc2NhbGUoZGlzdHBvbCRHc3RfdHJhbnNmb3JtZWQpDQpkaXN0cG9sJERwc190PC1zY2FsZShkaXN0cG9sJERwc190cmFuc2Zvcm1lZCkNCg0KZGlzdG94YSRHc3RfdDwtc2NhbGUoZGlzdG94YSRHc3RfdHJhbnNmb3JtZWQpDQpkaXN0b3hhJERwc190PC1zY2FsZShkaXN0b3hhJERwc190cmFuc2Zvcm1lZCkNCg0KZGlzdGFuZSRHc3RfdDwtc2NhbGUoZGlzdGFuZSRHc3RfdHJhbnNmb3JtZWQpDQpkaXN0YW5lJERwc190PC1zY2FsZShkaXN0YW5lJERwc190cmFuc2Zvcm1lZCkNCg0KZGlzdDwtcmJpbmQoZGlzdGFuZSxkaXN0b3hhLGRpc3Rwb2wpDQoNCmRpc3QkRGlzdGFuY2VfdDwtc2NhbGUoYm94Y294KGRpc3QkRGlzdGFuY2UsbWV0aG9kPSJwYyIpKQ0KZGlzdCRBZ2VfRGlmZl90PC1zY2FsZShib3hjb3goZGlzdCRBZ2VfRGlmZikgKQ0KZGlzdDwtZGlzdCU+JQ0KICByb3d3aXNlKCklPiUNCiAgbXV0YXRlKEFnZV9hYnNfeT0yMDIwLUFnZV95b3VuZ2VyKQ0KdHg8LWJveGNveChkaXN0JEFnZV9hYnNfeSkNCmRpc3QkQWdlX3lvdW5nZXJfdDwtc2NhbGUodHgpDQoNCm1lYW48LW1lYW4odHgpDQpzZDwtc2QodHgpDQoNCmRpc3RfYWdlYWJzX2xhbWJkYTwtYXR0cih0eCwibGFtYmRhIikjMC4yDQpkaXN0X2FnZWFic19hZGRlZDwtYXR0cih0eCwiYWRkZWQiKSMwDQoNCigoKDUwK2Rpc3RfYWdlYWJzX2FkZGVkKV5kaXN0X2FnZWFic19sYW1iZGEtMSkvZGlzdF9hZ2VhYnNfbGFtYmRhLW1lYW4pL3NkIyMtMC42OTI5NjkyDQooKCgxNTArZGlzdF9hZ2VhYnNfYWRkZWQpXmRpc3RfYWdlYWJzX2xhbWJkYS0xKS9kaXN0X2FnZWFic19sYW1iZGEtbWVhbikvc2QjIzAuNzkxNjg0OQ0KKCgoMjUwK2Rpc3RfYWdlYWJzX2FkZGVkKV5kaXN0X2FnZWFic19sYW1iZGEtMSkvZGlzdF9hZ2VhYnNfbGFtYmRhLW1lYW4pL3NkIyMxLjYwMTI3Nw0KKCgoMzUwK2Rpc3RfYWdlYWJzX2FkZGVkKV5kaXN0X2FnZWFic19sYW1iZGEtMSkvZGlzdF9hZ2VhYnNfbGFtYmRhLW1lYW4pL3NkIyMyLjE4MTU1MQ0KDQpzYXZlKGZpbGU9ImRpc3QuUkRhdGEiLGxpc3Q9ImRpc3QiKQ0KYGBgDQpHZW5ldGljIERpZmZlcmVudGlhdGlvbiAoY0dEKQ0KYGBge3J9DQpsb2FkKCJDRC5SRGF0YSIpDQpDb24uZGlzdDwtbWVyZ2UoZ2VvZGlzdCxDRCxieS54PSJYIixieS55PSJEaXN0SUQiKQ0KI3N3aXRjaCB0aGUgcG9zaXRpb24gb2YgaW5fcG9wIGFuZCBuZWFyX3BvcCBpZiBuZWNlc3NhcnkgdG8gbWF0Y2ggdGhlIG9yZGVyIG9mIHBvcHVsYXRpb24gcGFpciBpZCANCg0KQ29uLmRpc3Q8LW1lcmdlKENvbi5kaXN0LHBvcGFnZWRpZmZERixieS54PWMoIklOX1BPUC55IiwiTkVBUl9QT1AueSIpLGJ5Lnk9YygiSU5fUE9QIiwiTkVBUl9QT1AiKSkNCg0KYGBgDQpleHBsYW5hdG9yeSBhbmQgcmVzcG9uc2UgdmFyaWFibGUgYm94Y294IHRyYW5zZm9ybWF0aW9uIGFuZCBzY2FsaW5nDQpgYGB7cn0NCkNvbi5kaXN0LkFuZTwtc3Vic2V0KENvbi5kaXN0LENvbi5kaXN0JFNwZWNpZXMueD09IkFuZSIpDQpDb24uZGlzdC5BbmUkQ29uLkRfdDwtc2NhbGUoYm94Y294KENvbi5kaXN0LkFuZSRDb24uRGlzLG1ldGhvZD0icGMiKSkNCg0KQ29uLmRpc3QuT3hhPC1zdWJzZXQoQ29uLmRpc3QsQ29uLmRpc3QkU3BlY2llcy54PT0iT3hhIikNCkNvbi5kaXN0Lk94YSRDb24uRF90PC1zY2FsZShib3hjb3goQ29uLmRpc3QuT3hhJENvbi5EaXMsbWV0aG9kPSJwYyIpKQ0KDQpDb24uZGlzdC5Qb2w8LXN1YnNldChDb24uZGlzdCxDb24uZGlzdCRTcGVjaWVzLng9PSJQb2wiKQ0KQ29uLmRpc3QuUG9sJENvbi5EX3Q8LXNjYWxlKGJveGNveChDb24uZGlzdC5Qb2wkQ29uLkRpcyxtZXRob2Q9InBjIixwbG90PVQpKQ0KDQpDb24uZGlzdDwtcmJpbmQoQ29uLmRpc3QuQW5lLENvbi5kaXN0Lk94YSxDb24uZGlzdC5Qb2wpDQoNCkNvbi5kaXN0JGdlb2Rpc3RfdDwtc2NhbGUoYm94Y294KENvbi5kaXN0JERpc3RhbmNlKSkNCkNvbi5kaXN0JEFnZV9EaWZmX3Q8LXNjYWxlKGJveGNveChDb24uZGlzdCRBZ2VfRGlmZikpDQpDb24uZGlzdDwtQ29uLmRpc3QlPiUNCiAgcm93d2lzZSgpJT4lDQogIG11dGF0ZShBZ2VfYWJzX3k9MjAyMC1BZ2VfeW91bmdlcikNCnR4PC1ib3hjb3goQ29uLmRpc3QkQWdlX2Fic195KQ0KQ29uLmRpc3QkQWdlX3lvdW5nZXJfdDwtc2NhbGUodHgpDQptZWFuPC1tZWFuKHR4KQ0Kc2Q8LXNkKHR4KQ0KQ29uZGlzdF9hZ2VfbGFtYmRhPC1hdHRyKHR4LCJsYW1iZGEiKSMjMC4xNQ0KQ29uZGlzdF9hZ2VfYWRkZWQ8LWF0dHIodHgsImFkZGVkIikjIzANCigoKDE2K0NvbmRpc3RfYWdlX2FkZGVkKV5Db25kaXN0X2FnZV9sYW1iZGEtMSkvQ29uZGlzdF9hZ2VfbGFtYmRhLW1lYW4pL3NkIyMtMi4wMzc4NzkNCigoKDUwK0NvbmRpc3RfYWdlX2FkZGVkKV5Db25kaXN0X2FnZV9sYW1iZGEtMSkvQ29uZGlzdF9hZ2VfbGFtYmRhLW1lYW4pL3NkIyMtMC43MDc1NDczDQooKCgxNTArQ29uZGlzdF9hZ2VfYWRkZWQpXkNvbmRpc3RfYWdlX2xhbWJkYS0xKS9Db25kaXN0X2FnZV9sYW1iZGEtbWVhbikvc2QjIzAuODA5NDE4OA0KKCgoMjUwK0NvbmRpc3RfYWdlX2FkZGVkKV5Db25kaXN0X2FnZV9sYW1iZGEtMSkvQ29uZGlzdF9hZ2VfbGFtYmRhLW1lYW4pL3NkIyMxLjYwNDU1NQ0KKCgoMzUwK0NvbmRpc3RfYWdlX2FkZGVkKV5Db25kaXN0X2FnZV9sYW1iZGEtMSkvQ29uZGlzdF9hZ2VfbGFtYmRhLW1lYW4pL3NkIyMyLjE2MjU4NA0KDQoNCkNvbi5EaXM8LUNvbi5kaXN0WyxjKDE6NSw4OjksMTMsMTY6MjEpXQ0Kc2F2ZShmaWxlPSJDb25fRGlzLlJEYXRhIixsaXN0PSJDb24uRGlzIikNCmBgYA0KDQoNCg==
