# Supplementary material for: Limited effects of population age on the genetic structure of spatially isolated forest herb populations in temperate Europe
Source: Ecol Evol. 2024 Feb 26;14(2):e10971. doi: 10.1002/ece3.10971 (PMC10897356; doi:10.1002/ece3.10971)
Supplement: Supplementary file 1 — Appendix S1. [file ECE3-14-e10971-s001.zip › 05a_Population_Genetic_Diversity_and_Differentiation_RawData_Correlation_check.nb.html]

Population Genetic Diversity and Differentiation raw data & correlation


Code 

- Show All Code
- Hide All Code
- Download Rmd

# Population Genetic Diversity and Differentiation raw data & correlation


```
library("dplyr")
load("GenDiv_all.RData")
load("dist.RData")
load("CD.RData")
```


```
GenDiv_all%>%
  group_by(Species.x,LW.x)%>%
  summarise(meanAr=mean(Ar),sdAr=sd(Ar),meanHe=mean(He),sdHe=sd(He),meanHo=mean(Ho),sdHo=sd(Ho),meanFis=mean(Fis),sdFis=sd(Fis))
GenDiv_all%>%
  group_by(Species.x)%>%
  summarise(meanAr=mean(Ar),sdAr=sd(Ar),meanHe=mean(He),sdHe=sd(He),meanHo=mean(Ho),sdHo=sd(Ho),meanFis=mean(Fis),sdFis=sd(Fis))

dist%>%
  group_by(Species.x,LW)%>%
  summarise(meanGst=mean(Gst),sdGst=sd(Gst),meanDps=mean(Dps),sdDps=sd(Dps))
dist%>%
  group_by(Species.x)%>%
  summarise(meanGst=mean(Gst),sdGst=sd(Gst),meanDps=mean(Dps),sdDps=sd(Dps))
CD$LW<-unlist(lapply(strsplit(CD$IN_POP,"_"), function(x) (x[2])))
str(CD)

CD%>%
  group_by(Species,LW)%>%
  summarise(meancgd=mean(Con.Dis))
```


```
#test for correlation
subane<-subset(GenDiv_all,GenDiv_all$Species.x=="Ane")
with(subane,cor.test(PopSize_transformed,IFM_transformed)) ##p<0.01, r=0.76
with(subane,cor.test(PopSize_transformed,Age_abs)) #p<0.01, r=0.52
with(subane,cor.test(IFM_transformed,Age_abs))##p<0.01 r=0.38

suboxa<-subset(GenDiv_all,GenDiv_all$Species.x=="Oxa")
with(suboxa,cor.test(PopSize_transformed,IFM_transformed)) ##p<0.01, r=0.63
with(suboxa,cor.test(PopSize_transformed,Age_abs)) #p<0.01, r=0.46
with(suboxa,cor.test(IFM_transformed,Age_abs))##p<0.01 r=0.50

subpol<-subset(GenDiv_all,GenDiv_all$Species.x=="Pol")
with(subpol,cor.test(PopSize_transformed,IFM_transformed)) ##p<0.01, r=0.68
with(subpol,cor.test(PopSize_transformed,Age_abs)) #n.s. r=0.11
with(subpol,cor.test(IFM_transformed,Age_abs))##n.s. r=0.09

distane<-subset(dist,dist$Species.x=="Ane")
with(distane,cor.test(Distance,Age_Diff)) #r=0.04, p=0.48
with(distane,cor.test(Distance,Age_abs_y)) #r=-0.16, p=<0.01
with(distane,cor.test(Age_Diff,Age_abs_y))# r=-0.50, p<0.01

distoxa<-subset(dist,dist$Species.x=="Oxa")
with(distoxa,cor.test(Distance,Age_Diff)) #r=-0.04, p=0.64
with(distoxa,cor.test(Distance,Age_abs_y)) #r=-0.07, p=0.35
with(distoxa,cor.test(Age_Diff,Age_abs_y))#r=-0.58, p<0.01

distpol<-subset(dist,dist$Species.x=="Pol")
with(distpol,cor.test(Distance,Age_Diff)) #r=-0.05, p=0.41
with(distpol,cor.test(Distance,Age_abs_y)) #r=-0.02, p=0.80
with(distpol,cor.test(Age_Diff,Age_abs_y)) #r=-0.52, p<0.01
```


```
data_ane<-GenDiv_all[GenDiv_all$Species.x=="Ane",]
lm_ane<-lm(log(PopSize)~Age_abs,data=data_ane)
Age_abs<-seq(min(data_ane$Age_abs), max(data_ane$Age_abs), length.out=100)
predict<-predict(lm_ane,newdata = data.frame(x=Age_abs),interval='confidence')
predict_ane<-data.frame(Age_abs,as.data.frame(predict))


data_oxa<-GenDiv_all[GenDiv_all$Species.x=="Oxa",]
lm_oxa<-lm(log(PopSize)~Age_abs,data=data_oxa)
Age_abs<-seq(min(data_oxa$Age_abs),max(data_oxa$Age_abs),length.out=100)
predict<-predict(lm_oxa,newdata=data.frame(x=Age_abs),interval='confidence')
predict_oxa<-data.frame(Age_abs,as.data.frame(predict))

par(mfrow=c(1,3), mar=c(3.5,3.5,1.5,0.8)+0.1, mgp=c(1.9, 0.5, 0), tcl=-0.3,  cex=1)

colors <- c("#bb5566","#ddaa33","#004488")

## PopSize & Age
base::plot(x = GenDiv_all$Age_abs, 
           y = log(GenDiv_all$PopSize),
           xlab=c("POP_AGE (year)"),
           ylab=c("POP_SIZE"),
           yaxt='n',xaxt='n',
           bg = rep(colors,table(GenDiv_all$Species.x)),
           cex = 1.5,cex.lab=1.5,
           pch=21
)

axis(side=2,lwd=2,cex.axis=1.5,at=c(log(1e+01),log(1e+03),log(1e+05),log(1e+07)),labels = c("1e+01","1e+03","1e+05","1e+07"))
axis(side=1,lwd=2,cex.axis=1.5)

box(lwd=2)
with(data_ane, abline(lm(log(PopSize)~Age_abs),col=c("#bb5566"),lwd=2))
with(predict_ane, polygon(x=c(Age_abs,rev(Age_abs)),y=c(lwr,rev(upr)),col=alpha("#bb5566",0.3),border=NA))


with(GenDiv_all[GenDiv_all$Species.x=="Oxa",], abline(lm(log(PopSize)~Age_abs),col=c("#ddaa33"),lwd=2))
with(predict_oxa, polygon(x=c(Age_abs,rev(Age_abs)),y=c(lwr,rev(upr)),col=alpha("#ddaa33",0.3),border=NA))

with(GenDiv_all[GenDiv_all$Species.x=="Pol",], abline(lm(log(PopSize)~Age_abs),col=c("#004488"),lwd=2,lty=5))
mtext(adj=0,"(A)",cex=1.5)

lm_ane<-lm(IFM~Age_abs,data=data_ane)
Age_abs<-seq(min(data_ane$Age_abs), max(data_ane$Age_abs), length.out=100)
predict<-predict(lm_ane,newdata = data.frame(x=Age_abs),interval='confidence')
predict_ane<-data.frame(Age_abs,as.data.frame(predict))


lm_oxa<-lm(IFM~Age_abs,data=data_oxa)
Age_abs<-seq(min(data_oxa$Age_abs),max(data_oxa$Age_abs),length.out=100)
predict<-predict(lm_oxa,newdata=data.frame(x=Age_abs),interval='confidence')
predict_oxa<-data.frame(Age_abs,as.data.frame(predict))
plot(x = GenDiv_all$Age_abs, 
     y = GenDiv_all$IFM,
     xlab=c("POP_AGE (year)"),
     ylab=c("SPA_CON"),
     yaxt='n',xaxt='n',
     bg = rep(colors,table(GenDiv_all$Species.x)),
     cex = 1.5,cex.lab=1.5,
     pch=21
)
axis(side=2,lwd=2,cex.axis=1.5)
axis(side=1,lwd=2,cex.axis=1.5)
box(lwd=2)
with(GenDiv_all[GenDiv_all$Species.x=="Ane",], abline(lm(IFM~Age_abs),col=c("#bb5566"),lwd=2))
with(predict_ane, polygon(x=c(Age_abs,rev(Age_abs)),y=c(lwr,rev(upr)),col=alpha("#bb5566",0.3),border=NA))

with(GenDiv_all[GenDiv_all$Species.x=="Oxa",], abline(lm(IFM~Age_abs),col=c("#ddaa33"),lwd=2))
with(predict_oxa, polygon(x=c(Age_abs,rev(Age_abs)),y=c(lwr,rev(upr)),col=alpha("#ddaa33",0.3),border=NA))

with(GenDiv_all[GenDiv_all$Species.x=="Pol",], abline(lm(IFM~Age_abs),col=c("#004488"),lwd=2,lty=5))
mtext(adj=0,"(B)",cex=1.5)

plot(x=jitter(dist$Age_Diff,30),
     y=dist$Distance,
     xlab=c("AGE_DIFF (year)"),
     ylab=c("GEO_DIST (m)"),
     yaxt='n',xaxt='n',
     bg=rep(colors,table(GenDiv_all$Species.x)),
     cex=1.5,cex.lab=1.5,
     pch=21)
axis(side=2,lwd=2,cex.axis=1.5)
axis(side=1,lwd=2,cex.axis=1.5)
box(lwd=2)
with(dist[dist$Species.x=="Ane",], abline(lm(Distance~Age_Diff),col=c("#bb5566"),lwd=2,lty=5))
with(dist[dist$Species.x=="Oxa",], abline(lm(Distance~Age_Diff),col=c("#ddaa33"),lwd=2,lty=5))
with(dist[dist$Species.x=="Pol",], abline(lm(Distance~Age_Diff),col=c("#004488"),lwd=2,lty=5))
mtext(adj=0,"(C)",cex=1.5)
graphics::legend(140,7400,legend=expression(italic(A.nem.),italic(O.ace.),italic(P.mul.),"p > 0.05","p"<="0.05"),bty='n',
                 pch=c(21,21,21,NA,NA),pt.bg=c("#bb5566","#ddaa33","#004488",NA,NA),lty=c(NA,NA,NA,5,1),
                 seg.len=0.5,cex=1.2,x.intersp=0.5,y.intersp=0.5)
```


LS0tDQp0aXRsZTogIlBvcHVsYXRpb24gR2VuZXRpYyBEaXZlcnNpdHkgYW5kIERpZmZlcmVudGlhdGlvbiByYXcgZGF0YSAmIGNvcnJlbGF0aW9uIg0Kb3V0cHV0OiBodG1sX25vdGVib29rDQotLS0NCg0KYGBge3J9DQpsaWJyYXJ5KCJkcGx5ciIpDQpsb2FkKCJHZW5EaXZfYWxsLlJEYXRhIikNCmxvYWQoImRpc3QuUkRhdGEiKQ0KbG9hZCgiQ0QuUkRhdGEiKQ0KYGBgDQoNCg0KYGBge3J9DQpHZW5EaXZfYWxsJT4lDQogIGdyb3VwX2J5KFNwZWNpZXMueCxMVy54KSU+JQ0KICBzdW1tYXJpc2UobWVhbkFyPW1lYW4oQXIpLHNkQXI9c2QoQXIpLG1lYW5IZT1tZWFuKEhlKSxzZEhlPXNkKEhlKSxtZWFuSG89bWVhbihIbyksc2RIbz1zZChIbyksbWVhbkZpcz1tZWFuKEZpcyksc2RGaXM9c2QoRmlzKSkNCkdlbkRpdl9hbGwlPiUNCiAgZ3JvdXBfYnkoU3BlY2llcy54KSU+JQ0KICBzdW1tYXJpc2UobWVhbkFyPW1lYW4oQXIpLHNkQXI9c2QoQXIpLG1lYW5IZT1tZWFuKEhlKSxzZEhlPXNkKEhlKSxtZWFuSG89bWVhbihIbyksc2RIbz1zZChIbyksbWVhbkZpcz1tZWFuKEZpcyksc2RGaXM9c2QoRmlzKSkNCg0KZGlzdCU+JQ0KICBncm91cF9ieShTcGVjaWVzLngsTFcpJT4lDQogIHN1bW1hcmlzZShtZWFuR3N0PW1lYW4oR3N0KSxzZEdzdD1zZChHc3QpLG1lYW5EcHM9bWVhbihEcHMpLHNkRHBzPXNkKERwcykpDQpkaXN0JT4lDQogIGdyb3VwX2J5KFNwZWNpZXMueCklPiUNCiAgc3VtbWFyaXNlKG1lYW5Hc3Q9bWVhbihHc3QpLHNkR3N0PXNkKEdzdCksbWVhbkRwcz1tZWFuKERwcyksc2REcHM9c2QoRHBzKSkNCkNEJExXPC11bmxpc3QobGFwcGx5KHN0cnNwbGl0KENEJElOX1BPUCwiXyIpLCBmdW5jdGlvbih4KSAoeFsyXSkpKQ0Kc3RyKENEKQ0KDQpDRCU+JQ0KICBncm91cF9ieShTcGVjaWVzLExXKSU+JQ0KICBzdW1tYXJpc2UobWVhbmNnZD1tZWFuKENvbi5EaXMpKQ0KYGBgDQpgYGB7cn0NCiN0ZXN0IGZvciBjb3JyZWxhdGlvbg0Kc3ViYW5lPC1zdWJzZXQoR2VuRGl2X2FsbCxHZW5EaXZfYWxsJFNwZWNpZXMueD09IkFuZSIpDQp3aXRoKHN1YmFuZSxjb3IudGVzdChQb3BTaXplX3RyYW5zZm9ybWVkLElGTV90cmFuc2Zvcm1lZCkpICMjcDwwLjAxLCByPTAuNzYNCndpdGgoc3ViYW5lLGNvci50ZXN0KFBvcFNpemVfdHJhbnNmb3JtZWQsQWdlX2FicykpICNwPDAuMDEsIHI9MC41Mg0Kd2l0aChzdWJhbmUsY29yLnRlc3QoSUZNX3RyYW5zZm9ybWVkLEFnZV9hYnMpKSMjcDwwLjAxIHI9MC4zOA0KDQpzdWJveGE8LXN1YnNldChHZW5EaXZfYWxsLEdlbkRpdl9hbGwkU3BlY2llcy54PT0iT3hhIikNCndpdGgoc3Vib3hhLGNvci50ZXN0KFBvcFNpemVfdHJhbnNmb3JtZWQsSUZNX3RyYW5zZm9ybWVkKSkgIyNwPDAuMDEsIHI9MC42Mw0Kd2l0aChzdWJveGEsY29yLnRlc3QoUG9wU2l6ZV90cmFuc2Zvcm1lZCxBZ2VfYWJzKSkgI3A8MC4wMSwgcj0wLjQ2DQp3aXRoKHN1Ym94YSxjb3IudGVzdChJRk1fdHJhbnNmb3JtZWQsQWdlX2FicykpIyNwPDAuMDEgcj0wLjUwDQoNCnN1YnBvbDwtc3Vic2V0KEdlbkRpdl9hbGwsR2VuRGl2X2FsbCRTcGVjaWVzLng9PSJQb2wiKQ0Kd2l0aChzdWJwb2wsY29yLnRlc3QoUG9wU2l6ZV90cmFuc2Zvcm1lZCxJRk1fdHJhbnNmb3JtZWQpKSAjI3A8MC4wMSwgcj0wLjY4DQp3aXRoKHN1YnBvbCxjb3IudGVzdChQb3BTaXplX3RyYW5zZm9ybWVkLEFnZV9hYnMpKSAjbi5zLiByPTAuMTENCndpdGgoc3VicG9sLGNvci50ZXN0KElGTV90cmFuc2Zvcm1lZCxBZ2VfYWJzKSkjI24ucy4gcj0wLjA5DQoNCmRpc3RhbmU8LXN1YnNldChkaXN0LGRpc3QkU3BlY2llcy54PT0iQW5lIikNCndpdGgoZGlzdGFuZSxjb3IudGVzdChEaXN0YW5jZSxBZ2VfRGlmZikpICNyPTAuMDQsIHA9MC40OA0Kd2l0aChkaXN0YW5lLGNvci50ZXN0KERpc3RhbmNlLEFnZV9hYnNfeSkpICNyPS0wLjE2LCBwPTwwLjAxDQp3aXRoKGRpc3RhbmUsY29yLnRlc3QoQWdlX0RpZmYsQWdlX2Fic195KSkjIHI9LTAuNTAsIHA8MC4wMQ0KDQpkaXN0b3hhPC1zdWJzZXQoZGlzdCxkaXN0JFNwZWNpZXMueD09Ik94YSIpDQp3aXRoKGRpc3RveGEsY29yLnRlc3QoRGlzdGFuY2UsQWdlX0RpZmYpKSAjcj0tMC4wNCwgcD0wLjY0DQp3aXRoKGRpc3RveGEsY29yLnRlc3QoRGlzdGFuY2UsQWdlX2Fic195KSkgI3I9LTAuMDcsIHA9MC4zNQ0Kd2l0aChkaXN0b3hhLGNvci50ZXN0KEFnZV9EaWZmLEFnZV9hYnNfeSkpI3I9LTAuNTgsIHA8MC4wMQ0KDQpkaXN0cG9sPC1zdWJzZXQoZGlzdCxkaXN0JFNwZWNpZXMueD09IlBvbCIpDQp3aXRoKGRpc3Rwb2wsY29yLnRlc3QoRGlzdGFuY2UsQWdlX0RpZmYpKSAjcj0tMC4wNSwgcD0wLjQxDQp3aXRoKGRpc3Rwb2wsY29yLnRlc3QoRGlzdGFuY2UsQWdlX2Fic195KSkgI3I9LTAuMDIsIHA9MC44MA0Kd2l0aChkaXN0cG9sLGNvci50ZXN0KEFnZV9EaWZmLEFnZV9hYnNfeSkpICNyPS0wLjUyLCBwPDAuMDENCg0KYGBgDQoNCmBgYHtyfQ0KDQpkYXRhX2FuZTwtR2VuRGl2X2FsbFtHZW5EaXZfYWxsJFNwZWNpZXMueD09IkFuZSIsXQ0KbG1fYW5lPC1sbShsb2coUG9wU2l6ZSl+QWdlX2FicyxkYXRhPWRhdGFfYW5lKQ0KQWdlX2Ficzwtc2VxKG1pbihkYXRhX2FuZSRBZ2VfYWJzKSwgbWF4KGRhdGFfYW5lJEFnZV9hYnMpLCBsZW5ndGgub3V0PTEwMCkNCnByZWRpY3Q8LXByZWRpY3QobG1fYW5lLG5ld2RhdGEgPSBkYXRhLmZyYW1lKHg9QWdlX2FicyksaW50ZXJ2YWw9J2NvbmZpZGVuY2UnKQ0KcHJlZGljdF9hbmU8LWRhdGEuZnJhbWUoQWdlX2Ficyxhcy5kYXRhLmZyYW1lKHByZWRpY3QpKQ0KDQoNCmRhdGFfb3hhPC1HZW5EaXZfYWxsW0dlbkRpdl9hbGwkU3BlY2llcy54PT0iT3hhIixdDQpsbV9veGE8LWxtKGxvZyhQb3BTaXplKX5BZ2VfYWJzLGRhdGE9ZGF0YV9veGEpDQpBZ2VfYWJzPC1zZXEobWluKGRhdGFfb3hhJEFnZV9hYnMpLG1heChkYXRhX294YSRBZ2VfYWJzKSxsZW5ndGgub3V0PTEwMCkNCnByZWRpY3Q8LXByZWRpY3QobG1fb3hhLG5ld2RhdGE9ZGF0YS5mcmFtZSh4PUFnZV9hYnMpLGludGVydmFsPSdjb25maWRlbmNlJykNCnByZWRpY3Rfb3hhPC1kYXRhLmZyYW1lKEFnZV9hYnMsYXMuZGF0YS5mcmFtZShwcmVkaWN0KSkNCg0KcGFyKG1mcm93PWMoMSwzKSwgbWFyPWMoMy41LDMuNSwxLjUsMC44KSswLjEsIG1ncD1jKDEuOSwgMC41LCAwKSwgdGNsPS0wLjMsICBjZXg9MSkNCg0KY29sb3JzIDwtIGMoIiNiYjU1NjYiLCIjZGRhYTMzIiwiIzAwNDQ4OCIpDQoNCiMjIFBvcFNpemUgJiBBZ2UNCmJhc2U6OnBsb3QoeCA9IEdlbkRpdl9hbGwkQWdlX2FicywgDQogICAgICAgICAgIHkgPSBsb2coR2VuRGl2X2FsbCRQb3BTaXplKSwNCiAgICAgICAgICAgeGxhYj1jKCJQT1BfQUdFICh5ZWFyKSIpLA0KICAgICAgICAgICB5bGFiPWMoIlBPUF9TSVpFIiksDQogICAgICAgICAgIHlheHQ9J24nLHhheHQ9J24nLA0KICAgICAgICAgICBiZyA9IHJlcChjb2xvcnMsdGFibGUoR2VuRGl2X2FsbCRTcGVjaWVzLngpKSwNCiAgICAgICAgICAgY2V4ID0gMS41LGNleC5sYWI9MS41LA0KICAgICAgICAgICBwY2g9MjENCikNCg0KYXhpcyhzaWRlPTIsbHdkPTIsY2V4LmF4aXM9MS41LGF0PWMobG9nKDFlKzAxKSxsb2coMWUrMDMpLGxvZygxZSswNSksbG9nKDFlKzA3KSksbGFiZWxzID0gYygiMWUrMDEiLCIxZSswMyIsIjFlKzA1IiwiMWUrMDciKSkNCmF4aXMoc2lkZT0xLGx3ZD0yLGNleC5heGlzPTEuNSkNCg0KYm94KGx3ZD0yKQ0Kd2l0aChkYXRhX2FuZSwgYWJsaW5lKGxtKGxvZyhQb3BTaXplKX5BZ2VfYWJzKSxjb2w9YygiI2JiNTU2NiIpLGx3ZD0yKSkNCndpdGgocHJlZGljdF9hbmUsIHBvbHlnb24oeD1jKEFnZV9hYnMscmV2KEFnZV9hYnMpKSx5PWMobHdyLHJldih1cHIpKSxjb2w9YWxwaGEoIiNiYjU1NjYiLDAuMyksYm9yZGVyPU5BKSkNCg0KDQp3aXRoKEdlbkRpdl9hbGxbR2VuRGl2X2FsbCRTcGVjaWVzLng9PSJPeGEiLF0sIGFibGluZShsbShsb2coUG9wU2l6ZSl+QWdlX2FicyksY29sPWMoIiNkZGFhMzMiKSxsd2Q9MikpDQp3aXRoKHByZWRpY3Rfb3hhLCBwb2x5Z29uKHg9YyhBZ2VfYWJzLHJldihBZ2VfYWJzKSkseT1jKGx3cixyZXYodXByKSksY29sPWFscGhhKCIjZGRhYTMzIiwwLjMpLGJvcmRlcj1OQSkpDQoNCndpdGgoR2VuRGl2X2FsbFtHZW5EaXZfYWxsJFNwZWNpZXMueD09IlBvbCIsXSwgYWJsaW5lKGxtKGxvZyhQb3BTaXplKX5BZ2VfYWJzKSxjb2w9YygiIzAwNDQ4OCIpLGx3ZD0yLGx0eT01KSkNCm10ZXh0KGFkaj0wLCIoQSkiLGNleD0xLjUpDQoNCmxtX2FuZTwtbG0oSUZNfkFnZV9hYnMsZGF0YT1kYXRhX2FuZSkNCkFnZV9hYnM8LXNlcShtaW4oZGF0YV9hbmUkQWdlX2FicyksIG1heChkYXRhX2FuZSRBZ2VfYWJzKSwgbGVuZ3RoLm91dD0xMDApDQpwcmVkaWN0PC1wcmVkaWN0KGxtX2FuZSxuZXdkYXRhID0gZGF0YS5mcmFtZSh4PUFnZV9hYnMpLGludGVydmFsPSdjb25maWRlbmNlJykNCnByZWRpY3RfYW5lPC1kYXRhLmZyYW1lKEFnZV9hYnMsYXMuZGF0YS5mcmFtZShwcmVkaWN0KSkNCg0KDQoNCmxtX294YTwtbG0oSUZNfkFnZV9hYnMsZGF0YT1kYXRhX294YSkNCkFnZV9hYnM8LXNlcShtaW4oZGF0YV9veGEkQWdlX2FicyksbWF4KGRhdGFfb3hhJEFnZV9hYnMpLGxlbmd0aC5vdXQ9MTAwKQ0KcHJlZGljdDwtcHJlZGljdChsbV9veGEsbmV3ZGF0YT1kYXRhLmZyYW1lKHg9QWdlX2FicyksaW50ZXJ2YWw9J2NvbmZpZGVuY2UnKQ0KcHJlZGljdF9veGE8LWRhdGEuZnJhbWUoQWdlX2Ficyxhcy5kYXRhLmZyYW1lKHByZWRpY3QpKQ0KcGxvdCh4ID0gR2VuRGl2X2FsbCRBZ2VfYWJzLCANCiAgICAgeSA9IEdlbkRpdl9hbGwkSUZNLA0KICAgICB4bGFiPWMoIlBPUF9BR0UgKHllYXIpIiksDQogICAgIHlsYWI9YygiU1BBX0NPTiIpLA0KICAgICB5YXh0PSduJyx4YXh0PSduJywNCiAgICAgYmcgPSByZXAoY29sb3JzLHRhYmxlKEdlbkRpdl9hbGwkU3BlY2llcy54KSksDQogICAgIGNleCA9IDEuNSxjZXgubGFiPTEuNSwNCiAgICAgcGNoPTIxDQopDQpheGlzKHNpZGU9Mixsd2Q9MixjZXguYXhpcz0xLjUpDQpheGlzKHNpZGU9MSxsd2Q9MixjZXguYXhpcz0xLjUpDQpib3gobHdkPTIpDQp3aXRoKEdlbkRpdl9hbGxbR2VuRGl2X2FsbCRTcGVjaWVzLng9PSJBbmUiLF0sIGFibGluZShsbShJRk1+QWdlX2FicyksY29sPWMoIiNiYjU1NjYiKSxsd2Q9MikpDQp3aXRoKHByZWRpY3RfYW5lLCBwb2x5Z29uKHg9YyhBZ2VfYWJzLHJldihBZ2VfYWJzKSkseT1jKGx3cixyZXYodXByKSksY29sPWFscGhhKCIjYmI1NTY2IiwwLjMpLGJvcmRlcj1OQSkpDQoNCndpdGgoR2VuRGl2X2FsbFtHZW5EaXZfYWxsJFNwZWNpZXMueD09Ik94YSIsXSwgYWJsaW5lKGxtKElGTX5BZ2VfYWJzKSxjb2w9YygiI2RkYWEzMyIpLGx3ZD0yKSkNCndpdGgocHJlZGljdF9veGEsIHBvbHlnb24oeD1jKEFnZV9hYnMscmV2KEFnZV9hYnMpKSx5PWMobHdyLHJldih1cHIpKSxjb2w9YWxwaGEoIiNkZGFhMzMiLDAuMyksYm9yZGVyPU5BKSkNCg0Kd2l0aChHZW5EaXZfYWxsW0dlbkRpdl9hbGwkU3BlY2llcy54PT0iUG9sIixdLCBhYmxpbmUobG0oSUZNfkFnZV9hYnMpLGNvbD1jKCIjMDA0NDg4IiksbHdkPTIsbHR5PTUpKQ0KbXRleHQoYWRqPTAsIihCKSIsY2V4PTEuNSkNCg0KcGxvdCh4PWppdHRlcihkaXN0JEFnZV9EaWZmLDMwKSwNCiAgICAgeT1kaXN0JERpc3RhbmNlLA0KICAgICB4bGFiPWMoIkFHRV9ESUZGICh5ZWFyKSIpLA0KICAgICB5bGFiPWMoIkdFT19ESVNUIChtKSIpLA0KICAgICB5YXh0PSduJyx4YXh0PSduJywNCiAgICAgYmc9cmVwKGNvbG9ycyx0YWJsZShHZW5EaXZfYWxsJFNwZWNpZXMueCkpLA0KICAgICBjZXg9MS41LGNleC5sYWI9MS41LA0KICAgICBwY2g9MjEpDQpheGlzKHNpZGU9Mixsd2Q9MixjZXguYXhpcz0xLjUpDQpheGlzKHNpZGU9MSxsd2Q9MixjZXguYXhpcz0xLjUpDQpib3gobHdkPTIpDQp3aXRoKGRpc3RbZGlzdCRTcGVjaWVzLng9PSJBbmUiLF0sIGFibGluZShsbShEaXN0YW5jZX5BZ2VfRGlmZiksY29sPWMoIiNiYjU1NjYiKSxsd2Q9MixsdHk9NSkpDQp3aXRoKGRpc3RbZGlzdCRTcGVjaWVzLng9PSJPeGEiLF0sIGFibGluZShsbShEaXN0YW5jZX5BZ2VfRGlmZiksY29sPWMoIiNkZGFhMzMiKSxsd2Q9MixsdHk9NSkpDQp3aXRoKGRpc3RbZGlzdCRTcGVjaWVzLng9PSJQb2wiLF0sIGFibGluZShsbShEaXN0YW5jZX5BZ2VfRGlmZiksY29sPWMoIiMwMDQ0ODgiKSxsd2Q9MixsdHk9NSkpDQptdGV4dChhZGo9MCwiKEMpIixjZXg9MS41KQ0KZ3JhcGhpY3M6OmxlZ2VuZCgxNDAsNzQwMCxsZWdlbmQ9ZXhwcmVzc2lvbihpdGFsaWMoQS5uZW0uKSxpdGFsaWMoTy5hY2UuKSxpdGFsaWMoUC5tdWwuKSwicCA+IDAuMDUiLCJwIjw9IjAuMDUiKSxidHk9J24nLA0KICAgICAgICAgICAgICAgICBwY2g9YygyMSwyMSwyMSxOQSxOQSkscHQuYmc9YygiI2JiNTU2NiIsIiNkZGFhMzMiLCIjMDA0NDg4IixOQSxOQSksbHR5PWMoTkEsTkEsTkEsNSwxKSwNCiAgICAgICAgICAgICAgICAgc2VnLmxlbj0wLjUsY2V4PTEuMix4LmludGVyc3A9MC41LHkuaW50ZXJzcD0wLjUpDQpgYGANCg0K
