# Supplementary material for: Limited effects of population age on the genetic structure of spatially isolated forest herb populations in temperate Europe
Source: Ecol Evol. 2024 Feb 26;14(2):e10971. doi: 10.1002/ece3.10971 (PMC10897356; doi:10.1002/ece3.10971)
Supplement: Supplementary file 1 — Appendix S1. [file ECE3-14-e10971-s001.zip › 06_Linear_Regression.nb.html]

Linear model for genetic diversity and genetic differentiation as well as population graph


Code 

- Show All Code
- Hide All Code
- Download Rmd

# Linear model for genetic diversity and genetic differentiation as well as population graph

Genetic Diversity


```
library(openxlsx)
library(nlme)
library(dplyr)
library(psych)
library(MuMIn)
library(car)
library(MASS)
library(tidyverse)
library(corMLPE)
source("L:/05_Data analysis/boxcox.r")
load("GenDiv_all.RData")
GenDiv_all$Species.x<-factor(GenDiv_all$Species.x,ordered=F)
GenDiv_all$Spec2<-relevel(GenDiv_all$Species.x,ref="Oxa")
GenDiv_all$Spec3<-relevel(GenDiv_all$Species.x,ref="Pol")

lmm.Ar.b<- lme(Ar_t~(PopSize_transformed+IFM_transformed+Age_t)*Species.x,random=~1|Region/Population,data=GenDiv_all)
dredge(lmm.Ar.b,fixed=~Species.x, REML=F)
lmm.Ar.b.fin<-lme(Ar_t~IFM_transformed+PopSize_transformed+Species.x*Age_t,random=~1|Region/Population,data=GenDiv_all)

plot(lmm.Ar.b.fin, form=resid(., type = "p") ~ fitted(.), abline=0) #check
qqnorm(lmm.Ar.b.fin,~resid(., type = "p"), abline=c(0,1))

summary(lmm.Ar.b.fin)

lmm.Ar.b.oxa.fin<-lme(Ar_t~IFM_transformed+PopSize_transformed+Spec2*Age_t,random=~1|Region/Population,data=GenDiv_all)
summary(lmm.Ar.b.oxa.fin)

lmm.Ar.b.pol.fin<-lme(Ar_t~IFM_transformed+PopSize_transformed+Spec3*Age_t,random=~1|Region/Population,data=GenDiv_all)
summary(lmm.Ar.b.pol.fin)

r.squaredGLMM(lmm.Ar.b.fin)
```


```
```r
## He~(POP_SIZE+SPA_CON+POP_AGE)*SPECIES
lmm.He.b<-lme(He_t~(PopSize_transformed+IFM_transformed+Age_t)*Species.x,random=~1|Region/Population,data=GenDiv_all)
dredge(lmm.He.b,fixed=~Species.x, REML=F)
lmm.He.b.fin<-lme(He_t~IFM_transformed+Age_t*Species.x,random=~1|Region/Population,data=GenDiv_all)
plot(lmm.He.b.fin,form=resid(., type = \p\) ~ fitted(.), abline=0)
qqnorm(lmm.He.b.fin,~resid(., type = \p\), abline=c(0,1))
summary(lmm.He.b.fin)

lmm.He.b.oxa.fin<-lme(He_t~IFM_transformed+Age_t*Spec2,random=~1|Region/Population,data=GenDiv_all)
summary(lmm.He.b.oxa.fin)

lmm.He.b.pol.fin<-lme(He_t~IFM_transformed+Age_t*Spec3,random=~1|Region/Population,data=GenDiv_all)
summary(lmm.He.b.pol.fin)

r.squaredGLMM(lmm.He.b.fin)
```

```
<!-- rnb-source-end -->

<!-- rnb-chunk-end -->


<!-- rnb-text-begin -->


<!-- rnb-text-end -->


<!-- rnb-chunk-begin -->


<!-- rnb-source-begin eyJkYXRhIjoiYGBgclxuYGBgclxuIyMgSG9+KFBPUF9TSVpFK1NQQV9DT04rUE9QX0FHRSkqU1BFQ0lFU1xubG1tLkhvLmI8LWxtZShIb190fihQb3BTaXplX3RyYW5zZm9ybWVkK0lGTV90cmFuc2Zvcm1lZCtBZ2VfdCkqU3BlY2llcy54LHJhbmRvbT1+MXxSZWdpb24vUG9wdWxhdGlvbixkYXRhPUdlbkRpdl9hbGwpXG5kcmVkZ2UobG1tLkhvLmIsZml4ZWQ9flNwZWNpZXMueCxSRU1MPUYpXG5sbW0uSG8uYi5maW48LWxtZShIb190fkFnZV90K0lGTV90cmFuc2Zvcm1lZCpTcGVjaWVzLngscmFuZG9tPX4xfFJlZ2lvbi9Qb3B1bGF0aW9uLGRhdGE9R2VuRGl2X2FsbClcbnBsb3QobG1tLkhvLmIuZmluLGZvcm09cmVzaWQoLiwgdHlwZSA9IFxccFxcKSB+IGZpdHRlZCguKSwgYWJsaW5lPTApXG5xcW5vcm0obG1tLkhvLmIuZmluLH5yZXNpZCguLCB0eXBlID0gXFxwXFwpLCBhYmxpbmU9YygwLDEpKVxuc3VtbWFyeShsbW0uSG8uYi5maW4pXG5cbmxtbS5Iby5iLm94YS5maW48LWxtZShIb190fkFnZV90K0lGTV90cmFuc2Zvcm1lZCpTcGVjMixyYW5kb209fjF8UmVnaW9uL1BvcHVsYXRpb24sZGF0YT1HZW5EaXZfYWxsKVxuc3VtbWFyeShsbW0uSG8uYi5veGEuZmluKVxuXG5sbW0uSG8uYi5wb2wuZmluPC1sbWUoSG9fdH5BZ2VfdCtJRk1fdHJhbnNmb3JtZWQqU3BlYzMscmFuZG9tPX4xfFJlZ2lvbi9Qb3B1bGF0aW9uLGRhdGE9R2VuRGl2X2FsbClcbnN1bW1hcnkobG1tLkhvLmIucG9sLmZpbilcblxuXG5yLnNxdWFyZWRHTE1NKGxtbS5Iby5iLmZpbilcblxuYGBgXG5gYGAifQ== -->

```r
```r
## Ho~(POP_SIZE+SPA_CON+POP_AGE)*SPECIES
lmm.Ho.b<-lme(Ho_t~(PopSize_transformed+IFM_transformed+Age_t)*Species.x,random=~1|Region/Population,data=GenDiv_all)
dredge(lmm.Ho.b,fixed=~Species.x,REML=F)
lmm.Ho.b.fin<-lme(Ho_t~Age_t+IFM_transformed*Species.x,random=~1|Region/Population,data=GenDiv_all)
plot(lmm.Ho.b.fin,form=resid(., type = \p\) ~ fitted(.), abline=0)
qqnorm(lmm.Ho.b.fin,~resid(., type = \p\), abline=c(0,1))
summary(lmm.Ho.b.fin)

lmm.Ho.b.oxa.fin<-lme(Ho_t~Age_t+IFM_transformed*Spec2,random=~1|Region/Population,data=GenDiv_all)
summary(lmm.Ho.b.oxa.fin)

lmm.Ho.b.pol.fin<-lme(Ho_t~Age_t+IFM_transformed*Spec3,random=~1|Region/Population,data=GenDiv_all)
summary(lmm.Ho.b.pol.fin)


r.squaredGLMM(lmm.Ho.b.fin)
```

```
<!-- rnb-source-end -->

<!-- rnb-chunk-end -->


<!-- rnb-text-begin -->


<!-- rnb-text-end -->


<!-- rnb-chunk-begin -->


<!-- rnb-source-begin eyJkYXRhIjoiYGBgclxuYGBgclxuIyMgRmlzfihQT1BfU0laRStTUEFfQ09OK1BPUF9BR0UpKlNQRUNJRVNcbmxtbS5GaXMuYjwtbG1lKEZpc190fihQb3BTaXplX3RyYW5zZm9ybWVkK0lGTV90cmFuc2Zvcm1lZCtBZ2VfdCkqU3BlY2llcy54LHJhbmRvbT1+MXxSZWdpb24vUG9wdWxhdGlvbixkYXRhPUdlbkRpdl9hbGwpXG5kcmVkZ2UobG1tLkZpcy5iLGZpeGVkPX5TcGVjaWVzLngsUkVNTD1GKVxubG1tLkZpcy5iLmZpbjwtbG1lKEZpc190fklGTV90cmFuc2Zvcm1lZCtBZ2VfdCtTcGVjaWVzLngscmFuZG9tPX4xfFJlZ2lvbi9Qb3B1bGF0aW9uLGRhdGE9R2VuRGl2X2FsbClcbnBsb3QobG1tLkZpcy5iLmZpbixmb3JtPXJlc2lkKC4sIHR5cGUgPSBcXHBcXCkgfiBmaXR0ZWQoLiksIGFibGluZT0wKVxucXFub3JtKGxtbS5GaXMuYi5maW4sfnJlc2lkKC4sIHR5cGUgPSBcXHBcXCksIGFibGluZT1jKDAsMSkpXG5zdW1tYXJ5KGxtbS5GaXMuYi5maW4pXG5yLnNxdWFyZWRHTE1NKGxtbS5GaXMuYi5maW4pXG5cbmBgYFxuYGBgIn0= -->

```r
```r
## Fis~(POP_SIZE+SPA_CON+POP_AGE)*SPECIES
lmm.Fis.b<-lme(Fis_t~(PopSize_transformed+IFM_transformed+Age_t)*Species.x,random=~1|Region/Population,data=GenDiv_all)
dredge(lmm.Fis.b,fixed=~Species.x,REML=F)
lmm.Fis.b.fin<-lme(Fis_t~IFM_transformed+Age_t+Species.x,random=~1|Region/Population,data=GenDiv_all)
plot(lmm.Fis.b.fin,form=resid(., type = \p\) ~ fitted(.), abline=0)
qqnorm(lmm.Fis.b.fin,~resid(., type = \p\), abline=c(0,1))
summary(lmm.Fis.b.fin)
r.squaredGLMM(lmm.Fis.b.fin)
```

```
<!-- rnb-source-end -->

<!-- rnb-chunk-end -->


<!-- rnb-text-begin -->


<!-- rnb-text-end -->


<!-- rnb-chunk-begin -->


<!-- rnb-source-begin eyJkYXRhIjoiYGBgclxuYGBgclxuIyMgSW50ZXJhY3Rpb24gYmV0d2VlbiBQT1BfQUdFIGFuZCBQT1BfU0laRS9TUEFfQ09OXG4jIyBBbmVtb25lIG5lbW9yb3NhXG5zdWJhbmU8LXN1YnNldChHZW5EaXZfYWxsLEdlbkRpdl9hbGwkU3BlY2llcy54PT1cXEFuZVxcKVxuc3ViYW5lJEFnZV90PC1zY2FsZShib3hjb3goc3ViYW5lJEFnZV9hYnMpKVxuXG5sbW0uQXIuYW5lPC1sbWUoQXJfdH4oUG9wU2l6ZV90cmFuc2Zvcm1lZCtJRk1fdHJhbnNmb3JtZWQpKkFnZV90LHJhbmRvbT1+MXxSZWdpb24sZGF0YT1zdWJhbmUpXG5zdW1tYXJ5KGxtbS5Bci5hbmUpXG5yLnNxdWFyZWRHTE1NKGxtbS5Bci5hbmUpXG5cbmxtbS5IZS5hbmU8LWxtZShIZV90fihQb3BTaXplX3RyYW5zZm9ybWVkK0lGTV90cmFuc2Zvcm1lZCkqQWdlX3QscmFuZG9tPX4xfFJlZ2lvbixkYXRhPXN1YmFuZSlcbnN1bW1hcnkobG1tLkhlLmFuZSlcbnIuc3F1YXJlZEdMTU0obG1tLkhlLmFuZSlcblxubG1tLkhvLmFuZTwtbG1lKEhvX3R+KFBvcFNpemVfdHJhbnNmb3JtZWQrSUZNX3RyYW5zZm9ybWVkKSpBZ2VfdCxyYW5kb209fjF8UmVnaW9uLGRhdGE9c3ViYW5lKVxuc3VtbWFyeShsbW0uSG8uYW5lKVxuci5zcXVhcmVkR0xNTShsbW0uSG8uYW5lKVxuXG5sbW0uRmlzLmFuZTwtbG1lKEZpc190fihQb3BTaXplX3RyYW5zZm9ybWVkK0lGTV90cmFuc2Zvcm1lZCkqQWdlX3QscmFuZG9tPX4xfFJlZ2lvbixkYXRhPXN1YmFuZSlcbnN1bW1hcnkobG1tLkZpcy5hbmUpXG5yLnNxdWFyZWRHTE1NKGxtbS5GaXMuYW5lKVxuXG5gYGBcbmBgYCJ9 -->

```r
```r
## Interaction between POP_AGE and POP_SIZE/SPA_CON
## Anemone nemorosa
subane<-subset(GenDiv_all,GenDiv_all$Species.x==\Ane\)
subane$Age_t<-scale(boxcox(subane$Age_abs))

lmm.Ar.ane<-lme(Ar_t~(PopSize_transformed+IFM_transformed)*Age_t,random=~1|Region,data=subane)
summary(lmm.Ar.ane)
r.squaredGLMM(lmm.Ar.ane)

lmm.He.ane<-lme(He_t~(PopSize_transformed+IFM_transformed)*Age_t,random=~1|Region,data=subane)
summary(lmm.He.ane)
r.squaredGLMM(lmm.He.ane)

lmm.Ho.ane<-lme(Ho_t~(PopSize_transformed+IFM_transformed)*Age_t,random=~1|Region,data=subane)
summary(lmm.Ho.ane)
r.squaredGLMM(lmm.Ho.ane)

lmm.Fis.ane<-lme(Fis_t~(PopSize_transformed+IFM_transformed)*Age_t,random=~1|Region,data=subane)
summary(lmm.Fis.ane)
r.squaredGLMM(lmm.Fis.ane)
```

```
<!-- rnb-source-end -->

<!-- rnb-chunk-end -->


<!-- rnb-text-begin -->


<!-- rnb-text-end -->


<!-- rnb-chunk-begin -->


<!-- rnb-source-begin eyJkYXRhIjoiYGBgclxuYGBgclxuIyMgT3hhbGlzIGFjZXRvc2VsbGFcbnN1Ym94YTwtc3Vic2V0KEdlbkRpdl9hbGwsR2VuRGl2X2FsbCRTcGVjaWVzLng9PVxcT3hhXFwpXG5zdWJveGEkQWdlX3Q8LXNjYWxlKGJveGNveChzdWJveGEkQWdlX2FicykpXG5cbmxtbS5Bci5veGE8LWxtZShBcl90fihQb3BTaXplX3RyYW5zZm9ybWVkK0lGTV90cmFuc2Zvcm1lZCkqQWdlX3QscmFuZG9tPX4xfFJlZ2lvbixkYXRhPXN1Ym94YSlcbnN1bW1hcnkobG1tLkFyLm94YSlcbnIuc3F1YXJlZEdMTU0obG1tLkFyLm94YSlcblxubG1tLkhlLm94YTwtbG1lKEhlX3R+KFBvcFNpemVfdHJhbnNmb3JtZWQrSUZNX3RyYW5zZm9ybWVkKSpBZ2VfdCxyYW5kb209fjF8UmVnaW9uLGRhdGE9c3Vib3hhKVxuc3VtbWFyeShsbW0uSGUub3hhKVxuci5zcXVhcmVkR0xNTShsbW0uSGUub3hhKVxuXG5sbW0uSG8ub3hhPC1sbWUoSG9fdH4oUG9wU2l6ZV90cmFuc2Zvcm1lZCtJRk1fdHJhbnNmb3JtZWQpKkFnZV90LHJhbmRvbT1+MXxSZWdpb24sZGF0YT1zdWJveGEpXG5zdW1tYXJ5KGxtbS5Iby5veGEpXG5yLnNxdWFyZWRHTE1NKGxtbS5Iby5veGEpXG5cbmxtbS5GaXMub3hhPC1sbWUoRmlzX3R+KFBvcFNpemVfdHJhbnNmb3JtZWQrSUZNX3RyYW5zZm9ybWVkKSpBZ2VfdCxyYW5kb209fjF8UmVnaW9uLGRhdGE9c3Vib3hhKVxuc3VtbWFyeShsbW0uRmlzLm94YSlcbnIuc3F1YXJlZEdMTU0obG1tLkZpcy5veGEpXG5cbmBgYFxuYGBgIn0= -->

```r
```r
## Oxalis acetosella
suboxa<-subset(GenDiv_all,GenDiv_all$Species.x==\Oxa\)
suboxa$Age_t<-scale(boxcox(suboxa$Age_abs))

lmm.Ar.oxa<-lme(Ar_t~(PopSize_transformed+IFM_transformed)*Age_t,random=~1|Region,data=suboxa)
summary(lmm.Ar.oxa)
r.squaredGLMM(lmm.Ar.oxa)

lmm.He.oxa<-lme(He_t~(PopSize_transformed+IFM_transformed)*Age_t,random=~1|Region,data=suboxa)
summary(lmm.He.oxa)
r.squaredGLMM(lmm.He.oxa)

lmm.Ho.oxa<-lme(Ho_t~(PopSize_transformed+IFM_transformed)*Age_t,random=~1|Region,data=suboxa)
summary(lmm.Ho.oxa)
r.squaredGLMM(lmm.Ho.oxa)

lmm.Fis.oxa<-lme(Fis_t~(PopSize_transformed+IFM_transformed)*Age_t,random=~1|Region,data=suboxa)
summary(lmm.Fis.oxa)
r.squaredGLMM(lmm.Fis.oxa)
```

```
<!-- rnb-source-end -->

<!-- rnb-chunk-end -->


<!-- rnb-text-begin -->


<!-- rnb-text-end -->


<!-- rnb-chunk-begin -->


<!-- rnb-source-begin eyJkYXRhIjoiYGBgclxuYGBgclxuIyMgUG9seWdvbmF0dW0gbXVsdGlmbG9ydW1cbnN1YnBvbDwtc3Vic2V0KEdlbkRpdl9hbGwsR2VuRGl2X2FsbCRTcGVjaWVzLng9PVxcUG9sXFwpXG5zdWJwb2wkQWdlX3Q8LXNjYWxlKGJveGNveChzdWJwb2wkQWdlX2FicykpXG5cbmxtbS5Bci5wb2w8LWxtZShBcl90fihQb3BTaXplX3RyYW5zZm9ybWVkK0lGTV90cmFuc2Zvcm1lZCkqQWdlX3QscmFuZG9tPX4xfFJlZ2lvbixkYXRhPXN1YnBvbClcbnN1bW1hcnkobG1tLkFyLnBvbClcbnIuc3F1YXJlZEdMTU0obG1tLkFyLnBvbClcblxubG1tLkhlLnBvbDwtbG1lKEhlX3R+KFBvcFNpemVfdHJhbnNmb3JtZWQrSUZNX3RyYW5zZm9ybWVkKSpBZ2VfdCxyYW5kb209fjF8UmVnaW9uLGRhdGE9c3VicG9sKVxuc3VtbWFyeShsbW0uSGUucG9sKSMjKlxuci5zcXVhcmVkR0xNTShsbW0uSGUucG9sKVxuXG5sbW0uSG8ucG9sPC1sbWUoSG9fdH4oUG9wU2l6ZV90cmFuc2Zvcm1lZCtJRk1fdHJhbnNmb3JtZWQpKkFnZV90LHJhbmRvbT1+MXxSZWdpb24sZGF0YT1zdWJwb2wpXG5zdW1tYXJ5KGxtbS5Iby5wb2wpXG5yLnNxdWFyZWRHTE1NKGxtbS5Iby5wb2wpXG5cbmxtbS5GaXMucG9sPC1sbWUoRmlzX3R+KFBvcFNpemVfdHJhbnNmb3JtZWQrSUZNX3RyYW5zZm9ybWVkKSpBZ2VfdCxyYW5kb209fjF8UmVnaW9uLGRhdGE9c3VicG9sKVxuc3VtbWFyeShsbW0uRmlzLnBvbClcbnIuc3F1YXJlZEdMTU0obG1tLkZpcy5wb2wpXG5cbmBgYFxuYGBgIn0= -->

```r
```r
## Polygonatum multiflorum
subpol<-subset(GenDiv_all,GenDiv_all$Species.x==\Pol\)
subpol$Age_t<-scale(boxcox(subpol$Age_abs))

lmm.Ar.pol<-lme(Ar_t~(PopSize_transformed+IFM_transformed)*Age_t,random=~1|Region,data=subpol)
summary(lmm.Ar.pol)
r.squaredGLMM(lmm.Ar.pol)

lmm.He.pol<-lme(He_t~(PopSize_transformed+IFM_transformed)*Age_t,random=~1|Region,data=subpol)
summary(lmm.He.pol)##*
r.squaredGLMM(lmm.He.pol)

lmm.Ho.pol<-lme(Ho_t~(PopSize_transformed+IFM_transformed)*Age_t,random=~1|Region,data=subpol)
summary(lmm.Ho.pol)
r.squaredGLMM(lmm.Ho.pol)

lmm.Fis.pol<-lme(Fis_t~(PopSize_transformed+IFM_transformed)*Age_t,random=~1|Region,data=subpol)
summary(lmm.Fis.pol)
r.squaredGLMM(lmm.Fis.pol)
```

```
<!-- rnb-source-end -->

<!-- rnb-chunk-end -->


<!-- rnb-text-begin -->


Genetic Differentiation

<!-- rnb-text-end -->


<!-- rnb-chunk-begin -->


<!-- rnb-source-begin eyJkYXRhIjoiYGBgclxuYGBgclxuIyMgR3N0IGFuZCBEcHNcbmxvYWQoXFxkaXN0LlJEYXRhXFwpXG5kaXN0JFNwZWNpZXMueDwtYXMuZmFjdG9yKGRpc3QkU3BlY2llcy54KVxuZGlzdCRTcGVjMjwtcmVsZXZlbChkaXN0JFNwZWNpZXMueCxyZWY9XFxPeGFcXClcbmRpc3QkU3BlYzM8LXJlbGV2ZWwoZGlzdCRTcGVjaWVzLngscmVmPVxcUG9sXFwpXG5cbkdzdDwtbG1lKEdzdF90fihEaXN0YW5jZV90K0FnZV9EaWZmX3QqQWdlX3lvdW5nZXJfdCkqU3BlY2llcy54LHJhbmRvbT1+MXxSZWdpb24sY29ycmVsYXRpb24gPSBjb3JNTFBFKGZvcm09fklOX1BPUC55K05FQVJfUE9QLnl8UmVnaW9uKSxkYXRhPWRpc3QpXG5kcmVkZ2UoR3N0LGZpeGVkPX5TcGVjaWVzLngsUkVNTD1GKVxuR3N0LmZpbjwtbG1lKEdzdF90fkFnZV95b3VuZ2VyX3QrRGlzdGFuY2VfdCpTcGVjaWVzLngscmFuZG9tPX4xfFJlZ2lvbixjb3JyZWxhdGlvbj1jb3JNTFBFKGZvcm09fklOX1BPUC55K05FQVJfUE9QLnl8UmVnaW9uKSxkYXRhPWRpc3QpXG5zdW1tYXJ5KEdzdC5maW4pXG5Hc3Qub2FjLmZpbjwtbG1lKEdzdF90fkFnZV95b3VuZ2VyX3QrRGlzdGFuY2VfdCpTcGVjMixyYW5kb209fjF8UmVnaW9uLGNvcnJlbGF0aW9uPWNvck1MUEUoZm9ybT1+SU5fUE9QLnkrTkVBUl9QT1AueXxSZWdpb24pLGRhdGE9ZGlzdClcbnN1bW1hcnkoR3N0Lm9hYy5maW4pXG5Hc3QucG11bC5maW48LWxtZShHc3RfdH5BZ2VfeW91bmdlcl90K0Rpc3RhbmNlX3QqU3BlYzMscmFuZG9tPX4xfFJlZ2lvbixjb3JyZWxhdGlvbj1jb3JNTFBFKGZvcm09fklOX1BPUC55K05FQVJfUE9QLnl8UmVnaW9uKSxkYXRhPWRpc3QpXG5zdW1tYXJ5KEdzdC5wbXVsLmZpbilcbnIuc3F1YXJlZEdMTU0oR3N0LmZpbilcblxuXG5EcHM8LWxtZShEcHNfdH4oRGlzdGFuY2VfdCtBZ2VfRGlmZl90KkFnZV95b3VuZ2VyX3QpKlNwZWNpZXMueCxyYW5kb209fjF8UmVnaW9uLGNvcnJlbGF0aW9uID0gY29yTUxQRShmb3JtPX5JTl9QT1AueStORUFSX1BPUC55fFJlZ2lvbiksZGF0YT1kaXN0KVxuZHJlZGdlKERwcyxmaXhlZD1+U3BlY2llcy54LFJFTUw9RilcbkRwcy5maW48LWxtZShEcHNfdH5BZ2VfeW91bmdlcl90K0Rpc3RhbmNlX3QrU3BlY2llcy54LHJhbmRvbT1+MXxSZWdpb24sY29ycmVsYXRpb249Y29yTUxQRShmb3JtPX5JTl9QT1AueStORUFSX1BPUC55fFJlZ2lvbiksZGF0YT1kaXN0KVxucGxvdChEcHMuZmluLGZvcm09cmVzaWQoLiwgdHlwZSA9IFxccFxcKSB+IGZpdHRlZCguKSwgYWJsaW5lPTApXG5xcW5vcm0oRHBzLmZpbix+cmVzaWQoLiwgdHlwZSA9IFxccFxcKSwgYWJsaW5lPWMoMCwxKSlcbnN1bW1hcnkoRHBzLmZpbilcbnIuc3F1YXJlZEdMTU0oRHBzLmZpbilcbmBgYFxuYGBgIn0= -->

```r
```r
## Gst and Dps
load(\dist.RData\)
dist$Species.x<-as.factor(dist$Species.x)
dist$Spec2<-relevel(dist$Species.x,ref=\Oxa\)
dist$Spec3<-relevel(dist$Species.x,ref=\Pol\)

Gst<-lme(Gst_t~(Distance_t+Age_Diff_t*Age_younger_t)*Species.x,random=~1|Region,correlation = corMLPE(form=~IN_POP.y+NEAR_POP.y|Region),data=dist)
dredge(Gst,fixed=~Species.x,REML=F)
Gst.fin<-lme(Gst_t~Age_younger_t+Distance_t*Species.x,random=~1|Region,correlation=corMLPE(form=~IN_POP.y+NEAR_POP.y|Region),data=dist)
summary(Gst.fin)
Gst.oac.fin<-lme(Gst_t~Age_younger_t+Distance_t*Spec2,random=~1|Region,correlation=corMLPE(form=~IN_POP.y+NEAR_POP.y|Region),data=dist)
summary(Gst.oac.fin)
Gst.pmul.fin<-lme(Gst_t~Age_younger_t+Distance_t*Spec3,random=~1|Region,correlation=corMLPE(form=~IN_POP.y+NEAR_POP.y|Region),data=dist)
summary(Gst.pmul.fin)
r.squaredGLMM(Gst.fin)


Dps<-lme(Dps_t~(Distance_t+Age_Diff_t*Age_younger_t)*Species.x,random=~1|Region,correlation = corMLPE(form=~IN_POP.y+NEAR_POP.y|Region),data=dist)
dredge(Dps,fixed=~Species.x,REML=F)
Dps.fin<-lme(Dps_t~Age_younger_t+Distance_t+Species.x,random=~1|Region,correlation=corMLPE(form=~IN_POP.y+NEAR_POP.y|Region),data=dist)
plot(Dps.fin,form=resid(., type = \p\) ~ fitted(.), abline=0)
qqnorm(Dps.fin,~resid(., type = \p\), abline=c(0,1))
summary(Dps.fin)
r.squaredGLMM(Dps.fin)
```

```
<!-- rnb-source-end -->

<!-- rnb-chunk-end -->


<!-- rnb-text-begin -->


<!-- rnb-text-end -->


<!-- rnb-chunk-begin -->


<!-- rnb-source-begin eyJkYXRhIjoiYGBgclxuIyMgY0dEXG5sb2FkKFwiQ29uX0Rpcy5SRGF0YVwiKVxuY0dEPC1sbWUoQ29uLkRfdH4oZ2VvZGlzdF90K0FnZV9EaWZmX3QqQWdlX3lvdW5nZXJfdCkqU3BlY2llcy54LHJhbmRvbT1+MXxSZWdpb24sY29ycmVsYXRpb24gPSBjb3JNTFBFKGZvcm09fklOX1BPUC55K05FQVJfUE9QLnl8UmVnaW9uKSxkYXRhPUNvbi5EaXMpXG5kcmVkZ2UoY0dELGZpeGVkPX5TcGVjaWVzLngsUkVNTD1GKVxuYy5HRC55LmZpbjwtbG1lKENvbi5EX3R+Z2VvZGlzdF90K0FnZV95b3VuZ2VyX3QqU3BlY2llcy54LHJhbmRvbT1+MXxSZWdpb24sY29ycmVsYXRpb24gPSBjb3JNTFBFKGZvcm09fklOX1BPUC55K05FQVJfUE9QLnl8UmVnaW9uKSxkYXRhPUNvbi5EaXMpXG5zdW1tYXJ5KGMuR0QueS5maW4pXG5yLnNxdWFyZWRHTE1NKGMuR0QueS5maW4pXG5cbkNvbi5EaXMkU3BlY2llcy54PC1hcy5mYWN0b3IoQ29uLkRpcyRTcGVjaWVzLngpXG5Db24uRGlzJFNwZWMyPC1yZWxldmVsKENvbi5EaXMkU3BlY2llcy54LHJlZj1cIk94YVwiKVxuQ29uLkRpcyRTcGVjMzwtcmVsZXZlbChDb24uRGlzJFNwZWNpZXMueCxyZWY9XCJQb2xcIilcblxuYy5HRC55Lm94YTwtbG1lKENvbi5EX3R+Z2VvZGlzdF90K0FnZV95b3VuZ2VyX3QqU3BlYzIscmFuZG9tPX4xfFJlZ2lvbixjb3JyZWxhdGlvbiA9IGNvck1MUEUoZm9ybT1+SU5fUE9QLnkrTkVBUl9QT1AueXxSZWdpb24pLGRhdGE9Q29uLkRpcylcbnN1bW1hcnkoYy5HRC55Lm94YSlcbmMuR0QueS5wb2w8LWxtZShDb24uRF90fmdlb2Rpc3RfdCtBZ2VfeW91bmdlcl90KlNwZWMzLHJhbmRvbT1+MXxSZWdpb24sY29ycmVsYXRpb24gPSBjb3JNTFBFKGZvcm09fklOX1BPUC55K05FQVJfUE9QLnl8UmVnaW9uKSxkYXRhPUNvbi5EaXMpXG5zdW1tYXJ5KGMuR0QueS5wb2wpXG5cbmBgYCJ9 -->

```r
## cGD
load("Con_Dis.RData")
cGD<-lme(Con.D_t~(geodist_t+Age_Diff_t*Age_younger_t)*Species.x,random=~1|Region,correlation = corMLPE(form=~IN_POP.y+NEAR_POP.y|Region),data=Con.Dis)
dredge(cGD,fixed=~Species.x,REML=F)
c.GD.y.fin<-lme(Con.D_t~geodist_t+Age_younger_t*Species.x,random=~1|Region,correlation = corMLPE(form=~IN_POP.y+NEAR_POP.y|Region),data=Con.Dis)
summary(c.GD.y.fin)
r.squaredGLMM(c.GD.y.fin)

Con.Dis$Species.x<-as.factor(Con.Dis$Species.x)
Con.Dis$Spec2<-relevel(Con.Dis$Species.x,ref="Oxa")
Con.Dis$Spec3<-relevel(Con.Dis$Species.x,ref="Pol")

c.GD.y.oxa<-lme(Con.D_t~geodist_t+Age_younger_t*Spec2,random=~1|Region,correlation = corMLPE(form=~IN_POP.y+NEAR_POP.y|Region),data=Con.Dis)
summary(c.GD.y.oxa)
c.GD.y.pol<-lme(Con.D_t~geodist_t+Age_younger_t*Spec3,random=~1|Region,correlation = corMLPE(form=~IN_POP.y+NEAR_POP.y|Region),data=Con.Dis)
summary(c.GD.y.pol)
```


```
```r
## Interactions between GEO_DIST and AGE_BASE/AGE_DIFF
## Anemone nemorosa
distane<-subset(dist,dist$Species.x==\Ane\)
distane$Distance_t<-scale(boxcox(distane$Distance,method=\pc\))
distane$Age_Diff_t<-scale(boxcox(distane$Age_Diff))
distane$Age_younger_t<-scale(boxcox(distane$Age_abs_y))

Gst.ane<-lme(Gst_t~Distance_t+Age_Diff_t+Age_younger_t+Distance_t:Age_Diff_t+Distance_t:Age_younger_t,random=~1|Region,correlation = corMLPE(form=~IN_POP.y+NEAR_POP.y|Region),data=distane)
plot(Gst.ane,form=resid(., type = \p\) ~ fitted(.), abline=0)
qqnorm(Gst.ane,~resid(., type = \p\), abline=c(0,1))
summary(Gst.ane)
r.squaredGLMM(Gst.ane)

Dps.ane<-lme(Dps_t~Distance_t+Age_Diff_t+Age_younger_t+Distance_t:Age_Diff_t+Distance_t:Age_younger_t,random=~1|Region,correlation = corMLPE(form=~IN_POP.y+NEAR_POP.y|Region),data=distane)
summary(Dps.ane)
r.squaredGLMM(Dps.ane)

Con.Dis.Ane<-Con.Dis[c(1:82),]## here i selected data set for anemone
Con.Dis.Ane$geodist_t<-scale(boxcox(Con.Dis.Ane$Distance))
Con.Dis.Ane$Age_Diff_t<-scale(boxcox(Con.Dis.Ane$Age_Diff))
Con.Dis.Ane$Age_younger_t<-scale(boxcox(Con.Dis.Ane$Age_abs_y))

cgd.ane<-lme(Con.D_t~geodist_t+Age_Diff_t+Age_younger_t+geodist_t:Age_Diff_t+geodist_t:Age_younger_t,random=~1|Region,correlation=corMLPE(form=~IN_POP.y+NEAR_POP.y|Region),data=Con.Dis.Ane)
qqnorm(cgd.ane,~resid(., type=\p\),abline=c(0,1))
summary(cgd.ane)
r.squaredGLMM(cgd.ane)
```

```
<!-- rnb-source-end -->

<!-- rnb-chunk-end -->


<!-- rnb-chunk-begin -->


<!-- rnb-source-begin eyJkYXRhIjoiYGBgclxuYGBgclxuIyMgT3hhbGlzIGFjZXRvc2VsbGFcbmRpc3RveGE8LXN1YnNldChkaXN0LGRpc3QkU3BlY2llcy54PT1cXE94YVxcKVxuZGlzdG94YSREaXN0YW5jZV90PC1zY2FsZShib3hjb3goZGlzdG94YSREaXN0YW5jZSxtZXRob2Q9XFxwY1xcKSlcbmRpc3RveGEkQWdlX0RpZmZfdDwtc2NhbGUoYm94Y294KGRpc3RveGEkQWdlX0RpZmYpKSAjIyBhZ2UgZWxlbWVudCBvbmx5IHNjYWxlZCBidXQgbm90IGJveGNveCB0cmFuc2Zvcm1lZFxuZGlzdG94YSRBZ2VfeW91bmdlcl90PC1zY2FsZShib3hjb3goZGlzdG94YSRBZ2VfYWJzX3kpKVxuXG5Hc3Qub3hhPC1sbWUoR3N0X3R+RGlzdGFuY2VfdCtBZ2VfRGlmZl90K0FnZV95b3VuZ2VyX3QrRGlzdGFuY2VfdDpBZ2VfRGlmZl90K0Rpc3RhbmNlX3Q6QWdlX3lvdW5nZXJfdCxyYW5kb209fjF8UmVnaW9uLGNvcnJlbGF0aW9uID0gY29yTUxQRShmb3JtPX5JTl9QT1AueStORUFSX1BPUC55fFJlZ2lvbiksZGF0YT1kaXN0b3hhKVxuc3VtbWFyeShHc3Qub3hhKVxuci5zcXVhcmVkR0xNTShHc3Qub3hhKVxuRHBzLm94YTwtbG1lKERwc190fkRpc3RhbmNlX3QrQWdlX0RpZmZfdCtBZ2VfeW91bmdlcl90K0Rpc3RhbmNlX3Q6QWdlX0RpZmZfdCtEaXN0YW5jZV90OkFnZV95b3VuZ2VyX3QscmFuZG9tID0gfjF8UmVnaW9uLGNvcnJlbGF0aW9uPWNvck1MUEUoZm9ybT1+SU5fUE9QLnkrTkVBUl9QT1AueXxSZWdpb24pLGRhdGE9ZGlzdG94YSlcbnN1bW1hcnkoRHBzLm94YSlcbnIuc3F1YXJlZEdMTU0oRHBzLm94YSlcblxuQ29uLkRpcy5PeGE8LUNvbi5EaXNbYyg4MzoxMzQpLF1cbkNvbi5EaXMuT3hhJGdlb2Rpc3RfdDwtc2NhbGUoYm94Y294KENvbi5EaXMuT3hhJERpc3RhbmNlKSlcbkNvbi5EaXMuT3hhJEFnZV9EaWZmX3Q8LXNjYWxlKGJveGNveChDb24uRGlzLk94YSRBZ2VfRGlmZikpXG5Db24uRGlzLk94YSRBZ2VfeW91bmdlcl90PC1zY2FsZShib3hjb3goQ29uLkRpcy5PeGEkQWdlX2Fic195KSlcblxuXG5jZ2Qub3hhPC1sbWUoQ29uLkRfdH5nZW9kaXN0X3QrQWdlX0RpZmZfdCtBZ2VfeW91bmdlcl90K2dlb2Rpc3RfdDpBZ2VfRGlmZl90K2dlb2Rpc3RfdDpBZ2VfeW91bmdlcl90LHJhbmRvbT1+MXxSZWdpb24sY29ycmVsYXRpb249Y29yTUxQRShmb3JtPX5JTl9QT1AueStORUFSX1BPUC55fFJlZ2lvbiksZGF0YT1Db24uRGlzLk94YSlcbnFxbm9ybShjZ2Qub3hhLH5yZXNpZCguLCB0eXBlPVxccFxcKSxhYmxpbmU9YygwLDEpKVxuc3VtbWFyeShjZ2Qub3hhKVxuci5zcXVhcmVkR0xNTShjZ2Qub3hhKVxuXG5cbmBgYFxuYGBgIn0= -->

```r
```r
## Oxalis acetosella
distoxa<-subset(dist,dist$Species.x==\Oxa\)
distoxa$Distance_t<-scale(boxcox(distoxa$Distance,method=\pc\))
distoxa$Age_Diff_t<-scale(boxcox(distoxa$Age_Diff)) ## age element only scaled but not boxcox transformed
distoxa$Age_younger_t<-scale(boxcox(distoxa$Age_abs_y))

Gst.oxa<-lme(Gst_t~Distance_t+Age_Diff_t+Age_younger_t+Distance_t:Age_Diff_t+Distance_t:Age_younger_t,random=~1|Region,correlation = corMLPE(form=~IN_POP.y+NEAR_POP.y|Region),data=distoxa)
summary(Gst.oxa)
r.squaredGLMM(Gst.oxa)
Dps.oxa<-lme(Dps_t~Distance_t+Age_Diff_t+Age_younger_t+Distance_t:Age_Diff_t+Distance_t:Age_younger_t,random = ~1|Region,correlation=corMLPE(form=~IN_POP.y+NEAR_POP.y|Region),data=distoxa)
summary(Dps.oxa)
r.squaredGLMM(Dps.oxa)

Con.Dis.Oxa<-Con.Dis[c(83:134),]
Con.Dis.Oxa$geodist_t<-scale(boxcox(Con.Dis.Oxa$Distance))
Con.Dis.Oxa$Age_Diff_t<-scale(boxcox(Con.Dis.Oxa$Age_Diff))
Con.Dis.Oxa$Age_younger_t<-scale(boxcox(Con.Dis.Oxa$Age_abs_y))


cgd.oxa<-lme(Con.D_t~geodist_t+Age_Diff_t+Age_younger_t+geodist_t:Age_Diff_t+geodist_t:Age_younger_t,random=~1|Region,correlation=corMLPE(form=~IN_POP.y+NEAR_POP.y|Region),data=Con.Dis.Oxa)
qqnorm(cgd.oxa,~resid(., type=\p\),abline=c(0,1))
summary(cgd.oxa)
r.squaredGLMM(cgd.oxa)
```

```
<!-- rnb-source-end -->

<!-- rnb-chunk-end -->


<!-- rnb-chunk-begin -->


<!-- rnb-source-begin eyJkYXRhIjoiYGBgclxuYGBgclxuIyMgUG9seWdvbmF0dW0gbXVsdGlmbG9ydW1cbmRpc3Rwb2w8LXN1YnNldChkaXN0LGRpc3QkU3BlY2llcy54PT1cXFBvbFxcKVxuZGlzdHBvbCREaXN0YW5jZV90PC1zY2FsZShib3hjb3goZGlzdHBvbCREaXN0YW5jZSxtZXRob2Q9XFxwY1xcKSlcbmRpc3Rwb2wkQWdlX0RpZmZfdDwtc2NhbGUoYm94Y294KGRpc3Rwb2wkQWdlX0RpZmYpKSAjIyBhZ2UgZWxlbWVudCBvbmx5IHNjYWxlZCBidXQgbm90IGJveGNveCB0cmFuc2Zvcm1lZFxuZGlzdHBvbCRBZ2VfeW91bmdlcl90PC1zY2FsZShib3hjb3goZGlzdHBvbCRBZ2VfYWJzX3kpKVxuXG5Hc3QucG9sPC1sbWUoR3N0X3R+RGlzdGFuY2VfdCtBZ2VfRGlmZl90K0FnZV95b3VuZ2VyX3QrRGlzdGFuY2VfdDpBZ2VfRGlmZl90K0Rpc3RhbmNlX3Q6QWdlX3lvdW5nZXJfdCxyYW5kb209fjF8UmVnaW9uLGNvcnJlbGF0aW9uPWNvck1MUEUoZm9ybT1+SU5fUE9QLnkrTkVBUl9QT1AueXxSZWdpb24pLGRhdGE9ZGlzdHBvbClcbnN1bW1hcnkoR3N0LnBvbClcbnIuc3F1YXJlZEdMTU0oR3N0LnBvbClcbkRwcy5wb2w8LWxtZShEcHNfdH5EaXN0YW5jZV90K0FnZV9EaWZmX3QrQWdlX3lvdW5nZXJfdCtEaXN0YW5jZV90OkFnZV9EaWZmX3QrRGlzdGFuY2VfdDpBZ2VfeW91bmdlcl90LHJhbmRvbT1+MXxSZWdpb24sY29ycmVsYXRpb249Y29yTUxQRShmb3JtPX5JTl9QT1AueStORUFSX1BPUC55fFJlZ2lvbiksZGF0YT1kaXN0cG9sKVxuc3VtbWFyeShEcHMucG9sKVxuci5zcXVhcmVkR0xNTShEcHMucG9sKVxuXG5Db24uRGlzLlBvbDwtQ29uLkRpc1tjKDEzNToyMDgpLF1cbkNvbi5EaXMuUG9sJGdlb2Rpc3RfdDwtc2NhbGUoYm94Y294KENvbi5EaXMuUG9sJERpc3RhbmNlKSlcbkNvbi5EaXMuUG9sJEFnZV9EaWZmX3Q8LXNjYWxlKGJveGNveChDb24uRGlzLlBvbCRBZ2VfRGlmZikpXG5Db24uRGlzLlBvbCRBZ2VfeW91bmdlcl90PC1zY2FsZShib3hjb3goQ29uLkRpcy5Qb2wkQWdlX2Fic195KSlcblxuXG5jZ2QucG9sPC1sbWUoQ29uLkRfdH5nZW9kaXN0X3QrQWdlX0RpZmZfdCtBZ2VfeW91bmdlcl90K2dlb2Rpc3RfdDpBZ2VfRGlmZl90K2dlb2Rpc3RfdDpBZ2VfeW91bmdlcl90LHJhbmRvbT1+MXxSZWdpb24sY29ycmVsYXRpb249Y29yTUxQRShmb3JtPX5JTl9QT1AueStORUFSX1BPUC55fFJlZ2lvbiksZGF0YT1Db24uRGlzLlBvbClcbnFxbm9ybShjZ2QucG9sLH5yZXNpZCguLCB0eXBlPVxccFxcKSxhYmxpbmU9YygwLDEpKVxuc3VtbWFyeShjZ2QucG9sKVxuci5zcXVhcmVkR0xNTShjZ2QucG9sKVxuXG5cbmBgYFxuYGBgIn0= -->

```r
```r
## Polygonatum multiflorum
distpol<-subset(dist,dist$Species.x==\Pol\)
distpol$Distance_t<-scale(boxcox(distpol$Distance,method=\pc\))
distpol$Age_Diff_t<-scale(boxcox(distpol$Age_Diff)) ## age element only scaled but not boxcox transformed
distpol$Age_younger_t<-scale(boxcox(distpol$Age_abs_y))

Gst.pol<-lme(Gst_t~Distance_t+Age_Diff_t+Age_younger_t+Distance_t:Age_Diff_t+Distance_t:Age_younger_t,random=~1|Region,correlation=corMLPE(form=~IN_POP.y+NEAR_POP.y|Region),data=distpol)
summary(Gst.pol)
r.squaredGLMM(Gst.pol)
Dps.pol<-lme(Dps_t~Distance_t+Age_Diff_t+Age_younger_t+Distance_t:Age_Diff_t+Distance_t:Age_younger_t,random=~1|Region,correlation=corMLPE(form=~IN_POP.y+NEAR_POP.y|Region),data=distpol)
summary(Dps.pol)
r.squaredGLMM(Dps.pol)

Con.Dis.Pol<-Con.Dis[c(135:208),]
Con.Dis.Pol$geodist_t<-scale(boxcox(Con.Dis.Pol$Distance))
Con.Dis.Pol$Age_Diff_t<-scale(boxcox(Con.Dis.Pol$Age_Diff))
Con.Dis.Pol$Age_younger_t<-scale(boxcox(Con.Dis.Pol$Age_abs_y))


cgd.pol<-lme(Con.D_t~geodist_t+Age_Diff_t+Age_younger_t+geodist_t:Age_Diff_t+geodist_t:Age_younger_t,random=~1|Region,correlation=corMLPE(form=~IN_POP.y+NEAR_POP.y|Region),data=Con.Dis.Pol)
qqnorm(cgd.pol,~resid(., type=\p\),abline=c(0,1))
summary(cgd.pol)
r.squaredGLMM(cgd.pol)
```

```
<!-- rnb-source-end -->

<!-- rnb-chunk-end -->


<!-- rnb-chunk-begin -->


<!-- rnb-source-begin eyJkYXRhIjoiYGBgclxuYGBgclxuIyMgTGluayBsZXZlbCBESUZGX0dFTl9HRU9cbmxvYWQoXFxMaW5rLlJEYXRhXFwpXG5MaW5rJERpZmZfR19QPC1hcy5udW1lcmljKExpbmskRGlmZl9HX1ApXG5MaW5rX2FuZTwtc3Vic2V0KExpbmssTGluayRzcGVjaWVzPT1cXEFuZVxcKVxuTGlua19hbmUkRGlmZl9HX1BfdDwtc2NhbGUoTGlua19hbmUkRGlmZl9HX1ApXG5cbkxpbmtfb3hhPC1zdWJzZXQoTGluayxMaW5rJHNwZWNpZXM9PVxcT3hhXFwpXG5MaW5rX294YSREaWZmX0dfUF90PC1zY2FsZShMaW5rX294YSREaWZmX0dfUClcblxuTGlua19wb2w8LXN1YnNldChMaW5rLExpbmskc3BlY2llcz09XFxQb2xcXClcbkxpbmtfcG9sJERpZmZfR19QX3Q8LXNjYWxlKExpbmtfcG9sJERpZmZfR19QKVxuXG5MaW5rPC1yYmluZChMaW5rX2FuZSxMaW5rX294YSxMaW5rX3BvbClcblxuTGluayRBZ2VfRGlmZl90PC1zY2FsZShib3hjb3goTGluayRBZ2VfRGlmZikpXG5MaW5rPC1MaW5rJT4lXG4gIG11dGF0ZShBZ2VfYWJzX3k9MjAyMC1BZ2VfeW91bmdlcilcbkxpbmskQWdlX3lvdW5nZXJfdDwtc2NhbGUoYm94Y294KExpbmskQWdlX2Fic195KSlcblxubGluay5sbTwtbG1lKERpZmZfR19QX3R+QWdlX0RpZmZfdCpBZ2VfeW91bmdlcl90KlNwZWNpZXMscmFuZG9tPX4xfExXLngsY29ycmVsYXRpb24gPSBjb3JNTFBFKGZvcm09fm5vZGVfMStub2RlXzJ8TFcueCksZGF0YT1MaW5rKVxuZHJlZGdlKGxpbmsubG0sZml4ZWQ9flNwZWNpZXMsUkVNTD1GKVxuXG5saW5rLmZpbjwtbG1lKERpZmZfR19QX3R+QWdlX3lvdW5nZXJfdCtTcGVjaWVzLHJhbmRvbT1+MXxMVy54LGNvcnJlbGF0aW9uPWNvck1MUEUoZm9ybT1+bm9kZV8xK25vZGVfMnxMVy54KSxkYXRhPUxpbmspXG5wbG90KGxpbmsuZmluLGZvcm09cmVzaWQoLiwgdHlwZSA9IFxccFxcKSB+IGZpdHRlZCguKSwgYWJsaW5lPTApXG5xcW5vcm0obGluay5maW4sfnJlc2lkKC4sIHR5cGUgPSBcXHBcXCksIGFibGluZT1jKDAsMSkpXG5zdW1tYXJ5KGxpbmsuZmluKVxubGluay5maW4yPC1sbWUoRGlmZl9HX1BfdH5BZ2VfRGlmZl90K1NwZWNpZXMscmFuZG9tPX4xfExXLngsY29ycmVsYXRpb249Y29yTUxQRShmb3JtPX5ub2RlXzErbm9kZV8yfExXLngpLGRhdGE9TGluaylcbnN1bW1hcnkobGluay5maW4yKVxuci5zcXVhcmVkR0xNTShHc3QuZmluKVxuYGBgXG5gYGAifQ== -->

```r
```r
## Link level DIFF_GEN_GEO
load(\Link.RData\)
Link$Diff_G_P<-as.numeric(Link$Diff_G_P)
Link_ane<-subset(Link,Link$species==\Ane\)
Link_ane$Diff_G_P_t<-scale(Link_ane$Diff_G_P)

Link_oxa<-subset(Link,Link$species==\Oxa\)
Link_oxa$Diff_G_P_t<-scale(Link_oxa$Diff_G_P)

Link_pol<-subset(Link,Link$species==\Pol\)
Link_pol$Diff_G_P_t<-scale(Link_pol$Diff_G_P)

Link<-rbind(Link_ane,Link_oxa,Link_pol)

Link$Age_Diff_t<-scale(boxcox(Link$Age_Diff))
Link<-Link%>%
  mutate(Age_abs_y=2020-Age_younger)
Link$Age_younger_t<-scale(boxcox(Link$Age_abs_y))

link.lm<-lme(Diff_G_P_t~Age_Diff_t*Age_younger_t*Species,random=~1|LW.x,correlation = corMLPE(form=~node_1+node_2|LW.x),data=Link)
dredge(link.lm,fixed=~Species,REML=F)

link.fin<-lme(Diff_G_P_t~Age_younger_t+Species,random=~1|LW.x,correlation=corMLPE(form=~node_1+node_2|LW.x),data=Link)
plot(link.fin,form=resid(., type = \p\) ~ fitted(.), abline=0)
qqnorm(link.fin,~resid(., type = \p\), abline=c(0,1))
summary(link.fin)
link.fin2<-lme(Diff_G_P_t~Age_Diff_t+Species,random=~1|LW.x,correlation=corMLPE(form=~node_1+node_2|LW.x),data=Link)
summary(link.fin2)
r.squaredGLMM(Gst.fin)
```

```
<!-- rnb-source-end -->

<!-- rnb-chunk-end -->


<!-- rnb-text-begin -->


<!-- rnb-text-end -->


<!-- rnb-chunk-begin -->


<!-- rnb-source-begin eyJkYXRhIjoiYGBgclxuYGBgclxuIyMgTm9kZSBsZXZlbFxubG9hZChcXE5vZGUuUkRhdGFcXClcbk5vZGVfYW5lPC1zdWJzZXQoTm9kZSxOb2RlJFNwZWNpZXM9PVxcQW5lXFwpXG5Ob2RlX294YTwtc3Vic2V0KE5vZGUsTm9kZSRTcGVjaWVzPT1cXE94YVxcKVxuTm9kZV9wb2w8LXN1YnNldChOb2RlLE5vZGUkU3BlY2llcz09XFxQb2xcXClcblxuTm9kZV9hbmUkY2xvc2VuZXNzX3Q8LXNjYWxlKE5vZGVfYW5lJGNsb3NlbmVzcylcbk5vZGVfb3hhJGNsb3NlbmVzc190PC1zY2FsZShOb2RlX294YSRjbG9zZW5lc3MpXG5Ob2RlX3BvbCRjbG9zZW5lc3NfdDwtc2NhbGUoTm9kZV9wb2wkY2xvc2VuZXNzKVxuXG5Ob2RlPC1yYmluZChOb2RlX2FuZSxOb2RlX294YSxOb2RlX3BvbClcbk5vZGUkQWdlX3Q8LXNjYWxlKGJveGNveChOb2RlJEFnZV9hYnMpKSMjIGxhbWJkYT0wLjQ1LCBhZGRlZD0wXG5ub2RlLmxtPC1sbWUoY2xvc2VuZXNzX3R+QWdlX3QqU3BlY2llcyxyYW5kb209fjF8TFcsZGF0YT1Ob2RlKVxuZHJlZGdlKG5vZGUubG0sZml4ZWQ9flNwZWNpZXMsUkVNTD1GKVxubm9kZS5maW48LWxtZShjbG9zZW5lc3NfdH5BZ2VfdCtTcGVjaWVzLHJhbmRvbT1+MXxMVyxkYXRhPU5vZGUpXG5zdW1tYXJ5KG5vZGUuZmluKVxucGxvdChub2RlLmZpbixmb3JtPXJlc2lkKC4sIHR5cGUgPSBcXHBcXCkgfiBmaXR0ZWQoLiksIGFibGluZT0wKVxucXFub3JtKG5vZGUuZmluLH5yZXNpZCguLCB0eXBlID0gXFxwXFwpLCBhYmxpbmU9YygwLDEpKVxuXG5gYGBcbmBgYCJ9 -->

```r
```r
## Node level
load(\Node.RData\)
Node_ane<-subset(Node,Node$Species==\Ane\)
Node_oxa<-subset(Node,Node$Species==\Oxa\)
Node_pol<-subset(Node,Node$Species==\Pol\)

Node_ane$closeness_t<-scale(Node_ane$closeness)
Node_oxa$closeness_t<-scale(Node_oxa$closeness)
Node_pol$closeness_t<-scale(Node_pol$closeness)

Node<-rbind(Node_ane,Node_oxa,Node_pol)
Node$Age_t<-scale(boxcox(Node$Age_abs))## lambda=0.45, added=0
node.lm<-lme(closeness_t~Age_t*Species,random=~1|LW,data=Node)
dredge(node.lm,fixed=~Species,REML=F)
node.fin<-lme(closeness_t~Age_t+Species,random=~1|LW,data=Node)
summary(node.fin)
plot(node.fin,form=resid(., type = \p\) ~ fitted(.), abline=0)
qqnorm(node.fin,~resid(., type = \p\), abline=c(0,1))
```

```


LS0tDQp0aXRsZTogIkxpbmVhciBtb2RlbCBmb3IgZ2VuZXRpYyBkaXZlcnNpdHkgYW5kIGdlbmV0aWMgZGlmZmVyZW50aWF0aW9uIGFzIHdlbGwgYXMgcG9wdWxhdGlvbiBncmFwaCINCm91dHB1dDogaHRtbF9ub3RlYm9vaw0KLS0tDQoNCkdlbmV0aWMgRGl2ZXJzaXR5DQpgYGB7cn0NCmxpYnJhcnkob3Blbnhsc3gpDQpsaWJyYXJ5KG5sbWUpDQpsaWJyYXJ5KGRwbHlyKQ0KbGlicmFyeShwc3ljaCkNCmxpYnJhcnkoTXVNSW4pDQpsaWJyYXJ5KGNhcikNCmxpYnJhcnkoTUFTUykNCmxpYnJhcnkodGlkeXZlcnNlKQ0KbGlicmFyeShjb3JNTFBFKQ0Kc291cmNlKCJMOi8wNV9EYXRhIGFuYWx5c2lzL2JveGNveC5yIikNCmxvYWQoIkdlbkRpdl9hbGwuUkRhdGEiKQ0KR2VuRGl2X2FsbCRTcGVjaWVzLng8LWZhY3RvcihHZW5EaXZfYWxsJFNwZWNpZXMueCxvcmRlcmVkPUYpDQpHZW5EaXZfYWxsJFNwZWMyPC1yZWxldmVsKEdlbkRpdl9hbGwkU3BlY2llcy54LHJlZj0iT3hhIikNCkdlbkRpdl9hbGwkU3BlYzM8LXJlbGV2ZWwoR2VuRGl2X2FsbCRTcGVjaWVzLngscmVmPSJQb2wiKQ0KDQpsbW0uQXIuYjwtIGxtZShBcl90fihQb3BTaXplX3RyYW5zZm9ybWVkK0lGTV90cmFuc2Zvcm1lZCtBZ2VfdCkqU3BlY2llcy54LHJhbmRvbT1+MXxSZWdpb24vUG9wdWxhdGlvbixkYXRhPUdlbkRpdl9hbGwpDQpkcmVkZ2UobG1tLkFyLmIsZml4ZWQ9flNwZWNpZXMueCwgUkVNTD1GKQ0KbG1tLkFyLmIuZmluPC1sbWUoQXJfdH5JRk1fdHJhbnNmb3JtZWQrUG9wU2l6ZV90cmFuc2Zvcm1lZCtTcGVjaWVzLngqQWdlX3QscmFuZG9tPX4xfFJlZ2lvbi9Qb3B1bGF0aW9uLGRhdGE9R2VuRGl2X2FsbCkNCg0KcGxvdChsbW0uQXIuYi5maW4sIGZvcm09cmVzaWQoLiwgdHlwZSA9ICJwIikgfiBmaXR0ZWQoLiksIGFibGluZT0wKSAjY2hlY2sNCnFxbm9ybShsbW0uQXIuYi5maW4sfnJlc2lkKC4sIHR5cGUgPSAicCIpLCBhYmxpbmU9YygwLDEpKQ0KDQpzdW1tYXJ5KGxtbS5Bci5iLmZpbikNCg0KbG1tLkFyLmIub3hhLmZpbjwtbG1lKEFyX3R+SUZNX3RyYW5zZm9ybWVkK1BvcFNpemVfdHJhbnNmb3JtZWQrU3BlYzIqQWdlX3QscmFuZG9tPX4xfFJlZ2lvbi9Qb3B1bGF0aW9uLGRhdGE9R2VuRGl2X2FsbCkNCnN1bW1hcnkobG1tLkFyLmIub3hhLmZpbikNCg0KbG1tLkFyLmIucG9sLmZpbjwtbG1lKEFyX3R+SUZNX3RyYW5zZm9ybWVkK1BvcFNpemVfdHJhbnNmb3JtZWQrU3BlYzMqQWdlX3QscmFuZG9tPX4xfFJlZ2lvbi9Qb3B1bGF0aW9uLGRhdGE9R2VuRGl2X2FsbCkNCnN1bW1hcnkobG1tLkFyLmIucG9sLmZpbikNCg0Kci5zcXVhcmVkR0xNTShsbW0uQXIuYi5maW4pDQoNCmBgYA0KDQpgYGB7cn0NCiMjIEhlfihQT1BfU0laRStTUEFfQ09OK1BPUF9BR0UpKlNQRUNJRVMNCmxtbS5IZS5iPC1sbWUoSGVfdH4oUG9wU2l6ZV90cmFuc2Zvcm1lZCtJRk1fdHJhbnNmb3JtZWQrQWdlX3QpKlNwZWNpZXMueCxyYW5kb209fjF8UmVnaW9uL1BvcHVsYXRpb24sZGF0YT1HZW5EaXZfYWxsKQ0KZHJlZGdlKGxtbS5IZS5iLGZpeGVkPX5TcGVjaWVzLngsIFJFTUw9RikNCmxtbS5IZS5iLmZpbjwtbG1lKEhlX3R+SUZNX3RyYW5zZm9ybWVkK0FnZV90KlNwZWNpZXMueCxyYW5kb209fjF8UmVnaW9uL1BvcHVsYXRpb24sZGF0YT1HZW5EaXZfYWxsKQ0KcGxvdChsbW0uSGUuYi5maW4sZm9ybT1yZXNpZCguLCB0eXBlID0gInAiKSB+IGZpdHRlZCguKSwgYWJsaW5lPTApDQpxcW5vcm0obG1tLkhlLmIuZmluLH5yZXNpZCguLCB0eXBlID0gInAiKSwgYWJsaW5lPWMoMCwxKSkNCnN1bW1hcnkobG1tLkhlLmIuZmluKQ0KDQpsbW0uSGUuYi5veGEuZmluPC1sbWUoSGVfdH5JRk1fdHJhbnNmb3JtZWQrQWdlX3QqU3BlYzIscmFuZG9tPX4xfFJlZ2lvbi9Qb3B1bGF0aW9uLGRhdGE9R2VuRGl2X2FsbCkNCnN1bW1hcnkobG1tLkhlLmIub3hhLmZpbikNCg0KbG1tLkhlLmIucG9sLmZpbjwtbG1lKEhlX3R+SUZNX3RyYW5zZm9ybWVkK0FnZV90KlNwZWMzLHJhbmRvbT1+MXxSZWdpb24vUG9wdWxhdGlvbixkYXRhPUdlbkRpdl9hbGwpDQpzdW1tYXJ5KGxtbS5IZS5iLnBvbC5maW4pDQoNCnIuc3F1YXJlZEdMTU0obG1tLkhlLmIuZmluKQ0KDQpgYGANCg0KYGBge3J9DQojIyBIb34oUE9QX1NJWkUrU1BBX0NPTitQT1BfQUdFKSpTUEVDSUVTDQpsbW0uSG8uYjwtbG1lKEhvX3R+KFBvcFNpemVfdHJhbnNmb3JtZWQrSUZNX3RyYW5zZm9ybWVkK0FnZV90KSpTcGVjaWVzLngscmFuZG9tPX4xfFJlZ2lvbi9Qb3B1bGF0aW9uLGRhdGE9R2VuRGl2X2FsbCkNCmRyZWRnZShsbW0uSG8uYixmaXhlZD1+U3BlY2llcy54LFJFTUw9RikNCmxtbS5Iby5iLmZpbjwtbG1lKEhvX3R+QWdlX3QrSUZNX3RyYW5zZm9ybWVkKlNwZWNpZXMueCxyYW5kb209fjF8UmVnaW9uL1BvcHVsYXRpb24sZGF0YT1HZW5EaXZfYWxsKQ0KcGxvdChsbW0uSG8uYi5maW4sZm9ybT1yZXNpZCguLCB0eXBlID0gInAiKSB+IGZpdHRlZCguKSwgYWJsaW5lPTApDQpxcW5vcm0obG1tLkhvLmIuZmluLH5yZXNpZCguLCB0eXBlID0gInAiKSwgYWJsaW5lPWMoMCwxKSkNCnN1bW1hcnkobG1tLkhvLmIuZmluKQ0KDQpsbW0uSG8uYi5veGEuZmluPC1sbWUoSG9fdH5BZ2VfdCtJRk1fdHJhbnNmb3JtZWQqU3BlYzIscmFuZG9tPX4xfFJlZ2lvbi9Qb3B1bGF0aW9uLGRhdGE9R2VuRGl2X2FsbCkNCnN1bW1hcnkobG1tLkhvLmIub3hhLmZpbikNCg0KbG1tLkhvLmIucG9sLmZpbjwtbG1lKEhvX3R+QWdlX3QrSUZNX3RyYW5zZm9ybWVkKlNwZWMzLHJhbmRvbT1+MXxSZWdpb24vUG9wdWxhdGlvbixkYXRhPUdlbkRpdl9hbGwpDQpzdW1tYXJ5KGxtbS5Iby5iLnBvbC5maW4pDQoNCg0Kci5zcXVhcmVkR0xNTShsbW0uSG8uYi5maW4pDQoNCmBgYA0KDQpgYGB7cn0NCiMjIEZpc34oUE9QX1NJWkUrU1BBX0NPTitQT1BfQUdFKSpTUEVDSUVTDQpsbW0uRmlzLmI8LWxtZShGaXNfdH4oUG9wU2l6ZV90cmFuc2Zvcm1lZCtJRk1fdHJhbnNmb3JtZWQrQWdlX3QpKlNwZWNpZXMueCxyYW5kb209fjF8UmVnaW9uL1BvcHVsYXRpb24sZGF0YT1HZW5EaXZfYWxsKQ0KZHJlZGdlKGxtbS5GaXMuYixmaXhlZD1+U3BlY2llcy54LFJFTUw9RikNCmxtbS5GaXMuYi5maW48LWxtZShGaXNfdH5JRk1fdHJhbnNmb3JtZWQrQWdlX3QrU3BlY2llcy54LHJhbmRvbT1+MXxSZWdpb24vUG9wdWxhdGlvbixkYXRhPUdlbkRpdl9hbGwpDQpwbG90KGxtbS5GaXMuYi5maW4sZm9ybT1yZXNpZCguLCB0eXBlID0gInAiKSB+IGZpdHRlZCguKSwgYWJsaW5lPTApDQpxcW5vcm0obG1tLkZpcy5iLmZpbix+cmVzaWQoLiwgdHlwZSA9ICJwIiksIGFibGluZT1jKDAsMSkpDQpzdW1tYXJ5KGxtbS5GaXMuYi5maW4pDQpyLnNxdWFyZWRHTE1NKGxtbS5GaXMuYi5maW4pDQoNCmBgYA0KDQpgYGB7cn0NCiMjIEludGVyYWN0aW9uIGJldHdlZW4gUE9QX0FHRSBhbmQgUE9QX1NJWkUvU1BBX0NPTg0KIyMgQW5lbW9uZSBuZW1vcm9zYQ0Kc3ViYW5lPC1zdWJzZXQoR2VuRGl2X2FsbCxHZW5EaXZfYWxsJFNwZWNpZXMueD09IkFuZSIpDQpzdWJhbmUkQWdlX3Q8LXNjYWxlKGJveGNveChzdWJhbmUkQWdlX2FicykpDQoNCmxtbS5Bci5hbmU8LWxtZShBcl90fihQb3BTaXplX3RyYW5zZm9ybWVkK0lGTV90cmFuc2Zvcm1lZCkqQWdlX3QscmFuZG9tPX4xfFJlZ2lvbixkYXRhPXN1YmFuZSkNCnN1bW1hcnkobG1tLkFyLmFuZSkNCnIuc3F1YXJlZEdMTU0obG1tLkFyLmFuZSkNCg0KbG1tLkhlLmFuZTwtbG1lKEhlX3R+KFBvcFNpemVfdHJhbnNmb3JtZWQrSUZNX3RyYW5zZm9ybWVkKSpBZ2VfdCxyYW5kb209fjF8UmVnaW9uLGRhdGE9c3ViYW5lKQ0Kc3VtbWFyeShsbW0uSGUuYW5lKQ0Kci5zcXVhcmVkR0xNTShsbW0uSGUuYW5lKQ0KDQpsbW0uSG8uYW5lPC1sbWUoSG9fdH4oUG9wU2l6ZV90cmFuc2Zvcm1lZCtJRk1fdHJhbnNmb3JtZWQpKkFnZV90LHJhbmRvbT1+MXxSZWdpb24sZGF0YT1zdWJhbmUpDQpzdW1tYXJ5KGxtbS5Iby5hbmUpDQpyLnNxdWFyZWRHTE1NKGxtbS5Iby5hbmUpDQoNCmxtbS5GaXMuYW5lPC1sbWUoRmlzX3R+KFBvcFNpemVfdHJhbnNmb3JtZWQrSUZNX3RyYW5zZm9ybWVkKSpBZ2VfdCxyYW5kb209fjF8UmVnaW9uLGRhdGE9c3ViYW5lKQ0Kc3VtbWFyeShsbW0uRmlzLmFuZSkNCnIuc3F1YXJlZEdMTU0obG1tLkZpcy5hbmUpDQoNCmBgYA0KDQpgYGB7cn0NCiMjIE94YWxpcyBhY2V0b3NlbGxhDQpzdWJveGE8LXN1YnNldChHZW5EaXZfYWxsLEdlbkRpdl9hbGwkU3BlY2llcy54PT0iT3hhIikNCnN1Ym94YSRBZ2VfdDwtc2NhbGUoYm94Y294KHN1Ym94YSRBZ2VfYWJzKSkNCg0KbG1tLkFyLm94YTwtbG1lKEFyX3R+KFBvcFNpemVfdHJhbnNmb3JtZWQrSUZNX3RyYW5zZm9ybWVkKSpBZ2VfdCxyYW5kb209fjF8UmVnaW9uLGRhdGE9c3Vib3hhKQ0Kc3VtbWFyeShsbW0uQXIub3hhKQ0Kci5zcXVhcmVkR0xNTShsbW0uQXIub3hhKQ0KDQpsbW0uSGUub3hhPC1sbWUoSGVfdH4oUG9wU2l6ZV90cmFuc2Zvcm1lZCtJRk1fdHJhbnNmb3JtZWQpKkFnZV90LHJhbmRvbT1+MXxSZWdpb24sZGF0YT1zdWJveGEpDQpzdW1tYXJ5KGxtbS5IZS5veGEpDQpyLnNxdWFyZWRHTE1NKGxtbS5IZS5veGEpDQoNCmxtbS5Iby5veGE8LWxtZShIb190fihQb3BTaXplX3RyYW5zZm9ybWVkK0lGTV90cmFuc2Zvcm1lZCkqQWdlX3QscmFuZG9tPX4xfFJlZ2lvbixkYXRhPXN1Ym94YSkNCnN1bW1hcnkobG1tLkhvLm94YSkNCnIuc3F1YXJlZEdMTU0obG1tLkhvLm94YSkNCg0KbG1tLkZpcy5veGE8LWxtZShGaXNfdH4oUG9wU2l6ZV90cmFuc2Zvcm1lZCtJRk1fdHJhbnNmb3JtZWQpKkFnZV90LHJhbmRvbT1+MXxSZWdpb24sZGF0YT1zdWJveGEpDQpzdW1tYXJ5KGxtbS5GaXMub3hhKQ0Kci5zcXVhcmVkR0xNTShsbW0uRmlzLm94YSkNCg0KYGBgDQoNCmBgYHtyfQ0KIyMgUG9seWdvbmF0dW0gbXVsdGlmbG9ydW0NCnN1YnBvbDwtc3Vic2V0KEdlbkRpdl9hbGwsR2VuRGl2X2FsbCRTcGVjaWVzLng9PSJQb2wiKQ0Kc3VicG9sJEFnZV90PC1zY2FsZShib3hjb3goc3VicG9sJEFnZV9hYnMpKQ0KDQpsbW0uQXIucG9sPC1sbWUoQXJfdH4oUG9wU2l6ZV90cmFuc2Zvcm1lZCtJRk1fdHJhbnNmb3JtZWQpKkFnZV90LHJhbmRvbT1+MXxSZWdpb24sZGF0YT1zdWJwb2wpDQpzdW1tYXJ5KGxtbS5Bci5wb2wpDQpyLnNxdWFyZWRHTE1NKGxtbS5Bci5wb2wpDQoNCmxtbS5IZS5wb2w8LWxtZShIZV90fihQb3BTaXplX3RyYW5zZm9ybWVkK0lGTV90cmFuc2Zvcm1lZCkqQWdlX3QscmFuZG9tPX4xfFJlZ2lvbixkYXRhPXN1YnBvbCkNCnN1bW1hcnkobG1tLkhlLnBvbCkjIyoNCnIuc3F1YXJlZEdMTU0obG1tLkhlLnBvbCkNCg0KbG1tLkhvLnBvbDwtbG1lKEhvX3R+KFBvcFNpemVfdHJhbnNmb3JtZWQrSUZNX3RyYW5zZm9ybWVkKSpBZ2VfdCxyYW5kb209fjF8UmVnaW9uLGRhdGE9c3VicG9sKQ0Kc3VtbWFyeShsbW0uSG8ucG9sKQ0Kci5zcXVhcmVkR0xNTShsbW0uSG8ucG9sKQ0KDQpsbW0uRmlzLnBvbDwtbG1lKEZpc190fihQb3BTaXplX3RyYW5zZm9ybWVkK0lGTV90cmFuc2Zvcm1lZCkqQWdlX3QscmFuZG9tPX4xfFJlZ2lvbixkYXRhPXN1YnBvbCkNCnN1bW1hcnkobG1tLkZpcy5wb2wpDQpyLnNxdWFyZWRHTE1NKGxtbS5GaXMucG9sKQ0KDQpgYGANCg0KR2VuZXRpYyBEaWZmZXJlbnRpYXRpb24NCmBgYHtyfQ0KIyMgR3N0IGFuZCBEcHMNCmxvYWQoImRpc3QuUkRhdGEiKQ0KZGlzdCRTcGVjaWVzLng8LWFzLmZhY3RvcihkaXN0JFNwZWNpZXMueCkNCmRpc3QkU3BlYzI8LXJlbGV2ZWwoZGlzdCRTcGVjaWVzLngscmVmPSJPeGEiKQ0KZGlzdCRTcGVjMzwtcmVsZXZlbChkaXN0JFNwZWNpZXMueCxyZWY9IlBvbCIpDQoNCkdzdDwtbG1lKEdzdF90fihEaXN0YW5jZV90K0FnZV9EaWZmX3QqQWdlX3lvdW5nZXJfdCkqU3BlY2llcy54LHJhbmRvbT1+MXxSZWdpb24sY29ycmVsYXRpb24gPSBjb3JNTFBFKGZvcm09fklOX1BPUC55K05FQVJfUE9QLnl8UmVnaW9uKSxkYXRhPWRpc3QpDQpkcmVkZ2UoR3N0LGZpeGVkPX5TcGVjaWVzLngsUkVNTD1GKQ0KR3N0LmZpbjwtbG1lKEdzdF90fkFnZV95b3VuZ2VyX3QrRGlzdGFuY2VfdCpTcGVjaWVzLngscmFuZG9tPX4xfFJlZ2lvbixjb3JyZWxhdGlvbj1jb3JNTFBFKGZvcm09fklOX1BPUC55K05FQVJfUE9QLnl8UmVnaW9uKSxkYXRhPWRpc3QpDQpzdW1tYXJ5KEdzdC5maW4pDQpHc3Qub2FjLmZpbjwtbG1lKEdzdF90fkFnZV95b3VuZ2VyX3QrRGlzdGFuY2VfdCpTcGVjMixyYW5kb209fjF8UmVnaW9uLGNvcnJlbGF0aW9uPWNvck1MUEUoZm9ybT1+SU5fUE9QLnkrTkVBUl9QT1AueXxSZWdpb24pLGRhdGE9ZGlzdCkNCnN1bW1hcnkoR3N0Lm9hYy5maW4pDQpHc3QucG11bC5maW48LWxtZShHc3RfdH5BZ2VfeW91bmdlcl90K0Rpc3RhbmNlX3QqU3BlYzMscmFuZG9tPX4xfFJlZ2lvbixjb3JyZWxhdGlvbj1jb3JNTFBFKGZvcm09fklOX1BPUC55K05FQVJfUE9QLnl8UmVnaW9uKSxkYXRhPWRpc3QpDQpzdW1tYXJ5KEdzdC5wbXVsLmZpbikNCnIuc3F1YXJlZEdMTU0oR3N0LmZpbikNCg0KDQpEcHM8LWxtZShEcHNfdH4oRGlzdGFuY2VfdCtBZ2VfRGlmZl90KkFnZV95b3VuZ2VyX3QpKlNwZWNpZXMueCxyYW5kb209fjF8UmVnaW9uLGNvcnJlbGF0aW9uID0gY29yTUxQRShmb3JtPX5JTl9QT1AueStORUFSX1BPUC55fFJlZ2lvbiksZGF0YT1kaXN0KQ0KZHJlZGdlKERwcyxmaXhlZD1+U3BlY2llcy54LFJFTUw9RikNCkRwcy5maW48LWxtZShEcHNfdH5BZ2VfeW91bmdlcl90K0Rpc3RhbmNlX3QrU3BlY2llcy54LHJhbmRvbT1+MXxSZWdpb24sY29ycmVsYXRpb249Y29yTUxQRShmb3JtPX5JTl9QT1AueStORUFSX1BPUC55fFJlZ2lvbiksZGF0YT1kaXN0KQ0KcGxvdChEcHMuZmluLGZvcm09cmVzaWQoLiwgdHlwZSA9ICJwIikgfiBmaXR0ZWQoLiksIGFibGluZT0wKQ0KcXFub3JtKERwcy5maW4sfnJlc2lkKC4sIHR5cGUgPSAicCIpLCBhYmxpbmU9YygwLDEpKQ0Kc3VtbWFyeShEcHMuZmluKQ0Kci5zcXVhcmVkR0xNTShEcHMuZmluKQ0KYGBgDQoNCmBgYHtyfQ0KIyMgY0dEDQpsb2FkKCJDb25fRGlzLlJEYXRhIikNCmNHRDwtbG1lKENvbi5EX3R+KGdlb2Rpc3RfdCtBZ2VfRGlmZl90KkFnZV95b3VuZ2VyX3QpKlNwZWNpZXMueCxyYW5kb209fjF8UmVnaW9uLGNvcnJlbGF0aW9uID0gY29yTUxQRShmb3JtPX5JTl9QT1AueStORUFSX1BPUC55fFJlZ2lvbiksZGF0YT1Db24uRGlzKQ0KZHJlZGdlKGNHRCxmaXhlZD1+U3BlY2llcy54LFJFTUw9RikNCmMuR0QueS5maW48LWxtZShDb24uRF90fmdlb2Rpc3RfdCtBZ2VfeW91bmdlcl90KlNwZWNpZXMueCxyYW5kb209fjF8UmVnaW9uLGNvcnJlbGF0aW9uID0gY29yTUxQRShmb3JtPX5JTl9QT1AueStORUFSX1BPUC55fFJlZ2lvbiksZGF0YT1Db24uRGlzKQ0Kc3VtbWFyeShjLkdELnkuZmluKQ0Kci5zcXVhcmVkR0xNTShjLkdELnkuZmluKQ0KDQpDb24uRGlzJFNwZWNpZXMueDwtYXMuZmFjdG9yKENvbi5EaXMkU3BlY2llcy54KQ0KQ29uLkRpcyRTcGVjMjwtcmVsZXZlbChDb24uRGlzJFNwZWNpZXMueCxyZWY9Ik94YSIpDQpDb24uRGlzJFNwZWMzPC1yZWxldmVsKENvbi5EaXMkU3BlY2llcy54LHJlZj0iUG9sIikNCg0KYy5HRC55Lm94YTwtbG1lKENvbi5EX3R+Z2VvZGlzdF90K0FnZV95b3VuZ2VyX3QqU3BlYzIscmFuZG9tPX4xfFJlZ2lvbixjb3JyZWxhdGlvbiA9IGNvck1MUEUoZm9ybT1+SU5fUE9QLnkrTkVBUl9QT1AueXxSZWdpb24pLGRhdGE9Q29uLkRpcykNCnN1bW1hcnkoYy5HRC55Lm94YSkNCmMuR0QueS5wb2w8LWxtZShDb24uRF90fmdlb2Rpc3RfdCtBZ2VfeW91bmdlcl90KlNwZWMzLHJhbmRvbT1+MXxSZWdpb24sY29ycmVsYXRpb24gPSBjb3JNTFBFKGZvcm09fklOX1BPUC55K05FQVJfUE9QLnl8UmVnaW9uKSxkYXRhPUNvbi5EaXMpDQpzdW1tYXJ5KGMuR0QueS5wb2wpDQoNCmBgYA0KDQpgYGB7cn0NCiMjIEludGVyYWN0aW9ucyBiZXR3ZWVuIEdFT19ESVNUIGFuZCBBR0VfQkFTRS9BR0VfRElGRg0KIyMgQW5lbW9uZSBuZW1vcm9zYQ0KZGlzdGFuZTwtc3Vic2V0KGRpc3QsZGlzdCRTcGVjaWVzLng9PSJBbmUiKQ0KZGlzdGFuZSREaXN0YW5jZV90PC1zY2FsZShib3hjb3goZGlzdGFuZSREaXN0YW5jZSxtZXRob2Q9InBjIikpDQpkaXN0YW5lJEFnZV9EaWZmX3Q8LXNjYWxlKGJveGNveChkaXN0YW5lJEFnZV9EaWZmKSkNCmRpc3RhbmUkQWdlX3lvdW5nZXJfdDwtc2NhbGUoYm94Y294KGRpc3RhbmUkQWdlX2Fic195KSkNCg0KR3N0LmFuZTwtbG1lKEdzdF90fkRpc3RhbmNlX3QrQWdlX0RpZmZfdCtBZ2VfeW91bmdlcl90K0Rpc3RhbmNlX3Q6QWdlX0RpZmZfdCtEaXN0YW5jZV90OkFnZV95b3VuZ2VyX3QscmFuZG9tPX4xfFJlZ2lvbixjb3JyZWxhdGlvbiA9IGNvck1MUEUoZm9ybT1+SU5fUE9QLnkrTkVBUl9QT1AueXxSZWdpb24pLGRhdGE9ZGlzdGFuZSkNCnBsb3QoR3N0LmFuZSxmb3JtPXJlc2lkKC4sIHR5cGUgPSAicCIpIH4gZml0dGVkKC4pLCBhYmxpbmU9MCkNCnFxbm9ybShHc3QuYW5lLH5yZXNpZCguLCB0eXBlID0gInAiKSwgYWJsaW5lPWMoMCwxKSkNCnN1bW1hcnkoR3N0LmFuZSkNCnIuc3F1YXJlZEdMTU0oR3N0LmFuZSkNCg0KRHBzLmFuZTwtbG1lKERwc190fkRpc3RhbmNlX3QrQWdlX0RpZmZfdCtBZ2VfeW91bmdlcl90K0Rpc3RhbmNlX3Q6QWdlX0RpZmZfdCtEaXN0YW5jZV90OkFnZV95b3VuZ2VyX3QscmFuZG9tPX4xfFJlZ2lvbixjb3JyZWxhdGlvbiA9IGNvck1MUEUoZm9ybT1+SU5fUE9QLnkrTkVBUl9QT1AueXxSZWdpb24pLGRhdGE9ZGlzdGFuZSkNCnN1bW1hcnkoRHBzLmFuZSkNCnIuc3F1YXJlZEdMTU0oRHBzLmFuZSkNCg0KQ29uLkRpcy5BbmU8LUNvbi5EaXNbYygxOjgyKSxdIyMgaGVyZSBpIHNlbGVjdGVkIGRhdGEgc2V0IGZvciBhbmVtb25lDQpDb24uRGlzLkFuZSRnZW9kaXN0X3Q8LXNjYWxlKGJveGNveChDb24uRGlzLkFuZSREaXN0YW5jZSkpDQpDb24uRGlzLkFuZSRBZ2VfRGlmZl90PC1zY2FsZShib3hjb3goQ29uLkRpcy5BbmUkQWdlX0RpZmYpKQ0KQ29uLkRpcy5BbmUkQWdlX3lvdW5nZXJfdDwtc2NhbGUoYm94Y294KENvbi5EaXMuQW5lJEFnZV9hYnNfeSkpDQoNCmNnZC5hbmU8LWxtZShDb24uRF90fmdlb2Rpc3RfdCtBZ2VfRGlmZl90K0FnZV95b3VuZ2VyX3QrZ2VvZGlzdF90OkFnZV9EaWZmX3QrZ2VvZGlzdF90OkFnZV95b3VuZ2VyX3QscmFuZG9tPX4xfFJlZ2lvbixjb3JyZWxhdGlvbj1jb3JNTFBFKGZvcm09fklOX1BPUC55K05FQVJfUE9QLnl8UmVnaW9uKSxkYXRhPUNvbi5EaXMuQW5lKQ0KcXFub3JtKGNnZC5hbmUsfnJlc2lkKC4sIHR5cGU9InAiKSxhYmxpbmU9YygwLDEpKQ0Kc3VtbWFyeShjZ2QuYW5lKQ0Kci5zcXVhcmVkR0xNTShjZ2QuYW5lKQ0KDQoNCg0KYGBgDQpgYGB7cn0NCiMjIE94YWxpcyBhY2V0b3NlbGxhDQpkaXN0b3hhPC1zdWJzZXQoZGlzdCxkaXN0JFNwZWNpZXMueD09Ik94YSIpDQpkaXN0b3hhJERpc3RhbmNlX3Q8LXNjYWxlKGJveGNveChkaXN0b3hhJERpc3RhbmNlLG1ldGhvZD0icGMiKSkNCmRpc3RveGEkQWdlX0RpZmZfdDwtc2NhbGUoYm94Y294KGRpc3RveGEkQWdlX0RpZmYpKSAjIyBhZ2UgZWxlbWVudCBvbmx5IHNjYWxlZCBidXQgbm90IGJveGNveCB0cmFuc2Zvcm1lZA0KZGlzdG94YSRBZ2VfeW91bmdlcl90PC1zY2FsZShib3hjb3goZGlzdG94YSRBZ2VfYWJzX3kpKQ0KDQpHc3Qub3hhPC1sbWUoR3N0X3R+RGlzdGFuY2VfdCtBZ2VfRGlmZl90K0FnZV95b3VuZ2VyX3QrRGlzdGFuY2VfdDpBZ2VfRGlmZl90K0Rpc3RhbmNlX3Q6QWdlX3lvdW5nZXJfdCxyYW5kb209fjF8UmVnaW9uLGNvcnJlbGF0aW9uID0gY29yTUxQRShmb3JtPX5JTl9QT1AueStORUFSX1BPUC55fFJlZ2lvbiksZGF0YT1kaXN0b3hhKQ0Kc3VtbWFyeShHc3Qub3hhKQ0Kci5zcXVhcmVkR0xNTShHc3Qub3hhKQ0KRHBzLm94YTwtbG1lKERwc190fkRpc3RhbmNlX3QrQWdlX0RpZmZfdCtBZ2VfeW91bmdlcl90K0Rpc3RhbmNlX3Q6QWdlX0RpZmZfdCtEaXN0YW5jZV90OkFnZV95b3VuZ2VyX3QscmFuZG9tID0gfjF8UmVnaW9uLGNvcnJlbGF0aW9uPWNvck1MUEUoZm9ybT1+SU5fUE9QLnkrTkVBUl9QT1AueXxSZWdpb24pLGRhdGE9ZGlzdG94YSkNCnN1bW1hcnkoRHBzLm94YSkNCnIuc3F1YXJlZEdMTU0oRHBzLm94YSkNCg0KQ29uLkRpcy5PeGE8LUNvbi5EaXNbYyg4MzoxMzQpLF0NCkNvbi5EaXMuT3hhJGdlb2Rpc3RfdDwtc2NhbGUoYm94Y294KENvbi5EaXMuT3hhJERpc3RhbmNlKSkNCkNvbi5EaXMuT3hhJEFnZV9EaWZmX3Q8LXNjYWxlKGJveGNveChDb24uRGlzLk94YSRBZ2VfRGlmZikpDQpDb24uRGlzLk94YSRBZ2VfeW91bmdlcl90PC1zY2FsZShib3hjb3goQ29uLkRpcy5PeGEkQWdlX2Fic195KSkNCg0KDQpjZ2Qub3hhPC1sbWUoQ29uLkRfdH5nZW9kaXN0X3QrQWdlX0RpZmZfdCtBZ2VfeW91bmdlcl90K2dlb2Rpc3RfdDpBZ2VfRGlmZl90K2dlb2Rpc3RfdDpBZ2VfeW91bmdlcl90LHJhbmRvbT1+MXxSZWdpb24sY29ycmVsYXRpb249Y29yTUxQRShmb3JtPX5JTl9QT1AueStORUFSX1BPUC55fFJlZ2lvbiksZGF0YT1Db24uRGlzLk94YSkNCnFxbm9ybShjZ2Qub3hhLH5yZXNpZCguLCB0eXBlPSJwIiksYWJsaW5lPWMoMCwxKSkNCnN1bW1hcnkoY2dkLm94YSkNCnIuc3F1YXJlZEdMTU0oY2dkLm94YSkNCg0KDQpgYGANCmBgYHtyfQ0KIyMgUG9seWdvbmF0dW0gbXVsdGlmbG9ydW0NCmRpc3Rwb2w8LXN1YnNldChkaXN0LGRpc3QkU3BlY2llcy54PT0iUG9sIikNCmRpc3Rwb2wkRGlzdGFuY2VfdDwtc2NhbGUoYm94Y294KGRpc3Rwb2wkRGlzdGFuY2UsbWV0aG9kPSJwYyIpKQ0KZGlzdHBvbCRBZ2VfRGlmZl90PC1zY2FsZShib3hjb3goZGlzdHBvbCRBZ2VfRGlmZikpICMjIGFnZSBlbGVtZW50IG9ubHkgc2NhbGVkIGJ1dCBub3QgYm94Y294IHRyYW5zZm9ybWVkDQpkaXN0cG9sJEFnZV95b3VuZ2VyX3Q8LXNjYWxlKGJveGNveChkaXN0cG9sJEFnZV9hYnNfeSkpDQoNCkdzdC5wb2w8LWxtZShHc3RfdH5EaXN0YW5jZV90K0FnZV9EaWZmX3QrQWdlX3lvdW5nZXJfdCtEaXN0YW5jZV90OkFnZV9EaWZmX3QrRGlzdGFuY2VfdDpBZ2VfeW91bmdlcl90LHJhbmRvbT1+MXxSZWdpb24sY29ycmVsYXRpb249Y29yTUxQRShmb3JtPX5JTl9QT1AueStORUFSX1BPUC55fFJlZ2lvbiksZGF0YT1kaXN0cG9sKQ0Kc3VtbWFyeShHc3QucG9sKQ0Kci5zcXVhcmVkR0xNTShHc3QucG9sKQ0KRHBzLnBvbDwtbG1lKERwc190fkRpc3RhbmNlX3QrQWdlX0RpZmZfdCtBZ2VfeW91bmdlcl90K0Rpc3RhbmNlX3Q6QWdlX0RpZmZfdCtEaXN0YW5jZV90OkFnZV95b3VuZ2VyX3QscmFuZG9tPX4xfFJlZ2lvbixjb3JyZWxhdGlvbj1jb3JNTFBFKGZvcm09fklOX1BPUC55K05FQVJfUE9QLnl8UmVnaW9uKSxkYXRhPWRpc3Rwb2wpDQpzdW1tYXJ5KERwcy5wb2wpDQpyLnNxdWFyZWRHTE1NKERwcy5wb2wpDQoNCkNvbi5EaXMuUG9sPC1Db24uRGlzW2MoMTM1OjIwOCksXQ0KQ29uLkRpcy5Qb2wkZ2VvZGlzdF90PC1zY2FsZShib3hjb3goQ29uLkRpcy5Qb2wkRGlzdGFuY2UpKQ0KQ29uLkRpcy5Qb2wkQWdlX0RpZmZfdDwtc2NhbGUoYm94Y294KENvbi5EaXMuUG9sJEFnZV9EaWZmKSkNCkNvbi5EaXMuUG9sJEFnZV95b3VuZ2VyX3Q8LXNjYWxlKGJveGNveChDb24uRGlzLlBvbCRBZ2VfYWJzX3kpKQ0KDQoNCmNnZC5wb2w8LWxtZShDb24uRF90fmdlb2Rpc3RfdCtBZ2VfRGlmZl90K0FnZV95b3VuZ2VyX3QrZ2VvZGlzdF90OkFnZV9EaWZmX3QrZ2VvZGlzdF90OkFnZV95b3VuZ2VyX3QscmFuZG9tPX4xfFJlZ2lvbixjb3JyZWxhdGlvbj1jb3JNTFBFKGZvcm09fklOX1BPUC55K05FQVJfUE9QLnl8UmVnaW9uKSxkYXRhPUNvbi5EaXMuUG9sKQ0KcXFub3JtKGNnZC5wb2wsfnJlc2lkKC4sIHR5cGU9InAiKSxhYmxpbmU9YygwLDEpKQ0Kc3VtbWFyeShjZ2QucG9sKQ0Kci5zcXVhcmVkR0xNTShjZ2QucG9sKQ0KDQoNCmBgYA0KYGBge3J9DQojIyBMaW5rIGxldmVsIERJRkZfR0VOX0dFTw0KbG9hZCgiTGluay5SRGF0YSIpDQpMaW5rJERpZmZfR19QPC1hcy5udW1lcmljKExpbmskRGlmZl9HX1ApDQpMaW5rX2FuZTwtc3Vic2V0KExpbmssTGluayRzcGVjaWVzPT0iQW5lIikNCkxpbmtfYW5lJERpZmZfR19QX3Q8LXNjYWxlKExpbmtfYW5lJERpZmZfR19QKQ0KDQpMaW5rX294YTwtc3Vic2V0KExpbmssTGluayRzcGVjaWVzPT0iT3hhIikNCkxpbmtfb3hhJERpZmZfR19QX3Q8LXNjYWxlKExpbmtfb3hhJERpZmZfR19QKQ0KDQpMaW5rX3BvbDwtc3Vic2V0KExpbmssTGluayRzcGVjaWVzPT0iUG9sIikNCkxpbmtfcG9sJERpZmZfR19QX3Q8LXNjYWxlKExpbmtfcG9sJERpZmZfR19QKQ0KDQpMaW5rPC1yYmluZChMaW5rX2FuZSxMaW5rX294YSxMaW5rX3BvbCkNCg0KTGluayRBZ2VfRGlmZl90PC1zY2FsZShib3hjb3goTGluayRBZ2VfRGlmZikpDQpMaW5rPC1MaW5rJT4lDQogIG11dGF0ZShBZ2VfYWJzX3k9MjAyMC1BZ2VfeW91bmdlcikNCkxpbmskQWdlX3lvdW5nZXJfdDwtc2NhbGUoYm94Y294KExpbmskQWdlX2Fic195KSkNCg0KbGluay5sbTwtbG1lKERpZmZfR19QX3R+QWdlX0RpZmZfdCpBZ2VfeW91bmdlcl90KlNwZWNpZXMscmFuZG9tPX4xfExXLngsY29ycmVsYXRpb24gPSBjb3JNTFBFKGZvcm09fm5vZGVfMStub2RlXzJ8TFcueCksZGF0YT1MaW5rKQ0KZHJlZGdlKGxpbmsubG0sZml4ZWQ9flNwZWNpZXMsUkVNTD1GKQ0KDQpsaW5rLmZpbjwtbG1lKERpZmZfR19QX3R+QWdlX3lvdW5nZXJfdCtTcGVjaWVzLHJhbmRvbT1+MXxMVy54LGNvcnJlbGF0aW9uPWNvck1MUEUoZm9ybT1+bm9kZV8xK25vZGVfMnxMVy54KSxkYXRhPUxpbmspDQpwbG90KGxpbmsuZmluLGZvcm09cmVzaWQoLiwgdHlwZSA9ICJwIikgfiBmaXR0ZWQoLiksIGFibGluZT0wKQ0KcXFub3JtKGxpbmsuZmluLH5yZXNpZCguLCB0eXBlID0gInAiKSwgYWJsaW5lPWMoMCwxKSkNCnN1bW1hcnkobGluay5maW4pDQpsaW5rLmZpbjI8LWxtZShEaWZmX0dfUF90fkFnZV9EaWZmX3QrU3BlY2llcyxyYW5kb209fjF8TFcueCxjb3JyZWxhdGlvbj1jb3JNTFBFKGZvcm09fm5vZGVfMStub2RlXzJ8TFcueCksZGF0YT1MaW5rKQ0Kc3VtbWFyeShsaW5rLmZpbjIpDQpyLnNxdWFyZWRHTE1NKEdzdC5maW4pDQpgYGANCg0KYGBge3J9DQojIyBOb2RlIGxldmVsDQpsb2FkKCJOb2RlLlJEYXRhIikNCk5vZGVfYW5lPC1zdWJzZXQoTm9kZSxOb2RlJFNwZWNpZXM9PSJBbmUiKQ0KTm9kZV9veGE8LXN1YnNldChOb2RlLE5vZGUkU3BlY2llcz09Ik94YSIpDQpOb2RlX3BvbDwtc3Vic2V0KE5vZGUsTm9kZSRTcGVjaWVzPT0iUG9sIikNCg0KTm9kZV9hbmUkY2xvc2VuZXNzX3Q8LXNjYWxlKE5vZGVfYW5lJGNsb3NlbmVzcykNCk5vZGVfb3hhJGNsb3NlbmVzc190PC1zY2FsZShOb2RlX294YSRjbG9zZW5lc3MpDQpOb2RlX3BvbCRjbG9zZW5lc3NfdDwtc2NhbGUoTm9kZV9wb2wkY2xvc2VuZXNzKQ0KDQpOb2RlPC1yYmluZChOb2RlX2FuZSxOb2RlX294YSxOb2RlX3BvbCkNCk5vZGUkQWdlX3Q8LXNjYWxlKGJveGNveChOb2RlJEFnZV9hYnMpKSMjIGxhbWJkYT0wLjQ1LCBhZGRlZD0wDQpub2RlLmxtPC1sbWUoY2xvc2VuZXNzX3R+QWdlX3QqU3BlY2llcyxyYW5kb209fjF8TFcsZGF0YT1Ob2RlKQ0KZHJlZGdlKG5vZGUubG0sZml4ZWQ9flNwZWNpZXMsUkVNTD1GKQ0Kbm9kZS5maW48LWxtZShjbG9zZW5lc3NfdH5BZ2VfdCtTcGVjaWVzLHJhbmRvbT1+MXxMVyxkYXRhPU5vZGUpDQpzdW1tYXJ5KG5vZGUuZmluKQ0KcGxvdChub2RlLmZpbixmb3JtPXJlc2lkKC4sIHR5cGUgPSAicCIpIH4gZml0dGVkKC4pLCBhYmxpbmU9MCkNCnFxbm9ybShub2RlLmZpbix+cmVzaWQoLiwgdHlwZSA9ICJwIiksIGFibGluZT1jKDAsMSkpDQoNCmBgYA0KDQo=
