# Supplementary material for: Limited effects of population age on the genetic structure of spatially isolated forest herb populations in temperate Europe
Source: Ecol Evol. 2024 Feb 26;14(2):e10971. doi: 10.1002/ece3.10971 (PMC10897356; doi:10.1002/ece3.10971)
Supplement: Supplementary file 1 — Appendix S1. [file ECE3-14-e10971-s001.zip › 07_Result_Visualization.nb.html]

Result\_Visualization


Code 

- Show All Code
- Hide All Code
- Download Rmd

# Result\_Visualization


```
```r
library(dplyr)
library(effects)
library(scales)
library(plotly)
##Figure_1b
load(\GenDiv_all.RData\)

df<-GenDiv_all%>%
  select(population,Species.x,LW.x,Age_abs)%>%
  arrange(match(LW.x,c(\FrN\,\Be\,\GeW\,\GeE\,\Sw\,\Es\)))

df$LW<-as.numeric(factor(df$LW.x,levels=c(\FrN\,\Be\,\GeW\,\GeE\,\SwS\,\Est\)))


par(mfrow=c(1,3),bty=\L\,mar=c(4,4,2,0))
plot(3,type=\n\,xlim=c(0.5,6.5),ylim=c(0,350),xaxt='n',ann=F,yaxt='n')
axis(side=2,lwd=2,cex.axis=1.5)
axis(side=1,lwd=2,cex.axis=1.3,at=c(1,2,3,4,5,6),labels = c(\Fr\,\Be\,\GeW\,\GeE\,\Sw\,\Es\))
box(lwd=2)
mtext(adj=-0.1,~italic(\A. nemorosa\),cex=1)
title(xlab=c(\Sampling Site\),ylab=c(\Population Age (yr)\),line=2.5,cex.lab=1.5)
with(df[df$Species.x==\Ane\,], points(x=jitter(LW),y=Age_abs,cex=2,pch=0,lwd=2,
                                      col=c(\#bb5566\)))


plot(3,type=\n\,xlim=c(0.5,4.5),ylim=c(0,350),xaxt='n',ann=F,yaxt='n')
axis(side=2,lwd=2,cex.axis=1.5)
axis(side=1,lwd=2,cex.axis=1.3,at=c(1,2,3,4),labels = c(\GeW\,\GeE\,\Sw\,\Es\))
box(lwd=2)
mtext(adj=-0.1,~italic(\O. acetosella\),cex=1)
title(xlab=c(\Sampling Site\),ylab=c(\Population Age (yr)\),line=2.5,cex.lab=1.5)
with(df[df$Species.x==\Oxa\,], points(x=jitter(LW-2),y=Age_abs,cex=2,pch=1,lwd=2, 
                                      col=c(\#ddaa33\)))

plot(3,type=\n\,xlim=c(0.5,6.5),ylim=c(0,350),xaxt='n',ann=F,yaxt='n')
axis(side=2,lwd=2,cex.axis=1.5)
axis(side=1,lwd=2,cex.axis=1.3,at=c(1,2,3,4,5,6),labels = c(\Fr\,\Be\,\GeW\,\GeE\,\Sw\,\Es\))
box(lwd=2)
mtext(adj=-0.1,~italic(\P. multiflorum\),cex=1)
title(xlab=c(\Sampling Site\),ylab=c(\Population Age (yr)\),line=2.5,cex.lab=1.5)
with(df[df$Species.x==\Pol\,], points(x=jitter(LW),y=Age_abs,cex=1.5,pch=6,lwd=2, 
                                      col=c(\#004488\)))

##Figure_3
eff.Ar.Age<-effect(\Species.x:Age_t\,lmm.Ar.b.fin, partial.residuals=T)
plot.data.Age.all<-eff.Ar.Age$data
plot.data.Age.fit<-with(eff.Ar.Age, cbind(x,fit,lower,upper))

par(mfrow=c(1,4),mar=c(4,5,2,2))
plot(3,type=\n\,xlim=c(-2,2.5),ylim=c(-3,3.2),xaxt='n',ann=F,yaxt='n')
axis(side=2,lwd=2,cex.axis=2)
axis(side=1,lwd=2,cex.axis=2,at=c(-1.16596,0.159139,1.037124,1.736027),labels = c(50,150,250,350))
box(lwd=2)
#mtext(c(\O\,\Y\),at=c(1.05,2.05),side=1)
title(xlab=c(\POP_AGE (yr)\),ylab=expression(italic(H)[e]),line=2.5,cex.lab=2)
with(plot.data.Age.fit[plot.data.Age.fit$Species.x==\Ane\,],polygon(x=c(Age_t[Species.x==\Ane\],rev(Age_t[Species.x==\Ane\])),y=c(lower[Species.x==\Ane\], rev(upper[Species.x==\Ane\])),col=alpha(\#bb5566\,0.3), border=NA)) 

with(GenDiv_all[GenDiv_all$Species.x==\Ane\,], points(x=jitter(Age_t),y=He_t,cex=2,pch=21,lwd=2,
                                                      bg=c(\#bb5566\)))

with(GenDiv_all[GenDiv_all$Species.x==\Oxa\,], points(x=jitter(Age_t),y=He_t,cex=2,pch=21,lwd=2, 
                                                      bg=c(\#ddaa33\)))
with(GenDiv_all[GenDiv_all$Species.x==\Pol\,], points(x=jitter(Age_t),y=He_t,cex=2,pch=21,lwd=2, 
                                                      bg=c(\#004488\)))

with(plot.data.Age.fit[plot.data.Age.fit$Species.x==\Ane\,],lines(Age_t,fit,col=c(\#bb5566\),lwd=2))
with(plot.data.Age.fit[plot.data.Age.fit$Species.x==\Oxa\,],lines(Age_t,fit,col=c(\#ddaa33\),lwd=2,lty=5))
with(plot.data.Age.fit[plot.data.Age.fit$Species.x==\Pol\,],lines(Age_t,fit,col=c(\#004488\),lwd=2,lty=5))

with(plot.data.Age.fit, text(2.2, y=c(min(fit[Species.x==\Ane\]),
                                      max(fit[Species.x==\Oxa\]),
                                      max(fit[Species.x==\Pol\])+0.5),
                             labels=c(\a\, \b\,\b\), cex=1.5, adj=1))

mtext(adj=-0.1,\(A)\,cex=1.5)
graphics::legend(0.8,3.6,legend=expression(italic(A.nem.),italic(O.ace.),italic(P.mul.),\p > 0.05\,\p\<=\0.05\),bty='n',
                 pch=c(21,21,21,NA,NA),pt.bg=c(\#bb5566\,\#ddaa33\,\#004488\,NA,NA),lty=c(NA,NA,NA,5,1),
                 seg.len=0.5,cex=1.5,x.intersp=0.1,y.intersp=0.6)


eff.He.Age<-effect(\Age_t:Species.x\,lmm.He.b.fin, partial.residuals=T)
plot.data.Age.all<-eff.He.Age$data
plot.data.Age.fit<-with(eff.He.Age,cbind(x,fit,lower,upper))

par(mfrow=c(1,3), mar=c(4,5,2,2))

plot(3,type=\n\,xlim=c(-2,2.5),ylim=c(-3,3.2),xaxt='n',ann=F,yaxt='n')
axis(side=2,lwd=2,cex.axis=2)
axis(side=1,lwd=2,cex.axis=2,at=c(-1.16596,0.159139,1.037124,1.736027),labels = c(50,150,250,350))
box(lwd=2)
#mtext(c(\O\,\Y\),at=c(1.05,2.05),side=1)
title(xlab=c(\POP_AGE (yr)\),ylab=expression(italic(H)[e]),line=2.5,cex.lab=2)
with(plot.data.Age.fit[plot.data.Age.fit$Species.x==\Ane\,],polygon(x=c(Age_t[Species.x==\Ane\],rev(Age_t[Species.x==\Ane\])),y=c(lower[Species.x==\Ane\], rev(upper[Species.x==\Ane\])),col=alpha(\#bb5566\,0.3), border=NA)) 

with(GenDiv_all[GenDiv_all$Species.x==\Ane\,], points(x=jitter(Age_t),y=He_t,cex=2,pch=21,lwd=2,
                                                      bg=c(\#bb5566\)))

with(GenDiv_all[GenDiv_all$Species.x==\Oxa\,], points(x=jitter(Age_t),y=He_t,cex=2,pch=21,lwd=2, 
                                                      bg=c(\#ddaa33\)))
with(GenDiv_all[GenDiv_all$Species.x==\Pol\,], points(x=jitter(Age_t),y=He_t,cex=2,pch=21,lwd=2, 
                                                      bg=c(\#004488\)))

with(plot.data.Age.fit[plot.data.Age.fit$Species.x==\Ane\,],lines(Age_t,fit,col=c(\#bb5566\),lwd=2))
with(plot.data.Age.fit[plot.data.Age.fit$Species.x==\Oxa\,],lines(Age_t,fit,col=c(\#ddaa33\),lwd=2,lty=5))
with(plot.data.Age.fit[plot.data.Age.fit$Species.x==\Pol\,],lines(Age_t,fit,col=c(\#004488\),lwd=2,lty=5))

with(plot.data.Age.fit, text(2.2, y=c(min(fit[Species.x==\Ane\]),
                                      max(fit[Species.x==\Oxa\]),
                                      max(fit[Species.x==\Pol\])+0.5),
                             labels=c(\a\, \b\,\b\), cex=1.5, adj=1))

mtext(adj=-0.1,\(A)\,cex=1.5)
graphics::legend(0.8,3.6,legend=expression(italic(A.nem.),italic(O.ace.),italic(P.mul.),\p > 0.05\,\p\<=\0.05\),bty='n',
                 pch=c(21,21,21,NA,NA),pt.bg=c(\#bb5566\,\#ddaa33\,\#004488\,NA,NA),lty=c(NA,NA,NA,5,1),
                 seg.len=0.5,cex=1.5,x.intersp=0.1,y.intersp=0.6)


eff.Ho.Age<-effect(\Age_t\,lmm.Ho.b.fin, partial.residuals=T)
plot.data.Age.all<-eff.Ho.Age$data
plot.data.Age.fit<-with(eff.Ho.Age,cbind(x,fit,lower,upper))

plot(3,type=\n\,xlim=c(-2,2.5),ylim=c(-3,3.2),xaxt='n',ann=F,yaxt='n')
axis(side=2,lwd=2,cex.axis=2)
axis(side=1,lwd=2,cex.axis=2,at=c(-1.16596,0.159139,1.037124,1.736027),labels = c(50,150,250,350))
box(lwd=2)
title(xlab=c(\POP_AGE (year)\),ylab=expression(italic(H)[o]),line=2.5,cex.lab=2)
with(plot.data.Age.fit,polygon(x=c(Age_t, rev(Age_t)),y=c(lower, rev(upper)),
                               col=c(\#dcdcdc\), border=NA)) 

with(GenDiv_all[GenDiv_all$Species.x==\Ane\,], points(x=jitter(Age_t),y=Ho_t,pch=21,cex=2,lwd=2,
                                                      bg=c(\#bb5566\)))

with(GenDiv_all[GenDiv_all$Species.x==\Oxa\,], points(x=jitter(Age_t),y=Ho_t,pch=21,cex=2,lwd=2, 
                                                      bg=c(\#ddaa33\)))
with(GenDiv_all[GenDiv_all$Species.x==\Pol\,], points(x=jitter(Age_t),y=Ho_t,pch=21,cex=2,lwd=2, 
                                                      bg=c(\#004488\)))

with(plot.data.Age.fit,lines(Age_t,fit,col=rgb(0,0,0,maxColorValue = 255),lwd=2))

mtext(adj=-0.1,\(B)\,cex=1.5)

eff.Fis.Age<-effect(\Age_t\,lmm.Fis.b.fin, partial.residuals=T)
plot.data.Age.all<-eff.Fis.Age$data
plot.data.Age.fit<-with(eff.Fis.Age,cbind(x,fit,lower,upper))

plot(3,type=\n\,xlim=c(-2,2.5),ylim=c(-3,3.2),xaxt='n',ann=F,yaxt='n')
axis(side=2,lwd=2,cex.axis=2)
axis(side=1,lwd=2,cex.axis=2,at=c(-1.16596,0.159139,1.037124,1.736027),labels = c(50,150,250,350))
box(lwd=2)
title(xlab=c(\POP_AGE (year)\),ylab=expression(italic(F)),line=2.5,cex.lab=2)
with(plot.data.Age.fit,polygon(x=c(Age_t, rev(Age_t)),y=c(lower, rev(upper)),
                               col=c(\#dcdcdc\), border=NA)) 

with(GenDiv_all[GenDiv_all$Species.x==\Ane\,], points(x=jitter(Age_t),y=Fis_t,pch=21,cex=2,lwd=2,
                                                      bg=c(\#bb5566\)))

with(GenDiv_all[GenDiv_all$Species.x==\Oxa\,], points(x=jitter(Age_t),y=Fis_t,pch=21,cex=2,lwd=2, 
                                                      bg=c(\#ddaa33\)))
with(GenDiv_all[GenDiv_all$Species.x==\Pol\,], points(x=jitter(Age_t),y=Fis_t,pch=21,cex=2,lwd=2, 
                                                      bg=c(\#004488\)))

with(plot.data.Age.fit,lines(Age_t,fit,col=rgb(0,0,0,maxColorValue = 255),lwd=2))
mtext(adj=-0.1,\(C)\,cex=1.5)
```

```
<!-- rnb-source-end -->

<!-- rnb-chunk-end -->


<!-- rnb-text-begin -->


<!-- rnb-text-end -->


<!-- rnb-chunk-begin -->


<!-- rnb-source-begin eyJkYXRhIjoiYGBgclxuYGBgclxuYGBgclxuYGBgclxuIyNGaWd1cmVfNFxubGlicmFyeShwdGlucG9seSlcbmxpYnJhcnkocGxvdGx5KVxubiA8LSAxMDAgI251bWJlciBvZiBncmlkIHBvaW50cyB0byBiZSB1c2VkIGFsb25nIGVhY2ggYXhpc1xuZi5heCA8LSBsaXN0KGZhbWlseT1cXEFyaWFsXFwsIHNpemU9NDgpICNmb250IGZvciB0aWNrIGxhYmVscyAtLT4gdW5pdCB1bmNsZWFyIC0tPiBtdXN0IGJlIGxhcmdlciB3aGVuIGZpZ3VyZSBzaXplIGluIHB4IGlzIGxhcmdlclxuZi5sYWIgPC0gbGlzdChmYW1pbHk9XFxBcmlhbFxcLCBzaXplPTU2KSAjZm9udCBmb3IgYXhlcyBsYWJlbHNcbmYuYW5uIDwtIGxpc3QoZmFtaWx5PVxcQXJpYWxcXCwgc2l6ZT00OCwgY29sb3I9XFxzdGVlbGJsdWVcXClcblxuY3IgPC0gY29sb3JSYW1wKGNvbG9ycz1jKFxcIzM2NEI5QVxcLCBcXCM0QTdCQjdcXCwgXFwjNkVBNkNEXFwsIFxcIzk4Q0FFMVxcLCBcXCNDMkU0RUZcXCwgXFwjRUFFQ0NDXFwsIFxcI0ZFREE4QlxcLCBcXCNGREIzNjZcXCxcbiAgICAgICAgICAgICAgICAgICAgICAgICBcXCNGNjdFNEJcXCwgXFwjREQzRDJEXFwsIFxcI0E1MDAyNlxcKSkgIy0tPiBjb2xvciBzY2FsZSBcXHN1bnNldFxcIGRlc2lnbmVkIGJ5IFBhdWwgVG9sIGZvciBjb2xvcmJsaW5kIHBlb3BsZTogaHR0cHM6Ly9wZXJzb25hbC5zcm9uLm5sL35wYXVsdC9cblxuIyMjIyMjIyMjIyMjI094YSMjIyMjIyMjIyMjIyMjIyMjXG5cbiMjIyMjIyMgSGUjIyMjIyMjIyMjIyMjXG50ZXN0PC1hcy5kYXRhLmZyYW1lKG1vZGVsLm1hdHJpeChsbW0uSGUub3hhKSlcblxueCA8LSB3aXRoKHRlc3QsIHNlcShtaW4oQWdlX3QpLCBtYXgoQWdlX3QpLCBsZW5ndGgub3V0PW4pKVxueSA8LSB3aXRoKHRlc3QsIHNlcShtaW4oSUZNX3RyYW5zZm9ybWVkKSwgbWF4KElGTV90cmFuc2Zvcm1lZCksIGxlbmd0aC5vdXQ9bikpXG56IDwtIG1hdHJpeChucm93PW4sIG5jb2w9bikgI2NvbnRvdXIgaW50ZXJwcmV0cyB0aGUgeiBtYXRyaXggYXMgYSB0YWJsZSBvZiBmKHhbaV0sIHlbal0pIHZhbHVlcywgc28gdGhhdCB0aGUgeCBheGlzIGNvcnJlc3BvbmRzIHRvIHJvdyBudW1iZXIgYW5kIHRoZSB5IGF4aXMgdG8gY29sdW1uIG51bWJlciwgd2l0aCBjb2x1bW4gMSBhdCB0aGUgYm90dG9tLCBpLmUuIGEgOTAgZGVncmVlIGNvdW50ZXItY2xvY2t3aXNlIHJvdGF0aW9uIG9mIHRoZSBjb252ZW50aW9uYWwgdGV4dHVhbCBsYXlvdXQuXG5uZXdkYXQxIDwtIGRhdGEuZnJhbWUoUG9wU2l6ZV90cmFuc2Zvcm1lZD1yZXAobWVkaWFuKHRlc3QkUG9wU2l6ZV90cmFuc2Zvcm1lZCksbiksXG4gICAgICAgICAgICAgICAgICAgICAgSUZNX3RyYW5zZm9ybWVkPXkpXG5mb3IoaSBpbiAxOm4pIHtcbiAgbmV3ZGF0MiA8LSBuZXdkYXQxXG4gIG5ld2RhdDIkQWdlX3QgPC0gcmVwKHhbaV0sbilcbiAgeltpLF0gPC0gcHJlZGljdChsbW0uSGUub3hhLCBuZXdkYXRhPW5ld2RhdDIsIGxldmVsPTApICNlYWNoIHJvdyBhcyBhIGNvbnN0YW50IHggdmFsdWUsIGJ1dCBhIGNoYW5naW5nIHkgdmFsdWVcbn1cblxuY2xvdWQgPC0gdGVzdFssYyhcXEFnZV90XFwsXFxJRk1fdHJhbnNmb3JtZWRcXCldICN4IGFuZCB5IGNvb3JpZG5hdGVzIG9mIGEgcG9pbnQgY2xvdWRcbmh1bGwgPC0gYXMubWF0cml4KGNsb3VkW2NodWxsKGNsb3VkKSxdKSAjY29vcmRpbmF0ZXMgb2YgdGhlIGNvbnZleCBodWxsIHZlcnRpY2VzIChjaHVsbCByZXR1cm5zIHRoZSBpbmRpY2VzIG9mIHRoZSB2ZXJ0ZXggcG9pbnRzKVxuY2hlY2sgPC0gYXMubWF0cml4KGRhdGEuZnJhbWUoeD1yZXAoeCwgZWFjaD1uKSwgeT1yZXAoeSwgdGltZXM9bikpKSAgI1AtYnktMiBtYXRyaXggd2l0aCBwb2ludHMgdG8gYmUgY2hlY2tlZFxuXG5wb2ludC54PC1jaGVja1ssMV1cbnBvaW50Lnk8LWNoZWNrWywyXVxueF9hcnJheTwtaHVsbFssMV1cbnlfYXJyYXk8LWh1bGxbLDJdXG53aXRoaW48LXNwOjpwb2ludC5pbi5wb2x5Z29uKHBvaW50LngscG9pbnQueSx4X2FycmF5LHlfYXJyYXkpIyMgMDogcG9pbnQgaXMgb3V0IG9mIHRoZSBwb2x5Z29uLCAxOiBwb2ludCBpcyBpbiB0aGUgcG9seWdvbiwgMjogcG9pbnQgaXMgb24gdGhlIGxpbmVcbnVuaXF1ZSh3aXRoaW4pXG4jLS0+IG5vIGVycm9yIGNvZGVzXG53aXRoaW4gPC0gaWZlbHNlKHdpdGhpbj49MSwgVFJVRSwgRkFMU0UpXG5wbG90KGNoZWNrWywxXSwgY2hlY2tbLDJdLCBwY2g9MTYsIGNvbD1pZmVsc2Uod2l0aGluLFxccmVkXFwsIFxcd2hpdGVcXCkpXG53aXRoaW4gPC0gbWF0cml4KHdpdGhpbiwgbnJvdz1uLCBuY29sPW4sIGJ5cm93PVRSVUUpXG56WyF3aXRoaW5dIDwtIE5BICNzZXQgYWxsIHotdmFsdWVzIG91dHNpZGUgdGhlIGNvbnZleCBodWxsIHRvIE5BXG5cbnR6IDwtIHQoeikgI2JlY2F1c2UgcGxvdGx5IGludGVycHJldHMgeiBhcyB0KHopOyBhbHRlcm5hdGl2ZWx5LCB0aGUgYXJndW1lbnQgdHJhbnNwb3NlIGNhbiBiZSBzZXQgdG8gVFJVRSBpbiBwbG90X2x5KClcbmZpZzRhIDwtIHBsb3RfbHkoeD14LCB5PXksIHo9dHosIHR5cGU9XFxjb250b3VyXFwsXG4gICAgICAgICAgICAgICAgIGNvbG9ycz1jcixcbiAgICAgICAgICAgICAgICAgYXV0b2NvbnRvdXI9RiwgY29udG91cnM9bGlzdChzdGFydD1taW4oeixuYS5ybT1UKSxlbmQ9bWF4KHosbmEucm09VCksc2l6ZT0wLjEpLFxuICAgICAgICAgICAgICAgICB3aWR0aD0xMzMwLCBoZWlnaHQ9MTMzMC0xMzMwLzYpICAgI3NldCBwbG90IHdpZHRoIGFuZCBoZWlnaHQgaW4gcHhcbmZpZzRhIDwtIGZpZzRhICU+JSBjb2xvcmJhcihsZW49MSwgdGl0bGU9XFw8aT5IPFwvaT48c3ViPmVcXCwgdGl0bGVmb250PWYubGFiLCB0aWNrZm9udD1mLmF4LCB0aWNrMD0tMS41LCBkdGljaz0wLjUpIyBleHByZXNzaW9uKERbZXN0XSkgZG9lcyBub3Qgd29yay4uLlxuZmlnNGEgPC0gZmlnNGEgJT4lIGFkZF9tYXJrZXJzKHg9Y2xvdWQkQWdlX3QsIHk9Y2xvdWQkSUZNX3RyYW5zZm9ybWVkLCBpbmhlcml0PUZBTFNFLCBzaG93bGVnZW5kPUZBTFNFLFxuICAgICAgICAgICAgICAgICAgICAgICAgICAgICAgIG1hcmtlcj1saXN0KHN5bWJvbD1cXGNpcmNsZS1vcGVuXFwsIGNvbG9yPVxcYmxhY2tcXCwgc2l6ZT0xNikpXG5maWc0YSA8LSBmaWc0YSAlPiUgbGF5b3V0KHhheGlzPWxpc3QodGl0bGU9XFxQT1BfQUdFXFwsIHRpdGxlZm9udD1mLmxhYiwgdGlja2ZvbnQ9Zi5heCwgc2hvd2dyaWQ9RkFMU0UsIHplcm9saW5lPUZBTFNFLFxuICAgICAgICAgICAgICAgICAgICAgICAgICAgICAgICAgICAgIHNob3dsaW5lPVRSVUUsIHRpY2tsZW49MTUsIHRpY2t3aWR0aD0zLCBsaW5ld2lkdGg9MyksICAgICAgICAgICAjYWRkIGF4aXMgbGFiZWxzXG4gICAgICAgICAgICAgICAgICAgICAgICAgIHlheGlzPWxpc3QodGl0bGU9XFxTUEFfQ09OXFwsIHRpdGxlZm9udD1mLmxhYiwgdGlja2ZvbnQ9Zi5heCwgc2hvd2dyaWQ9RkFMU0UsIHplcm9saW5lPUZBTFNFLFxuICAgICAgICAgICAgICAgICAgICAgICAgICAgICAgICAgICAgIHNob3dsaW5lPVRSVUUsIHRpY2tsZW49MTUsIHRpY2t3aWR0aD0zLCBsaW5ld2lkdGg9MyksXG4gICAgICAgICAgICAgICAgICAgICAgICAgIG1hcmdpbj1saXN0KGw9NjAsIHI9ODAsIHQ9MjAsIGI9NjApKSAgICAgICAgICAgICAgICAgICAgICAgICAgIFxuZmlnNGFcblxuIyMjIyMgSG8jIyMjXG50ZXN0PC1hcy5kYXRhLmZyYW1lKG1vZGVsLm1hdHJpeChsbW0uSG8ub3hhKSlcbnggPC0gd2l0aCh0ZXN0LCBzZXEobWluKEFnZV90KSwgbWF4KEFnZV90KSwgbGVuZ3RoLm91dD1uKSlcbnkgPC0gd2l0aCh0ZXN0LCBzZXEobWluKFBvcFNpemVfdHJhbnNmb3JtZWQpLCBtYXgoUG9wU2l6ZV90cmFuc2Zvcm1lZCksIGxlbmd0aC5vdXQ9bikpXG56IDwtIG1hdHJpeChucm93PW4sIG5jb2w9bikgI2NvbnRvdXIgaW50ZXJwcmV0cyB0aGUgeiBtYXRyaXggYXMgYSB0YWJsZSBvZiBmKHhbaV0sIHlbal0pIHZhbHVlcywgc28gdGhhdCB0aGUgeCBheGlzIGNvcnJlc3BvbmRzIHRvIHJvdyBudW1iZXIgYW5kIHRoZSB5IGF4aXMgdG8gY29sdW1uIG51bWJlciwgd2l0aCBjb2x1bW4gMSBhdCB0aGUgYm90dG9tLCBpLmUuIGEgOTAgZGVncmVlIGNvdW50ZXItY2xvY2t3aXNlIHJvdGF0aW9uIG9mIHRoZSBjb252ZW50aW9uYWwgdGV4dHVhbCBsYXlvdXQuXG5uZXdkYXQxIDwtIGRhdGEuZnJhbWUoSUZNX3RyYW5zZm9ybWVkPXJlcChtZWRpYW4odGVzdCRJRk1fdHJhbnNmb3JtZWQpLG4pLFxuICAgICAgICAgICAgICAgICAgICAgIFBvcFNpemVfdHJhbnNmb3JtZWQ9eSlcbmZvcihpIGluIDE6bikge1xuICBuZXdkYXQyIDwtIG5ld2RhdDFcbiAgbmV3ZGF0MiRBZ2VfdCA8LSByZXAoeFtpXSxuKVxuICB6W2ksXSA8LSBwcmVkaWN0KGxtbS5Iby5veGEsIG5ld2RhdGE9bmV3ZGF0MiwgbGV2ZWw9MCkgI2VhY2ggcm93IGFzIGEgY29uc3RhbnQgeCB2YWx1ZSwgYnV0IGEgY2hhbmdpbmcgeSB2YWx1ZVxufVxuXG5jbG91ZCA8LSB0ZXN0WyxjKFxcQWdlX3RcXCxcXFBvcFNpemVfdHJhbnNmb3JtZWRcXCldICN4IGFuZCB5IGNvb3JpZG5hdGVzIG9mIGEgcG9pbnQgY2xvdWRcbmh1bGwgPC0gYXMubWF0cml4KGNsb3VkW2NodWxsKGNsb3VkKSxdKSAjY29vcmRpbmF0ZXMgb2YgdGhlIGNvbnZleCBodWxsIHZlcnRpY2VzIChjaHVsbCByZXR1cm5zIHRoZSBpbmRpY2VzIG9mIHRoZSB2ZXJ0ZXggcG9pbnRzKVxuY2hlY2sgPC0gYXMubWF0cml4KGRhdGEuZnJhbWUoeD1yZXAoeCwgZWFjaD1uKSwgeT1yZXAoeSwgdGltZXM9bikpKSAgI1AtYnktMiBtYXRyaXggd2l0aCBwb2ludHMgdG8gYmUgY2hlY2tlZFxuXG5wb2ludC54PC1jaGVja1ssMV1cbnBvaW50Lnk8LWNoZWNrWywyXVxueF9hcnJheTwtaHVsbFssMV1cbnlfYXJyYXk8LWh1bGxbLDJdXG53aXRoaW48LXNwOjpwb2ludC5pbi5wb2x5Z29uKHBvaW50LngscG9pbnQueSx4X2FycmF5LHlfYXJyYXkpIyMgMDogcG9pbnQgaXMgb3V0IG9mIHRoZSBwb2x5Z29uLCAxOiBwb2ludCBpcyBpbiB0aGUgcG9seWdvbiwgMjogcG9pbnQgaXMgb24gdGhlIGxpbmVcbnVuaXF1ZSh3aXRoaW4pXG4jLS0+IG5vIGVycm9yIGNvZGVzXG53aXRoaW4gPC0gaWZlbHNlKHdpdGhpbj49MSwgVFJVRSwgRkFMU0UpXG5wbG90KGNoZWNrWywxXSwgY2hlY2tbLDJdLCBwY2g9MTYsIGNvbD1pZmVsc2Uod2l0aGluLFxccmVkXFwsIFxcd2hpdGVcXCkpXG53aXRoaW4gPC0gbWF0cml4KHdpdGhpbiwgbnJvdz1uLCBuY29sPW4sIGJ5cm93PVRSVUUpXG56WyF3aXRoaW5dIDwtIE5BICNzZXQgYWxsIHotdmFsdWVzIG91dHNpZGUgdGhlIGNvbnZleCBodWxsIHRvIE5BXG5cbnR6IDwtIHQoeikgI2JlY2F1c2UgcGxvdGx5IGludGVycHJldHMgeiBhcyB0KHopOyBhbHRlcm5hdGl2ZWx5LCB0aGUgYXJndW1lbnQgdHJhbnNwb3NlIGNhbiBiZSBzZXQgdG8gVFJVRSBpbiBwbG90X2x5KClcbmZpZzRiIDwtIHBsb3RfbHkoeD14LCB5PXksIHo9dHosIHR5cGU9XFxjb250b3VyXFwsXG4gICAgICAgICAgICAgICAgIGNvbG9ycz1jcixcbiAgICAgICAgICAgICAgICAgYXV0b2NvbnRvdXI9RiwgY29udG91cnM9bGlzdChzdGFydD1taW4oeixuYS5ybT1UKSxlbmQ9bWF4KHosbmEucm09VCksc2l6ZT0wLjEpLFxuICAgICAgICAgICAgICAgICB3aWR0aD0xMzMwLCBoZWlnaHQ9MTMzMC0xMzMwLzYpICAgI3NldCBwbG90IHdpZHRoIGFuZCBoZWlnaHQgaW4gcHhcbmZpZzRiIDwtIGZpZzRiICU+JSBjb2xvcmJhcihsZW49MSwgdGl0bGU9XFw8aT5IPFwvaT48c3ViPm9cXCwgdGl0bGVmb250PWYubGFiLCB0aWNrZm9udD1mLmF4LCB0aWNrMD0tMS41LCBkdGljaz0wLjUpIyBleHByZXNzaW9uKERbZXN0XSkgZG9lcyBub3Qgd29yay4uLlxuZmlnNGIgPC0gZmlnNGIgJT4lIGFkZF9tYXJrZXJzKHg9Y2xvdWQkQWdlX3QsIHk9Y2xvdWQkUG9wU2l6ZV90cmFuc2Zvcm1lZCwgaW5oZXJpdD1GQUxTRSwgc2hvd2xlZ2VuZD1GQUxTRSxcbiAgICAgICAgICAgICAgICAgICAgICAgICAgICAgICBtYXJrZXI9bGlzdChzeW1ib2w9XFxjaXJjbGUtb3BlblxcLCBjb2xvcj1cXGJsYWNrXFwsIHNpemU9MTYpKVxuZmlnNGIgPC0gZmlnNGIgJT4lIGxheW91dCh4YXhpcz1saXN0KHRpdGxlPVxcUE9QX0FHRVxcLCB0aXRsZWZvbnQ9Zi5sYWIsIHRpY2tmb250PWYuYXgsIHNob3dncmlkPUZBTFNFLCB6ZXJvbGluZT1GQUxTRSxcbiAgICAgICAgICAgICAgICAgICAgICAgICAgICAgICAgICAgICBzaG93bGluZT1UUlVFLCB0aWNrbGVuPTE1LCB0aWNrd2lkdGg9MywgbGluZXdpZHRoPTMpLCAgICAgICAgICAgI2FkZCBheGlzIGxhYmVsc1xuICAgICAgICAgICAgICAgICAgICAgICAgICB5YXhpcz1saXN0KHRpdGxlPVxcUE9QX1NJWkVcXCwgdGl0bGVmb250PWYubGFiLCB0aWNrZm9udD1mLmF4LCBzaG93Z3JpZD1GQUxTRSwgemVyb2xpbmU9RkFMU0UsXG4gICAgICAgICAgICAgICAgICAgICAgICAgICAgICAgICAgICAgc2hvd2xpbmU9VFJVRSwgdGlja2xlbj0xNSwgdGlja3dpZHRoPTMsIGxpbmV3aWR0aD0zKSxcbiAgICAgICAgICAgICAgICAgICAgICAgICAgbWFyZ2luPWxpc3QobD02MCwgcj04MCwgdD0yMCwgYj02MCkpICAgICAgICAgICAgICAgICAgICAgICAgICAgICNzZXRzIHRoZSBwbG90IG1hcmdpbiBpbiBweCwgc2VlIGh0dHBzOi8vcGxvdGx5LmNvbS9yL3JlZmVyZW5jZS9sYXlvdXQvI2xheW91dC1hdXRvc2l6ZVxuZmlnNGJcbiMjIyMjIyMjIyMjIyNQb2wjIyMjIyMjIyMjIyMjIyMjI1xudGVzdDwtYXMuZGF0YS5mcmFtZShtb2RlbC5tYXRyaXgobG1tLkhlLnBvbCkpXG5cbiMjIyMjIyMjIyMjIyMjIyNwb3BzaXplJiBIZSMjIyMjIyMjIyMjIyMjIyMjIyMjIyMjIyNcbnggPC0gd2l0aCh0ZXN0LCBzZXEobWluKEFnZV90KSwgbWF4KEFnZV90KSwgbGVuZ3RoLm91dD1uKSlcbnkgPC0gd2l0aCh0ZXN0LCBzZXEobWluKFBvcFNpemVfdHJhbnNmb3JtZWQpLCBtYXgoUG9wU2l6ZV90cmFuc2Zvcm1lZCksIGxlbmd0aC5vdXQ9bikpXG56IDwtIG1hdHJpeChucm93PW4sIG5jb2w9bikgI2NvbnRvdXIgaW50ZXJwcmV0cyB0aGUgeiBtYXRyaXggYXMgYSB0YWJsZSBvZiBmKHhbaV0sIHlbal0pIHZhbHVlcywgc28gdGhhdCB0aGUgeCBheGlzIGNvcnJlc3BvbmRzIHRvIHJvdyBudW1iZXIgYW5kIHRoZSB5IGF4aXMgdG8gY29sdW1uIG51bWJlciwgd2l0aCBjb2x1bW4gMSBhdCB0aGUgYm90dG9tLCBpLmUuIGEgOTAgZGVncmVlIGNvdW50ZXItY2xvY2t3aXNlIHJvdGF0aW9uIG9mIHRoZSBjb252ZW50aW9uYWwgdGV4dHVhbCBsYXlvdXQuXG5uZXdkYXQxIDwtIGRhdGEuZnJhbWUoSUZNX3RyYW5zZm9ybWVkPXJlcChtZWRpYW4odGVzdCRJRk1fdHJhbnNmb3JtZWQpLG4pLFxuICAgICAgICAgICAgICAgICAgICAgIFBvcFNpemVfdHJhbnNmb3JtZWQ9eSlcbmZvcihpIGluIDE6bikge1xuICBuZXdkYXQyIDwtIG5ld2RhdDFcbiAgbmV3ZGF0MiRBZ2VfdCA8LSByZXAoeFtpXSxuKVxuICB6W2ksXSA8LSBwcmVkaWN0KGxtbS5IZS5wb2wsIG5ld2RhdGE9bmV3ZGF0MiwgbGV2ZWw9MCkgI2VhY2ggcm93IGFzIGEgY29uc3RhbnQgeCB2YWx1ZSwgYnV0IGEgY2hhbmdpbmcgeSB2YWx1ZVxufVxuXG5jbG91ZCA8LSB0ZXN0WyxjKFxcQWdlX3RcXCxcXFBvcFNpemVfdHJhbnNmb3JtZWRcXCldICN4IGFuZCB5IGNvb3JpZG5hdGVzIG9mIGEgcG9pbnQgY2xvdWRcbmh1bGwgPC0gYXMubWF0cml4KGNsb3VkW2NodWxsKGNsb3VkKSxdKSAjY29vcmRpbmF0ZXMgb2YgdGhlIGNvbnZleCBodWxsIHZlcnRpY2VzIChjaHVsbCByZXR1cm5zIHRoZSBpbmRpY2VzIG9mIHRoZSB2ZXJ0ZXggcG9pbnRzKVxuY2hlY2sgPC0gYXMubWF0cml4KGRhdGEuZnJhbWUoeD1yZXAoeCwgZWFjaD1uKSwgeT1yZXAoeSwgdGltZXM9bikpKSAgI1AtYnktMiBtYXRyaXggd2l0aCBwb2ludHMgdG8gYmUgY2hlY2tlZFxucG9pbnQueDwtY2hlY2tbLDFdXG5wb2ludC55PC1jaGVja1ssMl1cbnhfYXJyYXk8LWh1bGxbLDFdXG55X2FycmF5PC1odWxsWywyXVxud2l0aGluPC1zcDo6cG9pbnQuaW4ucG9seWdvbihwb2ludC54LHBvaW50LnkseF9hcnJheSx5X2FycmF5KSMjIDA6IHBvaW50IGlzIG91dCBvZiB0aGUgcG9seWdvbiwgMTogcG9pbnQgaXMgaW4gdGhlIHBvbHlnb24sIDI6IHBvaW50IGlzIG9uIHRoZSBsaW5lXG51bmlxdWUod2l0aGluKVxuIy0tPiBubyBlcnJvciBjb2Rlc1xud2l0aGluIDwtIGlmZWxzZSh3aXRoaW4+PTEsIFRSVUUsIEZBTFNFKVxucGxvdChjaGVja1ssMV0sIGNoZWNrWywyXSwgcGNoPTE2LCBjb2w9aWZlbHNlKHdpdGhpbixcXHJlZFxcLCBcXHdoaXRlXFwpKVxud2l0aGluIDwtIG1hdHJpeCh3aXRoaW4sIG5yb3c9biwgbmNvbD1uLCBieXJvdz1UUlVFKVxuelshd2l0aGluXSA8LSBOQSAjc2V0IGFsbCB6LXZhbHVlcyBvdXRzaWRlIHRoZSBjb252ZXggaHVsbCB0byBOQVxuXG50eiA8LSB0KHopICNiZWNhdXNlIHBsb3RseSBpbnRlcnByZXRzIHogYXMgdCh6KTsgYWx0ZXJuYXRpdmVseSwgdGhlIGFyZ3VtZW50IHRyYW5zcG9zZSBjYW4gYmUgc2V0IHRvIFRSVUUgaW4gcGxvdF9seSgpXG5maWc0YyA8LSBwbG90X2x5KHg9eCwgeT15LCB6PXR6LCB0eXBlPVxcY29udG91clxcLFxuICAgICAgICAgICAgICAgICBjb2xvcnM9Y3IsXG4gICAgICAgICAgICAgICAgIGF1dG9jb250b3VyPUYsIGNvbnRvdXJzPWxpc3Qoc3RhcnQ9bWluKHosbmEucm09VCksZW5kPW1heCh6LG5hLnJtPVQpLHNpemU9MC4xKSxcbiAgICAgICAgICAgICAgICAgd2lkdGg9MTMzMCwgaGVpZ2h0PTEzMzAtMTMzMC82KSAgICNzZXQgcGxvdCB3aWR0aCBhbmQgaGVpZ2h0IGluIHB4XG5maWc0YyA8LSBmaWc0YyAlPiUgY29sb3JiYXIobGVuPTEsIHRpdGxlPVxcPGk+SDxcL2k+PHN1Yj5lXFwsIHRpdGxlZm9udD1mLmxhYiwgdGlja2ZvbnQ9Zi5heCwgdGljazA9LTEuNSwgZHRpY2s9MC41KSMgZXhwcmVzc2lvbihEW2VzdF0pIGRvZXMgbm90IHdvcmsuLi5cbmZpZzRjIDwtIGZpZzRjICU+JSBhZGRfbWFya2Vycyh4PWNsb3VkJEFnZV90LCB5PWNsb3VkJFBvcFNpemVfdHJhbnNmb3JtZWQsIGluaGVyaXQ9RkFMU0UsIHNob3dsZWdlbmQ9RkFMU0UsXG4gICAgICAgICAgICAgICAgICAgICAgICAgICAgICAgbWFya2VyPWxpc3Qoc3ltYm9sPVxcY2lyY2xlLW9wZW5cXCwgY29sb3I9XFxibGFja1xcLCBzaXplPTE2KSlcbmZpZzRjIDwtIGZpZzRjICU+JSBsYXlvdXQoeGF4aXM9bGlzdCh0aXRsZT1cXFBPUF9BR0VcXCwgdGl0bGVmb250PWYubGFiLCB0aWNrZm9udD1mLmF4LCBzaG93Z3JpZD1GQUxTRSwgemVyb2xpbmU9RkFMU0UsXG4gICAgICAgICAgICAgICAgICAgICAgICAgICAgICAgICAgICAgc2hvd2xpbmU9VFJVRSwgdGlja2xlbj0xNSwgdGlja3dpZHRoPTMsIGxpbmV3aWR0aD0zKSwgICAgICAgICAgICNhZGQgYXhpcyBsYWJlbHNcbiAgICAgICAgICAgICAgICAgICAgICAgICAgeWF4aXM9bGlzdCh0aXRsZT1cXFBPUF9TSVpFXFwsIHRpdGxlZm9udD1mLmxhYiwgdGlja2ZvbnQ9Zi5heCwgc2hvd2dyaWQ9RkFMU0UsIHplcm9saW5lPUZBTFNFLFxuICAgICAgICAgICAgICAgICAgICAgICAgICAgICAgICAgICAgIHNob3dsaW5lPVRSVUUsIHRpY2tsZW49MTUsIHRpY2t3aWR0aD0zLCBsaW5ld2lkdGg9MyksXG4gICAgICAgICAgICAgICAgICAgICAgICAgIG1hcmdpbj1saXN0KGw9NjAsIHI9ODAsIHQ9MjAsIGI9NjApKSAgICAgICAgICAgICAgICAgICAgICAgICAgICAjc2V0cyB0aGUgcGxvdCBtYXJnaW4gaW4gcHgsIHNlZSBodHRwczovL3Bsb3RseS5jb20vci9yZWZlcmVuY2UvbGF5b3V0LyNsYXlvdXQtYXV0b3NpemVcbmZpZzRjXG5gYGBcbmBgYFxuYGBgXG5gYGAifQ== -->

```r
```r
```r
```r
##Figure_4
library(ptinpoly)
library(plotly)
n <- 100 #number of grid points to be used along each axis
f.ax <- list(family=\Arial\, size=48) #font for tick labels --> unit unclear --> must be larger when figure size in px is larger
f.lab <- list(family=\Arial\, size=56) #font for axes labels
f.ann <- list(family=\Arial\, size=48, color=\steelblue\)

cr <- colorRamp(colors=c(\#364B9A\, \#4A7BB7\, \#6EA6CD\, \#98CAE1\, \#C2E4EF\, \#EAECCC\, \#FEDA8B\, \#FDB366\,
                         \#F67E4B\, \#DD3D2D\, \#A50026\)) #--> color scale \sunset\ designed by Paul Tol for colorblind people: https://personal.sron.nl/~pault/

#############Oxa#################

####### He#############
test<-as.data.frame(model.matrix(lmm.He.oxa))

x <- with(test, seq(min(Age_t), max(Age_t), length.out=n))
y <- with(test, seq(min(IFM_transformed), max(IFM_transformed), length.out=n))
z <- matrix(nrow=n, ncol=n) #contour interprets the z matrix as a table of f(x[i], y[j]) values, so that the x axis corresponds to row number and the y axis to column number, with column 1 at the bottom, i.e. a 90 degree counter-clockwise rotation of the conventional textual layout.
newdat1 <- data.frame(PopSize_transformed=rep(median(test$PopSize_transformed),n),
                      IFM_transformed=y)
for(i in 1:n) {
  newdat2 <- newdat1
  newdat2$Age_t <- rep(x[i],n)
  z[i,] <- predict(lmm.He.oxa, newdata=newdat2, level=0) #each row as a constant x value, but a changing y value
}

cloud <- test[,c(\Age_t\,\IFM_transformed\)] #x and y cooridnates of a point cloud
hull <- as.matrix(cloud[chull(cloud),]) #coordinates of the convex hull vertices (chull returns the indices of the vertex points)
check <- as.matrix(data.frame(x=rep(x, each=n), y=rep(y, times=n)))  #P-by-2 matrix with points to be checked

point.x<-check[,1]
point.y<-check[,2]
x_array<-hull[,1]
y_array<-hull[,2]
within<-sp::point.in.polygon(point.x,point.y,x_array,y_array)## 0: point is out of the polygon, 1: point is in the polygon, 2: point is on the line
unique(within)
#--> no error codes
within <- ifelse(within>=1, TRUE, FALSE)
plot(check[,1], check[,2], pch=16, col=ifelse(within,\red\, \white\))
within <- matrix(within, nrow=n, ncol=n, byrow=TRUE)
z[!within] <- NA #set all z-values outside the convex hull to NA

tz <- t(z) #because plotly interprets z as t(z); alternatively, the argument transpose can be set to TRUE in plot_ly()
fig4a <- plot_ly(x=x, y=y, z=tz, type=\contour\,
                 colors=cr,
                 autocontour=F, contours=list(start=min(z,na.rm=T),end=max(z,na.rm=T),size=0.1),
                 width=1330, height=1330-1330/6)   #set plot width and height in px
fig4a <- fig4a %>% colorbar(len=1, title=\<i>H</i><sub>e\, titlefont=f.lab, tickfont=f.ax, tick0=-1.5, dtick=0.5)# expression(D[est]) does not work...
fig4a <- fig4a %>% add_markers(x=cloud$Age_t, y=cloud$IFM_transformed, inherit=FALSE, showlegend=FALSE,
                               marker=list(symbol=\circle-open\, color=\black\, size=16))
fig4a <- fig4a %>% layout(xaxis=list(title=\POP_AGE\, titlefont=f.lab, tickfont=f.ax, showgrid=FALSE, zeroline=FALSE,
                                     showline=TRUE, ticklen=15, tickwidth=3, linewidth=3),           #add axis labels
                          yaxis=list(title=\SPA_CON\, titlefont=f.lab, tickfont=f.ax, showgrid=FALSE, zeroline=FALSE,
                                     showline=TRUE, ticklen=15, tickwidth=3, linewidth=3),
                          margin=list(l=60, r=80, t=20, b=60))                           
fig4a

##### Ho####
test<-as.data.frame(model.matrix(lmm.Ho.oxa))
x <- with(test, seq(min(Age_t), max(Age_t), length.out=n))
y <- with(test, seq(min(PopSize_transformed), max(PopSize_transformed), length.out=n))
z <- matrix(nrow=n, ncol=n) #contour interprets the z matrix as a table of f(x[i], y[j]) values, so that the x axis corresponds to row number and the y axis to column number, with column 1 at the bottom, i.e. a 90 degree counter-clockwise rotation of the conventional textual layout.
newdat1 <- data.frame(IFM_transformed=rep(median(test$IFM_transformed),n),
                      PopSize_transformed=y)
for(i in 1:n) {
  newdat2 <- newdat1
  newdat2$Age_t <- rep(x[i],n)
  z[i,] <- predict(lmm.Ho.oxa, newdata=newdat2, level=0) #each row as a constant x value, but a changing y value
}

cloud <- test[,c(\Age_t\,\PopSize_transformed\)] #x and y cooridnates of a point cloud
hull <- as.matrix(cloud[chull(cloud),]) #coordinates of the convex hull vertices (chull returns the indices of the vertex points)
check <- as.matrix(data.frame(x=rep(x, each=n), y=rep(y, times=n)))  #P-by-2 matrix with points to be checked

point.x<-check[,1]
point.y<-check[,2]
x_array<-hull[,1]
y_array<-hull[,2]
within<-sp::point.in.polygon(point.x,point.y,x_array,y_array)## 0: point is out of the polygon, 1: point is in the polygon, 2: point is on the line
unique(within)
#--> no error codes
within <- ifelse(within>=1, TRUE, FALSE)
plot(check[,1], check[,2], pch=16, col=ifelse(within,\red\, \white\))
within <- matrix(within, nrow=n, ncol=n, byrow=TRUE)
z[!within] <- NA #set all z-values outside the convex hull to NA

tz <- t(z) #because plotly interprets z as t(z); alternatively, the argument transpose can be set to TRUE in plot_ly()
fig4b <- plot_ly(x=x, y=y, z=tz, type=\contour\,
                 colors=cr,
                 autocontour=F, contours=list(start=min(z,na.rm=T),end=max(z,na.rm=T),size=0.1),
                 width=1330, height=1330-1330/6)   #set plot width and height in px
fig4b <- fig4b %>% colorbar(len=1, title=\<i>H</i><sub>o\, titlefont=f.lab, tickfont=f.ax, tick0=-1.5, dtick=0.5)# expression(D[est]) does not work...
fig4b <- fig4b %>% add_markers(x=cloud$Age_t, y=cloud$PopSize_transformed, inherit=FALSE, showlegend=FALSE,
                               marker=list(symbol=\circle-open\, color=\black\, size=16))
fig4b <- fig4b %>% layout(xaxis=list(title=\POP_AGE\, titlefont=f.lab, tickfont=f.ax, showgrid=FALSE, zeroline=FALSE,
                                     showline=TRUE, ticklen=15, tickwidth=3, linewidth=3),           #add axis labels
                          yaxis=list(title=\POP_SIZE\, titlefont=f.lab, tickfont=f.ax, showgrid=FALSE, zeroline=FALSE,
                                     showline=TRUE, ticklen=15, tickwidth=3, linewidth=3),
                          margin=list(l=60, r=80, t=20, b=60))                            #sets the plot margin in px, see https://plotly.com/r/reference/layout/#layout-autosize
fig4b
#############Pol#################
test<-as.data.frame(model.matrix(lmm.He.pol))

################popsize& He#########################
x <- with(test, seq(min(Age_t), max(Age_t), length.out=n))
y <- with(test, seq(min(PopSize_transformed), max(PopSize_transformed), length.out=n))
z <- matrix(nrow=n, ncol=n) #contour interprets the z matrix as a table of f(x[i], y[j]) values, so that the x axis corresponds to row number and the y axis to column number, with column 1 at the bottom, i.e. a 90 degree counter-clockwise rotation of the conventional textual layout.
newdat1 <- data.frame(IFM_transformed=rep(median(test$IFM_transformed),n),
                      PopSize_transformed=y)
for(i in 1:n) {
  newdat2 <- newdat1
  newdat2$Age_t <- rep(x[i],n)
  z[i,] <- predict(lmm.He.pol, newdata=newdat2, level=0) #each row as a constant x value, but a changing y value
}

cloud <- test[,c(\Age_t\,\PopSize_transformed\)] #x and y cooridnates of a point cloud
hull <- as.matrix(cloud[chull(cloud),]) #coordinates of the convex hull vertices (chull returns the indices of the vertex points)
check <- as.matrix(data.frame(x=rep(x, each=n), y=rep(y, times=n)))  #P-by-2 matrix with points to be checked
point.x<-check[,1]
point.y<-check[,2]
x_array<-hull[,1]
y_array<-hull[,2]
within<-sp::point.in.polygon(point.x,point.y,x_array,y_array)## 0: point is out of the polygon, 1: point is in the polygon, 2: point is on the line
unique(within)
#--> no error codes
within <- ifelse(within>=1, TRUE, FALSE)
plot(check[,1], check[,2], pch=16, col=ifelse(within,\red\, \white\))
within <- matrix(within, nrow=n, ncol=n, byrow=TRUE)
z[!within] <- NA #set all z-values outside the convex hull to NA

tz <- t(z) #because plotly interprets z as t(z); alternatively, the argument transpose can be set to TRUE in plot_ly()
fig4c <- plot_ly(x=x, y=y, z=tz, type=\contour\,
                 colors=cr,
                 autocontour=F, contours=list(start=min(z,na.rm=T),end=max(z,na.rm=T),size=0.1),
                 width=1330, height=1330-1330/6)   #set plot width and height in px
fig4c <- fig4c %>% colorbar(len=1, title=\<i>H</i><sub>e\, titlefont=f.lab, tickfont=f.ax, tick0=-1.5, dtick=0.5)# expression(D[est]) does not work...
fig4c <- fig4c %>% add_markers(x=cloud$Age_t, y=cloud$PopSize_transformed, inherit=FALSE, showlegend=FALSE,
                               marker=list(symbol=\circle-open\, color=\black\, size=16))
fig4c <- fig4c %>% layout(xaxis=list(title=\POP_AGE\, titlefont=f.lab, tickfont=f.ax, showgrid=FALSE, zeroline=FALSE,
                                     showline=TRUE, ticklen=15, tickwidth=3, linewidth=3),           #add axis labels
                          yaxis=list(title=\POP_SIZE\, titlefont=f.lab, tickfont=f.ax, showgrid=FALSE, zeroline=FALSE,
                                     showline=TRUE, ticklen=15, tickwidth=3, linewidth=3),
                          margin=list(l=60, r=80, t=20, b=60))                            #sets the plot margin in px, see https://plotly.com/r/reference/layout/#layout-autosize
fig4c
```

```
<!-- rnb-source-end -->

<!-- rnb-chunk-end -->


<!-- rnb-text-begin -->

Genetic Differentiation

<!-- rnb-text-end -->


<!-- rnb-chunk-begin -->


<!-- rnb-source-begin eyJkYXRhIjoiYGBgclxuYGBgclxuXG4jIyMgRmlndXJlXzVcbiMjIyMjIyMjIyMjIyMjIyMjIyMjI0FHRV9CQVNFIyNcbnBhcihtZnJvdz1jKDEsMyksbWFyPWMoNSw1LDIsMikpXG4jIyBHc3RcbmVmZi5Hc3QuQWdlX3lvdW5nZXI8LWVmZmVjdChcXEFnZV95b3VuZ2VyX3RcXCxHc3QuZmluLCBwYXJ0aWFsLnJlc2lkdWFscz1UKVxucGxvdC5kYXRhLkFnZS5hbGw8LWVmZi5Hc3QuQWdlX3lvdW5nZXIkZGF0YVxucGxvdC5kYXRhLkFnZS5maXQ8LXdpdGgoZWZmLkdzdC5BZ2VfeW91bmdlcixjYmluZCh4LGZpdCxsb3dlcix1cHBlcikpXG5cbnBsb3QoMyx0eXBlPVxcblxcLHhsaW09YygtMiwzKSx5bGltPWMoLTMsMi41KSx4YXh0PSduJyxhbm49Rix5YXh0PSduJylcbmF4aXMoc2lkZT0yLGx3ZD0yLGNleC5heGlzPTEuNSlcbmF4aXMoc2lkZT0xLGx3ZD0yLGNleC5heGlzPTEuNSxhdD1jKC0wLjY5Mjk2OTIsMC43OTE2ODQ5LDEuNjAxMjc3LDIuMTgxNTUxKSxsYWJlbHMgPSBjKDUwLDE1MCwyNTAsMzUwKSlcbmJveChsd2Q9MilcbnRpdGxlKHhsYWI9XFxBR0VfQkFTRSAoeWVhcilcXCx5bGFiPWV4cHJlc3Npb24oaXRhbGljKHBhc3RlKFxcRycnXFwpKVtTVF0pLGxpbmU9MyxjZXgubGFiPTEuNSlcbndpdGgocGxvdC5kYXRhLkFnZS5maXQscG9seWdvbih4PWMoQWdlX3lvdW5nZXJfdCwgcmV2KEFnZV95b3VuZ2VyX3QpKSx5PWMobG93ZXIsIHJldih1cHBlcikpLFxuICAgICAgICAgICAgICAgICAgICAgICAgICAgICAgIGNvbD1jKFxcI2RjZGNkY1xcKSwgYm9yZGVyPU5BKSkgXG5cbndpdGgoZGlzdFtkaXN0JFNwZWNpZXMueD09XFxBbmVcXCxdLCBwb2ludHMoeD1qaXR0ZXIoQWdlX3lvdW5nZXJfdCkseT1Hc3RfdCxwY2g9MjEsY2V4PTIsbHdkPTEuNSxcbiAgICAgICAgICAgICAgICAgICAgICAgICAgICAgICAgICAgICAgICAgIGJnPWMoXFwjYmI1NTY2XFwpKSlcblxud2l0aChkaXN0W2Rpc3QkU3BlY2llcy54PT1cXE94YVxcLF0sIHBvaW50cyh4PWppdHRlcihBZ2VfeW91bmdlcl90KSx5PUdzdF90LHBjaD0yMSxjZXg9Mixsd2Q9MS41LCBcbiAgICAgICAgICAgICAgICAgICAgICAgICAgICAgICAgICAgICAgICAgIGJnPWMoXFwjZGRhYTMzXFwpKSlcbndpdGgoZGlzdFtkaXN0JFNwZWNpZXMueD09XFxQb2xcXCxdLCBwb2ludHMoeD1qaXR0ZXIoQWdlX3lvdW5nZXJfdCkseT1Hc3RfdCxwY2g9MjEsY2V4PTIsbHdkPTEuNSwgXG4gICAgICAgICAgICAgICAgICAgICAgICAgICAgICAgICAgICAgICAgICBiZz1jKFxcIzAwNDQ4OFxcKSkpXG5cbndpdGgocGxvdC5kYXRhLkFnZS5maXQsbGluZXMoQWdlX3lvdW5nZXJfdCxmaXQsY29sPXJnYigwLDAsMCxtYXhDb2xvclZhbHVlID0gMjU1KSxsd2Q9MixsdHk9NSkpXG5tdGV4dChhZGo9LTAuMixcXChBKVxcLGNleD0xLjUpXG4jI0Rwc1xuIyMjIyMjIyMjIyMjIyMjIyNcbmVmZi5EcHMuQWdlX3lvdW5nZXI8LWVmZmVjdChcXEFnZV95b3VuZ2VyX3RcXCxEcHMuZmluLCBwYXJ0aWFsLnJlc2lkdWFscz1UKVxucGxvdC5kYXRhLkFnZS5hbGw8LWVmZi5EcHMuQWdlX3lvdW5nZXIkZGF0YVxucGxvdC5kYXRhLkFnZS5maXQ8LXdpdGgoZWZmLkRwcy5BZ2VfeW91bmdlcixjYmluZCh4LGZpdCxsb3dlcix1cHBlcikpXG5cbnBsb3QoMyx0eXBlPVxcblxcLHhsaW09YygtMiwzKSx5bGltPWMoLTMsMi41KSx4YXh0PSduJyxhbm49Rix5YXh0PSduJylcbmF4aXMoc2lkZT0yLGx3ZD0yLGNleC5heGlzPTEuNSlcbmF4aXMoc2lkZT0xLGx3ZD0yLGNleC5heGlzPTEuNSxhdD1jKC0wLjY5Mjk2OTIsMC43OTE2ODQ5LDEuNjAxMjc3LDIuMTgxNTUxKSxsYWJlbHMgPSBjKDUwLDE1MCwyNTAsMzUwKSlcbmJveChsd2Q9MilcbnRpdGxlKHhsYWI9XFxBR0VfQkFTRSAoeWVhcilcXCx5bGFiPWV4cHJlc3Npb24oaXRhbGljKHBhc3RlKFxcRFxcKSlbUFNdKSxsaW5lPTMsY2V4LmxhYj0xLjUpXG53aXRoKHBsb3QuZGF0YS5BZ2UuZml0LHBvbHlnb24oeD1jKEFnZV95b3VuZ2VyX3QsIHJldihBZ2VfeW91bmdlcl90KSkseT1jKGxvd2VyLCByZXYodXBwZXIpKSxcbiAgICAgICAgICAgICAgICAgICAgICAgICAgICAgICBjb2w9YyhcXCNkY2RjZGNcXCksIGJvcmRlcj1OQSkpIFxuXG53aXRoKGRpc3RbZGlzdCRTcGVjaWVzLng9PVxcQW5lXFwsXSwgcG9pbnRzKHg9aml0dGVyKEFnZV95b3VuZ2VyX3QpLHk9RHBzX3QscGNoPTIxLGNleD0yLGx3ZD0xLjUsXG4gICAgICAgICAgICAgICAgICAgICAgICAgICAgICAgICAgICAgICAgICBiZz1jKFxcI2JiNTU2NlxcKSkpXG5cbndpdGgoZGlzdFtkaXN0JFNwZWNpZXMueD09XFxPeGFcXCxdLCBwb2ludHMoeD1qaXR0ZXIoQWdlX3lvdW5nZXJfdCkseT1EcHNfdCxwY2g9MjEsY2V4PTIsbHdkPTEuNSwgXG4gICAgICAgICAgICAgICAgICAgICAgICAgICAgICAgICAgICAgICAgICBiZz1jKFxcI2RkYWEzM1xcKSkpXG53aXRoKGRpc3RbZGlzdCRTcGVjaWVzLng9PVxcUG9sXFwsXSwgcG9pbnRzKHg9aml0dGVyKEFnZV95b3VuZ2VyX3QpLHk9RHBzX3QscGNoPTIxLGNleD0yLGx3ZD0xLjUsIFxuICAgICAgICAgICAgICAgICAgICAgICAgICAgICAgICAgICAgICAgICAgYmc9YyhcXCMwMDQ0ODhcXCkpKVxuXG53aXRoKHBsb3QuZGF0YS5BZ2UuZml0LGxpbmVzKEFnZV95b3VuZ2VyX3QsZml0LGNvbD1yZ2IoMCwwLDAsbWF4Q29sb3JWYWx1ZSA9IDI1NSksbHdkPTIpKVxubXRleHQoYWRqPS0wLjIsXFwoQilcXCxjZXg9MS41KVxuIyMjIGNHRCMjIyMjIyMjIyNcbmVmZmVjdC5hZ2ViYXNlPC1lZmZlY3QoXFxBZ2VfeW91bmdlcl90OlNwZWNpZXMueFxcLGMuR0QueS5maW4scGFydGlhbC5yZXNpZHVhbHM9VClcbnBsb3QuZGF0YS5hbGw8LWVmZmVjdC5hZ2ViYXNlJGRhdGFcbnBsb3QuZGF0YS5maXQ8LXdpdGgoZWZmZWN0LmFnZWJhc2UsY2JpbmQoeCxmaXQsbG93ZXIsdXBwZXIpKVxucGxvdCgzLHR5cGU9XFxuXFwseGxpbT1jKC0yLDMpLHlsaW09YygtMywzKSx4YXh0PSduJyxhbm49Rix5YXh0PSduJylcbmF4aXMoc2lkZT0yLGx3ZD0yLGNleC5heGlzPTEuNSlcbmF4aXMoc2lkZT0xLGx3ZD0yLGNleC5heGlzPTEuNSxhdD1jKC0wLjcwNzU0NzMsMC44MDk0MTg4LDEuNjA0NTU1LDIuMTYyNTg0KSxsYWJlbHMgPSBjKDUwLDE1MCwyNTAsMzUwKSlcbmJveChsd2Q9MilcbnRpdGxlKHhsYWI9YyhcXEFHRV9CQVNFICh5ZWFyKVxcKSx5bGFiPWV4cHJlc3Npb24oaXRhbGljKGMpKlxcR0RcXCksbGluZT0zLGNleC5sYWI9MS41KVxuXG53aXRoKHBsb3QuZGF0YS5maXRbcGxvdC5kYXRhLmZpdCRTcGVjaWVzLng9PVxcQW5lXFwsXSxwb2x5Z29uKHg9YyhBZ2VfeW91bmdlcl90LCByZXYoQWdlX3lvdW5nZXJfdCkpLHk9Yyhsb3dlciwgcmV2KHVwcGVyKSksXG4gICAgICAgICAgICAgICAgICAgICAgICAgICAgICAgICAgICAgICAgICAgICAgICAgICAgICAgICAgICBjb2w9c2NhbGVzOjphbHBoYShcXCNiYjU1NjZcXCwwLjMpLCBib3JkZXI9TkEpKVxuXG53aXRoKHBsb3QuZGF0YS5maXRbcGxvdC5kYXRhLmZpdCRTcGVjaWVzLng9PVxcUG9sXFwsXSxwb2x5Z29uKHg9YyhBZ2VfeW91bmdlcl90LCByZXYoQWdlX3lvdW5nZXJfdCkpLHk9Yyhsb3dlciwgcmV2KHVwcGVyKSksXG4gICAgICAgICAgICAgICAgICAgICAgICAgICAgICAgICAgICAgICAgICAgICAgICAgICAgICAgICAgICBjb2w9c2NhbGVzOjphbHBoYShcXCMwMDQ0ODhcXCwwLjMpLCBib3JkZXI9TkEpKVxuXG5cbndpdGgocGxvdC5kYXRhLmFsbFtwbG90LmRhdGEuYWxsJFNwZWNpZXMueD09XFxBbmVcXCxdLCBwb2ludHMoeD1qaXR0ZXIoQWdlX3lvdW5nZXJfdCkseT1Db24uRF90LGNleD0yLHBjaD0yMSxsd2Q9MS41LFxuICAgICAgICAgICAgICAgICAgICAgICAgICAgICAgICAgICAgICAgICAgICAgICAgICAgICAgICAgICAgYmc9YyhcXCNiYjU1NjZcXCkpKVxuXG53aXRoKHBsb3QuZGF0YS5hbGxbcGxvdC5kYXRhLmFsbCRTcGVjaWVzLng9PVxcT3hhXFwsXSwgcG9pbnRzKHg9aml0dGVyKEFnZV95b3VuZ2VyX3QpLHk9Q29uLkRfdCxjZXg9MixwY2g9MjEsbHdkPTEuNSwgXG4gICAgICAgICAgICAgICAgICAgICAgICAgICAgICAgICAgICAgICAgICAgICAgICAgICAgICAgICAgICBiZz1jKFxcI2RkYWEzM1xcKSkpXG53aXRoKHBsb3QuZGF0YS5hbGxbcGxvdC5kYXRhLmFsbCRTcGVjaWVzLng9PVxcUG9sXFwsXSwgcG9pbnRzKHg9aml0dGVyKEFnZV95b3VuZ2VyX3QpLHk9Q29uLkRfdCxjZXg9MixwY2g9MjEsbHdkPTEuNSwgXG4gICAgICAgICAgICAgICAgICAgICAgICAgICAgICAgICAgICAgICAgICAgICAgICAgICAgICAgICAgICBiZz1jKFxcIzAwNDQ4OFxcKSkpXG5cbndpdGgocGxvdC5kYXRhLmZpdFtwbG90LmRhdGEuZml0JFNwZWNpZXMueD09XFxBbmVcXCxdLGxpbmVzKEFnZV95b3VuZ2VyX3QsZml0LGNvbD1jKFxcI2JiNTU2NlxcKSxsd2Q9MikpXG53aXRoKHBsb3QuZGF0YS5maXRbcGxvdC5kYXRhLmZpdCRTcGVjaWVzLng9PVxcT3hhXFwsXSxsaW5lcyhBZ2VfeW91bmdlcl90LGZpdCxjb2w9YyhcXCNkZGFhMzNcXCksbHdkPTIsbHR5PTUpKVxud2l0aChwbG90LmRhdGEuZml0W3Bsb3QuZGF0YS5maXQkU3BlY2llcy54PT1cXFBvbFxcLF0sbGluZXMoQWdlX3lvdW5nZXJfdCxmaXQsY29sPWMoXFwjMDA0NDg4XFwpLGx3ZD0yKSlcblxud2l0aChwbG90LmRhdGEuZml0LCB0ZXh0KDIuNSwgeT1jKG1pbihmaXRbU3BlY2llcy54PT1cXEFuZVxcXSksXG4gICAgICAgICAgICAgICAgICAgICAgICAgICAgICAgICAgbWluKGZpdFtTcGVjaWVzLng9PVxcT3hhXFxdKSxcbiAgICAgICAgICAgICAgICAgICAgICAgICAgICAgICAgICBtYXgoZml0W1NwZWNpZXMueD09XFxQb2xcXF0pKzAuNSksXG4gICAgICAgICAgICAgICAgICAgICAgICAgbGFiZWxzPWMoXFxhXFwsIFxcYWJcXCxcXGJcXCksIGNleD0xLjUsIGFkaj0xKSlcbm10ZXh0KGFkaj0tMC4yLFxcKEMpXFwsY2V4PTEuNSlcbmdyYXBoaWNzOjpsZWdlbmQoMS40LDMuMixsZWdlbmQ9ZXhwcmVzc2lvbihpdGFsaWMoQS5uZW0uKSxpdGFsaWMoTy5hY2UuKSxpdGFsaWMoUC5tdWwuKSxcXHAgPiAwLjA1XFwsXFxwXFw8PVxcMC4wNVxcKSxidHk9J24nLFxuICAgICAgICAgICAgICAgICBwY2g9YygyMSwyMSwyMSxOQSxOQSkscHQuYmc9YyhcXCNiYjU1NjZcXCxcXCNkZGFhMzNcXCxcXCMwMDQ0ODhcXCxOQSxOQSksbHR5PWMoTkEsTkEsTkEsNSwxKSxcbiAgICAgICAgICAgICAgICAgc2VnLmxlbj0wLjUsY2V4PTEuMyx4LmludGVyc3A9MC4xLHkuaW50ZXJzcD0wLjYpXG5cblxuYGBgXG5gYGAifQ== -->

```r
```r

### Figure_5
#####################AGE_BASE##
par(mfrow=c(1,3),mar=c(5,5,2,2))
## Gst
eff.Gst.Age_younger<-effect(\Age_younger_t\,Gst.fin, partial.residuals=T)
plot.data.Age.all<-eff.Gst.Age_younger$data
plot.data.Age.fit<-with(eff.Gst.Age_younger,cbind(x,fit,lower,upper))

plot(3,type=\n\,xlim=c(-2,3),ylim=c(-3,2.5),xaxt='n',ann=F,yaxt='n')
axis(side=2,lwd=2,cex.axis=1.5)
axis(side=1,lwd=2,cex.axis=1.5,at=c(-0.6929692,0.7916849,1.601277,2.181551),labels = c(50,150,250,350))
box(lwd=2)
title(xlab=\AGE_BASE (year)\,ylab=expression(italic(paste(\G''\))[ST]),line=3,cex.lab=1.5)
with(plot.data.Age.fit,polygon(x=c(Age_younger_t, rev(Age_younger_t)),y=c(lower, rev(upper)),
                               col=c(\#dcdcdc\), border=NA)) 

with(dist[dist$Species.x==\Ane\,], points(x=jitter(Age_younger_t),y=Gst_t,pch=21,cex=2,lwd=1.5,
                                          bg=c(\#bb5566\)))

with(dist[dist$Species.x==\Oxa\,], points(x=jitter(Age_younger_t),y=Gst_t,pch=21,cex=2,lwd=1.5, 
                                          bg=c(\#ddaa33\)))
with(dist[dist$Species.x==\Pol\,], points(x=jitter(Age_younger_t),y=Gst_t,pch=21,cex=2,lwd=1.5, 
                                          bg=c(\#004488\)))

with(plot.data.Age.fit,lines(Age_younger_t,fit,col=rgb(0,0,0,maxColorValue = 255),lwd=2,lty=5))
mtext(adj=-0.2,\(A)\,cex=1.5)
##Dps
#################
eff.Dps.Age_younger<-effect(\Age_younger_t\,Dps.fin, partial.residuals=T)
plot.data.Age.all<-eff.Dps.Age_younger$data
plot.data.Age.fit<-with(eff.Dps.Age_younger,cbind(x,fit,lower,upper))

plot(3,type=\n\,xlim=c(-2,3),ylim=c(-3,2.5),xaxt='n',ann=F,yaxt='n')
axis(side=2,lwd=2,cex.axis=1.5)
axis(side=1,lwd=2,cex.axis=1.5,at=c(-0.6929692,0.7916849,1.601277,2.181551),labels = c(50,150,250,350))
box(lwd=2)
title(xlab=\AGE_BASE (year)\,ylab=expression(italic(paste(\D\))[PS]),line=3,cex.lab=1.5)
with(plot.data.Age.fit,polygon(x=c(Age_younger_t, rev(Age_younger_t)),y=c(lower, rev(upper)),
                               col=c(\#dcdcdc\), border=NA)) 

with(dist[dist$Species.x==\Ane\,], points(x=jitter(Age_younger_t),y=Dps_t,pch=21,cex=2,lwd=1.5,
                                          bg=c(\#bb5566\)))

with(dist[dist$Species.x==\Oxa\,], points(x=jitter(Age_younger_t),y=Dps_t,pch=21,cex=2,lwd=1.5, 
                                          bg=c(\#ddaa33\)))
with(dist[dist$Species.x==\Pol\,], points(x=jitter(Age_younger_t),y=Dps_t,pch=21,cex=2,lwd=1.5, 
                                          bg=c(\#004488\)))

with(plot.data.Age.fit,lines(Age_younger_t,fit,col=rgb(0,0,0,maxColorValue = 255),lwd=2))
mtext(adj=-0.2,\(B)\,cex=1.5)
### cGD##########
effect.agebase<-effect(\Age_younger_t:Species.x\,c.GD.y.fin,partial.residuals=T)
plot.data.all<-effect.agebase$data
plot.data.fit<-with(effect.agebase,cbind(x,fit,lower,upper))
plot(3,type=\n\,xlim=c(-2,3),ylim=c(-3,3),xaxt='n',ann=F,yaxt='n')
axis(side=2,lwd=2,cex.axis=1.5)
axis(side=1,lwd=2,cex.axis=1.5,at=c(-0.7075473,0.8094188,1.604555,2.162584),labels = c(50,150,250,350))
box(lwd=2)
title(xlab=c(\AGE_BASE (year)\),ylab=expression(italic(c)*\GD\),line=3,cex.lab=1.5)

with(plot.data.fit[plot.data.fit$Species.x==\Ane\,],polygon(x=c(Age_younger_t, rev(Age_younger_t)),y=c(lower, rev(upper)),
                                                            col=scales::alpha(\#bb5566\,0.3), border=NA))

with(plot.data.fit[plot.data.fit$Species.x==\Pol\,],polygon(x=c(Age_younger_t, rev(Age_younger_t)),y=c(lower, rev(upper)),
                                                            col=scales::alpha(\#004488\,0.3), border=NA))


with(plot.data.all[plot.data.all$Species.x==\Ane\,], points(x=jitter(Age_younger_t),y=Con.D_t,cex=2,pch=21,lwd=1.5,
                                                            bg=c(\#bb5566\)))

with(plot.data.all[plot.data.all$Species.x==\Oxa\,], points(x=jitter(Age_younger_t),y=Con.D_t,cex=2,pch=21,lwd=1.5, 
                                                            bg=c(\#ddaa33\)))
with(plot.data.all[plot.data.all$Species.x==\Pol\,], points(x=jitter(Age_younger_t),y=Con.D_t,cex=2,pch=21,lwd=1.5, 
                                                            bg=c(\#004488\)))

with(plot.data.fit[plot.data.fit$Species.x==\Ane\,],lines(Age_younger_t,fit,col=c(\#bb5566\),lwd=2))
with(plot.data.fit[plot.data.fit$Species.x==\Oxa\,],lines(Age_younger_t,fit,col=c(\#ddaa33\),lwd=2,lty=5))
with(plot.data.fit[plot.data.fit$Species.x==\Pol\,],lines(Age_younger_t,fit,col=c(\#004488\),lwd=2))

with(plot.data.fit, text(2.5, y=c(min(fit[Species.x==\Ane\]),
                                  min(fit[Species.x==\Oxa\]),
                                  max(fit[Species.x==\Pol\])+0.5),
                         labels=c(\a\, \ab\,\b\), cex=1.5, adj=1))
mtext(adj=-0.2,\(C)\,cex=1.5)
graphics::legend(1.4,3.2,legend=expression(italic(A.nem.),italic(O.ace.),italic(P.mul.),\p > 0.05\,\p\<=\0.05\),bty='n',
                 pch=c(21,21,21,NA,NA),pt.bg=c(\#bb5566\,\#ddaa33\,\#004488\,NA,NA),lty=c(NA,NA,NA,5,1),
                 seg.len=0.5,cex=1.3,x.intersp=0.1,y.intersp=0.6)
```

```
<!-- rnb-source-end -->

<!-- rnb-chunk-end -->


<!-- rnb-text-begin -->

interaction differentiation

<!-- rnb-text-end -->


<!-- rnb-chunk-begin -->


<!-- rnb-source-begin eyJkYXRhIjoiYGBgclxuYGBgclxuYGBgclxuZWZmLk5vZGVfeW91bmdlcjwtZWZmZWN0KFxcQWdlX3RcXCxub2RlLmZpbiwgcGFydGlhbC5yZXNpZHVhbHM9VClcbnBsb3QuZGF0YS5BZ2UuYWxsPC1lZmYuTm9kZV95b3VuZ2VyJGRhdGFcbnBsb3QuZGF0YS5BZ2UuZml0PC13aXRoKGVmZi5Ob2RlX3lvdW5nZXIsY2JpbmQoeCxmaXQsbG93ZXIsdXBwZXIpKVxuXG5wbG90KDMsdHlwZT1cXG5cXCx4bGltPWMoLTIsMykseWxpbT1jKC0zLDIuNSkseGF4dD0nbicsYW5uPUYseWF4dD0nbicpXG5heGlzKHNpZGU9Mixsd2Q9MixjZXguYXhpcz0xLjMpXG5heGlzKHNpZGU9MSxsd2Q9MixjZXguYXhpcz0xLjMsYXQ9YygtMC42OTI5NjkyLDAuNzkxNjg0OSwxLjYwMTI3NywyLjE4MTU1MSksbGFiZWxzID0gYyg1MCwxNTAsMjUwLDM1MCkpXG5ib3gobHdkPTIpXG50aXRsZSh4bGFiPVxcQUdFX0JBU0UgKHllYXIpXFwseWxhYj1leHByZXNzaW9uKGl0YWxpYyhwYXN0ZShcXERcXCkpW1BTXSksbGluZT0zLGNleC5sYWI9MS41KVxud2l0aChwbG90LmRhdGEuQWdlLmZpdCxwb2x5Z29uKHg9YyhBZ2VfeW91bmdlcl90LCByZXYoQWdlX3lvdW5nZXJfdCkpLHk9Yyhsb3dlciwgcmV2KHVwcGVyKSksXG4gICAgICAgICAgICAgICAgICAgICAgICAgICAgICAgY29sPWMoXFwjZGNkY2RjXFwpLCBib3JkZXI9TkEpKSBcblxud2l0aChkaXN0W2Rpc3QkU3BlY2llcy54PT1cXEFuZVxcLF0sIHBvaW50cyh4PWppdHRlcihBZ2VfeW91bmdlcl90KSx5PURwc190LHBjaD0yMSxjZXg9MS41LGx3ZD0yLFxuICAgICAgICAgICAgICAgICAgICAgICAgICAgICAgICAgICAgICAgICAgYmc9YyhcXCNiYjU1NjZcXCkpKVxuXG53aXRoKGRpc3RbZGlzdCRTcGVjaWVzLng9PVxcT3hhXFwsXSwgcG9pbnRzKHg9aml0dGVyKEFnZV95b3VuZ2VyX3QpLHk9RHBzX3QscGNoPTIxLGNleD0xLjUsbHdkPTIsIFxuICAgICAgICAgICAgICAgICAgICAgICAgICAgICAgICAgICAgICAgICAgYmc9YyhcXCNkZGFhMzNcXCkpKVxud2l0aChkaXN0W2Rpc3QkU3BlY2llcy54PT1cXFBvbFxcLF0sIHBvaW50cyh4PWppdHRlcihBZ2VfeW91bmdlcl90KSx5PURwc190LHBjaD0yMSxjZXg9MS41LGx3ZD0yLCBcbiAgICAgICAgICAgICAgICAgICAgICAgICAgICAgICAgICAgICAgICAgIGJnPWMoXFwjMDA0NDg4XFwpKSlcblxud2l0aChwbG90LmRhdGEuQWdlLmZpdCxsaW5lcyhBZ2VfeW91bmdlcl90LGZpdCxjb2w9cmdiKDAsMCwwLG1heENvbG9yVmFsdWUgPSAyNTUpLGx3ZD0yLGx0eT01KSlcbmBgYFxuYGBgXG5gYGAifQ== -->

```r
```r
```r
eff.Node_younger<-effect(\Age_t\,node.fin, partial.residuals=T)
plot.data.Age.all<-eff.Node_younger$data
plot.data.Age.fit<-with(eff.Node_younger,cbind(x,fit,lower,upper))

plot(3,type=\n\,xlim=c(-2,3),ylim=c(-3,2.5),xaxt='n',ann=F,yaxt='n')
axis(side=2,lwd=2,cex.axis=1.3)
axis(side=1,lwd=2,cex.axis=1.3,at=c(-0.6929692,0.7916849,1.601277,2.181551),labels = c(50,150,250,350))
box(lwd=2)
title(xlab=\AGE_BASE (year)\,ylab=expression(italic(paste(\D\))[PS]),line=3,cex.lab=1.5)
with(plot.data.Age.fit,polygon(x=c(Age_younger_t, rev(Age_younger_t)),y=c(lower, rev(upper)),
                               col=c(\#dcdcdc\), border=NA)) 

with(dist[dist$Species.x==\Ane\,], points(x=jitter(Age_younger_t),y=Dps_t,pch=21,cex=1.5,lwd=2,
                                          bg=c(\#bb5566\)))

with(dist[dist$Species.x==\Oxa\,], points(x=jitter(Age_younger_t),y=Dps_t,pch=21,cex=1.5,lwd=2, 
                                          bg=c(\#ddaa33\)))
with(dist[dist$Species.x==\Pol\,], points(x=jitter(Age_younger_t),y=Dps_t,pch=21,cex=1.5,lwd=2, 
                                          bg=c(\#004488\)))

with(plot.data.Age.fit,lines(Age_younger_t,fit,col=rgb(0,0,0,maxColorValue = 255),lwd=2,lty=5))
```


Population graph


```
```r
eff.Node_younger<-effect(\Age_t\,node.fin, partial.residuals=T)
plot.data.Age.all<-eff.Node_younger$data
plot.data.Age.fit<-with(eff.Node_younger,cbind(x,fit,lower,upper))

plot(3,type=\n\,xlim=c(-2,3),ylim=c(-3,2.5),xaxt='n',ann=F,yaxt='n')
axis(side=2,lwd=2,cex.axis=1.3)
axis(side=1,lwd=2,cex.axis=1.3,at=c(-0.6929692,0.7916849,1.601277,2.181551),labels = c(50,150,250,350))
box(lwd=2)
title(xlab=\AGE_BASE (year)\,ylab=expression(italic(paste(\D\))[PS]),line=3,cex.lab=1.5)
#with(plot.data.Age.fit,polygon(x=c(Age_younger_t, rev(Age_younger_t)),y=c(lower, rev(upper)),
#                               col=c(\#dcdcdc\), border=NA)) 

with(dist[dist$Species.x==\Ane\,], points(x=jitter(Age_younger_t),y=Dps_t,pch=19,cex=1.5,
                                        col=c(\#bb5566\)))

with(dist[dist$Species.x==\Oxa\,], points(x=jitter(Age_younger_t),y=Dps_t,pch=19,cex=1.5, 
                                          col=c(\#ddaa33\)))
with(dist[dist$Species.x==\Pol\,], points(x=jitter(Age_younger_t),y=Dps_t,pch=19,cex=1.5,
                                          col=c(\#004488\)))

with(plot.data.Age.fit,lines(Age_younger_t,fit,col=rgb(0,0,0,maxColorValue = 255),lwd=2,lty=5))
```

```
<!-- rnb-source-end -->

<!-- rnb-chunk-end -->


<!-- rnb-text-begin -->

## Figure_S9

<!-- rnb-text-end -->


<!-- rnb-chunk-begin -->


<!-- rnb-source-begin eyJkYXRhIjoiYGBgclxuYGBgclxucGFyKG1mcm93PWMoMSwzKSxtYXI9Yyg1LDUsMiwyKSlcbmVmZi5Ob2RlX3lvdW5nZXI8LWVmZmVjdChcXEFnZV90XFwsbm9kZS5maW4sIHBhcnRpYWwucmVzaWR1YWxzPVQpXG5wbG90LmRhdGEuQWdlLmFsbDwtZWZmLk5vZGVfeW91bmdlciRkYXRhXG5wbG90LmRhdGEuQWdlLmZpdDwtd2l0aChlZmYuTm9kZV95b3VuZ2VyLGNiaW5kKHgsZml0LGxvd2VyLHVwcGVyKSlcblxuI21lYW48LW1lYW4oYm94Y294KE5vZGUkQWdlX2FicykpXG4jc2Q8LXNkKGJveGNveChOb2RlJEFnZV9hYnMpKVxuIygoNTBeMC40NS0xKS8wLjQ1LW1lYW4pL3NkXG4jKCgxNTBeMC40NS0xKS8wLjQ1LW1lYW4pL3NkXG4jKCgyNTBeMC40NS0xKS8wLjQ1LW1lYW4pL3NkXG4jKCgzNTBeMC40NS0xKS8wLjQ1LW1lYW4pL3NkXG5cbnBsb3QoMyx0eXBlPVxcblxcLHhsaW09YygtMiwyKSx5bGltPWMoLTMsMi41KSx4YXh0PSduJyxhbm49Rix5YXh0PSduJylcbmF4aXMoc2lkZT0yLGx3ZD0yLGNleC5heGlzPTEuNSlcbmF4aXMoc2lkZT0xLGx3ZD0yLGNleC5heGlzPTEuNSxhdD1jKC0xLjEwMDUzNSwwLjE1OTEzOSwxLjAzNzEyNCwxLjczNjAyNyksbGFiZWxzID0gYyg1MCwxNTAsMjUwLDM1MCkpXG5ib3gobHdkPTIpXG5cbiMjbm9kZS5maW48LWxtZShjbG9zZW5lc3NfdH5BZ2VfdCxyYW5kb209fjF8TFcsZGF0YT1Ob2RlKVxuXG50aXRsZSh4bGFiPVxcUE9QX0FHRSAoeWVhcilcXCx5bGFiPWV4cHJlc3Npb24oaXRhbGljKHBhc3RlKFxcTkhjXFwpKSksbGluZT0zLGNleC5sYWI9MS41KVxuI3dpdGgocGxvdC5kYXRhLkFnZS5maXQscG9seWdvbih4PWMoQWdlX3QsIHJldihBZ2VfdCkpLHk9Yyhsb3dlciwgcmV2KHVwcGVyKSksXG4jICAgICAgICAgICAgICAgICAgICAgICAgICAgICAgIGNvbD1jKFxcI2RjZGNkY1xcKSwgYm9yZGVyPU5BKSkgXG5cbndpdGgoTm9kZVtOb2RlJFNwZWNpZXM9PVxcQW5lXFwsXSwgcG9pbnRzKHg9aml0dGVyKEFnZV90KSx5PWNsb3NlbmVzc190LHBjaD0xOSxjZXg9MixcbiAgICAgICAgICAgICAgICAgICAgICAgICAgICAgICAgICAgICAgICBjb2w9YyhcXCNiYjU1NjZcXCkpKVxuXG53aXRoKE5vZGVbTm9kZSRTcGVjaWVzPT1cXE94YVxcLF0sIHBvaW50cyh4PWppdHRlcihBZ2VfdCkseT1jbG9zZW5lc3NfdCxwY2g9MTksY2V4PTIsIFxuICAgICAgICAgICAgICAgICAgICAgICAgICAgICAgICAgICAgICAgIGNvbD1jKFxcI2RkYWEzM1xcKSkpXG53aXRoKE5vZGVbTm9kZSRTcGVjaWVzPT1cXFBvbFxcLF0sIHBvaW50cyh4PWppdHRlcihBZ2VfdCkseT1jbG9zZW5lc3NfdCxwY2g9MTksY2V4PTIsIFxuICAgICAgICAgICAgICAgICAgICAgICAgICAgICAgICAgICAgICAgIGNvbD1jKFxcIzAwNDQ4OFxcKSkpXG5cbndpdGgocGxvdC5kYXRhLkFnZS5maXQsbGluZXMoQWdlX3QsZml0LGNvbD1yZ2IoMCwwLDAsbWF4Q29sb3JWYWx1ZSA9IDI1NSksbHdkPTIsbHR5PTUpKVxubXRleHQoYWRqPS0wLjIsXFwoQSlcXCxjZXg9MS41KVxuZ3JhcGhpY3M6OmxlZ2VuZCgtMiwtMixsZWdlbmQ9ZXhwcmVzc2lvbihpdGFsaWMoQS5uZW0uKSxpdGFsaWMoTy5hY2UuKSxpdGFsaWMoUC5tdWwuKSxcXHAgPiAwLjA1XFwsXFxwXFw8PVxcMC4wNVxcKSxidHk9J24nLFxuICAgICAgICAgICAgICAgICBwY2g9YygxOSwxOSwxOSxOQSxOQSksY29sPWMoXFwjYmI1NTY2XFwsXFwjZGRhYTMzXFwsXFwjMDA0NDg4XFwsXFxibGFja1xcLFxcYmxhY2tcXCksbHR5PWMoTkEsTkEsTkEsNSwxKSxcbiAgICAgICAgICAgICAgICAgc2VnLmxlbj0wLjUsY2V4PTEuMyx4LmludGVyc3A9MC4xLHkuaW50ZXJzcD0wLjYpXG5cblxuIyNsaW5rLmZpbjwtbG1lKERpZmZfR19QX3R+QWdlX3lvdW5nZXJfdCxyYW5kb209fjF8TFcueCxjb3JyZWxhdGlvbj1jb3JNTFBFKGZvcm09fm5vZGVfMStub2RlXzJ8TFcueCksZGF0YT1MaW5rKVxuI21lYW48LW1lYW4oYm94Y294KExpbmskQWdlX2Fic195KSkjIyBsYW1iZGE9MC4xNSwgYWRkZWQ9MFxuI3NkPC1zZChib3hjb3goTGluayRBZ2VfYWJzX3kpKVxuIygoNTBeMC4xNS0xKS8wLjE1LW1lYW4pL3NkXG4jKCgxNTBeMC4xNS0xKS8wLjE1LW1lYW4pL3NkXG4jKCgyNTBeMC4xNS0xKS8wLjE1LW1lYW4pL3NkXG4jKCgzNTBeMC4xNS0xKS8wLjE1LW1lYW4pL3NkXG5cbmVmZi5MaW5rX3lvdW5nZXI8LWVmZmVjdChcXEFnZV95b3VuZ2VyX3RcXCxsaW5rLmZpbiwgcGFydGlhbC5yZXNpZHVhbHM9VClcbnBsb3QuZGF0YS5BZ2UuYWxsPC1lZmYuTGlua195b3VuZ2VyJGRhdGFcbnBsb3QuZGF0YS5BZ2UuZml0PC13aXRoKGVmZi5MaW5rX3lvdW5nZXIsY2JpbmQoeCxmaXQsbG93ZXIsdXBwZXIpKVxucGxvdCgzLHR5cGU9XFxuXFwseGxpbT1jKC0yLDIuNSkseWxpbT1jKC0zLDIuNSkseGF4dD0nbicsYW5uPUYseWF4dD0nbicpXG5heGlzKHNpZGU9Mixsd2Q9MixjZXguYXhpcz0xLjUpXG5heGlzKHNpZGU9MSxsd2Q9MixjZXguYXhpcz0xLjUsYXQ9YygtMC43MDc1NDczLDAuODA5NDE4OCwxLjYwNDU1NSwyLjE2MjU4NCksbGFiZWxzID0gYyg1MCwxNTAsMjUwLDM1MCkpXG5ib3gobHdkPTIpXG5cbiMjbm9kZS5maW48LWxtZShjbG9zZW5lc3NfdH5BZ2VfdCxyYW5kb209fjF8TFcsZGF0YT1Ob2RlKVxuXG50aXRsZSh4bGFiPVxcQUdFX0JBU0UgKHllYXIpXFwseWxhYj1leHByZXNzaW9uKGl0YWxpYyhwYXN0ZShcXERJRkZfR0VOX0dFT1xcKSkpLGxpbmU9MyxjZXgubGFiPTEuNSlcbndpdGgocGxvdC5kYXRhLkFnZS5maXQscG9seWdvbih4PWMoQWdlX3lvdW5nZXJfdCwgcmV2KEFnZV95b3VuZ2VyX3QpKSx5PWMobG93ZXIsIHJldih1cHBlcikpLFxuICAgICAgICAgICAgICAgICAgICAgICAgICAgICAgIGNvbD1jKFxcI2RjZGNkY1xcKSwgYm9yZGVyPU5BKSkgXG5cbndpdGgoTGlua1tMaW5rJFNwZWNpZXM9PVxcQW5lXFwsXSwgcG9pbnRzKHg9aml0dGVyKEFnZV95b3VuZ2VyX3QpLHk9RGlmZl9HX1BfdCxwY2g9MjEsY2V4PTIsbHdkPTEuNSxcbiAgICAgICAgICAgICAgICAgICAgICAgICAgICAgICAgICAgICAgICBiZz1jKFxcI2JiNTU2NlxcKSkpXG5cbndpdGgoTGlua1tMaW5rJFNwZWNpZXM9PVxcT3hhXFwsXSwgcG9pbnRzKHg9aml0dGVyKEFnZV95b3VuZ2VyX3QpLHk9RGlmZl9HX1BfdCxwY2g9MjEsY2V4PTIsbHdkPTEuNSwgXG4gICAgICAgICAgICAgICAgICAgICAgICAgICAgICAgICAgICAgICAgYmc9YyhcXCNkZGFhMzNcXCkpKVxud2l0aChMaW5rW0xpbmskU3BlY2llcz09XFxQb2xcXCxdLCBwb2ludHMoeD1qaXR0ZXIoQWdlX3lvdW5nZXJfdCkseT1EaWZmX0dfUF90LHBjaD0yMSxjZXg9Mixsd2Q9MS41LCBcbiAgICAgICAgICAgICAgICAgICAgICAgICAgICAgICAgICAgICAgICBiZz1jKFxcIzAwNDQ4OFxcKSkpXG5cbndpdGgocGxvdC5kYXRhLkFnZS5maXQsbGluZXMoQWdlX3lvdW5nZXJfdCxmaXQsY29sPXJnYigwLDAsMCxtYXhDb2xvclZhbHVlID0gMjU1KSxsd2Q9MixsdHk9NSkpXG5tdGV4dChhZGo9LTAuMixcXChCKVxcLGNleD0xLjUpXG4jbGluay5maW4yPC1sbWUoRGlmZl9HX1BfdH5BZ2VfRGlmZl90LHJhbmRvbT1+MXxMVy54LGNvcnJlbGF0aW9uPWNvck1MUEUoZm9ybT1+bm9kZV8xK25vZGVfMnxMVy54KSxkYXRhPUxpbmspXG4jbWVhbjwtbWVhbihib3hjb3goTGluayRBZ2VfRGlmZikpIyMgbGFtYmRhPTAuNywgYWRkZWQ9MFxuI3NkPC1zZChib3hjb3goTGluayRBZ2VfRGlmZikpXG4jKCg1MF4wLjctMSkvMC43LW1lYW4pL3NkXG4jKCgxMDBeMC43LTEpLzAuNy1tZWFuKS9zZFxuIygoMTUwXjAuNy0xKS8wLjctbWVhbikvc2RcbiMoKDIwMF4wLjctMSkvMC43LW1lYW4pL3NkXG4jKCgyNTBeMC43LTEpLzAuNy1tZWFuKS9zZFxuXG5lZmYuTGlua195b3VuZ2VyPC1lZmZlY3QoXFxBZ2VfRGlmZl90XFwsbGluay5maW4yLCBwYXJ0aWFsLnJlc2lkdWFscz1UKVxucGxvdC5kYXRhLkFnZS5hbGw8LWVmZi5MaW5rX3lvdW5nZXIkZGF0YVxucGxvdC5kYXRhLkFnZS5maXQ8LXdpdGgoZWZmLkxpbmtfeW91bmdlcixjYmluZCh4LGZpdCxsb3dlcix1cHBlcikpXG5wbG90KDMsdHlwZT1cXG5cXCx4bGltPWMoLTEuMywyKSx5bGltPWMoLTMsMi41KSx4YXh0PSduJyxhbm49Rix5YXh0PSduJylcbmF4aXMoc2lkZT0yLGx3ZD0yLGNleC5heGlzPTEuNSlcbmF4aXMoc2lkZT0xLGx3ZD0yLGNleC5heGlzPTEuNSxhdD1jKC0wLjIwMTM3NDMsMC4zMTcxMTkyLDAuNzU5Nzc3NywxLjE1OTQxNCwxLjUyOTgzMiksbGFiZWxzID0gYyg1MCwxMDAsMTUwLDIwMCwyNTApKVxuYm94KGx3ZD0yKVxuXG4jI25vZGUuZmluPC1sbWUoY2xvc2VuZXNzX3R+QWdlX3QscmFuZG9tPX4xfExXLGRhdGE9Tm9kZSlcblxudGl0bGUoeGxhYj1cXEFHRV9ESUZGICh5ZWFyKVxcLHlsYWI9ZXhwcmVzc2lvbihpdGFsaWMocGFzdGUoXFxESUZGX0dFTl9HRU9cXCkpKSxsaW5lPTMsY2V4LmxhYj0xLjUpXG53aXRoKHBsb3QuZGF0YS5BZ2UuZml0LHBvbHlnb24oeD1jKEFnZV9EaWZmX3QsIHJldihBZ2VfRGlmZl90KSkseT1jKGxvd2VyLCByZXYodXBwZXIpKSxcbiAgICAgICAgICAgICAgICAgICAgICAgICAgICAgICBjb2w9YyhcXCNkY2RjZGNcXCksIGJvcmRlcj1OQSkpIFxuXG53aXRoKExpbmtbTGluayRTcGVjaWVzPT1cXEFuZVxcLF0sIHBvaW50cyh4PWppdHRlcihBZ2VfRGlmZl90KSx5PURpZmZfR19QX3QscGNoPTIxLGNleD0yLGx3ZD0xLjUsXG4gICAgICAgICAgICAgICAgICAgICAgICAgICAgICAgICAgICAgICAgYmc9YyhcXCNiYjU1NjZcXCkpKVxuXG53aXRoKExpbmtbTGluayRTcGVjaWVzPT1cXE94YVxcLF0sIHBvaW50cyh4PWppdHRlcihBZ2VfRGlmZl90KSx5PURpZmZfR19QX3QscGNoPTIxLGNleD0yLGx3ZD0xLjUsIFxuICAgICAgICAgICAgICAgICAgICAgICAgICAgICAgICAgICAgICAgIGJnPWMoXFwjZGRhYTMzXFwpKSlcbndpdGgoTGlua1tMaW5rJFNwZWNpZXM9PVxcUG9sXFwsXSwgcG9pbnRzKHg9aml0dGVyKEFnZV9EaWZmX3QpLHk9RGlmZl9HX1BfdCxwY2g9MjEsY2V4PTIsbHdkPTEuNSwgXG4gICAgICAgICAgICAgICAgICAgICAgICAgICAgICAgICAgICAgICAgYmc9YyhcXCMwMDQ0ODhcXCkpKVxuXG53aXRoKHBsb3QuZGF0YS5BZ2UuZml0LGxpbmVzKEFnZV9EaWZmX3QsZml0LGNvbD1yZ2IoMCwwLDAsbWF4Q29sb3JWYWx1ZSA9IDI1NSksbHdkPTIsbHR5PTUpKVxubXRleHQoYWRqPS0wLjIsXFwoQylcXCxjZXg9MS41KVxuXG5cbmBgYFxuYGBgIn0= -->

```r
```r
par(mfrow=c(1,3),mar=c(5,5,2,2))
eff.Node_younger<-effect(\Age_t\,node.fin, partial.residuals=T)
plot.data.Age.all<-eff.Node_younger$data
plot.data.Age.fit<-with(eff.Node_younger,cbind(x,fit,lower,upper))

#mean<-mean(boxcox(Node$Age_abs))
#sd<-sd(boxcox(Node$Age_abs))
#((50^0.45-1)/0.45-mean)/sd
#((150^0.45-1)/0.45-mean)/sd
#((250^0.45-1)/0.45-mean)/sd
#((350^0.45-1)/0.45-mean)/sd

plot(3,type=\n\,xlim=c(-2,2),ylim=c(-3,2.5),xaxt='n',ann=F,yaxt='n')
axis(side=2,lwd=2,cex.axis=1.5)
axis(side=1,lwd=2,cex.axis=1.5,at=c(-1.100535,0.159139,1.037124,1.736027),labels = c(50,150,250,350))
box(lwd=2)

##node.fin<-lme(closeness_t~Age_t,random=~1|LW,data=Node)

title(xlab=\POP_AGE (year)\,ylab=expression(italic(paste(\NHc\))),line=3,cex.lab=1.5)
#with(plot.data.Age.fit,polygon(x=c(Age_t, rev(Age_t)),y=c(lower, rev(upper)),
#                               col=c(\#dcdcdc\), border=NA)) 

with(Node[Node$Species==\Ane\,], points(x=jitter(Age_t),y=closeness_t,pch=19,cex=2,
                                        col=c(\#bb5566\)))

with(Node[Node$Species==\Oxa\,], points(x=jitter(Age_t),y=closeness_t,pch=19,cex=2, 
                                        col=c(\#ddaa33\)))
with(Node[Node$Species==\Pol\,], points(x=jitter(Age_t),y=closeness_t,pch=19,cex=2, 
                                        col=c(\#004488\)))

with(plot.data.Age.fit,lines(Age_t,fit,col=rgb(0,0,0,maxColorValue = 255),lwd=2,lty=5))
mtext(adj=-0.2,\(A)\,cex=1.5)
graphics::legend(-2,-2,legend=expression(italic(A.nem.),italic(O.ace.),italic(P.mul.),\p > 0.05\,\p\<=\0.05\),bty='n',
                 pch=c(19,19,19,NA,NA),col=c(\#bb5566\,\#ddaa33\,\#004488\,\black\,\black\),lty=c(NA,NA,NA,5,1),
                 seg.len=0.5,cex=1.3,x.intersp=0.1,y.intersp=0.6)


##link.fin<-lme(Diff_G_P_t~Age_younger_t,random=~1|LW.x,correlation=corMLPE(form=~node_1+node_2|LW.x),data=Link)
#mean<-mean(boxcox(Link$Age_abs_y))## lambda=0.15, added=0
#sd<-sd(boxcox(Link$Age_abs_y))
#((50^0.15-1)/0.15-mean)/sd
#((150^0.15-1)/0.15-mean)/sd
#((250^0.15-1)/0.15-mean)/sd
#((350^0.15-1)/0.15-mean)/sd

eff.Link_younger<-effect(\Age_younger_t\,link.fin, partial.residuals=T)
plot.data.Age.all<-eff.Link_younger$data
plot.data.Age.fit<-with(eff.Link_younger,cbind(x,fit,lower,upper))
plot(3,type=\n\,xlim=c(-2,2.5),ylim=c(-3,2.5),xaxt='n',ann=F,yaxt='n')
axis(side=2,lwd=2,cex.axis=1.5)
axis(side=1,lwd=2,cex.axis=1.5,at=c(-0.7075473,0.8094188,1.604555,2.162584),labels = c(50,150,250,350))
box(lwd=2)

##node.fin<-lme(closeness_t~Age_t,random=~1|LW,data=Node)

title(xlab=\AGE_BASE (year)\,ylab=expression(italic(paste(\DIFF_GEN_GEO\))),line=3,cex.lab=1.5)
with(plot.data.Age.fit,polygon(x=c(Age_younger_t, rev(Age_younger_t)),y=c(lower, rev(upper)),
                               col=c(\#dcdcdc\), border=NA)) 

with(Link[Link$Species==\Ane\,], points(x=jitter(Age_younger_t),y=Diff_G_P_t,pch=21,cex=2,lwd=1.5,
                                        bg=c(\#bb5566\)))

with(Link[Link$Species==\Oxa\,], points(x=jitter(Age_younger_t),y=Diff_G_P_t,pch=21,cex=2,lwd=1.5, 
                                        bg=c(\#ddaa33\)))
with(Link[Link$Species==\Pol\,], points(x=jitter(Age_younger_t),y=Diff_G_P_t,pch=21,cex=2,lwd=1.5, 
                                        bg=c(\#004488\)))

with(plot.data.Age.fit,lines(Age_younger_t,fit,col=rgb(0,0,0,maxColorValue = 255),lwd=2,lty=5))
mtext(adj=-0.2,\(B)\,cex=1.5)
#link.fin2<-lme(Diff_G_P_t~Age_Diff_t,random=~1|LW.x,correlation=corMLPE(form=~node_1+node_2|LW.x),data=Link)
#mean<-mean(boxcox(Link$Age_Diff))## lambda=0.7, added=0
#sd<-sd(boxcox(Link$Age_Diff))
#((50^0.7-1)/0.7-mean)/sd
#((100^0.7-1)/0.7-mean)/sd
#((150^0.7-1)/0.7-mean)/sd
#((200^0.7-1)/0.7-mean)/sd
#((250^0.7-1)/0.7-mean)/sd

eff.Link_younger<-effect(\Age_Diff_t\,link.fin2, partial.residuals=T)
plot.data.Age.all<-eff.Link_younger$data
plot.data.Age.fit<-with(eff.Link_younger,cbind(x,fit,lower,upper))
plot(3,type=\n\,xlim=c(-1.3,2),ylim=c(-3,2.5),xaxt='n',ann=F,yaxt='n')
axis(side=2,lwd=2,cex.axis=1.5)
axis(side=1,lwd=2,cex.axis=1.5,at=c(-0.2013743,0.3171192,0.7597777,1.159414,1.529832),labels = c(50,100,150,200,250))
box(lwd=2)

##node.fin<-lme(closeness_t~Age_t,random=~1|LW,data=Node)

title(xlab=\AGE_DIFF (year)\,ylab=expression(italic(paste(\DIFF_GEN_GEO\))),line=3,cex.lab=1.5)
with(plot.data.Age.fit,polygon(x=c(Age_Diff_t, rev(Age_Diff_t)),y=c(lower, rev(upper)),
                               col=c(\#dcdcdc\), border=NA)) 

with(Link[Link$Species==\Ane\,], points(x=jitter(Age_Diff_t),y=Diff_G_P_t,pch=21,cex=2,lwd=1.5,
                                        bg=c(\#bb5566\)))

with(Link[Link$Species==\Oxa\,], points(x=jitter(Age_Diff_t),y=Diff_G_P_t,pch=21,cex=2,lwd=1.5, 
                                        bg=c(\#ddaa33\)))
with(Link[Link$Species==\Pol\,], points(x=jitter(Age_Diff_t),y=Diff_G_P_t,pch=21,cex=2,lwd=1.5, 
                                        bg=c(\#004488\)))

with(plot.data.Age.fit,lines(Age_Diff_t,fit,col=rgb(0,0,0,maxColorValue = 255),lwd=2,lty=5))
mtext(adj=-0.2,\(C)\,cex=1.5)
```

```
<!-- rnb-source-end -->

<!-- rnb-chunk-end -->


<!-- rnb-text-begin -->


#Figure_S9

<!-- rnb-text-end -->


<!-- rnb-chunk-begin -->


<!-- rnb-source-begin eyJkYXRhIjoiYGBgclxucGFyKG1mcm93PWMoMSwzKSxtYXI9Yyg1LDUsMiwyKSlcbmVmZi5Ob2RlX3lvdW5nZXI8LWVmZmVjdChcIkFnZV90XCIsbm9kZS5maW4sIHBhcnRpYWwucmVzaWR1YWxzPVQpXG5wbG90LmRhdGEuQWdlLmFsbDwtZWZmLk5vZGVfeW91bmdlciRkYXRhXG5wbG90LmRhdGEuQWdlLmZpdDwtd2l0aChlZmYuTm9kZV95b3VuZ2VyLGNiaW5kKHgsZml0LGxvd2VyLHVwcGVyKSlcblxuI21lYW48LW1lYW4oYm94Y294KE5vZGUkQWdlX2FicykpXG4jc2Q8LXNkKGJveGNveChOb2RlJEFnZV9hYnMpKVxuIygoNTBeMC40NS0xKS8wLjQ1LW1lYW4pL3NkXG4jKCgxNTBeMC40NS0xKS8wLjQ1LW1lYW4pL3NkXG4jKCgyNTBeMC40NS0xKS8wLjQ1LW1lYW4pL3NkXG4jKCgzNTBeMC40NS0xKS8wLjQ1LW1lYW4pL3NkXG5cbnBsb3QoMyx0eXBlPVwiblwiLHhsaW09YygtMiwyKSx5bGltPWMoLTMsMi41KSx4YXh0PSduJyxhbm49Rix5YXh0PSduJylcbmF4aXMoc2lkZT0yLGx3ZD0yLGNleC5heGlzPTEuNSlcbmF4aXMoc2lkZT0xLGx3ZD0yLGNleC5heGlzPTEuNSxhdD1jKC0xLjEwMDUzNSwwLjE1OTEzOSwxLjAzNzEyNCwxLjczNjAyNyksbGFiZWxzID0gYyg1MCwxNTAsMjUwLDM1MCkpXG5ib3gobHdkPTIpXG5cbiMjbm9kZS5maW48LWxtZShjbG9zZW5lc3NfdH5BZ2VfdCxyYW5kb209fjF8TFcsZGF0YT1Ob2RlKVxuXG50aXRsZSh4bGFiPVwiUE9QX0FHRSAoeWVhcilcIix5bGFiPWV4cHJlc3Npb24oaXRhbGljKHBhc3RlKFwiTkhjXCIpKSksbGluZT0zLGNleC5sYWI9MS41KVxuI3dpdGgocGxvdC5kYXRhLkFnZS5maXQscG9seWdvbih4PWMoQWdlX3QsIHJldihBZ2VfdCkpLHk9Yyhsb3dlciwgcmV2KHVwcGVyKSksXG4jICAgICAgICAgICAgICAgICAgICAgICAgICAgICAgIGNvbD1jKFwiI2RjZGNkY1wiKSwgYm9yZGVyPU5BKSkgXG5cbndpdGgoTm9kZVtOb2RlJFNwZWNpZXM9PVwiQW5lXCIsXSwgcG9pbnRzKHg9aml0dGVyKEFnZV90KSx5PWNsb3NlbmVzc190LHBjaD0xOSxjZXg9MixcbiAgICAgICAgICAgICAgICAgICAgICAgICAgICAgICAgICAgICAgICBjb2w9YyhcIiNiYjU1NjZcIikpKVxuXG53aXRoKE5vZGVbTm9kZSRTcGVjaWVzPT1cIk94YVwiLF0sIHBvaW50cyh4PWppdHRlcihBZ2VfdCkseT1jbG9zZW5lc3NfdCxwY2g9MTksY2V4PTIsIFxuICAgICAgICAgICAgICAgICAgICAgICAgICAgICAgICAgICAgICAgIGNvbD1jKFwiI2RkYWEzM1wiKSkpXG53aXRoKE5vZGVbTm9kZSRTcGVjaWVzPT1cIlBvbFwiLF0sIHBvaW50cyh4PWppdHRlcihBZ2VfdCkseT1jbG9zZW5lc3NfdCxwY2g9MTksY2V4PTIsIFxuICAgICAgICAgICAgICAgICAgICAgICAgICAgICAgICAgICAgICAgIGNvbD1jKFwiIzAwNDQ4OFwiKSkpXG5cbndpdGgocGxvdC5kYXRhLkFnZS5maXQsbGluZXMoQWdlX3QsZml0LGNvbD1yZ2IoMCwwLDAsbWF4Q29sb3JWYWx1ZSA9IDI1NSksbHdkPTIsbHR5PTUpKVxubXRleHQoYWRqPS0wLjIsXCIoQSlcIixjZXg9MS41KVxuZ3JhcGhpY3M6OmxlZ2VuZCgtMiwtMixsZWdlbmQ9ZXhwcmVzc2lvbihpdGFsaWMoQS5uZW0uKSxpdGFsaWMoTy5hY2UuKSxpdGFsaWMoUC5tdWwuKSxcInAgPiAwLjA1XCIsXCJwXCI8PVwiMC4wNVwiKSxidHk9J24nLFxuICAgICAgICAgICAgICAgICBwY2g9YygxOSwxOSwxOSxOQSxOQSksY29sPWMoXCIjYmI1NTY2XCIsXCIjZGRhYTMzXCIsXCIjMDA0NDg4XCIsXCJibGFja1wiLFwiYmxhY2tcIiksbHR5PWMoTkEsTkEsTkEsNSwxKSxcbiAgICAgICAgICAgICAgICAgc2VnLmxlbj0wLjUsY2V4PTEuMyx4LmludGVyc3A9MC4xLHkuaW50ZXJzcD0wLjYpXG5cblxuIyNsaW5rLmZpbjwtbG1lKERpZmZfR19QX3R+QWdlX3lvdW5nZXJfdCxyYW5kb209fjF8TFcueCxjb3JyZWxhdGlvbj1jb3JNTFBFKGZvcm09fm5vZGVfMStub2RlXzJ8TFcueCksZGF0YT1MaW5rKVxuI21lYW48LW1lYW4oYm94Y294KExpbmskQWdlX2Fic195KSkjIyBsYW1iZGE9MC4xNSwgYWRkZWQ9MFxuI3NkPC1zZChib3hjb3goTGluayRBZ2VfYWJzX3kpKVxuIygoNTBeMC4xNS0xKS8wLjE1LW1lYW4pL3NkXG4jKCgxNTBeMC4xNS0xKS8wLjE1LW1lYW4pL3NkXG4jKCgyNTBeMC4xNS0xKS8wLjE1LW1lYW4pL3NkXG4jKCgzNTBeMC4xNS0xKS8wLjE1LW1lYW4pL3NkXG5cbmVmZi5MaW5rX3lvdW5nZXI8LWVmZmVjdChcIkFnZV95b3VuZ2VyX3RcIixsaW5rLmZpbiwgcGFydGlhbC5yZXNpZHVhbHM9VClcbnBsb3QuZGF0YS5BZ2UuYWxsPC1lZmYuTGlua195b3VuZ2VyJGRhdGFcbnBsb3QuZGF0YS5BZ2UuZml0PC13aXRoKGVmZi5MaW5rX3lvdW5nZXIsY2JpbmQoeCxmaXQsbG93ZXIsdXBwZXIpKVxucGxvdCgzLHR5cGU9XCJuXCIseGxpbT1jKC0yLDIuNSkseWxpbT1jKC0zLDIuNSkseGF4dD0nbicsYW5uPUYseWF4dD0nbicpXG5heGlzKHNpZGU9Mixsd2Q9MixjZXguYXhpcz0xLjUpXG5heGlzKHNpZGU9MSxsd2Q9MixjZXguYXhpcz0xLjUsYXQ9YygtMC43MDc1NDczLDAuODA5NDE4OCwxLjYwNDU1NSwyLjE2MjU4NCksbGFiZWxzID0gYyg1MCwxNTAsMjUwLDM1MCkpXG5ib3gobHdkPTIpXG5cbiMjbm9kZS5maW48LWxtZShjbG9zZW5lc3NfdH5BZ2VfdCxyYW5kb209fjF8TFcsZGF0YT1Ob2RlKVxuXG50aXRsZSh4bGFiPVwiQUdFX0JBU0UgKHllYXIpXCIseWxhYj1leHByZXNzaW9uKGl0YWxpYyhwYXN0ZShcIkRJRkZfR0VOX0dFT1wiKSkpLGxpbmU9MyxjZXgubGFiPTEuNSlcbndpdGgocGxvdC5kYXRhLkFnZS5maXQscG9seWdvbih4PWMoQWdlX3lvdW5nZXJfdCwgcmV2KEFnZV95b3VuZ2VyX3QpKSx5PWMobG93ZXIsIHJldih1cHBlcikpLFxuICAgICAgICAgICAgICAgICAgICAgICAgICAgICAgIGNvbD1jKFwiI2RjZGNkY1wiKSwgYm9yZGVyPU5BKSkgXG5cbndpdGgoTGlua1tMaW5rJFNwZWNpZXM9PVwiQW5lXCIsXSwgcG9pbnRzKHg9aml0dGVyKEFnZV95b3VuZ2VyX3QpLHk9RGlmZl9HX1BfdCxwY2g9MjEsY2V4PTIsbHdkPTEuNSxcbiAgICAgICAgICAgICAgICAgICAgICAgICAgICAgICAgICAgICAgICBiZz1jKFwiI2JiNTU2NlwiKSkpXG5cbndpdGgoTGlua1tMaW5rJFNwZWNpZXM9PVwiT3hhXCIsXSwgcG9pbnRzKHg9aml0dGVyKEFnZV95b3VuZ2VyX3QpLHk9RGlmZl9HX1BfdCxwY2g9MjEsY2V4PTIsbHdkPTEuNSwgXG4gICAgICAgICAgICAgICAgICAgICAgICAgICAgICAgICAgICAgICAgYmc9YyhcIiNkZGFhMzNcIikpKVxud2l0aChMaW5rW0xpbmskU3BlY2llcz09XCJQb2xcIixdLCBwb2ludHMoeD1qaXR0ZXIoQWdlX3lvdW5nZXJfdCkseT1EaWZmX0dfUF90LHBjaD0yMSxjZXg9Mixsd2Q9MS41LCBcbiAgICAgICAgICAgICAgICAgICAgICAgICAgICAgICAgICAgICAgICBiZz1jKFwiIzAwNDQ4OFwiKSkpXG5cbndpdGgocGxvdC5kYXRhLkFnZS5maXQsbGluZXMoQWdlX3lvdW5nZXJfdCxmaXQsY29sPXJnYigwLDAsMCxtYXhDb2xvclZhbHVlID0gMjU1KSxsd2Q9MixsdHk9NSkpXG5tdGV4dChhZGo9LTAuMixcIihCKVwiLGNleD0xLjUpXG4jbGluay5maW4yPC1sbWUoRGlmZl9HX1BfdH5BZ2VfRGlmZl90LHJhbmRvbT1+MXxMVy54LGNvcnJlbGF0aW9uPWNvck1MUEUoZm9ybT1+bm9kZV8xK25vZGVfMnxMVy54KSxkYXRhPUxpbmspXG4jbWVhbjwtbWVhbihib3hjb3goTGluayRBZ2VfRGlmZikpIyMgbGFtYmRhPTAuNywgYWRkZWQ9MFxuI3NkPC1zZChib3hjb3goTGluayRBZ2VfRGlmZikpXG4jKCg1MF4wLjctMSkvMC43LW1lYW4pL3NkXG4jKCgxMDBeMC43LTEpLzAuNy1tZWFuKS9zZFxuIygoMTUwXjAuNy0xKS8wLjctbWVhbikvc2RcbiMoKDIwMF4wLjctMSkvMC43LW1lYW4pL3NkXG4jKCgyNTBeMC43LTEpLzAuNy1tZWFuKS9zZFxuXG5lZmYuTGlua195b3VuZ2VyPC1lZmZlY3QoXCJBZ2VfRGlmZl90XCIsbGluay5maW4yLCBwYXJ0aWFsLnJlc2lkdWFscz1UKVxucGxvdC5kYXRhLkFnZS5hbGw8LWVmZi5MaW5rX3lvdW5nZXIkZGF0YVxucGxvdC5kYXRhLkFnZS5maXQ8LXdpdGgoZWZmLkxpbmtfeW91bmdlcixjYmluZCh4LGZpdCxsb3dlcix1cHBlcikpXG5wbG90KDMsdHlwZT1cIm5cIix4bGltPWMoLTEuMywyKSx5bGltPWMoLTMsMi41KSx4YXh0PSduJyxhbm49Rix5YXh0PSduJylcbmF4aXMoc2lkZT0yLGx3ZD0yLGNleC5heGlzPTEuNSlcbmF4aXMoc2lkZT0xLGx3ZD0yLGNleC5heGlzPTEuNSxhdD1jKC0wLjIwMTM3NDMsMC4zMTcxMTkyLDAuNzU5Nzc3NywxLjE1OTQxNCwxLjUyOTgzMiksbGFiZWxzID0gYyg1MCwxMDAsMTUwLDIwMCwyNTApKVxuYm94KGx3ZD0yKVxuXG4jI25vZGUuZmluPC1sbWUoY2xvc2VuZXNzX3R+QWdlX3QscmFuZG9tPX4xfExXLGRhdGE9Tm9kZSlcblxudGl0bGUoeGxhYj1cIkFHRV9ESUZGICh5ZWFyKVwiLHlsYWI9ZXhwcmVzc2lvbihpdGFsaWMocGFzdGUoXCJESUZGX0dFTl9HRU9cIikpKSxsaW5lPTMsY2V4LmxhYj0xLjUpXG53aXRoKHBsb3QuZGF0YS5BZ2UuZml0LHBvbHlnb24oeD1jKEFnZV9EaWZmX3QsIHJldihBZ2VfRGlmZl90KSkseT1jKGxvd2VyLCByZXYodXBwZXIpKSxcbiAgICAgICAgICAgICAgICAgICAgICAgICAgICAgICBjb2w9YyhcIiNkY2RjZGNcIiksIGJvcmRlcj1OQSkpIFxuXG53aXRoKExpbmtbTGluayRTcGVjaWVzPT1cIkFuZVwiLF0sIHBvaW50cyh4PWppdHRlcihBZ2VfRGlmZl90KSx5PURpZmZfR19QX3QscGNoPTIxLGNleD0yLGx3ZD0xLjUsXG4gICAgICAgICAgICAgICAgICAgICAgICAgICAgICAgICAgICAgICAgYmc9YyhcIiNiYjU1NjZcIikpKVxuXG53aXRoKExpbmtbTGluayRTcGVjaWVzPT1cIk94YVwiLF0sIHBvaW50cyh4PWppdHRlcihBZ2VfRGlmZl90KSx5PURpZmZfR19QX3QscGNoPTIxLGNleD0yLGx3ZD0xLjUsIFxuICAgICAgICAgICAgICAgICAgICAgICAgICAgICAgICAgICAgICAgIGJnPWMoXCIjZGRhYTMzXCIpKSlcbndpdGgoTGlua1tMaW5rJFNwZWNpZXM9PVwiUG9sXCIsXSwgcG9pbnRzKHg9aml0dGVyKEFnZV9EaWZmX3QpLHk9RGlmZl9HX1BfdCxwY2g9MjEsY2V4PTIsbHdkPTEuNSwgXG4gICAgICAgICAgICAgICAgICAgICAgICAgICAgICAgICAgICAgICAgYmc9YyhcIiMwMDQ0ODhcIikpKVxuXG53aXRoKHBsb3QuZGF0YS5BZ2UuZml0LGxpbmVzKEFnZV9EaWZmX3QsZml0LGNvbD1yZ2IoMCwwLDAsbWF4Q29sb3JWYWx1ZSA9IDI1NSksbHdkPTIsbHR5PTUpKVxubXRleHQoYWRqPS0wLjIsXCIoQylcIixjZXg9MS41KVxuXG5cbmBgYCJ9 -->

```r
par(mfrow=c(1,3),mar=c(5,5,2,2))
eff.Node_younger<-effect("Age_t",node.fin, partial.residuals=T)
plot.data.Age.all<-eff.Node_younger$data
plot.data.Age.fit<-with(eff.Node_younger,cbind(x,fit,lower,upper))

#mean<-mean(boxcox(Node$Age_abs))
#sd<-sd(boxcox(Node$Age_abs))
#((50^0.45-1)/0.45-mean)/sd
#((150^0.45-1)/0.45-mean)/sd
#((250^0.45-1)/0.45-mean)/sd
#((350^0.45-1)/0.45-mean)/sd

plot(3,type="n",xlim=c(-2,2),ylim=c(-3,2.5),xaxt='n',ann=F,yaxt='n')
axis(side=2,lwd=2,cex.axis=1.5)
axis(side=1,lwd=2,cex.axis=1.5,at=c(-1.100535,0.159139,1.037124,1.736027),labels = c(50,150,250,350))
box(lwd=2)

##node.fin<-lme(closeness_t~Age_t,random=~1|LW,data=Node)

title(xlab="POP_AGE (year)",ylab=expression(italic(paste("NHc"))),line=3,cex.lab=1.5)
#with(plot.data.Age.fit,polygon(x=c(Age_t, rev(Age_t)),y=c(lower, rev(upper)),
#                               col=c("#dcdcdc"), border=NA)) 

with(Node[Node$Species=="Ane",], points(x=jitter(Age_t),y=closeness_t,pch=19,cex=2,
                                        col=c("#bb5566")))

with(Node[Node$Species=="Oxa",], points(x=jitter(Age_t),y=closeness_t,pch=19,cex=2, 
                                        col=c("#ddaa33")))
with(Node[Node$Species=="Pol",], points(x=jitter(Age_t),y=closeness_t,pch=19,cex=2, 
                                        col=c("#004488")))

with(plot.data.Age.fit,lines(Age_t,fit,col=rgb(0,0,0,maxColorValue = 255),lwd=2,lty=5))
mtext(adj=-0.2,"(A)",cex=1.5)
graphics::legend(-2,-2,legend=expression(italic(A.nem.),italic(O.ace.),italic(P.mul.),"p > 0.05","p"<="0.05"),bty='n',
                 pch=c(19,19,19,NA,NA),col=c("#bb5566","#ddaa33","#004488","black","black"),lty=c(NA,NA,NA,5,1),
                 seg.len=0.5,cex=1.3,x.intersp=0.1,y.intersp=0.6)


##link.fin<-lme(Diff_G_P_t~Age_younger_t,random=~1|LW.x,correlation=corMLPE(form=~node_1+node_2|LW.x),data=Link)
#mean<-mean(boxcox(Link$Age_abs_y))## lambda=0.15, added=0
#sd<-sd(boxcox(Link$Age_abs_y))
#((50^0.15-1)/0.15-mean)/sd
#((150^0.15-1)/0.15-mean)/sd
#((250^0.15-1)/0.15-mean)/sd
#((350^0.15-1)/0.15-mean)/sd

eff.Link_younger<-effect("Age_younger_t",link.fin, partial.residuals=T)
plot.data.Age.all<-eff.Link_younger$data
plot.data.Age.fit<-with(eff.Link_younger,cbind(x,fit,lower,upper))
plot(3,type="n",xlim=c(-2,2.5),ylim=c(-3,2.5),xaxt='n',ann=F,yaxt='n')
axis(side=2,lwd=2,cex.axis=1.5)
axis(side=1,lwd=2,cex.axis=1.5,at=c(-0.7075473,0.8094188,1.604555,2.162584),labels = c(50,150,250,350))
box(lwd=2)

##node.fin<-lme(closeness_t~Age_t,random=~1|LW,data=Node)

title(xlab="AGE_BASE (year)",ylab=expression(italic(paste("DIFF_GEN_GEO"))),line=3,cex.lab=1.5)
with(plot.data.Age.fit,polygon(x=c(Age_younger_t, rev(Age_younger_t)),y=c(lower, rev(upper)),
                               col=c("#dcdcdc"), border=NA)) 

with(Link[Link$Species=="Ane",], points(x=jitter(Age_younger_t),y=Diff_G_P_t,pch=21,cex=2,lwd=1.5,
                                        bg=c("#bb5566")))

with(Link[Link$Species=="Oxa",], points(x=jitter(Age_younger_t),y=Diff_G_P_t,pch=21,cex=2,lwd=1.5, 
                                        bg=c("#ddaa33")))
with(Link[Link$Species=="Pol",], points(x=jitter(Age_younger_t),y=Diff_G_P_t,pch=21,cex=2,lwd=1.5, 
                                        bg=c("#004488")))

with(plot.data.Age.fit,lines(Age_younger_t,fit,col=rgb(0,0,0,maxColorValue = 255),lwd=2,lty=5))
mtext(adj=-0.2,"(B)",cex=1.5)
#link.fin2<-lme(Diff_G_P_t~Age_Diff_t,random=~1|LW.x,correlation=corMLPE(form=~node_1+node_2|LW.x),data=Link)
#mean<-mean(boxcox(Link$Age_Diff))## lambda=0.7, added=0
#sd<-sd(boxcox(Link$Age_Diff))
#((50^0.7-1)/0.7-mean)/sd
#((100^0.7-1)/0.7-mean)/sd
#((150^0.7-1)/0.7-mean)/sd
#((200^0.7-1)/0.7-mean)/sd
#((250^0.7-1)/0.7-mean)/sd

eff.Link_younger<-effect("Age_Diff_t",link.fin2, partial.residuals=T)
plot.data.Age.all<-eff.Link_younger$data
plot.data.Age.fit<-with(eff.Link_younger,cbind(x,fit,lower,upper))
plot(3,type="n",xlim=c(-1.3,2),ylim=c(-3,2.5),xaxt='n',ann=F,yaxt='n')
axis(side=2,lwd=2,cex.axis=1.5)
axis(side=1,lwd=2,cex.axis=1.5,at=c(-0.2013743,0.3171192,0.7597777,1.159414,1.529832),labels = c(50,100,150,200,250))
box(lwd=2)

##node.fin<-lme(closeness_t~Age_t,random=~1|LW,data=Node)

title(xlab="AGE_DIFF (year)",ylab=expression(italic(paste("DIFF_GEN_GEO"))),line=3,cex.lab=1.5)
with(plot.data.Age.fit,polygon(x=c(Age_Diff_t, rev(Age_Diff_t)),y=c(lower, rev(upper)),
                               col=c("#dcdcdc"), border=NA)) 

with(Link[Link$Species=="Ane",], points(x=jitter(Age_Diff_t),y=Diff_G_P_t,pch=21,cex=2,lwd=1.5,
                                        bg=c("#bb5566")))

with(Link[Link$Species=="Oxa",], points(x=jitter(Age_Diff_t),y=Diff_G_P_t,pch=21,cex=2,lwd=1.5, 
                                        bg=c("#ddaa33")))
with(Link[Link$Species=="Pol",], points(x=jitter(Age_Diff_t),y=Diff_G_P_t,pch=21,cex=2,lwd=1.5, 
                                        bg=c("#004488")))

with(plot.data.Age.fit,lines(Age_Diff_t,fit,col=rgb(0,0,0,maxColorValue = 255),lwd=2,lty=5))
mtext(adj=-0.2,"(C)",cex=1.5)
```


LS0tDQp0aXRsZTogIlJlc3VsdF9WaXN1YWxpemF0aW9uIg0Kb3V0cHV0OiBodG1sX25vdGVib29rDQotLS0NCg0KDQpgYGB7cn0NCmxpYnJhcnkoZHBseXIpDQpsaWJyYXJ5KGVmZmVjdHMpDQpsaWJyYXJ5KHNjYWxlcykNCmxpYnJhcnkocGxvdGx5KQ0KIyNGaWd1cmVfMWItZA0KbG9hZCgiR2VuRGl2X2FsbC5SRGF0YSIpDQoNCmRmPC1HZW5EaXZfYWxsJT4lDQogIHNlbGVjdChwb3B1bGF0aW9uLFNwZWNpZXMueCxMVy54LEFnZV9hYnMpJT4lDQogIGFycmFuZ2UobWF0Y2goTFcueCxjKCJGck4iLCJCZSIsIkdlVyIsIkdlRSIsIlN3IiwiRXMiKSkpDQoNCmRmJExXPC1hcy5udW1lcmljKGZhY3RvcihkZiRMVy54LGxldmVscz1jKCJGck4iLCJCZSIsIkdlVyIsIkdlRSIsIlN3UyIsIkVzdCIpKSkNCg0KDQpwYXIobWZyb3c9YygxLDMpLGJ0eT0iTCIsbWFyPWMoNCw0LDIsMCkpDQpwbG90KDMsdHlwZT0ibiIseGxpbT1jKDAuNSw2LjUpLHlsaW09YygwLDM1MCkseGF4dD0nbicsYW5uPUYseWF4dD0nbicpDQpheGlzKHNpZGU9Mixsd2Q9MixjZXguYXhpcz0xLjUpDQpheGlzKHNpZGU9MSxsd2Q9MixjZXguYXhpcz0xLjMsYXQ9YygxLDIsMyw0LDUsNiksbGFiZWxzID0gYygiRnIiLCJCZSIsIkdlVyIsIkdlRSIsIlN3IiwiRXMiKSkNCmJveChsd2Q9MikNCm10ZXh0KGFkaj0tMC4xLH5pdGFsaWMoIkEuIG5lbW9yb3NhIiksY2V4PTEpDQp0aXRsZSh4bGFiPWMoIkxhbmRzY2FwZSBXaW5kb3ciKSx5bGFiPWMoIlBvcHVsYXRpb24gQWdlICh5cikiKSxsaW5lPTIuNSxjZXgubGFiPTEuNSkNCndpdGgoZGZbZGYkU3BlY2llcy54PT0iQW5lIixdLCBwb2ludHMoeD1qaXR0ZXIoTFcpLHk9QWdlX2FicyxjZXg9MixwY2g9MCxsd2Q9MiwNCiAgICAgICAgICAgICAgICAgICAgICAgICAgICAgICAgICAgICAgY29sPWMoIiNiYjU1NjYiKSkpDQoNCg0KcGxvdCgzLHR5cGU9Im4iLHhsaW09YygwLjUsNC41KSx5bGltPWMoMCwzNTApLHhheHQ9J24nLGFubj1GLHlheHQ9J24nKQ0KYXhpcyhzaWRlPTIsbHdkPTIsY2V4LmF4aXM9MS41KQ0KYXhpcyhzaWRlPTEsbHdkPTIsY2V4LmF4aXM9MS4zLGF0PWMoMSwyLDMsNCksbGFiZWxzID0gYygiR2VXIiwiR2VFIiwiU3ciLCJFcyIpKQ0KYm94KGx3ZD0yKQ0KbXRleHQoYWRqPS0wLjEsfml0YWxpYygiTy4gYWNldG9zZWxsYSIpLGNleD0xKQ0KdGl0bGUoeGxhYj1jKCJMYW5kc2NhcGUgV2luZG93IikseWxhYj1jKCJQb3B1bGF0aW9uIEFnZSAoeXIpIiksbGluZT0yLjUsY2V4LmxhYj0xLjUpDQp3aXRoKGRmW2RmJFNwZWNpZXMueD09Ik94YSIsXSwgcG9pbnRzKHg9aml0dGVyKExXLTIpLHk9QWdlX2FicyxjZXg9MixwY2g9MSxsd2Q9MiwgDQogICAgICAgICAgICAgICAgICAgICAgICAgICAgICAgICAgICAgIGNvbD1jKCIjZGRhYTMzIikpKQ0KDQpwbG90KDMsdHlwZT0ibiIseGxpbT1jKDAuNSw2LjUpLHlsaW09YygwLDM1MCkseGF4dD0nbicsYW5uPUYseWF4dD0nbicpDQpheGlzKHNpZGU9Mixsd2Q9MixjZXguYXhpcz0xLjUpDQpheGlzKHNpZGU9MSxsd2Q9MixjZXguYXhpcz0xLjMsYXQ9YygxLDIsMyw0LDUsNiksbGFiZWxzID0gYygiRnIiLCJCZSIsIkdlVyIsIkdlRSIsIlN3IiwiRXMiKSkNCmJveChsd2Q9MikNCm10ZXh0KGFkaj0tMC4xLH5pdGFsaWMoIlAuIG11bHRpZmxvcnVtIiksY2V4PTEpDQp0aXRsZSh4bGFiPWMoIkxhbmRzY2FwZSBXaW5kb3ciKSx5bGFiPWMoIlBvcHVsYXRpb24gQWdlICh5cikiKSxsaW5lPTIuNSxjZXgubGFiPTEuNSkNCndpdGgoZGZbZGYkU3BlY2llcy54PT0iUG9sIixdLCBwb2ludHMoeD1qaXR0ZXIoTFcpLHk9QWdlX2FicyxjZXg9MS41LHBjaD02LGx3ZD0yLCANCiAgICAgICAgICAgICAgICAgICAgICAgICAgICAgICAgICAgICAgY29sPWMoIiMwMDQ0ODgiKSkpDQoNCiMjRmlndXJlXzMNCmVmZi5Bci5BZ2U8LWVmZmVjdCgiU3BlY2llcy54OkFnZV90IixsbW0uQXIuYi5maW4sIHBhcnRpYWwucmVzaWR1YWxzPVQpDQpwbG90LmRhdGEuQWdlLmFsbDwtZWZmLkFyLkFnZSRkYXRhDQpwbG90LmRhdGEuQWdlLmZpdDwtd2l0aChlZmYuQXIuQWdlLCBjYmluZCh4LGZpdCxsb3dlcix1cHBlcikpDQoNCnBhcihtZnJvdz1jKDEsNCksbWFyPWMoNCw1LDIsMikpDQpwbG90KDMsdHlwZT0ibiIseGxpbT1jKC0yLDIuNSkseWxpbT1jKC0zLDMuMikseGF4dD0nbicsYW5uPUYseWF4dD0nbicpDQpheGlzKHNpZGU9Mixsd2Q9MixjZXguYXhpcz0yKQ0KYXhpcyhzaWRlPTEsbHdkPTIsY2V4LmF4aXM9MixhdD1jKC0xLjE2NTk2LDAuMTU5MTM5LDEuMDM3MTI0LDEuNzM2MDI3KSxsYWJlbHMgPSBjKDUwLDE1MCwyNTAsMzUwKSkNCmJveChsd2Q9MikNCiNtdGV4dChjKCJPIiwiWSIpLGF0PWMoMS4wNSwyLjA1KSxzaWRlPTEpDQp0aXRsZSh4bGFiPWMoIlBPUF9BR0UgKHlyKSIpLHlsYWI9ZXhwcmVzc2lvbihpdGFsaWMoSClbZV0pLGxpbmU9Mi41LGNleC5sYWI9MikNCndpdGgocGxvdC5kYXRhLkFnZS5maXRbcGxvdC5kYXRhLkFnZS5maXQkU3BlY2llcy54PT0iQW5lIixdLHBvbHlnb24oeD1jKEFnZV90W1NwZWNpZXMueD09IkFuZSJdLHJldihBZ2VfdFtTcGVjaWVzLng9PSJBbmUiXSkpLHk9Yyhsb3dlcltTcGVjaWVzLng9PSJBbmUiXSwgcmV2KHVwcGVyW1NwZWNpZXMueD09IkFuZSJdKSksY29sPWFscGhhKCIjYmI1NTY2IiwwLjMpLCBib3JkZXI9TkEpKSANCg0Kd2l0aChHZW5EaXZfYWxsW0dlbkRpdl9hbGwkU3BlY2llcy54PT0iQW5lIixdLCBwb2ludHMoeD1qaXR0ZXIoQWdlX3QpLHk9SGVfdCxjZXg9MixwY2g9MjEsbHdkPTIsDQogICAgICAgICAgICAgICAgICAgICAgICAgICAgICAgICAgICAgICAgICAgICAgICAgICAgICBiZz1jKCIjYmI1NTY2IikpKQ0KDQp3aXRoKEdlbkRpdl9hbGxbR2VuRGl2X2FsbCRTcGVjaWVzLng9PSJPeGEiLF0sIHBvaW50cyh4PWppdHRlcihBZ2VfdCkseT1IZV90LGNleD0yLHBjaD0yMSxsd2Q9MiwgDQogICAgICAgICAgICAgICAgICAgICAgICAgICAgICAgICAgICAgICAgICAgICAgICAgICAgICBiZz1jKCIjZGRhYTMzIikpKQ0Kd2l0aChHZW5EaXZfYWxsW0dlbkRpdl9hbGwkU3BlY2llcy54PT0iUG9sIixdLCBwb2ludHMoeD1qaXR0ZXIoQWdlX3QpLHk9SGVfdCxjZXg9MixwY2g9MjEsbHdkPTIsIA0KICAgICAgICAgICAgICAgICAgICAgICAgICAgICAgICAgICAgICAgICAgICAgICAgICAgICAgYmc9YygiIzAwNDQ4OCIpKSkNCg0Kd2l0aChwbG90LmRhdGEuQWdlLmZpdFtwbG90LmRhdGEuQWdlLmZpdCRTcGVjaWVzLng9PSJBbmUiLF0sbGluZXMoQWdlX3QsZml0LGNvbD1jKCIjYmI1NTY2IiksbHdkPTIpKQ0Kd2l0aChwbG90LmRhdGEuQWdlLmZpdFtwbG90LmRhdGEuQWdlLmZpdCRTcGVjaWVzLng9PSJPeGEiLF0sbGluZXMoQWdlX3QsZml0LGNvbD1jKCIjZGRhYTMzIiksbHdkPTIsbHR5PTUpKQ0Kd2l0aChwbG90LmRhdGEuQWdlLmZpdFtwbG90LmRhdGEuQWdlLmZpdCRTcGVjaWVzLng9PSJQb2wiLF0sbGluZXMoQWdlX3QsZml0LGNvbD1jKCIjMDA0NDg4IiksbHdkPTIsbHR5PTUpKQ0KDQp3aXRoKHBsb3QuZGF0YS5BZ2UuZml0LCB0ZXh0KDIuMiwgeT1jKG1pbihmaXRbU3BlY2llcy54PT0iQW5lIl0pLA0KICAgICAgICAgICAgICAgICAgICAgICAgICAgICAgICAgICAgICBtYXgoZml0W1NwZWNpZXMueD09Ik94YSJdKSwNCiAgICAgICAgICAgICAgICAgICAgICAgICAgICAgICAgICAgICAgbWF4KGZpdFtTcGVjaWVzLng9PSJQb2wiXSkrMC41KSwNCiAgICAgICAgICAgICAgICAgICAgICAgICAgICAgbGFiZWxzPWMoImEiLCAiYiIsImIiKSwgY2V4PTEuNSwgYWRqPTEpKQ0KDQptdGV4dChhZGo9LTAuMSwiKEEpIixjZXg9MS41KQ0KZ3JhcGhpY3M6OmxlZ2VuZCgwLjgsMy42LGxlZ2VuZD1leHByZXNzaW9uKGl0YWxpYyhBLm5lbS4pLGl0YWxpYyhPLmFjZS4pLGl0YWxpYyhQLm11bC4pLCJwID4gMC4wNSIsInAiPD0iMC4wNSIpLGJ0eT0nbicsDQogICAgICAgICAgICAgICAgIHBjaD1jKDIxLDIxLDIxLE5BLE5BKSxwdC5iZz1jKCIjYmI1NTY2IiwiI2RkYWEzMyIsIiMwMDQ0ODgiLE5BLE5BKSxsdHk9YyhOQSxOQSxOQSw1LDEpLA0KICAgICAgICAgICAgICAgICBzZWcubGVuPTAuNSxjZXg9MS41LHguaW50ZXJzcD0wLjEseS5pbnRlcnNwPTAuNikNCg0KDQplZmYuSGUuQWdlPC1lZmZlY3QoIkFnZV90OlNwZWNpZXMueCIsbG1tLkhlLmIuZmluLCBwYXJ0aWFsLnJlc2lkdWFscz1UKQ0KcGxvdC5kYXRhLkFnZS5hbGw8LWVmZi5IZS5BZ2UkZGF0YQ0KcGxvdC5kYXRhLkFnZS5maXQ8LXdpdGgoZWZmLkhlLkFnZSxjYmluZCh4LGZpdCxsb3dlcix1cHBlcikpDQoNCnBhcihtZnJvdz1jKDEsMyksIG1hcj1jKDQsNSwyLDIpKQ0KDQpwbG90KDMsdHlwZT0ibiIseGxpbT1jKC0yLDIuNSkseWxpbT1jKC0zLDMuMikseGF4dD0nbicsYW5uPUYseWF4dD0nbicpDQpheGlzKHNpZGU9Mixsd2Q9MixjZXguYXhpcz0yKQ0KYXhpcyhzaWRlPTEsbHdkPTIsY2V4LmF4aXM9MixhdD1jKC0xLjE2NTk2LDAuMTU5MTM5LDEuMDM3MTI0LDEuNzM2MDI3KSxsYWJlbHMgPSBjKDUwLDE1MCwyNTAsMzUwKSkNCmJveChsd2Q9MikNCiNtdGV4dChjKCJPIiwiWSIpLGF0PWMoMS4wNSwyLjA1KSxzaWRlPTEpDQp0aXRsZSh4bGFiPWMoIlBPUF9BR0UgKHlyKSIpLHlsYWI9ZXhwcmVzc2lvbihpdGFsaWMoSClbZV0pLGxpbmU9Mi41LGNleC5sYWI9MikNCndpdGgocGxvdC5kYXRhLkFnZS5maXRbcGxvdC5kYXRhLkFnZS5maXQkU3BlY2llcy54PT0iQW5lIixdLHBvbHlnb24oeD1jKEFnZV90W1NwZWNpZXMueD09IkFuZSJdLHJldihBZ2VfdFtTcGVjaWVzLng9PSJBbmUiXSkpLHk9Yyhsb3dlcltTcGVjaWVzLng9PSJBbmUiXSwgcmV2KHVwcGVyW1NwZWNpZXMueD09IkFuZSJdKSksY29sPWFscGhhKCIjYmI1NTY2IiwwLjMpLCBib3JkZXI9TkEpKSANCg0Kd2l0aChHZW5EaXZfYWxsW0dlbkRpdl9hbGwkU3BlY2llcy54PT0iQW5lIixdLCBwb2ludHMoeD1qaXR0ZXIoQWdlX3QpLHk9SGVfdCxjZXg9MixwY2g9MjEsbHdkPTIsDQogICAgICAgICAgICAgICAgICAgICAgICAgICAgICAgICAgICAgICAgICAgICAgICAgICAgICBiZz1jKCIjYmI1NTY2IikpKQ0KDQp3aXRoKEdlbkRpdl9hbGxbR2VuRGl2X2FsbCRTcGVjaWVzLng9PSJPeGEiLF0sIHBvaW50cyh4PWppdHRlcihBZ2VfdCkseT1IZV90LGNleD0yLHBjaD0yMSxsd2Q9MiwgDQogICAgICAgICAgICAgICAgICAgICAgICAgICAgICAgICAgICAgICAgICAgICAgICAgICAgICBiZz1jKCIjZGRhYTMzIikpKQ0Kd2l0aChHZW5EaXZfYWxsW0dlbkRpdl9hbGwkU3BlY2llcy54PT0iUG9sIixdLCBwb2ludHMoeD1qaXR0ZXIoQWdlX3QpLHk9SGVfdCxjZXg9MixwY2g9MjEsbHdkPTIsIA0KICAgICAgICAgICAgICAgICAgICAgICAgICAgICAgICAgICAgICAgICAgICAgICAgICAgICAgYmc9YygiIzAwNDQ4OCIpKSkNCg0Kd2l0aChwbG90LmRhdGEuQWdlLmZpdFtwbG90LmRhdGEuQWdlLmZpdCRTcGVjaWVzLng9PSJBbmUiLF0sbGluZXMoQWdlX3QsZml0LGNvbD1jKCIjYmI1NTY2IiksbHdkPTIpKQ0Kd2l0aChwbG90LmRhdGEuQWdlLmZpdFtwbG90LmRhdGEuQWdlLmZpdCRTcGVjaWVzLng9PSJPeGEiLF0sbGluZXMoQWdlX3QsZml0LGNvbD1jKCIjZGRhYTMzIiksbHdkPTIsbHR5PTUpKQ0Kd2l0aChwbG90LmRhdGEuQWdlLmZpdFtwbG90LmRhdGEuQWdlLmZpdCRTcGVjaWVzLng9PSJQb2wiLF0sbGluZXMoQWdlX3QsZml0LGNvbD1jKCIjMDA0NDg4IiksbHdkPTIsbHR5PTUpKQ0KDQp3aXRoKHBsb3QuZGF0YS5BZ2UuZml0LCB0ZXh0KDIuMiwgeT1jKG1pbihmaXRbU3BlY2llcy54PT0iQW5lIl0pLA0KICAgICAgICAgICAgICAgICAgICAgICAgICAgICAgICAgICAgICBtYXgoZml0W1NwZWNpZXMueD09Ik94YSJdKSwNCiAgICAgICAgICAgICAgICAgICAgICAgICAgICAgICAgICAgICAgbWF4KGZpdFtTcGVjaWVzLng9PSJQb2wiXSkrMC41KSwNCiAgICAgICAgICAgICAgICAgICAgICAgICAgICAgbGFiZWxzPWMoImEiLCAiYiIsImIiKSwgY2V4PTEuNSwgYWRqPTEpKQ0KDQptdGV4dChhZGo9LTAuMSwiKEEpIixjZXg9MS41KQ0KZ3JhcGhpY3M6OmxlZ2VuZCgwLjgsMy42LGxlZ2VuZD1leHByZXNzaW9uKGl0YWxpYyhBLm5lbS4pLGl0YWxpYyhPLmFjZS4pLGl0YWxpYyhQLm11bC4pLCJwID4gMC4wNSIsInAiPD0iMC4wNSIpLGJ0eT0nbicsDQogICAgICAgICAgICAgICAgIHBjaD1jKDIxLDIxLDIxLE5BLE5BKSxwdC5iZz1jKCIjYmI1NTY2IiwiI2RkYWEzMyIsIiMwMDQ0ODgiLE5BLE5BKSxsdHk9YyhOQSxOQSxOQSw1LDEpLA0KICAgICAgICAgICAgICAgICBzZWcubGVuPTAuNSxjZXg9MS41LHguaW50ZXJzcD0wLjEseS5pbnRlcnNwPTAuNikNCg0KDQplZmYuSG8uQWdlPC1lZmZlY3QoIkFnZV90IixsbW0uSG8uYi5maW4sIHBhcnRpYWwucmVzaWR1YWxzPVQpDQpwbG90LmRhdGEuQWdlLmFsbDwtZWZmLkhvLkFnZSRkYXRhDQpwbG90LmRhdGEuQWdlLmZpdDwtd2l0aChlZmYuSG8uQWdlLGNiaW5kKHgsZml0LGxvd2VyLHVwcGVyKSkNCg0KcGxvdCgzLHR5cGU9Im4iLHhsaW09YygtMiwyLjUpLHlsaW09YygtMywzLjIpLHhheHQ9J24nLGFubj1GLHlheHQ9J24nKQ0KYXhpcyhzaWRlPTIsbHdkPTIsY2V4LmF4aXM9MikNCmF4aXMoc2lkZT0xLGx3ZD0yLGNleC5heGlzPTIsYXQ9YygtMS4xNjU5NiwwLjE1OTEzOSwxLjAzNzEyNCwxLjczNjAyNyksbGFiZWxzID0gYyg1MCwxNTAsMjUwLDM1MCkpDQpib3gobHdkPTIpDQp0aXRsZSh4bGFiPWMoIlBPUF9BR0UgKHllYXIpIikseWxhYj1leHByZXNzaW9uKGl0YWxpYyhIKVtvXSksbGluZT0yLjUsY2V4LmxhYj0yKQ0Kd2l0aChwbG90LmRhdGEuQWdlLmZpdCxwb2x5Z29uKHg9YyhBZ2VfdCwgcmV2KEFnZV90KSkseT1jKGxvd2VyLCByZXYodXBwZXIpKSwNCiAgICAgICAgICAgICAgICAgICAgICAgICAgICAgICBjb2w9YygiI2RjZGNkYyIpLCBib3JkZXI9TkEpKSANCg0Kd2l0aChHZW5EaXZfYWxsW0dlbkRpdl9hbGwkU3BlY2llcy54PT0iQW5lIixdLCBwb2ludHMoeD1qaXR0ZXIoQWdlX3QpLHk9SG9fdCxwY2g9MjEsY2V4PTIsbHdkPTIsDQogICAgICAgICAgICAgICAgICAgICAgICAgICAgICAgICAgICAgICAgICAgICAgICAgICAgICBiZz1jKCIjYmI1NTY2IikpKQ0KDQp3aXRoKEdlbkRpdl9hbGxbR2VuRGl2X2FsbCRTcGVjaWVzLng9PSJPeGEiLF0sIHBvaW50cyh4PWppdHRlcihBZ2VfdCkseT1Ib190LHBjaD0yMSxjZXg9Mixsd2Q9MiwgDQogICAgICAgICAgICAgICAgICAgICAgICAgICAgICAgICAgICAgICAgICAgICAgICAgICAgICBiZz1jKCIjZGRhYTMzIikpKQ0Kd2l0aChHZW5EaXZfYWxsW0dlbkRpdl9hbGwkU3BlY2llcy54PT0iUG9sIixdLCBwb2ludHMoeD1qaXR0ZXIoQWdlX3QpLHk9SG9fdCxwY2g9MjEsY2V4PTIsbHdkPTIsIA0KICAgICAgICAgICAgICAgICAgICAgICAgICAgICAgICAgICAgICAgICAgICAgICAgICAgICAgYmc9YygiIzAwNDQ4OCIpKSkNCg0Kd2l0aChwbG90LmRhdGEuQWdlLmZpdCxsaW5lcyhBZ2VfdCxmaXQsY29sPXJnYigwLDAsMCxtYXhDb2xvclZhbHVlID0gMjU1KSxsd2Q9MikpDQoNCm10ZXh0KGFkaj0tMC4xLCIoQikiLGNleD0xLjUpDQoNCmVmZi5GaXMuQWdlPC1lZmZlY3QoIkFnZV90IixsbW0uRmlzLmIuZmluLCBwYXJ0aWFsLnJlc2lkdWFscz1UKQ0KcGxvdC5kYXRhLkFnZS5hbGw8LWVmZi5GaXMuQWdlJGRhdGENCnBsb3QuZGF0YS5BZ2UuZml0PC13aXRoKGVmZi5GaXMuQWdlLGNiaW5kKHgsZml0LGxvd2VyLHVwcGVyKSkNCg0KcGxvdCgzLHR5cGU9Im4iLHhsaW09YygtMiwyLjUpLHlsaW09YygtMywzLjIpLHhheHQ9J24nLGFubj1GLHlheHQ9J24nKQ0KYXhpcyhzaWRlPTIsbHdkPTIsY2V4LmF4aXM9MikNCmF4aXMoc2lkZT0xLGx3ZD0yLGNleC5heGlzPTIsYXQ9YygtMS4xNjU5NiwwLjE1OTEzOSwxLjAzNzEyNCwxLjczNjAyNyksbGFiZWxzID0gYyg1MCwxNTAsMjUwLDM1MCkpDQpib3gobHdkPTIpDQp0aXRsZSh4bGFiPWMoIlBPUF9BR0UgKHllYXIpIikseWxhYj1leHByZXNzaW9uKGl0YWxpYyhGKSksbGluZT0yLjUsY2V4LmxhYj0yKQ0Kd2l0aChwbG90LmRhdGEuQWdlLmZpdCxwb2x5Z29uKHg9YyhBZ2VfdCwgcmV2KEFnZV90KSkseT1jKGxvd2VyLCByZXYodXBwZXIpKSwNCiAgICAgICAgICAgICAgICAgICAgICAgICAgICAgICBjb2w9YygiI2RjZGNkYyIpLCBib3JkZXI9TkEpKSANCg0Kd2l0aChHZW5EaXZfYWxsW0dlbkRpdl9hbGwkU3BlY2llcy54PT0iQW5lIixdLCBwb2ludHMoeD1qaXR0ZXIoQWdlX3QpLHk9RmlzX3QscGNoPTIxLGNleD0yLGx3ZD0yLA0KICAgICAgICAgICAgICAgICAgICAgICAgICAgICAgICAgICAgICAgICAgICAgICAgICAgICAgYmc9YygiI2JiNTU2NiIpKSkNCg0Kd2l0aChHZW5EaXZfYWxsW0dlbkRpdl9hbGwkU3BlY2llcy54PT0iT3hhIixdLCBwb2ludHMoeD1qaXR0ZXIoQWdlX3QpLHk9RmlzX3QscGNoPTIxLGNleD0yLGx3ZD0yLCANCiAgICAgICAgICAgICAgICAgICAgICAgICAgICAgICAgICAgICAgICAgICAgICAgICAgICAgIGJnPWMoIiNkZGFhMzMiKSkpDQp3aXRoKEdlbkRpdl9hbGxbR2VuRGl2X2FsbCRTcGVjaWVzLng9PSJQb2wiLF0sIHBvaW50cyh4PWppdHRlcihBZ2VfdCkseT1GaXNfdCxwY2g9MjEsY2V4PTIsbHdkPTIsIA0KICAgICAgICAgICAgICAgICAgICAgICAgICAgICAgICAgICAgICAgICAgICAgICAgICAgICAgYmc9YygiIzAwNDQ4OCIpKSkNCg0Kd2l0aChwbG90LmRhdGEuQWdlLmZpdCxsaW5lcyhBZ2VfdCxmaXQsY29sPXJnYigwLDAsMCxtYXhDb2xvclZhbHVlID0gMjU1KSxsd2Q9MikpDQptdGV4dChhZGo9LTAuMSwiKEMpIixjZXg9MS41KQ0KDQogICAgICAgICAgIA0KYGBgDQoNCmBgYHtyfQ0KIyNGaWd1cmVfNA0KbGlicmFyeShwdGlucG9seSkNCmxpYnJhcnkocGxvdGx5KQ0KbiA8LSAxMDAgI251bWJlciBvZiBncmlkIHBvaW50cyB0byBiZSB1c2VkIGFsb25nIGVhY2ggYXhpcw0KZi5heCA8LSBsaXN0KGZhbWlseT0iQXJpYWwiLCBzaXplPTQ4KSAjZm9udCBmb3IgdGljayBsYWJlbHMgLS0+IHVuaXQgdW5jbGVhciAtLT4gbXVzdCBiZSBsYXJnZXIgd2hlbiBmaWd1cmUgc2l6ZSBpbiBweCBpcyBsYXJnZXINCmYubGFiIDwtIGxpc3QoZmFtaWx5PSJBcmlhbCIsIHNpemU9NTYpICNmb250IGZvciBheGVzIGxhYmVscw0KZi5hbm4gPC0gbGlzdChmYW1pbHk9IkFyaWFsIiwgc2l6ZT00OCwgY29sb3I9InN0ZWVsYmx1ZSIpDQoNCmNyIDwtIGNvbG9yUmFtcChjb2xvcnM9YygiIzM2NEI5QSIsICIjNEE3QkI3IiwgIiM2RUE2Q0QiLCAiIzk4Q0FFMSIsICIjQzJFNEVGIiwgIiNFQUVDQ0MiLCAiI0ZFREE4QiIsICIjRkRCMzY2IiwNCiAgICAgICAgICAgICAgICAgICAgICAgICAiI0Y2N0U0QiIsICIjREQzRDJEIiwgIiNBNTAwMjYiKSkgIy0tPiBjb2xvciBzY2FsZSAic3Vuc2V0IiBkZXNpZ25lZCBieSBQYXVsIFRvbCBmb3IgY29sb3JibGluZCBwZW9wbGU6IGh0dHBzOi8vcGVyc29uYWwuc3Jvbi5ubC9+cGF1bHQvDQoNCiMjIyMjIyMjIyMjIyNPeGEjIyMjIyMjIyMjIyMjIyMjIw0KDQojIyMjIyMjIEhlIyMjIyMjIyMjIyMjIw0KdGVzdDwtYXMuZGF0YS5mcmFtZShtb2RlbC5tYXRyaXgobG1tLkhlLm94YSkpDQoNCnggPC0gd2l0aCh0ZXN0LCBzZXEobWluKEFnZV90KSwgbWF4KEFnZV90KSwgbGVuZ3RoLm91dD1uKSkNCnkgPC0gd2l0aCh0ZXN0LCBzZXEobWluKElGTV90cmFuc2Zvcm1lZCksIG1heChJRk1fdHJhbnNmb3JtZWQpLCBsZW5ndGgub3V0PW4pKQ0KeiA8LSBtYXRyaXgobnJvdz1uLCBuY29sPW4pICNjb250b3VyIGludGVycHJldHMgdGhlIHogbWF0cml4IGFzIGEgdGFibGUgb2YgZih4W2ldLCB5W2pdKSB2YWx1ZXMsIHNvIHRoYXQgdGhlIHggYXhpcyBjb3JyZXNwb25kcyB0byByb3cgbnVtYmVyIGFuZCB0aGUgeSBheGlzIHRvIGNvbHVtbiBudW1iZXIsIHdpdGggY29sdW1uIDEgYXQgdGhlIGJvdHRvbSwgaS5lLiBhIDkwIGRlZ3JlZSBjb3VudGVyLWNsb2Nrd2lzZSByb3RhdGlvbiBvZiB0aGUgY29udmVudGlvbmFsIHRleHR1YWwgbGF5b3V0Lg0KbmV3ZGF0MSA8LSBkYXRhLmZyYW1lKFBvcFNpemVfdHJhbnNmb3JtZWQ9cmVwKG1lZGlhbih0ZXN0JFBvcFNpemVfdHJhbnNmb3JtZWQpLG4pLA0KICAgICAgICAgICAgICAgICAgICAgIElGTV90cmFuc2Zvcm1lZD15KQ0KZm9yKGkgaW4gMTpuKSB7DQogIG5ld2RhdDIgPC0gbmV3ZGF0MQ0KICBuZXdkYXQyJEFnZV90IDwtIHJlcCh4W2ldLG4pDQogIHpbaSxdIDwtIHByZWRpY3QobG1tLkhlLm94YSwgbmV3ZGF0YT1uZXdkYXQyLCBsZXZlbD0wKSAjZWFjaCByb3cgYXMgYSBjb25zdGFudCB4IHZhbHVlLCBidXQgYSBjaGFuZ2luZyB5IHZhbHVlDQp9DQoNCmNsb3VkIDwtIHRlc3RbLGMoIkFnZV90IiwiSUZNX3RyYW5zZm9ybWVkIildICN4IGFuZCB5IGNvb3JpZG5hdGVzIG9mIGEgcG9pbnQgY2xvdWQNCmh1bGwgPC0gYXMubWF0cml4KGNsb3VkW2NodWxsKGNsb3VkKSxdKSAjY29vcmRpbmF0ZXMgb2YgdGhlIGNvbnZleCBodWxsIHZlcnRpY2VzIChjaHVsbCByZXR1cm5zIHRoZSBpbmRpY2VzIG9mIHRoZSB2ZXJ0ZXggcG9pbnRzKQ0KY2hlY2sgPC0gYXMubWF0cml4KGRhdGEuZnJhbWUoeD1yZXAoeCwgZWFjaD1uKSwgeT1yZXAoeSwgdGltZXM9bikpKSAgI1AtYnktMiBtYXRyaXggd2l0aCBwb2ludHMgdG8gYmUgY2hlY2tlZA0KDQpwb2ludC54PC1jaGVja1ssMV0NCnBvaW50Lnk8LWNoZWNrWywyXQ0KeF9hcnJheTwtaHVsbFssMV0NCnlfYXJyYXk8LWh1bGxbLDJdDQp3aXRoaW48LXNwOjpwb2ludC5pbi5wb2x5Z29uKHBvaW50LngscG9pbnQueSx4X2FycmF5LHlfYXJyYXkpIyMgMDogcG9pbnQgaXMgb3V0IG9mIHRoZSBwb2x5Z29uLCAxOiBwb2ludCBpcyBpbiB0aGUgcG9seWdvbiwgMjogcG9pbnQgaXMgb24gdGhlIGxpbmUNCnVuaXF1ZSh3aXRoaW4pDQojLS0+IG5vIGVycm9yIGNvZGVzDQp3aXRoaW4gPC0gaWZlbHNlKHdpdGhpbj49MSwgVFJVRSwgRkFMU0UpDQpwbG90KGNoZWNrWywxXSwgY2hlY2tbLDJdLCBwY2g9MTYsIGNvbD1pZmVsc2Uod2l0aGluLCJyZWQiLCAid2hpdGUiKSkNCndpdGhpbiA8LSBtYXRyaXgod2l0aGluLCBucm93PW4sIG5jb2w9biwgYnlyb3c9VFJVRSkNCnpbIXdpdGhpbl0gPC0gTkEgI3NldCBhbGwgei12YWx1ZXMgb3V0c2lkZSB0aGUgY29udmV4IGh1bGwgdG8gTkENCg0KdHogPC0gdCh6KSAjYmVjYXVzZSBwbG90bHkgaW50ZXJwcmV0cyB6IGFzIHQoeik7IGFsdGVybmF0aXZlbHksIHRoZSBhcmd1bWVudCB0cmFuc3Bvc2UgY2FuIGJlIHNldCB0byBUUlVFIGluIHBsb3RfbHkoKQ0KZmlnNGEgPC0gcGxvdF9seSh4PXgsIHk9eSwgej10eiwgdHlwZT0iY29udG91ciIsDQogICAgICAgICAgICAgICAgIGNvbG9ycz1jciwNCiAgICAgICAgICAgICAgICAgYXV0b2NvbnRvdXI9RiwgY29udG91cnM9bGlzdChzdGFydD1taW4oeixuYS5ybT1UKSxlbmQ9bWF4KHosbmEucm09VCksc2l6ZT0wLjEpLA0KICAgICAgICAgICAgICAgICB3aWR0aD0xMzMwLCBoZWlnaHQ9MTMzMC0xMzMwLzYpICAgI3NldCBwbG90IHdpZHRoIGFuZCBoZWlnaHQgaW4gcHgNCmZpZzRhIDwtIGZpZzRhICU+JSBjb2xvcmJhcihsZW49MSwgdGl0bGU9IjxpPkg8L2k+PHN1Yj5lIiwgdGl0bGVmb250PWYubGFiLCB0aWNrZm9udD1mLmF4LCB0aWNrMD0tMS41LCBkdGljaz0wLjUpIyBleHByZXNzaW9uKERbZXN0XSkgZG9lcyBub3Qgd29yay4uLg0KZmlnNGEgPC0gZmlnNGEgJT4lIGFkZF9tYXJrZXJzKHg9Y2xvdWQkQWdlX3QsIHk9Y2xvdWQkSUZNX3RyYW5zZm9ybWVkLCBpbmhlcml0PUZBTFNFLCBzaG93bGVnZW5kPUZBTFNFLA0KICAgICAgICAgICAgICAgICAgICAgICAgICAgICAgIG1hcmtlcj1saXN0KHN5bWJvbD0iY2lyY2xlLW9wZW4iLCBjb2xvcj0iYmxhY2siLCBzaXplPTE2KSkNCmZpZzRhIDwtIGZpZzRhICU+JSBsYXlvdXQoeGF4aXM9bGlzdCh0aXRsZT0iUE9QX0FHRSIsIHRpdGxlZm9udD1mLmxhYiwgdGlja2ZvbnQ9Zi5heCwgc2hvd2dyaWQ9RkFMU0UsIHplcm9saW5lPUZBTFNFLA0KICAgICAgICAgICAgICAgICAgICAgICAgICAgICAgICAgICAgIHNob3dsaW5lPVRSVUUsIHRpY2tsZW49MTUsIHRpY2t3aWR0aD0zLCBsaW5ld2lkdGg9MyksICAgICAgICAgICAjYWRkIGF4aXMgbGFiZWxzDQogICAgICAgICAgICAgICAgICAgICAgICAgIHlheGlzPWxpc3QodGl0bGU9IlNQQV9DT04iLCB0aXRsZWZvbnQ9Zi5sYWIsIHRpY2tmb250PWYuYXgsIHNob3dncmlkPUZBTFNFLCB6ZXJvbGluZT1GQUxTRSwNCiAgICAgICAgICAgICAgICAgICAgICAgICAgICAgICAgICAgICBzaG93bGluZT1UUlVFLCB0aWNrbGVuPTE1LCB0aWNrd2lkdGg9MywgbGluZXdpZHRoPTMpLA0KICAgICAgICAgICAgICAgICAgICAgICAgICBtYXJnaW49bGlzdChsPTYwLCByPTgwLCB0PTIwLCBiPTYwKSkgICAgICAgICAgICAgICAgICAgICAgICAgICANCmZpZzRhDQoNCiMjIyMjIEhvIyMjIw0KdGVzdDwtYXMuZGF0YS5mcmFtZShtb2RlbC5tYXRyaXgobG1tLkhvLm94YSkpDQp4IDwtIHdpdGgodGVzdCwgc2VxKG1pbihBZ2VfdCksIG1heChBZ2VfdCksIGxlbmd0aC5vdXQ9bikpDQp5IDwtIHdpdGgodGVzdCwgc2VxKG1pbihQb3BTaXplX3RyYW5zZm9ybWVkKSwgbWF4KFBvcFNpemVfdHJhbnNmb3JtZWQpLCBsZW5ndGgub3V0PW4pKQ0KeiA8LSBtYXRyaXgobnJvdz1uLCBuY29sPW4pICNjb250b3VyIGludGVycHJldHMgdGhlIHogbWF0cml4IGFzIGEgdGFibGUgb2YgZih4W2ldLCB5W2pdKSB2YWx1ZXMsIHNvIHRoYXQgdGhlIHggYXhpcyBjb3JyZXNwb25kcyB0byByb3cgbnVtYmVyIGFuZCB0aGUgeSBheGlzIHRvIGNvbHVtbiBudW1iZXIsIHdpdGggY29sdW1uIDEgYXQgdGhlIGJvdHRvbSwgaS5lLiBhIDkwIGRlZ3JlZSBjb3VudGVyLWNsb2Nrd2lzZSByb3RhdGlvbiBvZiB0aGUgY29udmVudGlvbmFsIHRleHR1YWwgbGF5b3V0Lg0KbmV3ZGF0MSA8LSBkYXRhLmZyYW1lKElGTV90cmFuc2Zvcm1lZD1yZXAobWVkaWFuKHRlc3QkSUZNX3RyYW5zZm9ybWVkKSxuKSwNCiAgICAgICAgICAgICAgICAgICAgICBQb3BTaXplX3RyYW5zZm9ybWVkPXkpDQpmb3IoaSBpbiAxOm4pIHsNCiAgbmV3ZGF0MiA8LSBuZXdkYXQxDQogIG5ld2RhdDIkQWdlX3QgPC0gcmVwKHhbaV0sbikNCiAgeltpLF0gPC0gcHJlZGljdChsbW0uSG8ub3hhLCBuZXdkYXRhPW5ld2RhdDIsIGxldmVsPTApICNlYWNoIHJvdyBhcyBhIGNvbnN0YW50IHggdmFsdWUsIGJ1dCBhIGNoYW5naW5nIHkgdmFsdWUNCn0NCg0KY2xvdWQgPC0gdGVzdFssYygiQWdlX3QiLCJQb3BTaXplX3RyYW5zZm9ybWVkIildICN4IGFuZCB5IGNvb3JpZG5hdGVzIG9mIGEgcG9pbnQgY2xvdWQNCmh1bGwgPC0gYXMubWF0cml4KGNsb3VkW2NodWxsKGNsb3VkKSxdKSAjY29vcmRpbmF0ZXMgb2YgdGhlIGNvbnZleCBodWxsIHZlcnRpY2VzIChjaHVsbCByZXR1cm5zIHRoZSBpbmRpY2VzIG9mIHRoZSB2ZXJ0ZXggcG9pbnRzKQ0KY2hlY2sgPC0gYXMubWF0cml4KGRhdGEuZnJhbWUoeD1yZXAoeCwgZWFjaD1uKSwgeT1yZXAoeSwgdGltZXM9bikpKSAgI1AtYnktMiBtYXRyaXggd2l0aCBwb2ludHMgdG8gYmUgY2hlY2tlZA0KDQpwb2ludC54PC1jaGVja1ssMV0NCnBvaW50Lnk8LWNoZWNrWywyXQ0KeF9hcnJheTwtaHVsbFssMV0NCnlfYXJyYXk8LWh1bGxbLDJdDQp3aXRoaW48LXNwOjpwb2ludC5pbi5wb2x5Z29uKHBvaW50LngscG9pbnQueSx4X2FycmF5LHlfYXJyYXkpIyMgMDogcG9pbnQgaXMgb3V0IG9mIHRoZSBwb2x5Z29uLCAxOiBwb2ludCBpcyBpbiB0aGUgcG9seWdvbiwgMjogcG9pbnQgaXMgb24gdGhlIGxpbmUNCnVuaXF1ZSh3aXRoaW4pDQojLS0+IG5vIGVycm9yIGNvZGVzDQp3aXRoaW4gPC0gaWZlbHNlKHdpdGhpbj49MSwgVFJVRSwgRkFMU0UpDQpwbG90KGNoZWNrWywxXSwgY2hlY2tbLDJdLCBwY2g9MTYsIGNvbD1pZmVsc2Uod2l0aGluLCJyZWQiLCAid2hpdGUiKSkNCndpdGhpbiA8LSBtYXRyaXgod2l0aGluLCBucm93PW4sIG5jb2w9biwgYnlyb3c9VFJVRSkNCnpbIXdpdGhpbl0gPC0gTkEgI3NldCBhbGwgei12YWx1ZXMgb3V0c2lkZSB0aGUgY29udmV4IGh1bGwgdG8gTkENCg0KdHogPC0gdCh6KSAjYmVjYXVzZSBwbG90bHkgaW50ZXJwcmV0cyB6IGFzIHQoeik7IGFsdGVybmF0aXZlbHksIHRoZSBhcmd1bWVudCB0cmFuc3Bvc2UgY2FuIGJlIHNldCB0byBUUlVFIGluIHBsb3RfbHkoKQ0KZmlnNGIgPC0gcGxvdF9seSh4PXgsIHk9eSwgej10eiwgdHlwZT0iY29udG91ciIsDQogICAgICAgICAgICAgICAgIGNvbG9ycz1jciwNCiAgICAgICAgICAgICAgICAgYXV0b2NvbnRvdXI9RiwgY29udG91cnM9bGlzdChzdGFydD1taW4oeixuYS5ybT1UKSxlbmQ9bWF4KHosbmEucm09VCksc2l6ZT0wLjEpLA0KICAgICAgICAgICAgICAgICB3aWR0aD0xMzMwLCBoZWlnaHQ9MTMzMC0xMzMwLzYpICAgI3NldCBwbG90IHdpZHRoIGFuZCBoZWlnaHQgaW4gcHgNCmZpZzRiIDwtIGZpZzRiICU+JSBjb2xvcmJhcihsZW49MSwgdGl0bGU9IjxpPkg8L2k+PHN1Yj5vIiwgdGl0bGVmb250PWYubGFiLCB0aWNrZm9udD1mLmF4LCB0aWNrMD0tMS41LCBkdGljaz0wLjUpIyBleHByZXNzaW9uKERbZXN0XSkgZG9lcyBub3Qgd29yay4uLg0KZmlnNGIgPC0gZmlnNGIgJT4lIGFkZF9tYXJrZXJzKHg9Y2xvdWQkQWdlX3QsIHk9Y2xvdWQkUG9wU2l6ZV90cmFuc2Zvcm1lZCwgaW5oZXJpdD1GQUxTRSwgc2hvd2xlZ2VuZD1GQUxTRSwNCiAgICAgICAgICAgICAgICAgICAgICAgICAgICAgICBtYXJrZXI9bGlzdChzeW1ib2w9ImNpcmNsZS1vcGVuIiwgY29sb3I9ImJsYWNrIiwgc2l6ZT0xNikpDQpmaWc0YiA8LSBmaWc0YiAlPiUgbGF5b3V0KHhheGlzPWxpc3QodGl0bGU9IlBPUF9BR0UiLCB0aXRsZWZvbnQ9Zi5sYWIsIHRpY2tmb250PWYuYXgsIHNob3dncmlkPUZBTFNFLCB6ZXJvbGluZT1GQUxTRSwNCiAgICAgICAgICAgICAgICAgICAgICAgICAgICAgICAgICAgICBzaG93bGluZT1UUlVFLCB0aWNrbGVuPTE1LCB0aWNrd2lkdGg9MywgbGluZXdpZHRoPTMpLCAgICAgICAgICAgI2FkZCBheGlzIGxhYmVscw0KICAgICAgICAgICAgICAgICAgICAgICAgICB5YXhpcz1saXN0KHRpdGxlPSJQT1BfU0laRSIsIHRpdGxlZm9udD1mLmxhYiwgdGlja2ZvbnQ9Zi5heCwgc2hvd2dyaWQ9RkFMU0UsIHplcm9saW5lPUZBTFNFLA0KICAgICAgICAgICAgICAgICAgICAgICAgICAgICAgICAgICAgIHNob3dsaW5lPVRSVUUsIHRpY2tsZW49MTUsIHRpY2t3aWR0aD0zLCBsaW5ld2lkdGg9MyksDQogICAgICAgICAgICAgICAgICAgICAgICAgIG1hcmdpbj1saXN0KGw9NjAsIHI9ODAsIHQ9MjAsIGI9NjApKSAgICAgICAgICAgICAgICAgICAgICAgICAgICAjc2V0cyB0aGUgcGxvdCBtYXJnaW4gaW4gcHgsIHNlZSBodHRwczovL3Bsb3RseS5jb20vci9yZWZlcmVuY2UvbGF5b3V0LyNsYXlvdXQtYXV0b3NpemUNCmZpZzRiDQojIyMjIyMjIyMjIyMjUG9sIyMjIyMjIyMjIyMjIyMjIyMNCnRlc3Q8LWFzLmRhdGEuZnJhbWUobW9kZWwubWF0cml4KGxtbS5IZS5wb2wpKQ0KDQojIyMjIyMjIyMjIyMjIyMjcG9wc2l6ZSYgSGUjIyMjIyMjIyMjIyMjIyMjIyMjIyMjIyMjDQp4IDwtIHdpdGgodGVzdCwgc2VxKG1pbihBZ2VfdCksIG1heChBZ2VfdCksIGxlbmd0aC5vdXQ9bikpDQp5IDwtIHdpdGgodGVzdCwgc2VxKG1pbihQb3BTaXplX3RyYW5zZm9ybWVkKSwgbWF4KFBvcFNpemVfdHJhbnNmb3JtZWQpLCBsZW5ndGgub3V0PW4pKQ0KeiA8LSBtYXRyaXgobnJvdz1uLCBuY29sPW4pICNjb250b3VyIGludGVycHJldHMgdGhlIHogbWF0cml4IGFzIGEgdGFibGUgb2YgZih4W2ldLCB5W2pdKSB2YWx1ZXMsIHNvIHRoYXQgdGhlIHggYXhpcyBjb3JyZXNwb25kcyB0byByb3cgbnVtYmVyIGFuZCB0aGUgeSBheGlzIHRvIGNvbHVtbiBudW1iZXIsIHdpdGggY29sdW1uIDEgYXQgdGhlIGJvdHRvbSwgaS5lLiBhIDkwIGRlZ3JlZSBjb3VudGVyLWNsb2Nrd2lzZSByb3RhdGlvbiBvZiB0aGUgY29udmVudGlvbmFsIHRleHR1YWwgbGF5b3V0Lg0KbmV3ZGF0MSA8LSBkYXRhLmZyYW1lKElGTV90cmFuc2Zvcm1lZD1yZXAobWVkaWFuKHRlc3QkSUZNX3RyYW5zZm9ybWVkKSxuKSwNCiAgICAgICAgICAgICAgICAgICAgICBQb3BTaXplX3RyYW5zZm9ybWVkPXkpDQpmb3IoaSBpbiAxOm4pIHsNCiAgbmV3ZGF0MiA8LSBuZXdkYXQxDQogIG5ld2RhdDIkQWdlX3QgPC0gcmVwKHhbaV0sbikNCiAgeltpLF0gPC0gcHJlZGljdChsbW0uSGUucG9sLCBuZXdkYXRhPW5ld2RhdDIsIGxldmVsPTApICNlYWNoIHJvdyBhcyBhIGNvbnN0YW50IHggdmFsdWUsIGJ1dCBhIGNoYW5naW5nIHkgdmFsdWUNCn0NCg0KY2xvdWQgPC0gdGVzdFssYygiQWdlX3QiLCJQb3BTaXplX3RyYW5zZm9ybWVkIildICN4IGFuZCB5IGNvb3JpZG5hdGVzIG9mIGEgcG9pbnQgY2xvdWQNCmh1bGwgPC0gYXMubWF0cml4KGNsb3VkW2NodWxsKGNsb3VkKSxdKSAjY29vcmRpbmF0ZXMgb2YgdGhlIGNvbnZleCBodWxsIHZlcnRpY2VzIChjaHVsbCByZXR1cm5zIHRoZSBpbmRpY2VzIG9mIHRoZSB2ZXJ0ZXggcG9pbnRzKQ0KY2hlY2sgPC0gYXMubWF0cml4KGRhdGEuZnJhbWUoeD1yZXAoeCwgZWFjaD1uKSwgeT1yZXAoeSwgdGltZXM9bikpKSAgI1AtYnktMiBtYXRyaXggd2l0aCBwb2ludHMgdG8gYmUgY2hlY2tlZA0KcG9pbnQueDwtY2hlY2tbLDFdDQpwb2ludC55PC1jaGVja1ssMl0NCnhfYXJyYXk8LWh1bGxbLDFdDQp5X2FycmF5PC1odWxsWywyXQ0Kd2l0aGluPC1zcDo6cG9pbnQuaW4ucG9seWdvbihwb2ludC54LHBvaW50LnkseF9hcnJheSx5X2FycmF5KSMjIDA6IHBvaW50IGlzIG91dCBvZiB0aGUgcG9seWdvbiwgMTogcG9pbnQgaXMgaW4gdGhlIHBvbHlnb24sIDI6IHBvaW50IGlzIG9uIHRoZSBsaW5lDQp1bmlxdWUod2l0aGluKQ0KIy0tPiBubyBlcnJvciBjb2Rlcw0Kd2l0aGluIDwtIGlmZWxzZSh3aXRoaW4+PTEsIFRSVUUsIEZBTFNFKQ0KcGxvdChjaGVja1ssMV0sIGNoZWNrWywyXSwgcGNoPTE2LCBjb2w9aWZlbHNlKHdpdGhpbiwicmVkIiwgIndoaXRlIikpDQp3aXRoaW4gPC0gbWF0cml4KHdpdGhpbiwgbnJvdz1uLCBuY29sPW4sIGJ5cm93PVRSVUUpDQp6WyF3aXRoaW5dIDwtIE5BICNzZXQgYWxsIHotdmFsdWVzIG91dHNpZGUgdGhlIGNvbnZleCBodWxsIHRvIE5BDQoNCnR6IDwtIHQoeikgI2JlY2F1c2UgcGxvdGx5IGludGVycHJldHMgeiBhcyB0KHopOyBhbHRlcm5hdGl2ZWx5LCB0aGUgYXJndW1lbnQgdHJhbnNwb3NlIGNhbiBiZSBzZXQgdG8gVFJVRSBpbiBwbG90X2x5KCkNCmZpZzRjIDwtIHBsb3RfbHkoeD14LCB5PXksIHo9dHosIHR5cGU9ImNvbnRvdXIiLA0KICAgICAgICAgICAgICAgICBjb2xvcnM9Y3IsDQogICAgICAgICAgICAgICAgIGF1dG9jb250b3VyPUYsIGNvbnRvdXJzPWxpc3Qoc3RhcnQ9bWluKHosbmEucm09VCksZW5kPW1heCh6LG5hLnJtPVQpLHNpemU9MC4xKSwNCiAgICAgICAgICAgICAgICAgd2lkdGg9MTMzMCwgaGVpZ2h0PTEzMzAtMTMzMC82KSAgICNzZXQgcGxvdCB3aWR0aCBhbmQgaGVpZ2h0IGluIHB4DQpmaWc0YyA8LSBmaWc0YyAlPiUgY29sb3JiYXIobGVuPTEsIHRpdGxlPSI8aT5IPC9pPjxzdWI+ZSIsIHRpdGxlZm9udD1mLmxhYiwgdGlja2ZvbnQ9Zi5heCwgdGljazA9LTEuNSwgZHRpY2s9MC41KSMgZXhwcmVzc2lvbihEW2VzdF0pIGRvZXMgbm90IHdvcmsuLi4NCmZpZzRjIDwtIGZpZzRjICU+JSBhZGRfbWFya2Vycyh4PWNsb3VkJEFnZV90LCB5PWNsb3VkJFBvcFNpemVfdHJhbnNmb3JtZWQsIGluaGVyaXQ9RkFMU0UsIHNob3dsZWdlbmQ9RkFMU0UsDQogICAgICAgICAgICAgICAgICAgICAgICAgICAgICAgbWFya2VyPWxpc3Qoc3ltYm9sPSJjaXJjbGUtb3BlbiIsIGNvbG9yPSJibGFjayIsIHNpemU9MTYpKQ0KZmlnNGMgPC0gZmlnNGMgJT4lIGxheW91dCh4YXhpcz1saXN0KHRpdGxlPSJQT1BfQUdFIiwgdGl0bGVmb250PWYubGFiLCB0aWNrZm9udD1mLmF4LCBzaG93Z3JpZD1GQUxTRSwgemVyb2xpbmU9RkFMU0UsDQogICAgICAgICAgICAgICAgICAgICAgICAgICAgICAgICAgICAgc2hvd2xpbmU9VFJVRSwgdGlja2xlbj0xNSwgdGlja3dpZHRoPTMsIGxpbmV3aWR0aD0zKSwgICAgICAgICAgICNhZGQgYXhpcyBsYWJlbHMNCiAgICAgICAgICAgICAgICAgICAgICAgICAgeWF4aXM9bGlzdCh0aXRsZT0iUE9QX1NJWkUiLCB0aXRsZWZvbnQ9Zi5sYWIsIHRpY2tmb250PWYuYXgsIHNob3dncmlkPUZBTFNFLCB6ZXJvbGluZT1GQUxTRSwNCiAgICAgICAgICAgICAgICAgICAgICAgICAgICAgICAgICAgICBzaG93bGluZT1UUlVFLCB0aWNrbGVuPTE1LCB0aWNrd2lkdGg9MywgbGluZXdpZHRoPTMpLA0KICAgICAgICAgICAgICAgICAgICAgICAgICBtYXJnaW49bGlzdChsPTYwLCByPTgwLCB0PTIwLCBiPTYwKSkgICAgICAgICAgICAgICAgICAgICAgICAgICAgI3NldHMgdGhlIHBsb3QgbWFyZ2luIGluIHB4LCBzZWUgaHR0cHM6Ly9wbG90bHkuY29tL3IvcmVmZXJlbmNlL2xheW91dC8jbGF5b3V0LWF1dG9zaXplDQpmaWc0Yw0KYGBgDQpHZW5ldGljIERpZmZlcmVudGlhdGlvbg0KYGBge3J9DQoNCiMjIyBGaWd1cmVfNQ0KIyMjIyMjIyMjIyMjIyMjIyMjIyMjQUdFX0JBU0UjIw0KcGFyKG1mcm93PWMoMSwzKSxtYXI9Yyg1LDUsMiwyKSkNCiMjIEdzdA0KZWZmLkdzdC5BZ2VfeW91bmdlcjwtZWZmZWN0KCJBZ2VfeW91bmdlcl90IixHc3QuZmluLCBwYXJ0aWFsLnJlc2lkdWFscz1UKQ0KcGxvdC5kYXRhLkFnZS5hbGw8LWVmZi5Hc3QuQWdlX3lvdW5nZXIkZGF0YQ0KcGxvdC5kYXRhLkFnZS5maXQ8LXdpdGgoZWZmLkdzdC5BZ2VfeW91bmdlcixjYmluZCh4LGZpdCxsb3dlcix1cHBlcikpDQoNCnBsb3QoMyx0eXBlPSJuIix4bGltPWMoLTIsMykseWxpbT1jKC0zLDIuNSkseGF4dD0nbicsYW5uPUYseWF4dD0nbicpDQpheGlzKHNpZGU9Mixsd2Q9MixjZXguYXhpcz0xLjUpDQpheGlzKHNpZGU9MSxsd2Q9MixjZXguYXhpcz0xLjUsYXQ9YygtMC42OTI5NjkyLDAuNzkxNjg0OSwxLjYwMTI3NywyLjE4MTU1MSksbGFiZWxzID0gYyg1MCwxNTAsMjUwLDM1MCkpDQpib3gobHdkPTIpDQp0aXRsZSh4bGFiPSJBR0VfQkFTRSAoeWVhcikiLHlsYWI9ZXhwcmVzc2lvbihpdGFsaWMocGFzdGUoIkcnJyIpKVtTVF0pLGxpbmU9MyxjZXgubGFiPTEuNSkNCndpdGgocGxvdC5kYXRhLkFnZS5maXQscG9seWdvbih4PWMoQWdlX3lvdW5nZXJfdCwgcmV2KEFnZV95b3VuZ2VyX3QpKSx5PWMobG93ZXIsIHJldih1cHBlcikpLA0KICAgICAgICAgICAgICAgICAgICAgICAgICAgICAgIGNvbD1jKCIjZGNkY2RjIiksIGJvcmRlcj1OQSkpIA0KDQp3aXRoKGRpc3RbZGlzdCRTcGVjaWVzLng9PSJBbmUiLF0sIHBvaW50cyh4PWppdHRlcihBZ2VfeW91bmdlcl90KSx5PUdzdF90LHBjaD0yMSxjZXg9Mixsd2Q9MS41LA0KICAgICAgICAgICAgICAgICAgICAgICAgICAgICAgICAgICAgICAgICAgYmc9YygiI2JiNTU2NiIpKSkNCg0Kd2l0aChkaXN0W2Rpc3QkU3BlY2llcy54PT0iT3hhIixdLCBwb2ludHMoeD1qaXR0ZXIoQWdlX3lvdW5nZXJfdCkseT1Hc3RfdCxwY2g9MjEsY2V4PTIsbHdkPTEuNSwgDQogICAgICAgICAgICAgICAgICAgICAgICAgICAgICAgICAgICAgICAgICBiZz1jKCIjZGRhYTMzIikpKQ0Kd2l0aChkaXN0W2Rpc3QkU3BlY2llcy54PT0iUG9sIixdLCBwb2ludHMoeD1qaXR0ZXIoQWdlX3lvdW5nZXJfdCkseT1Hc3RfdCxwY2g9MjEsY2V4PTIsbHdkPTEuNSwgDQogICAgICAgICAgICAgICAgICAgICAgICAgICAgICAgICAgICAgICAgICBiZz1jKCIjMDA0NDg4IikpKQ0KDQp3aXRoKHBsb3QuZGF0YS5BZ2UuZml0LGxpbmVzKEFnZV95b3VuZ2VyX3QsZml0LGNvbD1yZ2IoMCwwLDAsbWF4Q29sb3JWYWx1ZSA9IDI1NSksbHdkPTIsbHR5PTUpKQ0KbXRleHQoYWRqPS0wLjIsIihBKSIsY2V4PTEuNSkNCiMjRHBzDQojIyMjIyMjIyMjIyMjIyMjIw0KZWZmLkRwcy5BZ2VfeW91bmdlcjwtZWZmZWN0KCJBZ2VfeW91bmdlcl90IixEcHMuZmluLCBwYXJ0aWFsLnJlc2lkdWFscz1UKQ0KcGxvdC5kYXRhLkFnZS5hbGw8LWVmZi5EcHMuQWdlX3lvdW5nZXIkZGF0YQ0KcGxvdC5kYXRhLkFnZS5maXQ8LXdpdGgoZWZmLkRwcy5BZ2VfeW91bmdlcixjYmluZCh4LGZpdCxsb3dlcix1cHBlcikpDQoNCnBsb3QoMyx0eXBlPSJuIix4bGltPWMoLTIsMykseWxpbT1jKC0zLDIuNSkseGF4dD0nbicsYW5uPUYseWF4dD0nbicpDQpheGlzKHNpZGU9Mixsd2Q9MixjZXguYXhpcz0xLjUpDQpheGlzKHNpZGU9MSxsd2Q9MixjZXguYXhpcz0xLjUsYXQ9YygtMC42OTI5NjkyLDAuNzkxNjg0OSwxLjYwMTI3NywyLjE4MTU1MSksbGFiZWxzID0gYyg1MCwxNTAsMjUwLDM1MCkpDQpib3gobHdkPTIpDQp0aXRsZSh4bGFiPSJBR0VfQkFTRSAoeWVhcikiLHlsYWI9ZXhwcmVzc2lvbihpdGFsaWMocGFzdGUoIkQiKSlbUFNdKSxsaW5lPTMsY2V4LmxhYj0xLjUpDQp3aXRoKHBsb3QuZGF0YS5BZ2UuZml0LHBvbHlnb24oeD1jKEFnZV95b3VuZ2VyX3QsIHJldihBZ2VfeW91bmdlcl90KSkseT1jKGxvd2VyLCByZXYodXBwZXIpKSwNCiAgICAgICAgICAgICAgICAgICAgICAgICAgICAgICBjb2w9YygiI2RjZGNkYyIpLCBib3JkZXI9TkEpKSANCg0Kd2l0aChkaXN0W2Rpc3QkU3BlY2llcy54PT0iQW5lIixdLCBwb2ludHMoeD1qaXR0ZXIoQWdlX3lvdW5nZXJfdCkseT1EcHNfdCxwY2g9MjEsY2V4PTIsbHdkPTEuNSwNCiAgICAgICAgICAgICAgICAgICAgICAgICAgICAgICAgICAgICAgICAgIGJnPWMoIiNiYjU1NjYiKSkpDQoNCndpdGgoZGlzdFtkaXN0JFNwZWNpZXMueD09Ik94YSIsXSwgcG9pbnRzKHg9aml0dGVyKEFnZV95b3VuZ2VyX3QpLHk9RHBzX3QscGNoPTIxLGNleD0yLGx3ZD0xLjUsIA0KICAgICAgICAgICAgICAgICAgICAgICAgICAgICAgICAgICAgICAgICAgYmc9YygiI2RkYWEzMyIpKSkNCndpdGgoZGlzdFtkaXN0JFNwZWNpZXMueD09IlBvbCIsXSwgcG9pbnRzKHg9aml0dGVyKEFnZV95b3VuZ2VyX3QpLHk9RHBzX3QscGNoPTIxLGNleD0yLGx3ZD0xLjUsIA0KICAgICAgICAgICAgICAgICAgICAgICAgICAgICAgICAgICAgICAgICAgYmc9YygiIzAwNDQ4OCIpKSkNCg0Kd2l0aChwbG90LmRhdGEuQWdlLmZpdCxsaW5lcyhBZ2VfeW91bmdlcl90LGZpdCxjb2w9cmdiKDAsMCwwLG1heENvbG9yVmFsdWUgPSAyNTUpLGx3ZD0yKSkNCm10ZXh0KGFkaj0tMC4yLCIoQikiLGNleD0xLjUpDQojIyMgY0dEIyMjIyMjIyMjIw0KZWZmZWN0LmFnZWJhc2U8LWVmZmVjdCgiQWdlX3lvdW5nZXJfdDpTcGVjaWVzLngiLGMuR0QueS5maW4scGFydGlhbC5yZXNpZHVhbHM9VCkNCnBsb3QuZGF0YS5hbGw8LWVmZmVjdC5hZ2ViYXNlJGRhdGENCnBsb3QuZGF0YS5maXQ8LXdpdGgoZWZmZWN0LmFnZWJhc2UsY2JpbmQoeCxmaXQsbG93ZXIsdXBwZXIpKQ0KcGxvdCgzLHR5cGU9Im4iLHhsaW09YygtMiwzKSx5bGltPWMoLTMsMykseGF4dD0nbicsYW5uPUYseWF4dD0nbicpDQpheGlzKHNpZGU9Mixsd2Q9MixjZXguYXhpcz0xLjUpDQpheGlzKHNpZGU9MSxsd2Q9MixjZXguYXhpcz0xLjUsYXQ9YygtMC43MDc1NDczLDAuODA5NDE4OCwxLjYwNDU1NSwyLjE2MjU4NCksbGFiZWxzID0gYyg1MCwxNTAsMjUwLDM1MCkpDQpib3gobHdkPTIpDQp0aXRsZSh4bGFiPWMoIkFHRV9CQVNFICh5ZWFyKSIpLHlsYWI9ZXhwcmVzc2lvbihpdGFsaWMoYykqIkdEIiksbGluZT0zLGNleC5sYWI9MS41KQ0KDQp3aXRoKHBsb3QuZGF0YS5maXRbcGxvdC5kYXRhLmZpdCRTcGVjaWVzLng9PSJBbmUiLF0scG9seWdvbih4PWMoQWdlX3lvdW5nZXJfdCwgcmV2KEFnZV95b3VuZ2VyX3QpKSx5PWMobG93ZXIsIHJldih1cHBlcikpLA0KICAgICAgICAgICAgICAgICAgICAgICAgICAgICAgICAgICAgICAgICAgICAgICAgICAgICAgICAgICAgY29sPXNjYWxlczo6YWxwaGEoIiNiYjU1NjYiLDAuMyksIGJvcmRlcj1OQSkpDQoNCndpdGgocGxvdC5kYXRhLmZpdFtwbG90LmRhdGEuZml0JFNwZWNpZXMueD09IlBvbCIsXSxwb2x5Z29uKHg9YyhBZ2VfeW91bmdlcl90LCByZXYoQWdlX3lvdW5nZXJfdCkpLHk9Yyhsb3dlciwgcmV2KHVwcGVyKSksDQogICAgICAgICAgICAgICAgICAgICAgICAgICAgICAgICAgICAgICAgICAgICAgICAgICAgICAgICAgICBjb2w9c2NhbGVzOjphbHBoYSgiIzAwNDQ4OCIsMC4zKSwgYm9yZGVyPU5BKSkNCg0KDQp3aXRoKHBsb3QuZGF0YS5hbGxbcGxvdC5kYXRhLmFsbCRTcGVjaWVzLng9PSJBbmUiLF0sIHBvaW50cyh4PWppdHRlcihBZ2VfeW91bmdlcl90KSx5PUNvbi5EX3QsY2V4PTIscGNoPTIxLGx3ZD0xLjUsDQogICAgICAgICAgICAgICAgICAgICAgICAgICAgICAgICAgICAgICAgICAgICAgICAgICAgICAgICAgICBiZz1jKCIjYmI1NTY2IikpKQ0KDQp3aXRoKHBsb3QuZGF0YS5hbGxbcGxvdC5kYXRhLmFsbCRTcGVjaWVzLng9PSJPeGEiLF0sIHBvaW50cyh4PWppdHRlcihBZ2VfeW91bmdlcl90KSx5PUNvbi5EX3QsY2V4PTIscGNoPTIxLGx3ZD0xLjUsIA0KICAgICAgICAgICAgICAgICAgICAgICAgICAgICAgICAgICAgICAgICAgICAgICAgICAgICAgICAgICAgYmc9YygiI2RkYWEzMyIpKSkNCndpdGgocGxvdC5kYXRhLmFsbFtwbG90LmRhdGEuYWxsJFNwZWNpZXMueD09IlBvbCIsXSwgcG9pbnRzKHg9aml0dGVyKEFnZV95b3VuZ2VyX3QpLHk9Q29uLkRfdCxjZXg9MixwY2g9MjEsbHdkPTEuNSwgDQogICAgICAgICAgICAgICAgICAgICAgICAgICAgICAgICAgICAgICAgICAgICAgICAgICAgICAgICAgICBiZz1jKCIjMDA0NDg4IikpKQ0KDQp3aXRoKHBsb3QuZGF0YS5maXRbcGxvdC5kYXRhLmZpdCRTcGVjaWVzLng9PSJBbmUiLF0sbGluZXMoQWdlX3lvdW5nZXJfdCxmaXQsY29sPWMoIiNiYjU1NjYiKSxsd2Q9MikpDQp3aXRoKHBsb3QuZGF0YS5maXRbcGxvdC5kYXRhLmZpdCRTcGVjaWVzLng9PSJPeGEiLF0sbGluZXMoQWdlX3lvdW5nZXJfdCxmaXQsY29sPWMoIiNkZGFhMzMiKSxsd2Q9MixsdHk9NSkpDQp3aXRoKHBsb3QuZGF0YS5maXRbcGxvdC5kYXRhLmZpdCRTcGVjaWVzLng9PSJQb2wiLF0sbGluZXMoQWdlX3lvdW5nZXJfdCxmaXQsY29sPWMoIiMwMDQ0ODgiKSxsd2Q9MikpDQoNCndpdGgocGxvdC5kYXRhLmZpdCwgdGV4dCgyLjUsIHk9YyhtaW4oZml0W1NwZWNpZXMueD09IkFuZSJdKSwNCiAgICAgICAgICAgICAgICAgICAgICAgICAgICAgICAgICBtaW4oZml0W1NwZWNpZXMueD09Ik94YSJdKSwNCiAgICAgICAgICAgICAgICAgICAgICAgICAgICAgICAgICBtYXgoZml0W1NwZWNpZXMueD09IlBvbCJdKSswLjUpLA0KICAgICAgICAgICAgICAgICAgICAgICAgIGxhYmVscz1jKCJhIiwgImFiIiwiYiIpLCBjZXg9MS41LCBhZGo9MSkpDQptdGV4dChhZGo9LTAuMiwiKEMpIixjZXg9MS41KQ0KZ3JhcGhpY3M6OmxlZ2VuZCgxLjQsMy4yLGxlZ2VuZD1leHByZXNzaW9uKGl0YWxpYyhBLm5lbS4pLGl0YWxpYyhPLmFjZS4pLGl0YWxpYyhQLm11bC4pLCJwID4gMC4wNSIsInAiPD0iMC4wNSIpLGJ0eT0nbicsDQogICAgICAgICAgICAgICAgIHBjaD1jKDIxLDIxLDIxLE5BLE5BKSxwdC5iZz1jKCIjYmI1NTY2IiwiI2RkYWEzMyIsIiMwMDQ0ODgiLE5BLE5BKSxsdHk9YyhOQSxOQSxOQSw1LDEpLA0KICAgICAgICAgICAgICAgICBzZWcubGVuPTAuNSxjZXg9MS4zLHguaW50ZXJzcD0wLjEseS5pbnRlcnNwPTAuNikNCg0KDQpgYGANCmludGVyYWN0aW9uIGRpZmZlcmVudGlhdGlvbg0KYGBge3J9DQojI3Zpc3VhbGlzYXRpb24gb2YgaW50ZXJhY3Rpb24jIw0KIyNBbmUjIyMjIyMjIw0KbiA8LSAxMDAgI251bWJlciBvZiBncmlkIHBvaW50cyB0byBiZSB1c2VkIGFsb25nIGVhY2ggYXhpcw0KZi5heCA8LSBsaXN0KGZhbWlseT0iQXJpYWwiLCBzaXplPTQ4KSAjZm9udCBmb3IgdGljayBsYWJlbHMgLS0+IHVuaXQgdW5jbGVhciAtLT4gbXVzdCBiZSBsYXJnZXIgd2hlbiBmaWd1cmUgc2l6ZSBpbiBweCBpcyBsYXJnZXINCmYubGFiIDwtIGxpc3QoZmFtaWx5PSJBcmlhbCIsIHNpemU9NTYpICNmb250IGZvciBheGVzIGxhYmVscw0KZi5hbm4gPC0gbGlzdChmYW1pbHk9IkFyaWFsIiwgc2l6ZT00OCwgY29sb3I9InN0ZWVsYmx1ZSIpDQoNCmNyIDwtIGNvbG9yUmFtcChjb2xvcnM9YygiIzM2NEI5QSIsICIjNEE3QkI3IiwgIiM2RUE2Q0QiLCAiIzk4Q0FFMSIsICIjQzJFNEVGIiwgIiNFQUVDQ0MiLCAiI0ZFREE4QiIsICIjRkRCMzY2IiwNCiAgICAgICAgICAgICAgICAgICAgICAgICAiI0Y2N0U0QiIsICIjREQzRDJEIiwgIiNBNTAwMjYiKSkgIy0tPiBjb2xvciBzY2FsZSAic3Vuc2V0IiBkZXNpZ25lZCBieSBQYXVsIFRvbCBmb3IgY29sb3JibGluZCBwZW9wbGU6IGh0dHBzOi8vcGVyc29uYWwuc3Jvbi5ubC9+cGF1bHQvDQoNCiMjIyMjIyMjIyMjIyMjIyMjIEdzdH4gR2VvRGlzdCAqIEFnZV9CYXNlIyMjIyMjIyMjIyMjIyMNCnRlc3Q8LWFzLmRhdGEuZnJhbWUobW9kZWwubWF0cml4KEdzdC5hbmUpKQ0KDQp4IDwtIHdpdGgodGVzdCwgc2VxKG1pbihBZ2VfeW91bmdlcl90KSwgbWF4KEFnZV95b3VuZ2VyX3QpLCBsZW5ndGgub3V0PW4pKQ0KeSA8LSB3aXRoKHRlc3QsIHNlcShtaW4oRGlzdGFuY2VfdCksIG1heChEaXN0YW5jZV90KSwgbGVuZ3RoLm91dD1uKSkNCnogPC0gbWF0cml4KG5yb3c9biwgbmNvbD1uKSAjY29udG91ciBpbnRlcnByZXRzIHRoZSB6IG1hdHJpeCBhcyBhIHRhYmxlIG9mIGYoeFtpXSwgeVtqXSkgdmFsdWVzLCBzbyB0aGF0IHRoZSB4IGF4aXMgY29ycmVzcG9uZHMgdG8gcm93IG51bWJlciBhbmQgdGhlIHkgYXhpcyB0byBjb2x1bW4gbnVtYmVyLCB3aXRoIGNvbHVtbiAxIGF0IHRoZSBib3R0b20sIGkuZS4gYSA5MCBkZWdyZWUgY291bnRlci1jbG9ja3dpc2Ugcm90YXRpb24gb2YgdGhlIGNvbnZlbnRpb25hbCB0ZXh0dWFsIGxheW91dC4NCm5ld2RhdDEgPC0gZGF0YS5mcmFtZShBZ2VfRGlmZl90PXJlcChtZWRpYW4odGVzdCRBZ2VfRGlmZl90KSxuKSwNCiAgICAgICAgICAgICAgICAgICAgICBEaXN0YW5jZV90PXkpDQpmb3IoaSBpbiAxOm4pIHsNCiAgbmV3ZGF0MiA8LSBuZXdkYXQxDQogIG5ld2RhdDIkQWdlX3lvdW5nZXJfdCA8LSByZXAoeFtpXSxuKQ0KICB6W2ksXSA8LSBwcmVkaWN0KEdzdC5hbmUsIG5ld2RhdGE9bmV3ZGF0MiwgbGV2ZWw9MCkgI2VhY2ggcm93IGFzIGEgY29uc3RhbnQgeCB2YWx1ZSwgYnV0IGEgY2hhbmdpbmcgeSB2YWx1ZQ0KfQ0KDQpjbG91ZCA8LSB0ZXN0WyxjKCJBZ2VfeW91bmdlcl90IiwiRGlzdGFuY2VfdCIpXSAjeCBhbmQgeSBjb29yaWRuYXRlcyBvZiBhIHBvaW50IGNsb3VkDQpodWxsIDwtIGFzLm1hdHJpeChjbG91ZFtjaHVsbChjbG91ZCksXSkgI2Nvb3JkaW5hdGVzIG9mIHRoZSBjb252ZXggaHVsbCB2ZXJ0aWNlcyAoY2h1bGwgcmV0dXJucyB0aGUgaW5kaWNlcyBvZiB0aGUgdmVydGV4IHBvaW50cykNCmNoZWNrIDwtIGFzLm1hdHJpeChkYXRhLmZyYW1lKHg9cmVwKHgsIGVhY2g9biksIHk9cmVwKHksIHRpbWVzPW4pKSkgICNQLWJ5LTIgbWF0cml4IHdpdGggcG9pbnRzIHRvIGJlIGNoZWNrZWQNCnBvaW50Lng8LWNoZWNrWywxXQ0KcG9pbnQueTwtY2hlY2tbLDJdDQp4X2FycmF5PC1odWxsWywxXQ0KeV9hcnJheTwtaHVsbFssMl0NCndpdGhpbjwtc3A6OnBvaW50LmluLnBvbHlnb24ocG9pbnQueCxwb2ludC55LHhfYXJyYXkseV9hcnJheSkjIyAwOiBwb2ludCBpcyBvdXQgb2YgdGhlIHBvbHlnb24sIDE6IHBvaW50IGlzIGluIHRoZSBwb2x5Z29uLCAyOiBwb2ludCBpcyBvbiB0aGUgbGluZQ0KdW5pcXVlKHdpdGhpbikNCiMtLT4gbm8gZXJyb3IgY29kZXMNCndpdGhpbiA8LSBpZmVsc2Uod2l0aGluPj0xLCBUUlVFLCBGQUxTRSkNCnBsb3QoY2hlY2tbLDFdLCBjaGVja1ssMl0sIHBjaD0xNiwgY29sPWlmZWxzZSh3aXRoaW4sInJlZCIsICJ3aGl0ZSIpKQ0Kd2l0aGluIDwtIG1hdHJpeCh3aXRoaW4sIG5yb3c9biwgbmNvbD1uLCBieXJvdz1UUlVFKQ0Kelshd2l0aGluXSA8LSBOQSAjc2V0IGFsbCB6LXZhbHVlcyBvdXRzaWRlIHRoZSBjb252ZXggaHVsbCB0byBOQQ0KDQp0eiA8LSB0KHopICNiZWNhdXNlIHBsb3RseSBpbnRlcnByZXRzIHogYXMgdCh6KTsgYWx0ZXJuYXRpdmVseSwgdGhlIGFyZ3VtZW50IHRyYW5zcG9zZSBjYW4gYmUgc2V0IHRvIFRSVUUgaW4gcGxvdF9seSgpDQpmaWc2YSA8LSBwbG90X2x5KHg9eCwgeT15LCB6PXR6LCB0eXBlPSJjb250b3VyIiwNCiAgICAgICAgICAgICAgICAgY29sb3JzPWNyLA0KICAgICAgICAgICAgICAgICBhdXRvY29udG91cj1GLCBjb250b3Vycz1saXN0KHN0YXJ0PW1pbih6LG5hLnJtPVQpLGVuZD1tYXgoeixuYS5ybT1UKSxzaXplPTAuMDUpLA0KICAgICAgICAgICAgICAgICB3aWR0aD0xMzMwLCBoZWlnaHQ9MTMzMC0xMzMwLzYpICAgI3NldCBwbG90IHdpZHRoIGFuZCBoZWlnaHQgaW4gcHgNCmZpZzZhIDwtIGZpZzZhICU+JSBjb2xvcmJhcihsZW49MSwgdGl0bGU9IjxpPkcnJzwvaT48c3ViPlNUIiwgdGl0bGVmb250PWYubGFiLCB0aWNrZm9udD1mLmF4LCB0aWNrMD0tMS41LCBkdGljaz0wLjUpIyBleHByZXNzaW9uKERbZXN0XSkgZG9lcyBub3Qgd29yay4uLg0KZmlnNmEgPC0gZmlnNmEgJT4lIGFkZF9tYXJrZXJzKHg9aml0dGVyKGNsb3VkJEFnZV95b3VuZ2VyX3QsMTApLCB5PWppdHRlcihjbG91ZCREaXN0YW5jZV90LDEwKSwgaW5oZXJpdD1GQUxTRSwgc2hvd2xlZ2VuZD1GQUxTRSwNCiAgICAgICAgICAgICAgICAgICAgICAgICAgICAgICBtYXJrZXI9bGlzdChzeW1ib2w9ImNpcmNsZS1vcGVuIiwgY29sb3I9ImJsYWNrIiwgc2l6ZT0xNikpDQpmaWc2YSA8LSBmaWc2YSAlPiUgbGF5b3V0KHhheGlzPWxpc3QodGl0bGU9IkFHRV9CQVNFIiwgdGl0bGVmb250PWYubGFiLCB0aWNrZm9udD1mLmF4LCBzaG93Z3JpZD1GQUxTRSwgemVyb2xpbmU9RkFMU0UsDQogICAgICAgICAgICAgICAgICAgICAgICAgICAgICAgICAgICAgc2hvd2xpbmU9VFJVRSwgdGlja2xlbj0xNSwgdGlja3dpZHRoPTMsIGxpbmV3aWR0aD0zKSwgICAgICAgICAgICNhZGQgYXhpcyBsYWJlbHMNCiAgICAgICAgICAgICAgICAgICAgICAgICAgeWF4aXM9bGlzdCh0aXRsZT0iR0VPX0RJU1QiLCB0aXRsZWZvbnQ9Zi5sYWIsIHRpY2tmb250PWYuYXgsIHNob3dncmlkPUZBTFNFLCB6ZXJvbGluZT1GQUxTRSwNCiAgICAgICAgICAgICAgICAgICAgICAgICAgICAgICAgICAgICBzaG93bGluZT1UUlVFLCB0aWNrbGVuPTE1LCB0aWNrd2lkdGg9MywgbGluZXdpZHRoPTMpLA0KICAgICAgICAgICAgICAgICAgICAgICAgICBtYXJnaW49bGlzdChsPTYwLCByPTgwLCB0PTIwLCBiPTYwKSkgICAgICAgICAgICAgICAgICAgICAgICAgICAgI3NldHMgdGhlIHBsb3QgbWFyZ2luIGluIHB4LCBzZWUgaHR0cHM6Ly9wbG90bHkuY29tL3IvcmVmZXJlbmNlL2xheW91dC8jbGF5b3V0LWF1dG9zaXplDQpmaWc2YQ0KIyMjIyMjIyMjIyMjIyMjIyMjIyMjIyMjIyMNCiMjI094YSMjIyMNCnRlc3Q8LWFzLmRhdGEuZnJhbWUobW9kZWwubWF0cml4KGNnZC5veGEpKQ0KDQp4IDwtIHdpdGgodGVzdCwgc2VxKG1pbihBZ2VfeW91bmdlcl90KSwgbWF4KEFnZV95b3VuZ2VyX3QpLCBsZW5ndGgub3V0PW4pKQ0KeSA8LSB3aXRoKHRlc3QsIHNlcShtaW4oZ2VvZGlzdF90KSwgbWF4KGdlb2Rpc3RfdCksIGxlbmd0aC5vdXQ9bikpDQp6IDwtIG1hdHJpeChucm93PW4sIG5jb2w9bikgI2NvbnRvdXIgaW50ZXJwcmV0cyB0aGUgeiBtYXRyaXggYXMgYSB0YWJsZSBvZiBmKHhbaV0sIHlbal0pIHZhbHVlcywgc28gdGhhdCB0aGUgeCBheGlzIGNvcnJlc3BvbmRzIHRvIHJvdyBudW1iZXIgYW5kIHRoZSB5IGF4aXMgdG8gY29sdW1uIG51bWJlciwgd2l0aCBjb2x1bW4gMSBhdCB0aGUgYm90dG9tLCBpLmUuIGEgOTAgZGVncmVlIGNvdW50ZXItY2xvY2t3aXNlIHJvdGF0aW9uIG9mIHRoZSBjb252ZW50aW9uYWwgdGV4dHVhbCBsYXlvdXQuDQpuZXdkYXQxIDwtIGRhdGEuZnJhbWUoQWdlX0RpZmZfdD1yZXAobWVkaWFuKHRlc3QkQWdlX0RpZmZfdCksbiksDQogICAgICAgICAgICAgICAgICAgICAgZ2VvZGlzdF90PXkpDQpmb3IoaSBpbiAxOm4pIHsNCiAgbmV3ZGF0MiA8LSBuZXdkYXQxDQogIG5ld2RhdDIkQWdlX3lvdW5nZXJfdCA8LSByZXAoeFtpXSxuKQ0KICB6W2ksXSA8LSBwcmVkaWN0KGNnZC5veGEsIG5ld2RhdGE9bmV3ZGF0MiwgbGV2ZWw9MCkgI2VhY2ggcm93IGFzIGEgY29uc3RhbnQgeCB2YWx1ZSwgYnV0IGEgY2hhbmdpbmcgeSB2YWx1ZQ0KfQ0KDQpjbG91ZCA8LSB0ZXN0WyxjKCJBZ2VfeW91bmdlcl90IiwiZ2VvZGlzdF90IildICN4IGFuZCB5IGNvb3JpZG5hdGVzIG9mIGEgcG9pbnQgY2xvdWQNCmh1bGwgPC0gYXMubWF0cml4KGNsb3VkW2NodWxsKGNsb3VkKSxdKSAjY29vcmRpbmF0ZXMgb2YgdGhlIGNvbnZleCBodWxsIHZlcnRpY2VzIChjaHVsbCByZXR1cm5zIHRoZSBpbmRpY2VzIG9mIHRoZSB2ZXJ0ZXggcG9pbnRzKQ0KY2hlY2sgPC0gYXMubWF0cml4KGRhdGEuZnJhbWUoeD1yZXAoeCwgZWFjaD1uKSwgeT1yZXAoeSwgdGltZXM9bikpKSAgI1AtYnktMiBtYXRyaXggd2l0aCBwb2ludHMgdG8gYmUgY2hlY2tlZA0KcG9pbnQueDwtY2hlY2tbLDFdDQpwb2ludC55PC1jaGVja1ssMl0NCnhfYXJyYXk8LWh1bGxbLDFdDQp5X2FycmF5PC1odWxsWywyXQ0Kd2l0aGluPC1zcDo6cG9pbnQuaW4ucG9seWdvbihwb2ludC54LHBvaW50LnkseF9hcnJheSx5X2FycmF5KSMjIDA6IHBvaW50IGlzIG91dCBvZiB0aGUgcG9seWdvbiwgMTogcG9pbnQgaXMgaW4gdGhlIHBvbHlnb24sIDI6IHBvaW50IGlzIG9uIHRoZSBsaW5lDQp1bmlxdWUod2l0aGluKQ0KIy0tPiBubyBlcnJvciBjb2Rlcw0Kd2l0aGluIDwtIGlmZWxzZSh3aXRoaW4+PTEsIFRSVUUsIEZBTFNFKQ0KcGxvdChjaGVja1ssMV0sIGNoZWNrWywyXSwgcGNoPTE2LCBjb2w9aWZlbHNlKHdpdGhpbiwicmVkIiwgIndoaXRlIikpDQp3aXRoaW4gPC0gbWF0cml4KHdpdGhpbiwgbnJvdz1uLCBuY29sPW4sIGJ5cm93PVRSVUUpDQp6WyF3aXRoaW5dIDwtIE5BICNzZXQgYWxsIHotdmFsdWVzIG91dHNpZGUgdGhlIGNvbnZleCBodWxsIHRvIE5BDQoNCnR6IDwtIHQoeikgI2JlY2F1c2UgcGxvdGx5IGludGVycHJldHMgeiBhcyB0KHopOyBhbHRlcm5hdGl2ZWx5LCB0aGUgYXJndW1lbnQgdHJhbnNwb3NlIGNhbiBiZSBzZXQgdG8gVFJVRSBpbiBwbG90X2x5KCkNCmZpZzZiIDwtIHBsb3RfbHkoeD14LCB5PXksIHo9dHosIHR5cGU9ImNvbnRvdXIiLA0KICAgICAgICAgICAgICAgICBjb2xvcnM9Y3IsDQogICAgICAgICAgICAgICAgIGF1dG9jb250b3VyPUYsIGNvbnRvdXJzPWxpc3Qoc3RhcnQ9bWluKHosbmEucm09VCksZW5kPW1heCh6LG5hLnJtPVQpLHNpemU9MC4wNSksDQogICAgICAgICAgICAgICAgIHdpZHRoPTEzMzAsIGhlaWdodD0xMzMwLTEzMzAvNikgICAjc2V0IHBsb3Qgd2lkdGggYW5kIGhlaWdodCBpbiBweA0KZmlnNmIgPC0gZmlnNmIgJT4lIGNvbG9yYmFyKGxlbj0xLCB0aXRsZT0iPGk+Y0dEPC9pPiIsIHRpdGxlZm9udD1mLmxhYiwgdGlja2ZvbnQ9Zi5heCwgdGljazA9LTEuNSwgZHRpY2s9MC41KSMgZXhwcmVzc2lvbihEW2VzdF0pIGRvZXMgbm90IHdvcmsuLi4NCmZpZzZiIDwtIGZpZzZiICU+JSBhZGRfbWFya2Vycyh4PWppdHRlcihjbG91ZCRBZ2VfeW91bmdlcl90LDEwKSwgeT1qaXR0ZXIoY2xvdWQkZ2VvZGlzdF90LDEwKSwgaW5oZXJpdD1GQUxTRSwgc2hvd2xlZ2VuZD1GQUxTRSwNCiAgICAgICAgICAgICAgICAgICAgICAgICAgICAgICBtYXJrZXI9bGlzdChzeW1ib2w9ImNpcmNsZS1vcGVuIiwgY29sb3I9ImJsYWNrIiwgc2l6ZT0xNikpDQpmaWc2YiA8LSBmaWc2YiAlPiUgbGF5b3V0KHhheGlzPWxpc3QodGl0bGU9IkFHRV9CQVNFIiwgdGl0bGVmb250PWYubGFiLCB0aWNrZm9udD1mLmF4LCBzaG93Z3JpZD1GQUxTRSwgemVyb2xpbmU9RkFMU0UsDQogICAgICAgICAgICAgICAgICAgICAgICAgICAgICAgICAgICAgc2hvd2xpbmU9VFJVRSwgdGlja2xlbj0xNSwgdGlja3dpZHRoPTMsIGxpbmV3aWR0aD0zKSwgICAgICAgICAgICNhZGQgYXhpcyBsYWJlbHMNCiAgICAgICAgICAgICAgICAgICAgICAgICAgeWF4aXM9bGlzdCh0aXRsZT0iR0VPX0RJU1QiLCB0aXRsZWZvbnQ9Zi5sYWIsIHRpY2tmb250PWYuYXgsIHNob3dncmlkPUZBTFNFLCB6ZXJvbGluZT1GQUxTRSwNCiAgICAgICAgICAgICAgICAgICAgICAgICAgICAgICAgICAgICBzaG93bGluZT1UUlVFLCB0aWNrbGVuPTE1LCB0aWNrd2lkdGg9MywgbGluZXdpZHRoPTMpLA0KICAgICAgICAgICAgICAgICAgICAgICAgICBtYXJnaW49bGlzdChsPTYwLCByPTgwLCB0PTIwLCBiPTYwKSkgICAgICAgICAgICAgICAgICAgICAgICAgICAgI3NldHMgdGhlIHBsb3QgbWFyZ2luIGluIHB4LCBzZWUgaHR0cHM6Ly9wbG90bHkuY29tL3IvcmVmZXJlbmNlL2xheW91dC8jbGF5b3V0LWF1dG9zaXplDQpmaWc2Yg0KIyMjIyMjIyMjIyMjIyMjIyMjIw0KDQojIyMjI3BvbCBHc3R+R2VvZGlzdCpBZ2VfeW91bmdlciMjDQoNCnRlc3Q8LWFzLmRhdGEuZnJhbWUobW9kZWwubWF0cml4KEdzdC5wb2wpKQ0KDQp4IDwtIHdpdGgodGVzdCwgc2VxKG1pbihBZ2VfeW91bmdlcl90KSwgbWF4KEFnZV95b3VuZ2VyX3QpLCBsZW5ndGgub3V0PW4pKQ0KeSA8LSB3aXRoKHRlc3QsIHNlcShtaW4oRGlzdGFuY2VfdCksIG1heChEaXN0YW5jZV90KSwgbGVuZ3RoLm91dD1uKSkNCnogPC0gbWF0cml4KG5yb3c9biwgbmNvbD1uKSAjY29udG91ciBpbnRlcnByZXRzIHRoZSB6IG1hdHJpeCBhcyBhIHRhYmxlIG9mIGYoeFtpXSwgeVtqXSkgdmFsdWVzLCBzbyB0aGF0IHRoZSB4IGF4aXMgY29ycmVzcG9uZHMgdG8gcm93IG51bWJlciBhbmQgdGhlIHkgYXhpcyB0byBjb2x1bW4gbnVtYmVyLCB3aXRoIGNvbHVtbiAxIGF0IHRoZSBib3R0b20sIGkuZS4gYSA5MCBkZWdyZWUgY291bnRlci1jbG9ja3dpc2Ugcm90YXRpb24gb2YgdGhlIGNvbnZlbnRpb25hbCB0ZXh0dWFsIGxheW91dC4NCm5ld2RhdDEgPC0gZGF0YS5mcmFtZShBZ2VfRGlmZl90PXJlcChtZWRpYW4odGVzdCRBZ2VfRGlmZl90KSxuKSwNCiAgICAgICAgICAgICAgICAgICAgICBEaXN0YW5jZV90PXkpDQpmb3IoaSBpbiAxOm4pIHsNCiAgbmV3ZGF0MiA8LSBuZXdkYXQxDQogIG5ld2RhdDIkQWdlX3lvdW5nZXJfdCA8LSByZXAoeFtpXSxuKQ0KICB6W2ksXSA8LSBwcmVkaWN0KEdzdC5wb2wsIG5ld2RhdGE9bmV3ZGF0MiwgbGV2ZWw9MCkgI2VhY2ggcm93IGFzIGEgY29uc3RhbnQgeCB2YWx1ZSwgYnV0IGEgY2hhbmdpbmcgeSB2YWx1ZQ0KfQ0KDQpjbG91ZCA8LSB0ZXN0WyxjKCJBZ2VfeW91bmdlcl90IiwiRGlzdGFuY2VfdCIpXSAjeCBhbmQgeSBjb29yaWRuYXRlcyBvZiBhIHBvaW50IGNsb3VkDQpodWxsIDwtIGFzLm1hdHJpeChjbG91ZFtjaHVsbChjbG91ZCksXSkgI2Nvb3JkaW5hdGVzIG9mIHRoZSBjb252ZXggaHVsbCB2ZXJ0aWNlcyAoY2h1bGwgcmV0dXJucyB0aGUgaW5kaWNlcyBvZiB0aGUgdmVydGV4IHBvaW50cykNCmNoZWNrIDwtIGFzLm1hdHJpeChkYXRhLmZyYW1lKHg9cmVwKHgsIGVhY2g9biksIHk9cmVwKHksIHRpbWVzPW4pKSkgICNQLWJ5LTIgbWF0cml4IHdpdGggcG9pbnRzIHRvIGJlIGNoZWNrZWQNCnBvaW50Lng8LWNoZWNrWywxXQ0KcG9pbnQueTwtY2hlY2tbLDJdDQp4X2FycmF5PC1odWxsWywxXQ0KeV9hcnJheTwtaHVsbFssMl0NCndpdGhpbjwtc3A6OnBvaW50LmluLnBvbHlnb24ocG9pbnQueCxwb2ludC55LHhfYXJyYXkseV9hcnJheSkjIyAwOiBwb2ludCBpcyBvdXQgb2YgdGhlIHBvbHlnb24sIDE6IHBvaW50IGlzIGluIHRoZSBwb2x5Z29uLCAyOiBwb2ludCBpcyBvbiB0aGUgbGluZQ0KdW5pcXVlKHdpdGhpbikNCiMtLT4gbm8gZXJyb3IgY29kZXMNCndpdGhpbiA8LSBpZmVsc2Uod2l0aGluPj0xLCBUUlVFLCBGQUxTRSkNCnBsb3QoY2hlY2tbLDFdLCBjaGVja1ssMl0sIHBjaD0xNiwgY29sPWlmZWxzZSh3aXRoaW4sInJlZCIsICJ3aGl0ZSIpKQ0Kd2l0aGluIDwtIG1hdHJpeCh3aXRoaW4sIG5yb3c9biwgbmNvbD1uLCBieXJvdz1UUlVFKQ0Kelshd2l0aGluXSA8LSBOQSAjc2V0IGFsbCB6LXZhbHVlcyBvdXRzaWRlIHRoZSBjb252ZXggaHVsbCB0byBOQQ0KDQp0eiA8LSB0KHopICNiZWNhdXNlIHBsb3RseSBpbnRlcnByZXRzIHogYXMgdCh6KTsgYWx0ZXJuYXRpdmVseSwgdGhlIGFyZ3VtZW50IHRyYW5zcG9zZSBjYW4gYmUgc2V0IHRvIFRSVUUgaW4gcGxvdF9seSgpDQpsaWJyYXJ5KHBsb3RseSkNCmZpZzZjIDwtIHBsb3RfbHkoeD14LCB5PXksIHo9dHosIHR5cGU9ImNvbnRvdXIiLA0KICAgICAgICAgICAgICAgICBjb2xvcnM9Y3IsDQogICAgICAgICAgICAgICAgIGF1dG9jb250b3VyPUYsIGNvbnRvdXJzPWxpc3Qoc3RhcnQ9bWluKHosbmEucm09VCksZW5kPW1heCh6LG5hLnJtPVQpLHNpemU9MC4wMiksDQogICAgICAgICAgICAgICAgIHdpZHRoPTEzMzAsIGhlaWdodD0xMzMwLTEzMzAvNikgICAjc2V0IHBsb3Qgd2lkdGggYW5kIGhlaWdodCBpbiBweA0KZmlnNmMgPC0gZmlnNmMgJT4lIGNvbG9yYmFyKGxlbj0xLCB0aXRsZT0iPGk+RycnPC9pPjxzdWI+U1QiLCB0aXRsZWZvbnQ9Zi5sYWIsIHRpY2tmb250PWYuYXgsIHRpY2swPS0xLjUsIGR0aWNrPTAuNSkjIGV4cHJlc3Npb24oRFtlc3RdKSBkb2VzIG5vdCB3b3JrLi4uDQpmaWc2YyA8LSBmaWc2YyAlPiUgYWRkX21hcmtlcnMoeD1qaXR0ZXIoY2xvdWQkQWdlX3lvdW5nZXJfdCwxMCksIHk9aml0dGVyKGNsb3VkJERpc3RhbmNlX3QsMTApLCBpbmhlcml0PUZBTFNFLCBzaG93bGVnZW5kPUZBTFNFLA0KICAgICAgICAgICAgICAgICAgICAgICAgICAgICAgIG1hcmtlcj1saXN0KHN5bWJvbD0iY2lyY2xlLW9wZW4iLCBjb2xvcj0iYmxhY2siLCBzaXplPTE2KSkNCmZpZzZjIDwtIGZpZzZjICU+JSBsYXlvdXQoeGF4aXM9bGlzdCh0aXRsZT0iQUdFX0JBU0UiLCB0aXRsZWZvbnQ9Zi5sYWIsIHRpY2tmb250PWYuYXgsIHNob3dncmlkPUZBTFNFLCB6ZXJvbGluZT1GQUxTRSwNCiAgICAgICAgICAgICAgICAgICAgICAgICAgICAgICAgICAgICBzaG93bGluZT1UUlVFLCB0aWNrbGVuPTE1LCB0aWNrd2lkdGg9MywgbGluZXdpZHRoPTMpLCAgICAgICAgICAgI2FkZCBheGlzIGxhYmVscw0KICAgICAgICAgICAgICAgICAgICAgICAgICB5YXhpcz1saXN0KHRpdGxlPSJHRU9fRElTVCIsIHRpdGxlZm9udD1mLmxhYiwgdGlja2ZvbnQ9Zi5heCwgc2hvd2dyaWQ9RkFMU0UsIHplcm9saW5lPUZBTFNFLA0KICAgICAgICAgICAgICAgICAgICAgICAgICAgICAgICAgICAgIHNob3dsaW5lPVRSVUUsIHRpY2tsZW49MTUsIHRpY2t3aWR0aD0zLCBsaW5ld2lkdGg9MyksDQogICAgICAgICAgICAgICAgICAgICAgICAgIG1hcmdpbj1saXN0KGw9NjAsIHI9ODAsIHQ9MjAsIGI9NjApKSAgICAgICAgICAgICAgICAgICAgICAgICAgICAjc2V0cyB0aGUgcGxvdCBtYXJnaW4gaW4gcHgsIHNlZSBodHRwczovL3Bsb3RseS5jb20vci9yZWZlcmVuY2UvbGF5b3V0LyNsYXlvdXQtYXV0b3NpemUNCmZpZzZjDQoNCg0KDQoNCmBgYA0KUG9wdWxhdGlvbiBncmFwaA0KYGBge3J9DQpwYXIobWZyb3c9Yyg2LDMpLG1hcj1jKDEsMSwzLDEpKQ0KIyMgQmUjIw0KcGxvdChnQmVfYW5lLCBlZGdlLmNvbG9yPWMoIiMxYjllNzciKSx2ZXJ0ZXguY29sb3I9IiNkNjYwNGQiLHZlcnRleC5sYWJlbD1OQSxlZGdlLndpZHRoPTIpDQptdGV4dChhZGo9MCxsaW5lPS0xLCJCZSIpDQptdGV4dChhZGo9MC41LGxpbmU9MSxleHByZXNzaW9uKGl0YWxpYyhBLm5lbSkpKQ0KcGxvdCgxLHR5cGU9J24nLGF4ZXM9Rix4bGFiPSIiLHlsYWI9IiIpDQptdGV4dChhZGo9MC41LGxpbmU9MSxleHByZXNzaW9uKGl0YWxpYyhPLmFjZSkpKQ0KcGxvdChnQmVfcG9sLCBlZGdlLmNvbG9yPWMoIiMxYjllNzciKSx2ZXJ0ZXguY29sb3I9IiNkNjYwNGQiLHZlcnRleC5sYWJlbD1OQSxlZGdlLndpZHRoPTIpDQptdGV4dChhZGo9MC41LGxpbmU9MSxleHByZXNzaW9uKGl0YWxpYyhQLm11bCkpKQ0KDQojI0VzdA0KcGxvdChnRXN0X2FuZSwgZWRnZS5jb2xvcj1jKCIjMWI5ZTc3IiksdmVydGV4LmNvbG9yPSIjZDY2MDRkIix2ZXJ0ZXgubGFiZWw9TkEsZWRnZS53aWR0aD0yKQ0KbXRleHQoYWRqPTAsbGluZT0tMSwiRXN0IikNCnBsb3QoZ0VzdF9veGEsIGVkZ2UuY29sb3I9YygiIzFiOWU3NyIpLHZlcnRleC5jb2xvcj0iI2Q2NjA0ZCIsdmVydGV4LmxhYmVsPU5BLGVkZ2Uud2lkdGg9MikNCnBsb3QoZ0VzdF9wb2wsIGVkZ2UuY29sb3I9YygiIzFiOWU3NyIpLHZlcnRleC5jb2xvcj0iI2Q2NjA0ZCIsdmVydGV4LmxhYmVsPU5BLGVkZ2Uud2lkdGg9MikNCiAgICAgDQojIyBGck4NCnBsb3QoZ0ZyTl9hbmUsIGVkZ2UuY29sb3I9YygiIzFiOWU3NyIpLHZlcnRleC5jb2xvcj0iI2Q2NjA0ZCIsdmVydGV4LmxhYmVsPU5BLGVkZ2Uud2lkdGg9MikNCm10ZXh0KGFkaj0wLGxpbmU9LTEsIkZyTiIpDQpwbG90KDEsdHlwZT0nbicsYXhlcz1GLHhsYWI9IiIseWxhYj0iIikNCnBsb3QoZ0ZyTl9wb2wsIGVkZ2UuY29sb3I9YygiIzFiOWU3NyIpLHZlcnRleC5jb2xvcj0iI2Q2NjA0ZCIsdmVydGV4LmxhYmVsPU5BLGVkZ2Uud2lkdGg9MikNCiAgICAgDQojI0dlRQ0KcGxvdChnR2VFX2FuZSwgZWRnZS5jb2xvcj1jKCIjMWI5ZTc3IiksdmVydGV4LmNvbG9yPSIjZDY2MDRkIix2ZXJ0ZXgubGFiZWw9TkEsZWRnZS53aWR0aD0yKQ0KbXRleHQoYWRqPTAsbGluZT0tMSwiR2VFIikNCnBsb3QoZ0dlRV9veGEsIGVkZ2UuY29sb3I9YygiIzFiOWU3NyIpLHZlcnRleC5jb2xvcj0iI2Q2NjA0ZCIsdmVydGV4LmxhYmVsPU5BLGVkZ2Uud2lkdGg9MikNCnBsb3QoZ0dlRV9wb2wsIGVkZ2UuY29sb3I9YygiIzFiOWU3NyIpLHZlcnRleC5jb2xvcj0iI2Q2NjA0ZCIsdmVydGV4LmxhYmVsPU5BLGVkZ2Uud2lkdGg9MikNCiAgICAgDQojIyBHZVcNCnBsb3QoZ0dlV19hbmUsIGVkZ2UuY29sb3I9YygiIzFiOWU3NyIpLHZlcnRleC5jb2xvcj0iI2Q2NjA0ZCIsdmVydGV4LmxhYmVsPU5BLGVkZ2Uud2lkdGg9MikNCm10ZXh0KGFkaj0wLGxpbmU9LTEsIkdlVyIpDQpwbG90KGdHZVdfb3hhLCBlZGdlLmNvbG9yPWMoIiMxYjllNzciKSx2ZXJ0ZXguY29sb3I9IiNkNjYwNGQiLHZlcnRleC5sYWJlbD1OQSxlZGdlLndpZHRoPTIpDQpwbG90KGdHZVdfcG9sLCBlZGdlLmNvbG9yPWMoIiMxYjllNzciKSx2ZXJ0ZXguY29sb3I9IiNkNjYwNGQiLHZlcnRleC5sYWJlbD1OQSxlZGdlLndpZHRoPTIpDQogICAgIA0KIyNTd1MNCnBsb3QoZ1N3U19hbmUsIGVkZ2UuY29sb3I9YygiIzFiOWU3NyIpLHZlcnRleC5jb2xvcj0iI2Q2NjA0ZCIsdmVydGV4LmxhYmVsPU5BLGVkZ2Uud2lkdGg9MikNCm10ZXh0KGFkaj0wLGxpbmU9LTAuNSwiU3dTIikNCnBsb3QoZ1N3U19veGEsIGVkZ2UuY29sb3I9YygiIzFiOWU3NyIpLHZlcnRleC5jb2xvcj0iI2Q2NjA0ZCIsdmVydGV4LmxhYmVsPU5BLGVkZ2Uud2lkdGg9MikNCnBsb3QoZ1N3U19wb2wsIGVkZ2UuY29sb3I9YygiIzFiOWU3NyIpLHZlcnRleC5jb2xvcj0iI2Q2NjA0ZCIsdmVydGV4LmxhYmVsPU5BLGVkZ2Uud2lkdGg9MikNCiAgICAgDQoNCnBhcihtZnJvdz1jKDEsMSkpDQpwbG90KDEsdHlwZT0nbicsYXhlcz1GLHhsYWI9IiIseWxhYj0iIikNCmxlZ2VuZCgiY2VudGVyIixsZWdlbmQ9YygiUG9wdWxhdGlvbiIsIkVkZ2UiKSwNCiAgICAgICBwY2g9YygyMSxOQSksbHR5PWMoTkEsMSksbHdkPWMoTkEsMikscHQuYmc9YygiI2Q2NjA0ZCIsTkEpLGNleD0yLGNvbD1jKCJibGFjayIsIiMxYjllNzciKSkNCg0KYGBgDQojIyBGaWd1cmVfUzkNCmBgYHtyfQ0KZWZmLk5vZGVfeW91bmdlcjwtZWZmZWN0KCJBZ2VfdCIsbm9kZS5maW4sIHBhcnRpYWwucmVzaWR1YWxzPVQpDQpwbG90LmRhdGEuQWdlLmFsbDwtZWZmLk5vZGVfeW91bmdlciRkYXRhDQpwbG90LmRhdGEuQWdlLmZpdDwtd2l0aChlZmYuTm9kZV95b3VuZ2VyLGNiaW5kKHgsZml0LGxvd2VyLHVwcGVyKSkNCg0KcGxvdCgzLHR5cGU9Im4iLHhsaW09YygtMiwzKSx5bGltPWMoLTMsMi41KSx4YXh0PSduJyxhbm49Rix5YXh0PSduJykNCmF4aXMoc2lkZT0yLGx3ZD0yLGNleC5heGlzPTEuMykNCmF4aXMoc2lkZT0xLGx3ZD0yLGNleC5heGlzPTEuMyxhdD1jKC0wLjY5Mjk2OTIsMC43OTE2ODQ5LDEuNjAxMjc3LDIuMTgxNTUxKSxsYWJlbHMgPSBjKDUwLDE1MCwyNTAsMzUwKSkNCmJveChsd2Q9MikNCnRpdGxlKHhsYWI9IkFHRV9CQVNFICh5ZWFyKSIseWxhYj1leHByZXNzaW9uKGl0YWxpYyhwYXN0ZSgiRCIpKVtQU10pLGxpbmU9MyxjZXgubGFiPTEuNSkNCiN3aXRoKHBsb3QuZGF0YS5BZ2UuZml0LHBvbHlnb24oeD1jKEFnZV95b3VuZ2VyX3QsIHJldihBZ2VfeW91bmdlcl90KSkseT1jKGxvd2VyLCByZXYodXBwZXIpKSwNCiMgICAgICAgICAgICAgICAgICAgICAgICAgICAgICAgY29sPWMoIiNkY2RjZGMiKSwgYm9yZGVyPU5BKSkgDQoNCndpdGgoZGlzdFtkaXN0JFNwZWNpZXMueD09IkFuZSIsXSwgcG9pbnRzKHg9aml0dGVyKEFnZV95b3VuZ2VyX3QpLHk9RHBzX3QscGNoPTE5LGNleD0xLjUsDQogICAgICAgICAgICAgICAgICAgICAgICAgICAgICAgICAgICAgICAgY29sPWMoIiNiYjU1NjYiKSkpDQoNCndpdGgoZGlzdFtkaXN0JFNwZWNpZXMueD09Ik94YSIsXSwgcG9pbnRzKHg9aml0dGVyKEFnZV95b3VuZ2VyX3QpLHk9RHBzX3QscGNoPTE5LGNleD0xLjUsIA0KICAgICAgICAgICAgICAgICAgICAgICAgICAgICAgICAgICAgICAgICAgY29sPWMoIiNkZGFhMzMiKSkpDQp3aXRoKGRpc3RbZGlzdCRTcGVjaWVzLng9PSJQb2wiLF0sIHBvaW50cyh4PWppdHRlcihBZ2VfeW91bmdlcl90KSx5PURwc190LHBjaD0xOSxjZXg9MS41LA0KICAgICAgICAgICAgICAgICAgICAgICAgICAgICAgICAgICAgICAgICAgY29sPWMoIiMwMDQ0ODgiKSkpDQoNCndpdGgocGxvdC5kYXRhLkFnZS5maXQsbGluZXMoQWdlX3lvdW5nZXJfdCxmaXQsY29sPXJnYigwLDAsMCxtYXhDb2xvclZhbHVlID0gMjU1KSxsd2Q9MixsdHk9NSkpDQpgYGANCg0KI0ZpZ3VyZV9TOQ0KYGBge3J9DQpwYXIobWZyb3c9YygxLDMpLG1hcj1jKDUsNSwyLDIpKQ0KZWZmLk5vZGVfeW91bmdlcjwtZWZmZWN0KCJBZ2VfdCIsbm9kZS5maW4sIHBhcnRpYWwucmVzaWR1YWxzPVQpDQpwbG90LmRhdGEuQWdlLmFsbDwtZWZmLk5vZGVfeW91bmdlciRkYXRhDQpwbG90LmRhdGEuQWdlLmZpdDwtd2l0aChlZmYuTm9kZV95b3VuZ2VyLGNiaW5kKHgsZml0LGxvd2VyLHVwcGVyKSkNCg0KI21lYW48LW1lYW4oYm94Y294KE5vZGUkQWdlX2FicykpDQojc2Q8LXNkKGJveGNveChOb2RlJEFnZV9hYnMpKQ0KIygoNTBeMC40NS0xKS8wLjQ1LW1lYW4pL3NkDQojKCgxNTBeMC40NS0xKS8wLjQ1LW1lYW4pL3NkDQojKCgyNTBeMC40NS0xKS8wLjQ1LW1lYW4pL3NkDQojKCgzNTBeMC40NS0xKS8wLjQ1LW1lYW4pL3NkDQoNCnBsb3QoMyx0eXBlPSJuIix4bGltPWMoLTIsMikseWxpbT1jKC0zLDIuNSkseGF4dD0nbicsYW5uPUYseWF4dD0nbicpDQpheGlzKHNpZGU9Mixsd2Q9MixjZXguYXhpcz0xLjUpDQpheGlzKHNpZGU9MSxsd2Q9MixjZXguYXhpcz0xLjUsYXQ9YygtMS4xMDA1MzUsMC4xNTkxMzksMS4wMzcxMjQsMS43MzYwMjcpLGxhYmVscyA9IGMoNTAsMTUwLDI1MCwzNTApKQ0KYm94KGx3ZD0yKQ0KDQojI25vZGUuZmluPC1sbWUoY2xvc2VuZXNzX3R+QWdlX3QscmFuZG9tPX4xfExXLGRhdGE9Tm9kZSkNCg0KdGl0bGUoeGxhYj0iUE9QX0FHRSAoeWVhcikiLHlsYWI9ZXhwcmVzc2lvbihpdGFsaWMocGFzdGUoIk5IYyIpKSksbGluZT0zLGNleC5sYWI9MS41KQ0KI3dpdGgocGxvdC5kYXRhLkFnZS5maXQscG9seWdvbih4PWMoQWdlX3QsIHJldihBZ2VfdCkpLHk9Yyhsb3dlciwgcmV2KHVwcGVyKSksDQojICAgICAgICAgICAgICAgICAgICAgICAgICAgICAgIGNvbD1jKCIjZGNkY2RjIiksIGJvcmRlcj1OQSkpIA0KDQp3aXRoKE5vZGVbTm9kZSRTcGVjaWVzPT0iQW5lIixdLCBwb2ludHMoeD1qaXR0ZXIoQWdlX3QpLHk9Y2xvc2VuZXNzX3QscGNoPTE5LGNleD0yLA0KICAgICAgICAgICAgICAgICAgICAgICAgICAgICAgICAgICAgICAgIGNvbD1jKCIjYmI1NTY2IikpKQ0KDQp3aXRoKE5vZGVbTm9kZSRTcGVjaWVzPT0iT3hhIixdLCBwb2ludHMoeD1qaXR0ZXIoQWdlX3QpLHk9Y2xvc2VuZXNzX3QscGNoPTE5LGNleD0yLCANCiAgICAgICAgICAgICAgICAgICAgICAgICAgICAgICAgICAgICAgICBjb2w9YygiI2RkYWEzMyIpKSkNCndpdGgoTm9kZVtOb2RlJFNwZWNpZXM9PSJQb2wiLF0sIHBvaW50cyh4PWppdHRlcihBZ2VfdCkseT1jbG9zZW5lc3NfdCxwY2g9MTksY2V4PTIsIA0KICAgICAgICAgICAgICAgICAgICAgICAgICAgICAgICAgICAgICAgIGNvbD1jKCIjMDA0NDg4IikpKQ0KDQp3aXRoKHBsb3QuZGF0YS5BZ2UuZml0LGxpbmVzKEFnZV90LGZpdCxjb2w9cmdiKDAsMCwwLG1heENvbG9yVmFsdWUgPSAyNTUpLGx3ZD0yLGx0eT01KSkNCm10ZXh0KGFkaj0tMC4yLCIoQSkiLGNleD0xLjUpDQpncmFwaGljczo6bGVnZW5kKC0yLC0yLGxlZ2VuZD1leHByZXNzaW9uKGl0YWxpYyhBLm5lbS4pLGl0YWxpYyhPLmFjZS4pLGl0YWxpYyhQLm11bC4pLCJwID4gMC4wNSIsInAiPD0iMC4wNSIpLGJ0eT0nbicsDQogICAgICAgICAgICAgICAgIHBjaD1jKDE5LDE5LDE5LE5BLE5BKSxjb2w9YygiI2JiNTU2NiIsIiNkZGFhMzMiLCIjMDA0NDg4IiwiYmxhY2siLCJibGFjayIpLGx0eT1jKE5BLE5BLE5BLDUsMSksDQogICAgICAgICAgICAgICAgIHNlZy5sZW49MC41LGNleD0xLjMseC5pbnRlcnNwPTAuMSx5LmludGVyc3A9MC42KQ0KDQoNCiMjbGluay5maW48LWxtZShEaWZmX0dfUF90fkFnZV95b3VuZ2VyX3QscmFuZG9tPX4xfExXLngsY29ycmVsYXRpb249Y29yTUxQRShmb3JtPX5ub2RlXzErbm9kZV8yfExXLngpLGRhdGE9TGluaykNCiNtZWFuPC1tZWFuKGJveGNveChMaW5rJEFnZV9hYnNfeSkpIyMgbGFtYmRhPTAuMTUsIGFkZGVkPTANCiNzZDwtc2QoYm94Y294KExpbmskQWdlX2Fic195KSkNCiMoKDUwXjAuMTUtMSkvMC4xNS1tZWFuKS9zZA0KIygoMTUwXjAuMTUtMSkvMC4xNS1tZWFuKS9zZA0KIygoMjUwXjAuMTUtMSkvMC4xNS1tZWFuKS9zZA0KIygoMzUwXjAuMTUtMSkvMC4xNS1tZWFuKS9zZA0KDQplZmYuTGlua195b3VuZ2VyPC1lZmZlY3QoIkFnZV95b3VuZ2VyX3QiLGxpbmsuZmluLCBwYXJ0aWFsLnJlc2lkdWFscz1UKQ0KcGxvdC5kYXRhLkFnZS5hbGw8LWVmZi5MaW5rX3lvdW5nZXIkZGF0YQ0KcGxvdC5kYXRhLkFnZS5maXQ8LXdpdGgoZWZmLkxpbmtfeW91bmdlcixjYmluZCh4LGZpdCxsb3dlcix1cHBlcikpDQpwbG90KDMsdHlwZT0ibiIseGxpbT1jKC0yLDIuNSkseWxpbT1jKC0zLDIuNSkseGF4dD0nbicsYW5uPUYseWF4dD0nbicpDQpheGlzKHNpZGU9Mixsd2Q9MixjZXguYXhpcz0xLjUpDQpheGlzKHNpZGU9MSxsd2Q9MixjZXguYXhpcz0xLjUsYXQ9YygtMC43MDc1NDczLDAuODA5NDE4OCwxLjYwNDU1NSwyLjE2MjU4NCksbGFiZWxzID0gYyg1MCwxNTAsMjUwLDM1MCkpDQpib3gobHdkPTIpDQoNCiMjbm9kZS5maW48LWxtZShjbG9zZW5lc3NfdH5BZ2VfdCxyYW5kb209fjF8TFcsZGF0YT1Ob2RlKQ0KDQp0aXRsZSh4bGFiPSJBR0VfQkFTRSAoeWVhcikiLHlsYWI9ZXhwcmVzc2lvbihpdGFsaWMocGFzdGUoIkRJRkZfR0VOX0dFTyIpKSksbGluZT0zLGNleC5sYWI9MS41KQ0Kd2l0aChwbG90LmRhdGEuQWdlLmZpdCxwb2x5Z29uKHg9YyhBZ2VfeW91bmdlcl90LCByZXYoQWdlX3lvdW5nZXJfdCkpLHk9Yyhsb3dlciwgcmV2KHVwcGVyKSksDQogICAgICAgICAgICAgICAgICAgICAgICAgICAgICAgY29sPWMoIiNkY2RjZGMiKSwgYm9yZGVyPU5BKSkgDQoNCndpdGgoTGlua1tMaW5rJFNwZWNpZXM9PSJBbmUiLF0sIHBvaW50cyh4PWppdHRlcihBZ2VfeW91bmdlcl90KSx5PURpZmZfR19QX3QscGNoPTIxLGNleD0yLGx3ZD0xLjUsDQogICAgICAgICAgICAgICAgICAgICAgICAgICAgICAgICAgICAgICAgYmc9YygiI2JiNTU2NiIpKSkNCg0Kd2l0aChMaW5rW0xpbmskU3BlY2llcz09Ik94YSIsXSwgcG9pbnRzKHg9aml0dGVyKEFnZV95b3VuZ2VyX3QpLHk9RGlmZl9HX1BfdCxwY2g9MjEsY2V4PTIsbHdkPTEuNSwgDQogICAgICAgICAgICAgICAgICAgICAgICAgICAgICAgICAgICAgICAgYmc9YygiI2RkYWEzMyIpKSkNCndpdGgoTGlua1tMaW5rJFNwZWNpZXM9PSJQb2wiLF0sIHBvaW50cyh4PWppdHRlcihBZ2VfeW91bmdlcl90KSx5PURpZmZfR19QX3QscGNoPTIxLGNleD0yLGx3ZD0xLjUsIA0KICAgICAgICAgICAgICAgICAgICAgICAgICAgICAgICAgICAgICAgIGJnPWMoIiMwMDQ0ODgiKSkpDQoNCndpdGgocGxvdC5kYXRhLkFnZS5maXQsbGluZXMoQWdlX3lvdW5nZXJfdCxmaXQsY29sPXJnYigwLDAsMCxtYXhDb2xvclZhbHVlID0gMjU1KSxsd2Q9MixsdHk9NSkpDQptdGV4dChhZGo9LTAuMiwiKEIpIixjZXg9MS41KQ0KI2xpbmsuZmluMjwtbG1lKERpZmZfR19QX3R+QWdlX0RpZmZfdCxyYW5kb209fjF8TFcueCxjb3JyZWxhdGlvbj1jb3JNTFBFKGZvcm09fm5vZGVfMStub2RlXzJ8TFcueCksZGF0YT1MaW5rKQ0KI21lYW48LW1lYW4oYm94Y294KExpbmskQWdlX0RpZmYpKSMjIGxhbWJkYT0wLjcsIGFkZGVkPTANCiNzZDwtc2QoYm94Y294KExpbmskQWdlX0RpZmYpKQ0KIygoNTBeMC43LTEpLzAuNy1tZWFuKS9zZA0KIygoMTAwXjAuNy0xKS8wLjctbWVhbikvc2QNCiMoKDE1MF4wLjctMSkvMC43LW1lYW4pL3NkDQojKCgyMDBeMC43LTEpLzAuNy1tZWFuKS9zZA0KIygoMjUwXjAuNy0xKS8wLjctbWVhbikvc2QNCg0KZWZmLkxpbmtfeW91bmdlcjwtZWZmZWN0KCJBZ2VfRGlmZl90IixsaW5rLmZpbjIsIHBhcnRpYWwucmVzaWR1YWxzPVQpDQpwbG90LmRhdGEuQWdlLmFsbDwtZWZmLkxpbmtfeW91bmdlciRkYXRhDQpwbG90LmRhdGEuQWdlLmZpdDwtd2l0aChlZmYuTGlua195b3VuZ2VyLGNiaW5kKHgsZml0LGxvd2VyLHVwcGVyKSkNCnBsb3QoMyx0eXBlPSJuIix4bGltPWMoLTEuMywyKSx5bGltPWMoLTMsMi41KSx4YXh0PSduJyxhbm49Rix5YXh0PSduJykNCmF4aXMoc2lkZT0yLGx3ZD0yLGNleC5heGlzPTEuNSkNCmF4aXMoc2lkZT0xLGx3ZD0yLGNleC5heGlzPTEuNSxhdD1jKC0wLjIwMTM3NDMsMC4zMTcxMTkyLDAuNzU5Nzc3NywxLjE1OTQxNCwxLjUyOTgzMiksbGFiZWxzID0gYyg1MCwxMDAsMTUwLDIwMCwyNTApKQ0KYm94KGx3ZD0yKQ0KDQojI25vZGUuZmluPC1sbWUoY2xvc2VuZXNzX3R+QWdlX3QscmFuZG9tPX4xfExXLGRhdGE9Tm9kZSkNCg0KdGl0bGUoeGxhYj0iQUdFX0RJRkYgKHllYXIpIix5bGFiPWV4cHJlc3Npb24oaXRhbGljKHBhc3RlKCJESUZGX0dFTl9HRU8iKSkpLGxpbmU9MyxjZXgubGFiPTEuNSkNCndpdGgocGxvdC5kYXRhLkFnZS5maXQscG9seWdvbih4PWMoQWdlX0RpZmZfdCwgcmV2KEFnZV9EaWZmX3QpKSx5PWMobG93ZXIsIHJldih1cHBlcikpLA0KICAgICAgICAgICAgICAgICAgICAgICAgICAgICAgIGNvbD1jKCIjZGNkY2RjIiksIGJvcmRlcj1OQSkpIA0KDQp3aXRoKExpbmtbTGluayRTcGVjaWVzPT0iQW5lIixdLCBwb2ludHMoeD1qaXR0ZXIoQWdlX0RpZmZfdCkseT1EaWZmX0dfUF90LHBjaD0yMSxjZXg9Mixsd2Q9MS41LA0KICAgICAgICAgICAgICAgICAgICAgICAgICAgICAgICAgICAgICAgIGJnPWMoIiNiYjU1NjYiKSkpDQoNCndpdGgoTGlua1tMaW5rJFNwZWNpZXM9PSJPeGEiLF0sIHBvaW50cyh4PWppdHRlcihBZ2VfRGlmZl90KSx5PURpZmZfR19QX3QscGNoPTIxLGNleD0yLGx3ZD0xLjUsIA0KICAgICAgICAgICAgICAgICAgICAgICAgICAgICAgICAgICAgICAgIGJnPWMoIiNkZGFhMzMiKSkpDQp3aXRoKExpbmtbTGluayRTcGVjaWVzPT0iUG9sIixdLCBwb2ludHMoeD1qaXR0ZXIoQWdlX0RpZmZfdCkseT1EaWZmX0dfUF90LHBjaD0yMSxjZXg9Mixsd2Q9MS41LCANCiAgICAgICAgICAgICAgICAgICAgICAgICAgICAgICAgICAgICAgICBiZz1jKCIjMDA0NDg4IikpKQ0KDQp3aXRoKHBsb3QuZGF0YS5BZ2UuZml0LGxpbmVzKEFnZV9EaWZmX3QsZml0LGNvbD1yZ2IoMCwwLDAsbWF4Q29sb3JWYWx1ZSA9IDI1NSksbHdkPTIsbHR5PTUpKQ0KbXRleHQoYWRqPS0wLjIsIihDKSIsY2V4PTEuNSkNCg0KDQpgYGANCg0K
