# Supplementary material for: Limited effects of population age on the genetic structure of spatially isolated forest herb populations in temperate Europe
Source: Ecol Evol. 2024 Feb 26;14(2):e10971. doi: 10.1002/ece3.10971 (PMC10897356; doi:10.1002/ece3.10971)
Supplement: Supplementary file 1 — Appendix S1. [file ECE3-14-e10971-s001.zip › Supplementary_material_CU_revised.docx]

Supplementary Material

Limited effects of population age on the genetic structure of spatially isolated forest herb populations across Europe

**Siyu Huang*, Jannis Till Feigs, Stephanie I. J. Holzhauer, Katja Kramp, Jörg Brunet, Guillaume Decocq, Pieter De Frenne, Martin Diekmann, Jaan Liira, Fabien Spicher, Pieter Vangansbeke, Thomas Vanneste, Kris Verheyen, Tobias Naaf**

* Correspondence: Siyu Huang: siyu.huang@zalf.de

Contents

[S1 Land cover in studied landscape windows. 2](#_Toc143095389)

[S2 Overview of historical maps 2](#_Toc143095390)

[S3 Source of information for Table 1 in the main text 3](#_Toc143095391)

[S4 Overview of populations sampled with age information 4](#_Toc143095392)

[S5 Number of individuals sampled and raw information of each population 5](#_Toc143095393)

[S6 Population genetics of the forest herb populations 6](#_Toc143095394)

[S7 Interactions between population age and spatial connectivity and population size 6](#_Toc143095395)

[S8 Population graphs 8](#_Toc143095396)

[S9 Interaction between geographical distance and age properties of population pairs. 9](#_Toc143095397)

[S10 Effects of population age on *NHc* and *DIFF_GEN_GEO* 10](#_Toc143095398)

[S11 R code for the analysis 10](#_Toc143095399)

[S12 Results of the linear mixed models used to model genetic diversity and differentiation as a function of population age 11](#_Toc143095400)

# S1 Land cover in studied landscape windows.

Table S1 percentage of area land-use types or length (m/km^2^) of linear land-use types in six landscape windows (5×5km^2^). (Fr: France, Be: Belgium, GeW: Western Germany, GeE: Eastern Germany, Sw: Sweden, Es. Estonia)

| Land-use type (area) | Fr | Be | GeW | GeE | Sw | Es |
| --- | --- | --- | --- | --- | --- | --- |
| Arable land | 18.93 | 37.15 | 61.06 | 58.56 | 75.08 | 40.06 |
| Forest | 11.19 | 8.94 | 12.72 | 18.68 | 4.63 | 27.55 |
| Grassland | 62.52 | 34.78 | 18.28 | 17.73 | 12.57 | 17.37 |
| Settlement | 4.90 | 16.94 | 5.82 | 3.03 | 5.38 | 5.87 |
| Others* | 2.46 | 2.19 | 2.12 | 2 | 2.34 | 9.15 |
| Land-use type (linear) |  |  |  |  |  |  |
| Herbaceous fringe | 1371 | 621 | 1269 | 2705 | 3952 | 1585 |
| Traffic lines | 9127 | 13303 | 8250 | 10901 | 8416 | 7245 |
| Drainage ditch or water | 2673 | 3052 | 4647 | 7156 | 4122 | 4627 |
| Woody element | 30616 | 4324 | 8346 | 8775 | 9438 | 1441 |

*****other land-use types include occasionally orchards, water bodies etc.

# S2 Overview of historical maps

Table S2 Series of historical maps from six landscape windows (Fr: France, Be: Belgium, GeW: Western Germany, GeE: Eastern Germany, Sw: Sweden, Es. Estonia) for determining age of forest patches as well as forest herb populations. The names of the maps are kept as original as possible so that they could be easily found. The numbers in the parentheses indicate the time when the map emerged. If there is no map available for this time and landscape window, this is indicated by “-“.

| Fr | Be | GeW | GeE | Sw | Es |
| --- | --- | --- | --- | --- | --- |
| Carte de Cassini (1760-1815) | Ferrariskaarten (1770-1778) | Kurhannoversche Landesaufnahme (1765-1770) | Schmettau’sche Karte (ca. 1780) | Skånska rekognosceringskartan (1812-1820) | Fempte deels transporterad charta öfwer första deelen aff Dörpts lähn (1684) |
| Carte d’Etat-Major (1818-1881) | Vandermaelenkaarten (1846-1854) | - | Preußische Uraufnahme (1825-1843) | Generalstabskartan (1864-1865) | - |
| - | - | Preußische Landesaufnahme (ca. 1900) | Preußische Landesaufnahme (ca. 1880) | Ekonomiska kartan (1915) | One-verst map of Estonia, Russian Empire (ca.1900) |
| Aerial photograph (1953-1954) | Carte topo  (1910-1940) | Aerial photograph (1963) | Aerial photograph (1953) | Aerial photograph (1947) | Estonian topographic map (1935-1939) |
| Aerial photograph | Aerial photograph (2000) | Aerial photograph (1987) | Aerial photograph (1985/91) | Aerial photograph (1986) | Topographic maps of soviet union (1947-1969) / Estonian Land Board Collection of historical aerial photographs and photomaps (1948-1991) |

# S3 Source of information for Table 1 in the main text

1. Baumberger, H. (1971). Chromosomenzahlbestimmungen und Karyotypanalysen bei den Gattungen Anemone, Hepatica und Pulsatilla. Ber Schweiz Bot Ges 80:17-95.
2. Berg, H. (2000). Differential seed dispersal in Oxalis acetosella, a cleistogamous perennial herb. Acta Oecol 21(2):109-118. <https://doi.org/10.1016/s1146-609x(00)00118-1>
3. Berg, H.; Redbo-Torstensson, P. (1998) Cleistogamy as a bet-hedging strategy in Oxalis acetosella, a perennial herb. J Ecol 86(3):491-500. <https://doi.org/10.1046/j.1365-2745.1998.00272.x>
4. Knuth, PEOW; Appel, O; Loew, E; Müller, H (1898). Handbuch der Blütenbiologie, Band 2. W. Engelmann, Leipzig
5. Kosiński, I. (2012). Generative reproduction dynamics in populations of the perennial herb Polygonatum multiflorum (Asparagaceae). Ann Bot Fenn 49(4):217-228. <https://doi.org/10.5735/085.049.0401>
6. Kosiński, I. (2015). Ontogenetic development and maturity of individuals of Polygonatum multiflorum. Flora 216:1-5. <https://doi.org/10.1016/j.flora.2015.08.002>
7. Müller, N; Schneller, J.J.; Holderegger, R. (2000). Variation in breeding system among populations of the common woodland herb Anemone nemorosa (Ranunculaceae). Plant Syst Evol 221(1-2):69-76. <https://doi.org/10.1007/bf01086381>
8. Packham, J.R. (1978). Biological flora of the British isles - Oxalis acetosella L. J Ecol 66(2):669-693. <https://doi.org/10.2307/2259158>
9. Redbo-Torstensson, P; Berg, H (1995). Seasonal cleistogamy - a conditional strategy to provide reproductive assurance. Acta Bot Neerl 44(3):247-256.
10. Shirreffs, D. A. (1985). Biological flora of the British isles - Anemone nemorosa L. J Ecol 73(3):1005-1020. <https://doi.org/10.2307/2260164>
11. Strickmann, M. (2008). Untersuchung zur Bestäubungsbiologie und Samenproduktion an Anemone nemorosa in Brandenburg. Master thesis, University of Münster, Germany
12. Naaf, T. (2021). Sensitivity to habitat fragmentation across European landscapes in three temperate forest herbs. Landscape Ecology 36 (10), 2831–2848. <https://doi.org/10.1007/s10980-021-01292-w>. Appendix S1.

# S4 Overview of populations sampled with age information

Figure S4 Six 5×5 km^2^ landscape windows with indicated locations of the sampled populations of the three forest herb species: A: *Anemone nemorosa*, O: *Oxalis acetosella*, P: *Polygonatum multiflorum*. Notation at the top of each panel indicates the names of the landscape window (Fr: France, Be: Belgium, GeW: Western Germany, GeE: Eastern Germany, Sw: Sweden, Es. Estonia). The time scale on the right defines the minimum age of forest patches according to historical maps (Table S1). Two *O. acetosella* populations and one *P. multiflorum* population in Sweden were sampled out of the landscape window. The location of these populations are indicated in the small legend at the bottom.


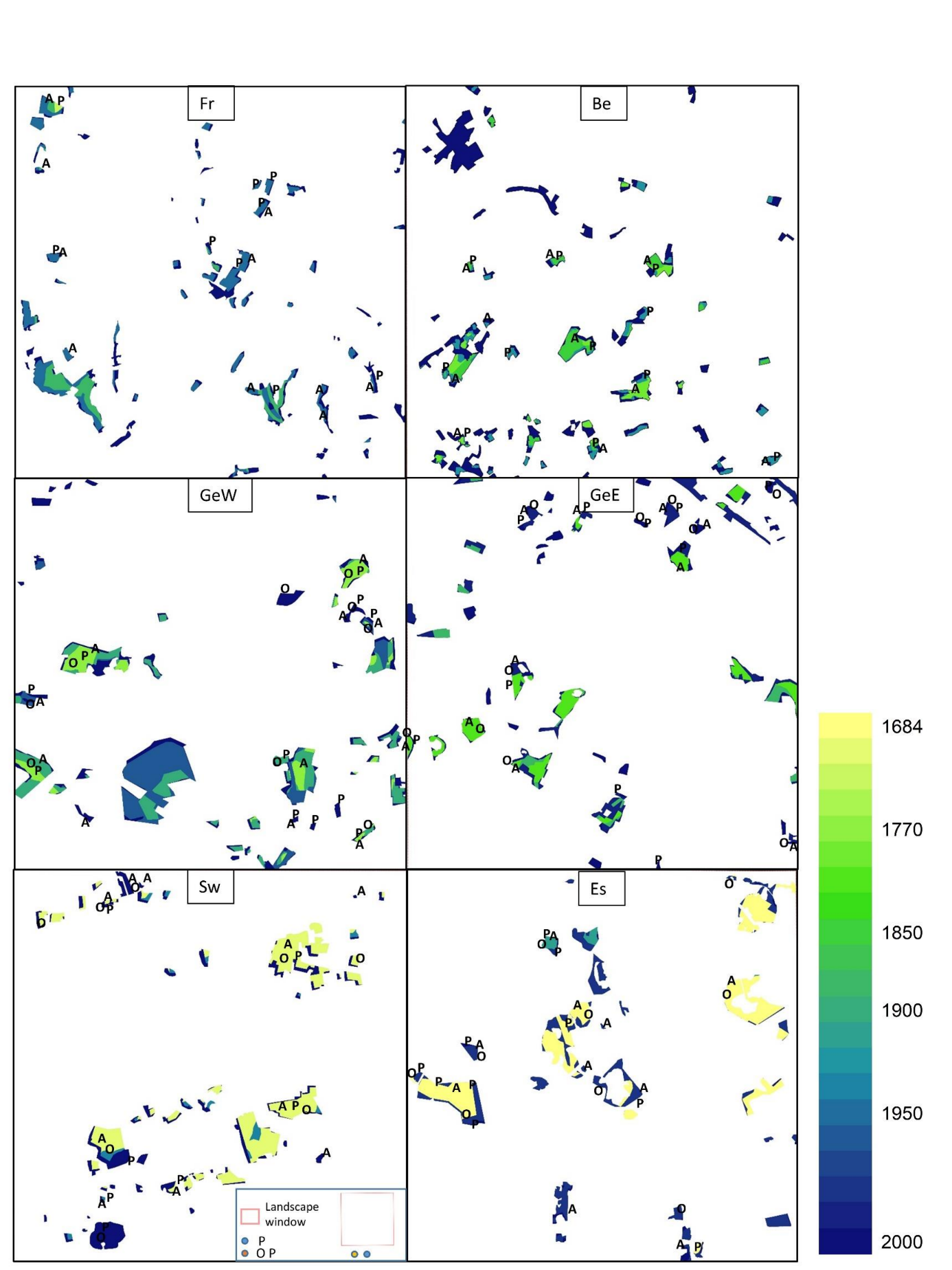

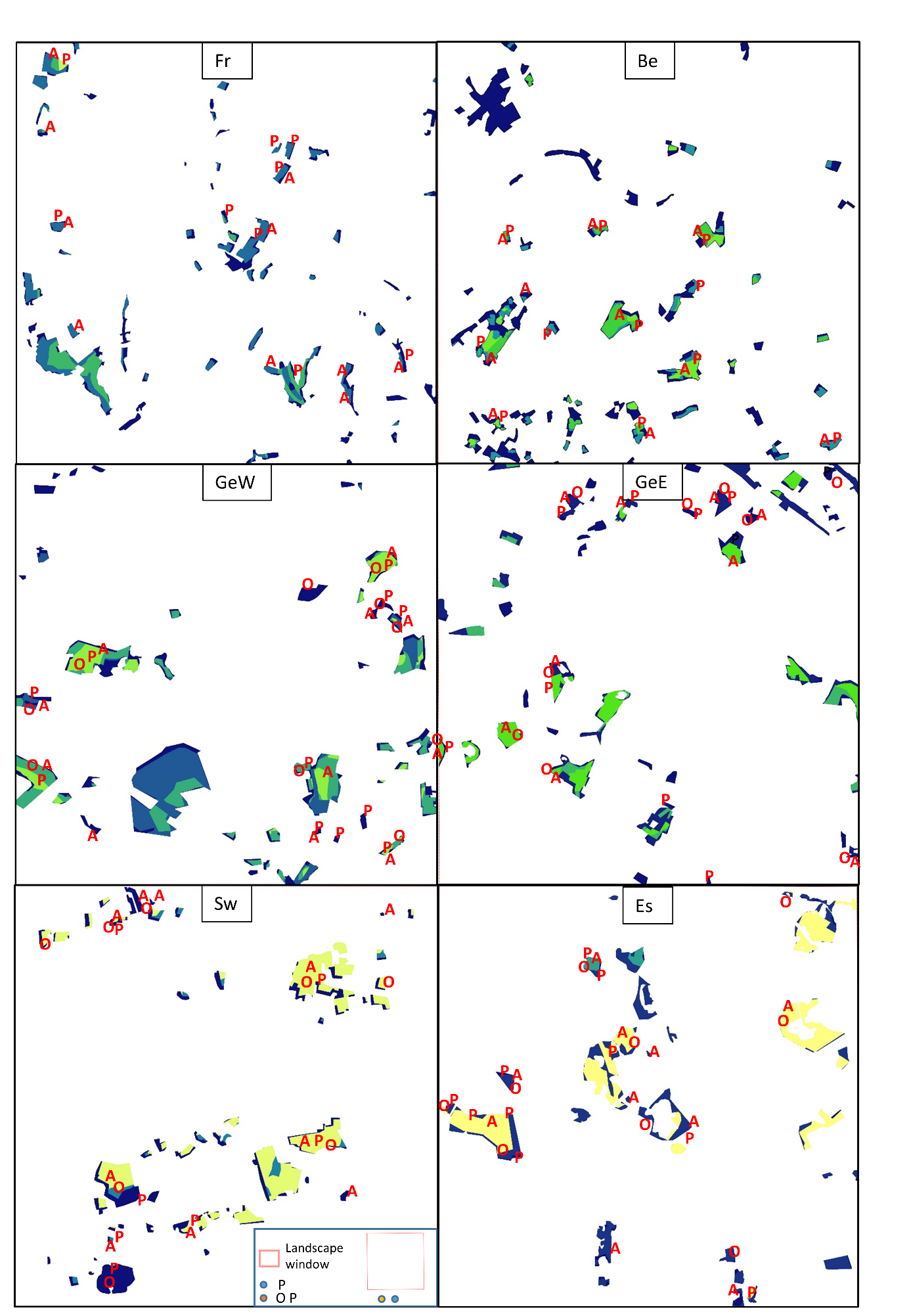


# S5 Number of individuals sampled and raw information of each population

An excel file “Gen_Div_all.xlsx” comprises information on attributes and calculated genetic diversity measures of all populations included in the analysis. Also, the information on the used microsatellite markers and the allele tables of *A. nemorosa*, *O. acetosella* and *P. multiflorum* are included.

# S6 Population genetics of the forest herb populations

Table S6 Allelic richness (*A_r_*), expected heterozygosity (*H_e_*), observed heterozygosity (*H_o_*) and inbreeding coefficient (*F*) as well as pairwise genetic differentiation measures (*G”_ST_*, *D_PS_*, *cGD*) of the populations of the three studied species (*A. nem.*: *Anemone nemorosa*: *O. ace.*: *Oxalis acetosella*; *P. mul.*: *Polygonatum multiflorum*) at the landscape windows (LW) (Fr: France, Be: Belgium, GeW: Western Germany, GeE: Eastern Germany, Sw: Sweden, Es: Estonia). Given are mean ± standard deviation as well as the average (Avg.) calculated from raw data.

|  | *LW* | *A_r_* | *H_e_* | *H_o_* | *F* | *G”_ST_* | | *D_PS_* | *cGD* |
| --- | --- | --- | --- | --- | --- | --- | --- | --- | --- |
| *A. nem.* | Fr | 5.51±1.27 | 0.71±0.04 | 0.58±0.08 | 0.18±0.10 | 0.34±0.13 | | 0.44±0.09 | 8.03±2.31 |
|  | Be | 6.90±0.52 | 0.73±0.02 | 0.62±0.08 | 0.15±0.10 | | 0.24±0.08 | 0.38±0.05 | 8.82±1.86 |
|  | GeW | 7.92±0.52 | 0.70±0.07 | 0.62±0.11 | 0.10±0.23 | 0.08±0.03 | | 0.27±0.03 | 5.23±0.45 |
|  | GeE | 8.20±0.56 | 0.70±0.05 | 0.62±0.05 | 0.10±0.11 | 0.05±0.03 | | 0.24±0.03 | 5.16±0.51 |
|  | Sw | 8.92±0.55 | 0.73±0.02 | 0.54±0.06 | 0.26±0.09 | 0.05±0.03 | | 0.25±0.04 | 5.32±0.83 |
|  | Es | 6.61±0.69 | 0.73±0.04 | 0.55±0.11 | 0.23±0.18 | 0.10±0.05 | | 0.27±0.05 | 5.16±1.09 |
|  | *Avg.* | 7.34±1.34 | 0.72±0.04 | 0.59±0.09 | 0.17±0.15 | 0.14±0.13 | | 0.31±0.09 | 6.31±2.05 |
| *O. ace.* | GeW | 2.38±0.21 | 0.39±0.05 | 0.36±0.08 | 0.09±0.17 | 0.14±0.09 | | 0.18±0.05 | 3.02±0.73 |
|  | GeE | 2.19±0.28 | 0.36±0.09 | 0.29±0.10 | 0.19±0.13 | 0.23±0.13 | | 0.24±0.07 | 3.65±1.16 |
|  | Sw | 2.42±0.55 | 0.43±0.09 | 0.45±0.08 | -0.05±0.21 | 0.25±0.13 | | 0.27±0.07 | 3.83±0.52 |
|  | Es | 2.51±0.29 | 0.40±0.06 | 0.34±0.05 | 0.13±0.15 | 0.15±0.12 | | 0.21±0.08 | 3.17±1.06 |
|  | *Avg.* | 2.37±0.36 | 0.39±0.08 | 0.36±0.09 | 0.09±0.18 | 0.19±0.13 | | 0.23±0.07 | 3.44±0.97 |
| *P. mul.* | Fr | 7.58±1.78 | 0.72±0.05 | 0.70±0.08 | 0.03±0.10 | 0.26±0.10 | | 0.52±0.07 | 8.34±1.88 |
|  | Be | 6.70±1.54 | 0.72±0.04 | 0.75±0.07 | -0.04±0.07 | 0.28±0.11 | | 0.53±0.06 | 9.40±1.57 |
|  | GeW | 8.14±1.30 | 0.73±0.04 | 0.72±0.07 | 0.02±0.07 | 0.21±0.09 | | 0.46±0.06 | 6.62±1.01 |
|  | GeE | 7.00±1.69 | 0.70±0.05 | 0.72±0.09 | -0.02±0.08 | 0.34±0.11 | | 0.51±0.06 | 8.06±1.61 |
|  | Sw | 7.61±0.81 | 0.73±0.02 | 0.82±0.05 | -0.12±0.07 | 0.26±0.08 | | 0.45±0.06 | 8.08±1.48 |
|  | Es | 5.32±1.76 | 0.67±0.08 | 0.63±0.09 | 0.05±0.10 | 0.29±0.13 | | 0.53±0.07 | 8.77±2.67 |
|  | *Avg.* | 7.06±1.72 | 0.71±0.05 | 0.72±0.09 | -0.01±0.10 | 0.27±0.11 | | 0.50±0.07 | 8.15±1.87 |

# S7 Interactions between population age and spatial connectivity and population size

Table S7.1 Interactive partial effects between population size (POP_SIZE) and population age (POP_AGE) as well as spatial connectivity (SPA_CON) and population age (POP_AGE) of ***Anemone nemorosa*** on genetic diversity. Given are standardized regression coefficients. Significance of regression coefficients is indicated by asterisks: *^n.s.^* *p* > 0.1; ^(*)^ *p* ≤ 0.1; ^*^ *p*≤ 0.05;^**^ *p* ≤ 0.01.

|  | ***A_r_*** | ***H_e_*** | ***H_o_*** | ***F*** |  |
| --- | --- | --- | --- | --- | --- |
| POP_SIZE | 0.08 ^n.s.^ | -0.12 ^n.s.^ | 0.44 ^(*).^ | -0.38 ^n.s.^ | |
| SPA_CON | 0.18 ^n.s.^ | 0.21 ^n.s.^ | -0.52 ^*^ | 0.52 ^*^ | |
| POP_AGE | -0.08 ^n.s.^ | -0.59 ^**^ | 0.14 ^n.s.^ | -0.30 ^(*)^ | |
| POP_SIZE:POP_AGE | -0.12 ^n.s.^ | -0.11 ^n.s.^ | 0.13 ^n.s.^ | -0.16 ^n.s.^ | |
| SPA_CON:POP_AGE | -0.03 ^n.s.^ | 0.33 ^n.s.^ | -0.30 ^n.s.^ | 0.36 ^n.s.^ | |
| Marginal *R^2^* | 0.06 | 0.30 | 0.11 | 0.17 | |
| Conditional *R^2^* | 0.72 | 0.44 | 0.11 | 0.22 | |

Table S7.2 Interactive partial effects between population size (POP_SIZE) and population age (POP_AGE) as well as spatial connectivity (SPA_CON) and population age (POP_AGE) of ***Oxalis acetosella*** on genetic diversity. Given are standardized regression coefficients. Significance of regression coefficients is indicated by asterisks: *^n.s.^* *p* > 0.1; ^(*)^ *p* ≤ 0.1; ^*^ *p* ≤ 0.05;^**^ *p* ≤ 0.01. Significant interactions are marked in bold.

|  | ***A_r_*** | ***H_e_*** | ***H_o_*** | ***F*** |
| --- | --- | --- | --- | --- |
| POP_SIZE | 0.26 ^n.s.^ | 0.23 ^n.s.^ | 0.21 ^n.s.^ | -0.05 ^n.s.^ |
| SPA_CON | 0.23 ^n.s.^ | 0.08 ^n.s.^ | 0.11 ^n.s.^ | -0.02 ^n.s.^ |
| POP_AGE | -0.01 ^n.s.^ | -0.14 ^n.s.^ | -0.08 ^n.s.^ | 0.00 ^n.s.^ |
| POP_SIZE:POP_AGE | 0.27 ^n.s.^ | 0.34 ^n.s.^ | **0.43 ^*^** | -0.32 ^n.s.^ |
| SPA_CON:POP_AGE | -0.35 ^n.s.^ | **-0.51 ^(*)^** | -0.24 ^n.s.^ | -0.09 ^n.s.^ |
| Marginal *R^2^* | 0.26 | 0.17 | 0.15 | 0.12 |
| Conditional *R^2^* | 0.33 | 0.28 | 0.58 | 0.39 |

Table S7.3 Interactive partial effects between population size (POP_SIZE) and population age (POP_AGE) as well as spatial connectivity (SPA_CON) and population age (POP_AGE) of ***Polygonatum multiflorum*** on genetic diversity. Given are standardized regression coefficients. Significance of regression coefficients is indicated by asterisks: *^n.s.^* *p* > 0.1; ^(*)^ *p*≤ 0.1; ^*^ *p* ≤ 0.05;^**^ *p* ≤ 0.01. Significant interactions are marked in bold.

|  | ***A_r_*** | ***H_e_*** | ***H_o_*** | ***F*** |
| --- | --- | --- | --- | --- |
| POP_SIZE | 0.43 ^**^ | 0.06 ^n.s.^ | -0.09 ^n.s.^ | 0.21 ^n.s.^ |
| SPA_CON | 0.26 ^(*)^ | 0.26 ^n.s.^ | 0.15 ^n.s.^ | -0.06 ^n.s.^ |
| POP_AGE | 0.11 ^n.s.^ | 0.16 ^n.s.^ | 0.40 ^**^ | -0.38 ^*^ |
| POP_SIZE:POP_AGE | -0.00 ^n.s.^ | **-0.38 ^*^** | -0.18 ^n.s.^ | 0.02 ^n.s.^ |
| SPA_CON:POP_AGE | 0.18 ^n.s.^ | 0.18 ^n.s.^ | -0.01 ^n.s.^ | 0.08 ^n.s.^ |
| Marginal *R^2^* | 0.39 | 0.20 | 0.17 | 0.13 |
| Conditional *R^2^* | 0.49 | 0.20 | 0.50 | 0.47 |

# S8 Population graphs

Figure S8 Population graph of populations of the three species (*A.nem.*: *Anemone nemorosa*; *O.ace.*: *Oxalis acetosella*; *P.mul.*: *Polygonatum multiflorum*) at the six landscape windows (Fr: France, Be: Belgium, GeW: Western Germany, GeE: Eastern Germany, Sw: Sweden, Es: Estonia). The size of the node represents the sample size.


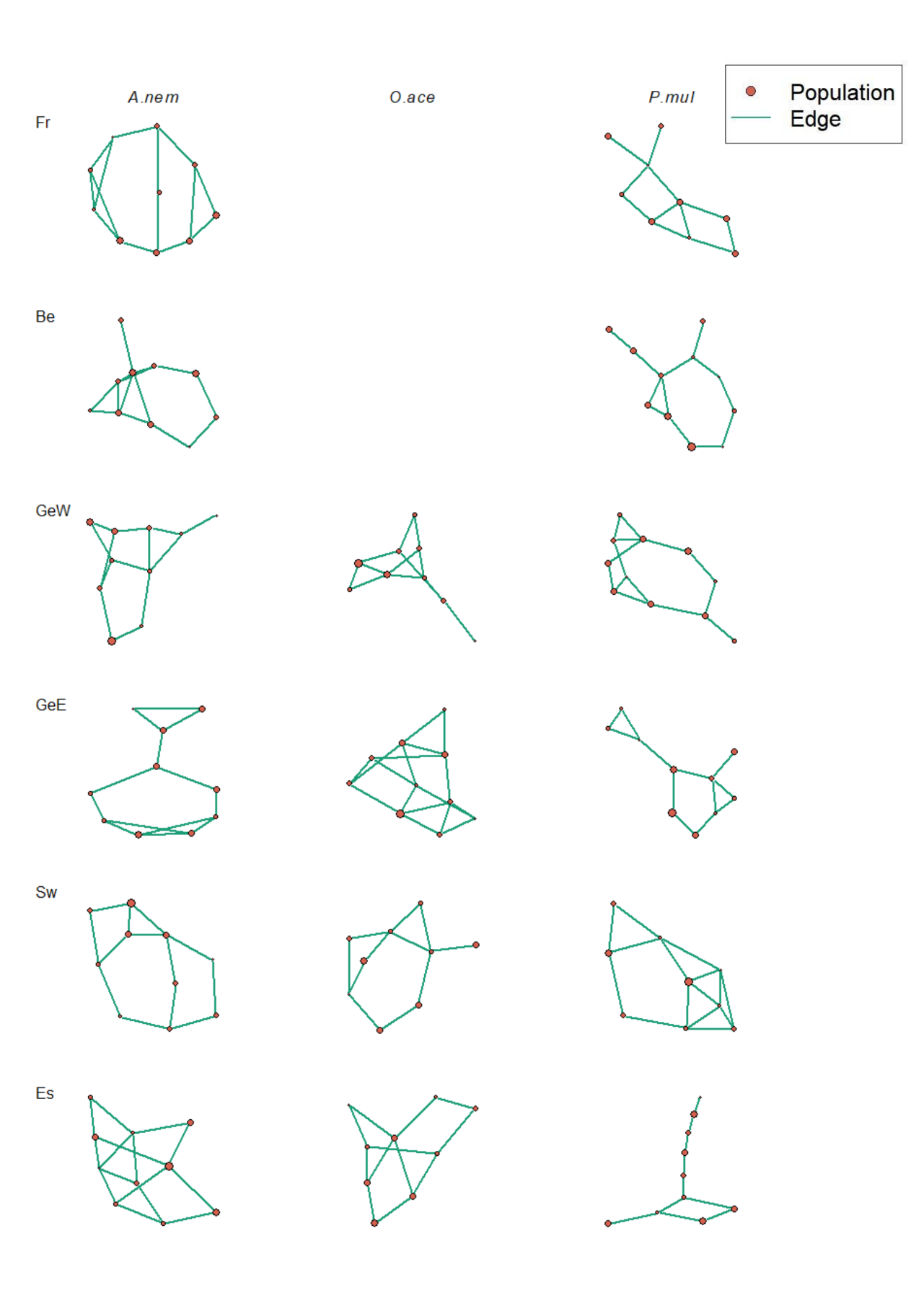


# S9 Interaction between geographical distance and age properties of population pairs.

Table S9.1 Interactive effects between geographical distance (GEO_DIST) and age properties of population pairs (AGE_BASE, AGE_DIFF) on genetic differentiation in ***Anemone nemorosa***. Significance of regression coefficient is indicated by asterisks: *^n.s.^* *p* > 0.1; ^(*)^ *p* ≤ 0.1; ^*^ *p* ≤ 0.05;^**^ *p* ≤ 0.01. Significant interactions are marked in bold.

|  | ***G”_ST_*** | ***D_PS_*** | ***cGD*** |
| --- | --- | --- | --- |
| GEO_DIST | 0.00 ^n.s.^ | 0.04 ^n.s.^ | 0.02 ^n.s.^ |
| AGE_DIFF | -0.04 ^n.s.^ | -0.02 ^n.s.^ | 0.01 ^n.s.^ |
| AGE_BASE | -0.17 ^*^ | -0.19 ^**^ | -0.25 ^*^ |
| GEO_DIST:AGE_DIFF | -0.05 ^n.s.^ | -0.05 ^n.s.^ | 0.04 ^n.s.^ |
| GEO_DIST:AGE_BASE | **-0.06 ^(*)^** | **-0.07 ^(*)^** | -0.08 ^n.s.^ |
| Marginal *R^2^* | 0.02 | 0.03 | 0.07 |
| Conditional *R^2^* | 0.74 | 0.70 | 0.67 |

Table S9.2 Interactive effects between geographical distance (GEO_DIST) and age properties of population pairs (AGE_BASE, AGE_DIFF) on genetic differentiation in ***Oxalis acetosella***. Significance of regression coefficient is indicated by asterisks: *^n.s.^* *p* > 0.1; ^(*)^ *p* ≤ 0.1; ^*^ *p* ≤ 0.05;^**^ *p* ≤ 0.01. Significant interactions are marked in bold.

|  | ***G”_ST_*** | ***D_PS_*** | ***cGD*** |
| --- | --- | --- | --- |
| GEO_DIST | 0.01 ^n.s.^ | 0.08 ^n.s.^ | 0.02 ^n.s.^ |
| AGE_DIFF | -0.11 ^n.s.^ | -0.12 ^n.s.^ | -0.26 ^*^ |
| AGE_BASE | -0.23 ^n.s.^ | -0.27 ^(*)^ | -0.28^n.s.^ |
| GEO_DIST:AGE_DIFF | 0.03 ^n.s.^ | 0.05^n.s.^ | 0.04 ^n.s.^ |
| GEO_DIST:AGE_BASE | 0.02 ^n.s.^ | 0.04 ^n.s.^ | **0.10 ^(*)^** |
| Marginal *R^2^* | 0.03 | 0.06 | 0.07 |
| Conditional *R^2^* | 0.03 | 0.10 | 0.07 |

Table S9.3 Interactive effects between geographical distance (GEO_DIST) and age properties of population pairs (AGE_BASE, AGE_DIFF) on genetic differentiation in ***Polygonatum multiflorum***. Significance of regression coefficient is indicated by asterisks: *^n.s.^* *p* > 0.1; ^(*)^ *p* ≤ 0.1; ^*^ *p* ≤ 0.05;^**^ *p* ≤ 0.01. Significant interactions are marked in bold.

|  | ***G”_ST_*** | ***D_PS_*** | ***cGD*** |
| --- | --- | --- | --- |
| GEO_DIST | 0.11 ^*^ | 0.14 ^**^ | 0.10 ^*.^ |
| AGE_DIFF | -0.01 ^n.s.^ | 0.00 ^n.s.^ | -0.04 ^n.s.^ |
| AGE_BASE | -0.01 ^n.s.^ | -0.07 ^n.s.^ | 0.19 ^n.s.^ |
| GEO_DIST:AGE_DIFF | -0.01 ^n.s.^ | -0.03 ^n.s.^ | -0.01 ^n.s.^ |
| GEO_DIST:AGE_BASE | **-0.09 ^*^** | **-0.09 ^(*).^** | -0.06 ^n.s.^ |
| Marginal *R^2^* | 0.02 | 0.03 | 0.07 |
| Conditional *R^2^* | 0.04 | 0.22 | 0.09 |

# S10 Effects of population age on *NHc* and *DIFF_GEN_GEO*


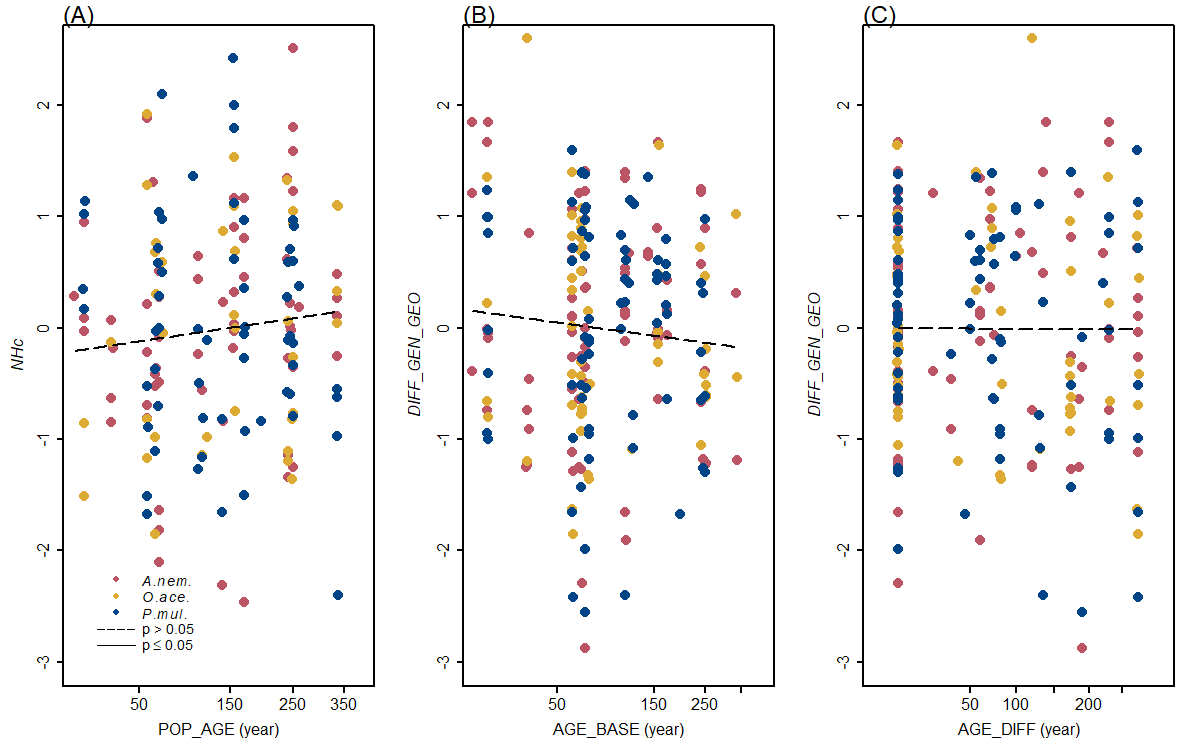


**Figure S10** Effects of age-related variables on population graph topological measures. (A) Effect of population age (POP_AGE) on normalized harmonic centrality (*NHc*). Effect of the (B) age of the younger population in a population pair (AGE_BASE) and (C) age difference of the two population in a population pair (AGE_DIFF) on the correspondence of genetic and geographic distance (DIFF_GEN_GEO).

# S11 R code for the analysis

The R code comprises seven R notebook files. The function of the code is indicated by its name.

01_Data_Preparation

02_Genetic_Diversity

03_Genetic_Differentiation

04_Population_Graph

05_Preparation_For_Linear_Regression

06_Linear_Regression

07_Result_Visualization

# S12 Results of the linear mixed models used to model genetic diversity and differentiation as a function of population age

Table S12 Complete results of the linear mixed models X ~ (POP_SIZE + SPA_CON + POP_AGE) * SPECIES after model selection, where X is a response variable of genetic diversity (*A_r_*, *H_e_*, *H_o_*, *F*) or genetic differentiation (*G”_ST_*, *D_PS_*, *cGD*). Given are the standardized regression slopes (coef), the standard error (SE), the degrees of freedom (df), the T-statistic and the P-value of a certain explanatory variable. If SPECIES occurs in an interaction term, also the differences (∆) in slope among species are given.

|  |  | *coef* | *SE* | *df* | *T* | *P* |
| --- | --- | --- | --- | --- | --- | --- |
| *A_r_* | SPA_CON | 0.36 | 0.09 | 66 | 3.99 | 0.00 |
|  | POP_SIZE | 0.26 | 0.09 | 66 | 2.30 | 0.00 |
|  | POP_AGE Ane | -0.25 | 0.11 | 66 | -2.38 | 0.02 |
|  | POP_AGE Oxa | -0.01 | 0.13 | 66 | -0.06 | 0.95 |
|  | POP_AGE Pol | 0.16 | 0.11 | 66 | 1.50 | 0.14 |
|  | *∆*POP_AGE Ane-Oxa | 0.24 | 0.16 | 66 | 1.56 | 0.12 |
|  | *∆*POP_AGE Ane-Pol | 0.41 | 0.15 | 66 | 2.83 | 0.01 |
|  | *∆*POP_AGE Oxa-Pol | 0.17 | 0.17 | 66 | 1.01 | 0.31 |
|  |  |  |  |  |  |  |
| *H_e_* | SPA_CON | 0.24 | 0.08 | 67 | 2.90 | 0.01 |
|  | POP_AGE Ane | -0.57 | 0.12 | 67 | -4.71 | 0.00 |
|  | POP_AGE Oxa | -0.09 | 0.15 | 67 | -0.56 | 0.57 |
|  | POP_AGE Pol | 0.16 | 0.13 | 67 | 1.25 | 0.22 |
|  | *∆*POP_AGE Ane-Oxa | -0.49 | 0.19 | 67 | 2.60 | 0.01 |
|  | *∆*POP_AGE Ane-Pol | -0.73 | 0.17 | 67 | 4.23 | 0.00 |
|  | *∆*POP_AGE Oxa-Pol | -0.24 | 0.20 | 67 | 1.24 | 0.22 |
|  |  |  |  |  |  |  |
| *H_o_* | POP_AGE | 0.26 | 0.08 | 67 | 3.25 | 0.00 |
|  | SPA_CON Ane | -0.33 | 0.13 | 67 | -2.49 | 0.02 |
|  | SPA_CON Oxa | 0.07 | 0.16 | 67 | 0.47 | 0.64 |
|  | SPA_CON Pol | 0.18 | 0.13 | 67 | 1.39 | 0.17 |
|  | *∆*SPA_CON Ane-Oxa | 0.41 | 0.20 | 67 | 2.05 | 0.04 |
|  | *∆*SPA_CON Ane-Pol | 0.51 | 0.19 | 67 | 2.75 | 0.01 |
|  | *∆*SPA_CON Oxa-Pol | 0.10 | 0.20 | 67 | 0.50 | 0.62 |
|  |  |  |  |  |  |  |
| *F* | SPA_CON | 0.16 | 0.08 | 69 | 1.89 | 0.06 |
|  | POP_AGE | -0.31 | 0.08 | 69 | -3.83 | 0.00 |
|  |  |  |  |  |  |  |
| *G”_ST_* | AGE_BASE | -0.05 | 0.04 | 683 | -1.50 | 0.13 |
|  | GEO_DIST Ane | 0.01 | 0.04 | 683 | 0.27 | 0.79 |
|  | GEO_DIST Oxa | 0.03 | 0.05 | 683 | 0.60 | 0.55 |
|  | GEO_DIST Pol | 0.13 | 0.04 | 683 | 3.32 | 0.00 |
|  | *∆*GEO_DIST Ane-Oxa | 0.01 | 0.06 | 683 | 0.30 | 0.76 |
|  | *∆*GEO_DIST Ane-Pol | 0.12 | 0.05 | 683 | 2.30 | 0.02 |
|  | *∆*GEO_DIST Oxa-Pol | 0.11 | 0.06 | 683 | 1.76 | 0.08 |
|  |  |  |  |  |  |  |
| *D_PS_* | AGE_BASE | -0.11 | 0.03 | 685 | -2.96 | 0.00 |
|  | GEO_DIST | 0.09 | 0.02 | 685 | 3.84 | 0.00 |
|  |  |  |  |  |  |  |
| *cGD* | GEO_DIST | 0.07 | 0.03 | 196 | 1.99 | 0.05 |
|  | AGE_BASE Ane | -0.28 | 0.08 | 196 | -3.58 | 0.00 |
|  | AGE_BASE Oxa | -0.05 | 0.09 | 196 | -0.58 | 0.57 |
|  | AGE_BASE Pol | 0.21 | 0.10 | 196 | 2.08 | 0.04 |
|  | *∆*AGE_BASE Ane-Oxa | 0.22 | 0.12 | 196 | 1.88 | 0.06 |
|  | *∆*AGE_BASE Ane-Pol | -0.48 | 0.13 | 196 | 3.86 | 0.00 |
|  | *∆*AGE_BASE Oxa-Pol | -0.26 | 0.14 | 196 | 1.93 | 0.06 |
